# Catalytic Asymmetric Synthesis of Cyclohexanes by Hydrogen Borrowing Annulations

**DOI:** 10.1002/anie.201907514

**Published:** 2019-08-07

**Authors:** Roly J. Armstrong, Wasim M. Akhtar, Tom A. Young, Fernanda Duarte, Timothy J. Donohoe

**Affiliations:** ^1^ Chemistry Research Laboratory University of Oxford Oxford OX1 3TA UK

**Keywords:** asymmetric catalysis, enantioselectivity, hydrogen borrowing, iridium

## Abstract

Hydrogen borrowing catalysis serves as a powerful alternative to enolate alkylation, enabling the direct coupling of ketones with unactivated alcohols. However, to date, methods that enable control over the absolute stereochemical outcome of such a process have remained elusive. Here we report a catalytic asymmetric method for the synthesis of enantioenriched cyclohexanes from 1,5‐diols via hydrogen borrowing catalysis. This reaction is mediated by the addition of a chiral iridium(I) complex, which is able to impart high levels of enantioselectivity upon the process. A series of enantioenriched cyclohexanes have been prepared and the mode of enantioinduction has been probed by a combination of experimental and DFT studies.

Enolate alkylation is a fundamental process in organic chemistry and is widely used as a strategy for C−C bond formation.^1^ In this chemistry a carbonyl substrate is typically deprotonated with a strong base (e.g., LDA) and the resulting enolate is then trapped with a reactive electrophile. Alkylation of a substituted enolate results in the generation of a new α‐stereogenic center and an abundance of methods (both stoichiometric and catalytic) have been developed which enable this process to be carried out in an asymmetric manner (Scheme 1 A).^2^ Whilst this approach is highly effective for alkylation with primary electrophiles, alkylation with secondary electrophiles is significantly more challenging and often results in sluggish reactivity accompanied by competing elimination processes.^1^ Moreover, when unsymmetrical secondary electrophiles are employed, a new stereogenic center is formed at the β‐position and only a handful of methods have been reported, which allow control over the stereochemical outcome of such a process.^3^

**Scheme 1:**
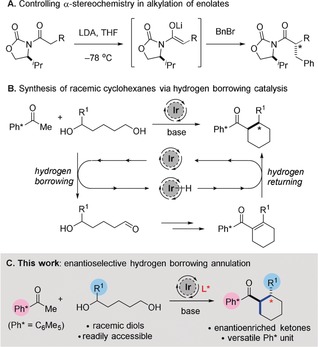
Previous work and strategy for catalytic asymmetric hydrogen borrowing. LDA=lithium diisopropylamide; THF=tetrahydrofuran.

Hydrogen borrowing catalysis represents a powerful alternative strategy to classical enolate alkylation, enabling direct alkylation of enolates with unactivated alcohols.^4^ Within this manifold, we recently reported that an achiral iridium(III) catalyst can promote alkylation of pentamethylphenyl (Ph*) ketones with alcohols leading to α‐ and β‐branched ketones.^5^ This was subsequently extended to a (5+1) annulation process in which racemic cyclohexanes could be accessed from 1,5‐diols (Scheme 1 B).^6^ These reactions proceed by oxidation of the alcohol by the iridium catalyst to generate the corresponding carbonyl compound in situ. After aldol condensation with an enolate and loss of water, the catalyst “returns” the abstracted hydrogen to provide the C−C coupled product and complete the catalytic cycle. The Ph* group plays a key role in facilitating this chemistry; the bulky doubly *ortho*‐substituted aromatic group is oriented orthogonal to the carbonyl and shields against competing reduction and homodimerization processes.^5^ Moreover, acyl Ph* derivatives can readily be converted to a wide range of functional groups via an *ipso*‐substitution process (>30 examples).^5^, ^6^ Remarkably, despite numerous recent advances in the field of enolate hydrogen borrowing catalysis, no general strategy has been reported allowing the absolute stereochemical outcome of this process to be controlled.^7^, ^8^ We rationalized that the enantiodetermining step in these reactions involves the return of iridium hydride to an achiral enone. Since this step bears some resemblance to existing methods for asymmetric hydrogenation we anticipated that a chiral transition‐metal complex might be able to control the facial selectivity of this process (Scheme 1 C).^9^ We recognized that the key to success would lie in identifying a transition metal complex that can perform three key roles: (i) efficient oxidation of alcohols; (ii) a challenging reduction of sterically demanding Ph* substituted enones; (iii) controlling facial selectivity within this reduction process resulting in high levels of enantioselectivity.

We commenced our study by investigating the reaction between pentamethylacetophenone **1** and commercially available hexane‐1,5‐diol **2 a**. In line with our previous studies,^6^ in the presence of an achiral Ir^III^ catalyst along with 4 equiv of KO^*t*^Bu in toluene at 110 °C we obtained racemic cyclohexane **3 a** in 75 % yield and 91:9 d.r. (Table 1, Entry 1). We have previously shown that the high *trans*‐diastereoselectivity in this reaction is a result of reversible deprotonation of the product.^6^ We were delighted to find that by switching to an Ir^I^ precatalyst along with 5 mol % (*R*)‐BINAP (**4**) we obtained cyclohexane **3 a** in 76 % yield with a modest but promising 68:32 e.r. (Table 1, Entry 2). At this point we embarked upon an extensive program of optimization (for full details, see Supporting Information). Changing the ligand to (*R*)‐H_8_‐BINAP (**5**) resulted in lower enantioselectivity whereas (*R*)‐MeO‐BIPHEP (**6**) afforded **3 a** with similar selectivity (Entries 3,4). We next evaluated a series of MeO‐BIPHEP based ligands (**6**–**10**) bearing phosphine groups with different steric and electronic properties. Difuryl‐substituted phosphine **7** resulted in a significant decrease in enantioselectivity, but when a 3,4,5‐trimethoxy substituted ligand **8** was employed, **3 a** was isolated in an improved 73:27 e.r. (Table 1, Entries 5, 6). Increasing the steric bulk of the phosphine clearly provided a beneficial effect—ligands **9** and **10** afforded **3 a** in improved selectivities of 86:14 and 87:13 e.r. respectively (Table 1, Entries 7, 8).

**Table 1:** Optimization of an enantioselective hydrogen borrowing reaction.^[a]^ 
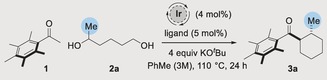

Entry	[Ir] (4 mol %)^[b]^	Ligand	Yield^[c]^	d.r.^[d]^	e.r.^[e]^
1	[IrCp*Cl_2_]_2_	–	75	91:9	–
2	[Ir(cod)Cl]_2_	**4**	76	95:5	68:32
3	[Ir(cod)Cl]_2_	**5**	78	93:7	64:36
4	[Ir(cod)Cl]_2_	**6**	79	96:4	69:31
5	[Ir(cod)Cl]_2_	**7**	74	91:9	55:45
6	[Ir(cod)Cl]_2_	**8**	76	91:9	73:27
7	[Ir(cod)Cl]_2_	**9**	75	91:9	86:14
8	[Ir(cod)Cl]_2_	**10**	77	91:9	87:13
9	[Ir(cod)Cl]_2_	**11**	81	92:8	88:12
10^[f]^	[Ir(cod)Cl]_2_	**11**	80	93:7	89:11
11^[f]^	Ir(cod)acac	**11**	85	92:8	90:10
12^[f,g]^	Ir(cod)acac	**11**	88(87)	91:9	92:8

[a] Reaction conditions: **1** (1 equiv), diol (2 equiv), [Ir] (4 mol %), ligand (5 mol %), KO^*t*^Bu (4 equiv), PhMe (3 m), 110 °C, 24 h. [b] loading refers to mol % Ir. [c] Determined by reverse phase HPLC analysis vs. durene as an internal standard; values in parentheses indicate the yield of isolated product. [d] Determined by reverse phase HPLC analysis. [e] Determined by normal phase HPLC analysis using a chiral stationary phase. [f] With ^*t*^BuOH as solvent. [g] With 2 mol % Ir(cod)acac and at [**1**]=1 M. cod=1,5‐cyclooctadiene; acac=acetylacetonate; DTBM=3,5‐di‐*tert*‐butyl‐4‐methoxyphenyl. 
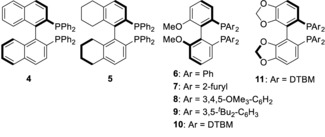

We found that changing the biaryl backbone of the ligand from MeO‐BIPHEP to SEGPHOS provided a small additional increase in enantioselectivity to 88:12 e.r. (Table 1, Entry 9). Conducting the reaction in *tert*‐butanol led to a further incremental improvement to 80 % yield and 89:11 e.r. (Table 1, Entry 10). Under these conditions we then screened a series of Ir, Rh, and Ru precatalysts (see Supporting Information for full details) and found that the best result was obtained with Ir(cod)(acac), which afforded **3 a** in 85 % yield and 90:10 e.r. (Table 1, Entry 11). Finally, we found that with a reduced Ir loading (2 mol %) and increased dilution (0.1 m) we were able to isolate **3 a** in 87 % yield and 92:8 e.r. (Table 1, Entry 12).

With optimal conditions in hand, we set out to evaluate the generality of the process. Substitution on the diol backbone was well tolerated with a diol bearing a geminal dimethyl group at the δ‐position cyclizing to afford **3 b** in 67 % yield, 90:10 d.r. and 94:6 e.r (Table 2, Entry 2). With substitution at the γ‐position we isolated cyclohexanes **3 c**–**3 e** in high yields and with excellent levels of diastereo‐ and enantioselectivity (Table 2, Entries 3–5). A diol bearing a *n*‐butyl group reacted to afford **3 f** in 87 % yield, 89:11 d.r. and 91:9 e.r. (Table 2, Entry 6). Interestingly, introduction of an isobutyl group resulted in poor conversion to cyclohexane **3 g** which was isolated in 24 % yield albeit still with good enantioselectivity (Table 2, Entry 7).^10^ Aromatic and heteroaromatic groups were well tolerated and cyclohexanes **3 h** and **3 i** were isolated in good yields with high levels of enantioselectivity (Table 2, Entries 8, 9). Diols bearing ether and thioether groups also cyclized smoothly to afford products **3 j** and **3 k** in excellent yields and high levels of stereoselectivity (Table 2, Entries 10,11). Even an acetal was tolerated in the chemistry providing **3 l** in 80 % yield and 86:14 e.r. with no evidence of any competing side‐reactions (Table 2, Entry 12). We also investigated an enantiopure diol derived from β‐thujone which we had previously found to undergo annulation with very poor diastereoselectivity (51:7:42 d.r.).^6^ We hoped that our optimized conditions might be able to augment this lack of substrate control and were pleased to find that **3 m** was isolated as a 90:10 mixture of diastereoisomers.^11^ Finally, we investigated formation of a cyclopentane from **1** and pentane‐1,4‐diol (Table 2, Entry 14). In this case, **3 n** was isolated in a reduced yield of 43 % albeit still with high levels of diastereo‐ and enantioselectivity.

**Table 2:** Scope of catalytic asymmetric hydrogen borrowing reaction.^[a]^ 
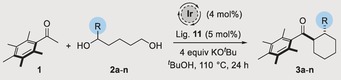

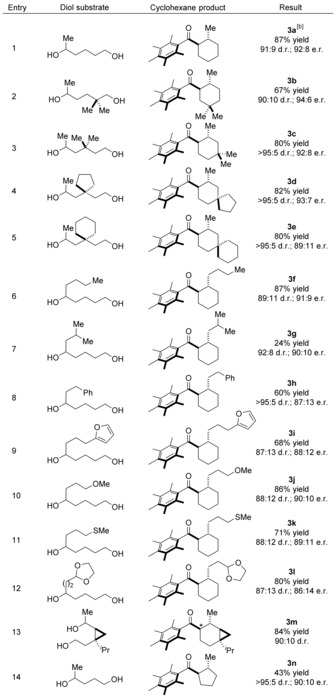

[a] Reaction conditions: **1** (1 equiv), diol (2 equiv), Ir(cod)acac (4 mol %), (*R*)‐DTBM‐SEGPHOS (5 mol %), KO^*t*^Bu (4 equiv), ^*t*^BuOH (3 m), 110 °C, 24 h. Major diastereoisomer depicted. Yields refer to isolated material after column chromatography. [b] Conditions from Table 1, Entry 12.

A further benefit of the Ph* group is its highly crystalline nature. All of the products **3 a**–**3 n** described above are crystalline solids and this provides an opportunity to enhance the enantiomeric purity by stereoselective crystallization. As a representative example, we carried out the reaction of pentamethylacetophenone with hexane‐1,5‐diol (**2 a**) on gram scale, obtaining **3 a** in 92 % yield with 93:7 d.r. and 92:8 e.r. (Scheme 2 A). After a single recrystallization (81 % recovery) we were able to significantly enhance this stereochemical purity to >95:5 d.r. and 98:2 e.r.

**Scheme 2:**
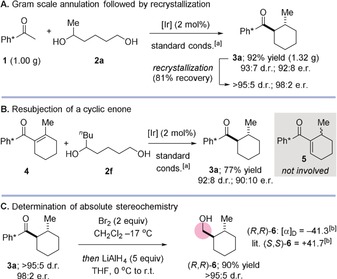
Large scale asymmetric annulation and experiments to determine competency of enone intermediate and absolute stereochemistry. [a] **1** (1 equiv), diol (2 equiv), Ir(cod)acac (2 mol %), (*R*)‐DTBM‐SEGPHOS (5 mol %), KO^*t*^Bu (4 equiv), ^*t*^BuOH (1 m), 110 °C, 24 h. [b] 23 °C, MeOH, *c*=1.00.

To probe the mechanism of the asymmetric hydrogen borrowing annulation, we independently synthesized the proposed key intermediate, cyclic enone **4** and subjected it to the optimized conditions with a *n*‐butyl substituted diol (Scheme 2 B). After this reaction we isolated **3 a** in 77 % yield and 90:10 e.r. The major enantiomer was the same as that obtained in the full hydrogen borrowing sequence and the yield, diastereo‐ and enantioselectivity were also very similar (c.f., Table 2, Entry 1). Based upon this result, we arrived at the following conclusions: (i) it is likely that cyclic enone **4** is an intermediate in the asymmetric hydrogen borrowing reaction; (ii) the absence of any crossover products implies that formation of **4** is an irreversible process; (iii) the similar enantioselectivities observed in the resubjection experiment and annulation process implies that the initial C−C bond formation between **1** and **2 a** occurs with complete regioselectivity at the primary end of the diol (i.e., reduction of isomeric enones such as **5** do not account for formation of the minor enantiomer). We have previously shown that Ph* containing products such as racemic **3 a**–**3 n** can be readily cleaved to the corresponding acid bromide in an *ipso*‐substitution reaction with Br_2_ and that the resulting acid bromides can be employed in situ to afford esters, amides, alcohols, carboxylic acids, and aldehydes without erosion of stereochemical purity.^5^, ^6^ This procedure gave us a convenient opportunity to determine the absolute stereochemistry of the cyclohexane products. To this end, ketone **3 a** was treated with Br_2_ to generate the corresponding acid bromide. Following addition of LiAlH_4_, alcohol **6** was isolated in 90 % yield with no stereochemical erosion (Scheme 2 C). Correlation of the specific rotation value of **6** with that previously reported in the literature allowed us to determine that the absolute configuration of **6** (and by extension **3 a**) is (*R*,*R*).^12^ The remaining examples in Table 2 are assigned by analogy.

To gain insight into the mechanism of the stereochemical determining step, density functional theory (DFT) modelling studies were conducted, employing a computationally tractable [Ir] complex ligated by (*R*)‐BINAP (Table 1, Entry 2). Following an extensive search for possible binding modes of an enone to a model Ir^I^ complex (for full details, see Supporting Information) the most stable was found to have both the carbonyl and alkene bound to the Ir center. The most stable [IrH(*R*‐BINAP)**4**] complex was then located (see Figures 1 and S5 and Table S1 in the Supporting Information).^13^ *Si*‐coordination of **4** (*Si*‐INT0) is computed to be favoured by 4.8 kcal mol^−1^ over its *Re* counterpart (*Re*‐INT0). 1,4 hydride insertion then proceeds from the *Si*‐face with a free energy barrier 0.8 kcal mol^−1^ lower than that for *Re*‐insertion and accounts for the experimentally observed e.r. (68:32=0.6 kcal mol^−1^ at 383 K, Tables 1 and S1). This preference results from the steric clash between Ph* and (P)Ph observed in the *Re*‐TS (Figure 1). Structures were optimized and thermodynamic/ solvent effects calculated at the PBE0‐D3BJ/def2‐SVP,def2‐TZVP(Ir) level of theory with the solvent accounted for using the SMD model. Single‐point energetics were evaluated on these stationary points at the PBE0‐D3BJ/def2‐TZVPP level of theory.^14^

**Figure 1:**
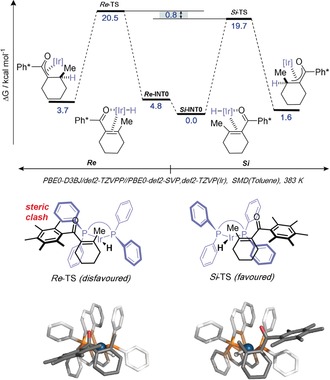
Computational studies to rationalise absolute stereochemical outcome.

In conclusion, we have developed a highly enantioselective synthesis of multisubstituted cyclohexanes via hydrogen borrowing catalysis. This process is mediated by two commercially available reagents: Ir(cod)(acac) and DTBM‐SEGPHOS and provides enantioenriched cyclohexanes with control over both diastereo‐ and enantioselectivity. The origins of stereoselectivity in this system have been probed by both experimental studies and DFT calculations. This approach constitutes the first general catalytic asymmetric strategy within the rapidly developing field of enolate hydrogen borrowing catalysis.

## Conflict of interest

The authors declare no conflict of interest.

## Supporting information

As a service to our authors and readers, this journal provides supporting information supplied by the authors. Such materials are peer reviewed and may be re‐organized for online delivery, but are not copy‐edited or typeset. Technical support issues arising from supporting information (other than missing files) should be addressed to the authors.

Supplementary
