# Supplementary material for: Catalytic Asymmetric Synthesis of Cyclohexanes by Hydrogen Borrowing Annulations
Source: Angew Chem Int Ed Engl. 2019 Aug 7;58(36):12558–62. doi: 10.1002/anie.201907514 (PMC6771629; doi:10.1002/anie.201907514)
Supplement: Supplementary file 1 — Supplementary [file ANIE-58-12558-s001.pdf]

Angewandte Chemie
Eine Zeitschrift der Gesellschaft Deutscher Chemiker

Supporting Information

Catalytic Asymmetric Synthesis of Cyclohexanes by Hydrogen Borrowing Annulations

Roly J. Armstrong, Wasim M. Akhtar, Tom A. Young, Fernanda Duarte, and Timothy J. Donohoe**

anie_201907514_sm_miscellaneous_information.pdf

Contents

1.	**General Information**	**S2**
2.	**General Procedure**	**S4**
3.	**Optimization**	**S5**
3.1.	*Index of Ligands*	**S5**
3.2.	*Extended Optimization Table*	**S6**
4.	**Experimental Procedures**	**S10**
4.1.	*Synthesis of Aryl Ketones*	**S10**
4.2.	*Synthesis of Diols*	**S12**
4.3.	*Asymmetric Hydrogen Borrowing Catalysis*	**S27**
4.4.	*Experiments from Scheme 2 and Additional Studies*	**S47**
5.	**Computational Modelling**	**S51**
5.1.	*Computational Methods*	**S51**
5.2	*Binding mode and conformational analysis*	**S51**
5.3.	*Origin of selectivity*	**S56**
5.4	*Methodology Validation*	**S57**
5.5	*Cartesian Coordinates and Energies*	**S59**
6.	**References**	**S95**
7.	**NMR spectra**	**S96**

1. General Information

Reactions were carried out in flame-dried glassware under an atmosphere of nitrogen unless stated otherwise. Room temperature (RT) refers to 20-25 °C. Temperatures of 0 °C were obtained using an ice/water bath. Temperatures of –17 °C were obtained using a salt/ice bath. Temperatures of –78 °C were obtained using a dry ice/acetone bath. Reflux conditions were obtained using an oil bath equipped with a contact thermometer.

Diethyl ether, CH_2Cl_2 and tetrahydrofuran were purified by filtration through activated alumina columns employing the method of Grubbs *et al*.[1] All other solvents and reagents were used as supplied without prior purification. All other reagents were used directly as supplied by major chemical suppliers, or following purification procedures described by Perrin and Armarego.[2]

Thin layer chromatography was performed on Merck Kieselgel 60 F_{254} 0.25 mm pre-coated aluminium plates. Product spots were visualized under UV light (λ = 254 nm) and/or by staining with potassium permanganate solution. Flash chromatography was performed using VWR silica gel 60 (40-63 µm particle size) using head pressure by means of a nitrogen line.

NMR spectroscopy was carried out using Bruker 400 MHz, 500 MHz, 600 MHz, 700 MHz or Cryo 500 MHz spectrometers in the deuterated solvent stated, using the residual non-deuterated solvent signal as an internal reference. Chemical shifts are quoted in ppm with signal splittings recorded as singlet (s), doublet (d), triplet (t), quartet (q), quintet (qn), sextet (sext), septet (sept), octet (oct), nonet (non) and multiplet (m). The abbreviation br denotes broad. Coupling constants, *J*, are measured to the nearest 0.1 Hz and are presented as observed.

Infrared spectra were recorded neat on a Bruker Tensor 27 spectrometer equipped with an attenuated total reflectance attachment with internal calibration. Absorption maxima (λ_{max}) are quoted in wavenumbers (cm^{-1}). The abbreviation br denotes broad.

Electrospray ionisation (ESI) HRMS were recorded on a Thermo Exactive orbitrap spectrometer equipped with a Waters Equity LC system, with a flow rate of 0.2 mL/min using water:methanol:formic acid (10:89.9:0.1) as eluent. The system uses a heated electrospray

ionisation (HESI-II) probe for ESI^+ and has a resolution of 50,000 FWHM under conditions for maximum sensitivity, with an accuracy of better than 5 ppm for 24 h following external calibration on the day of analysis. The mass reported is that containing the most abundant isotopes, with each value rounded to 4 decimal places and within 5 ppm of the calculated mass. Electron impact ionisation (EI) HRMS were performed on an Agilent 7200 quadrupole time of flight (Q-ToF) instrument equipped with a direct insertion probe supplied by Scientific Instrument Manufacturer (SIM) GmbH. Instrument control and data processing were performed using Agilent MassHunter software. The mass reported is that containing the most abundant isotopes, with each value to 4 decimal places and within 5 ppm of the calculated mass.

Optical rotations were recorded on a Schmidt Haensch Unipol L2000 polarimeter in a cell with a path length of 1 dm (using the sodium D line, 589 nm). Concentrations are reported in g/100 mL. Temperatures are reported in °C.

Chiral normal phase HPLC was performed on an Agilent 1260 Series HPLC unit equipped with UV-vis diode-array detector, fitted with the appropriate Daicel Chiralpak column (dimensions: 0.46 cm ø x 25 cm) along with the corresponding guard column (0.4 cm ø x 1 cm). Wavelengths (λ) are reported in nm, retention times (t_R) are reported in minutes and solvent flow rates are reported in mL min^{-1}.

Reverse phase HPLC was performed on a Dionex UltiMate 3000 system equipped with UV-vis variable wavelength detector, fitted with an Agilent InfinityLab Poroshell 120 EC-C18 column (0.46 cm ø x 150 mm, 4 µm pore size).

2. General Procedure

General Procedure A: Hydrogen Borrowing Catalysed Synthesis of Cyclohexanes

Under an air atmosphere, a 2–5 mL Biotage® microwave vial equipped with a stirrer bar, was sequentially charged with the appropriate diol (2.0 equiv.), pentamethylacetophenone **1** (1 equiv.), (*R*)-DTBM SEGPHOS (5 mol%), Ir(cod)acac (2 *or* 4 mol%), *tert*-butanol (1.0 *or* 0.33 mL/mmol pentamethylacetophenone) and KO^{t}Bu (4 equiv.). The reaction vessel was sealed with a microwave vial cap (containing a Reseal™ septum) and the vial was heated to 110 ^{o}C in a preheated oil bath for 24 h. The mixture was cooled to RT and filtered through a SiO_2 plug (eluting with ~50 mL Et_2O). For ease of purification from residual (*R*)-DTBM-SEGPHOS, the crude ethereal solution was treated with *tert*-butyl hydroperoxide (5-6 M in decane, 50 µL, ~0.28 mmol), swirled and allowed to stand at RT for 10 min. The resulting solution was then concentrated and purified by column chromatography (see experimental methods section for details). Racemic cyclohexane products were obtained using our previously reported procedure.[3]

3. Optimization

3.1 Index of Ligands

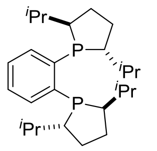
(*R*,*R*)-^{i}Pr-DUPHOS

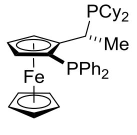
(*R*,*S*$_{P}$)-JOSIPHOS

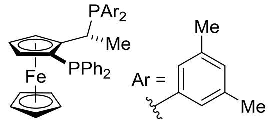
sl-j005

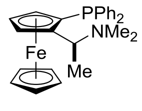
(*R*$_{P}$,*S*)-Me-PPFA

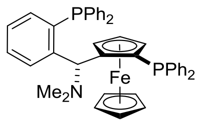
(*R*$_{P}$,*R*)-TANIAPHOS

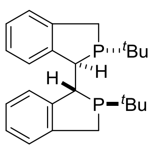
DUANPHOS

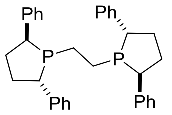
(*S*,*S*)-Ph-BPE

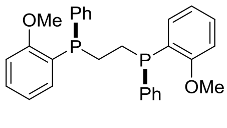
(*R*,*R*)-DIPAMP

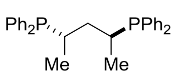
(*S*,*S*)-BDPP

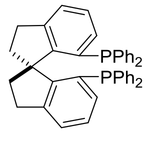
(*S*)-SDP

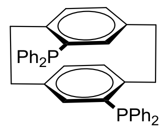
(*S*)-PHANEPHOS

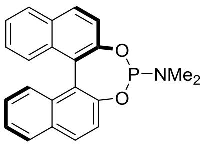
(*R*)-Monophos

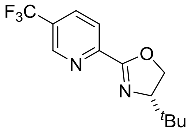
(*S*)-5-CF_3-Pyrox-^{t}Bu

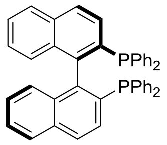
(*R*)-BINAP

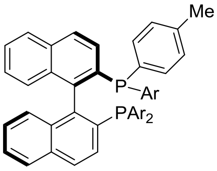
(*R*)-Tol-BINAP

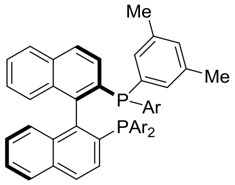
(*R*)-Xyl-BINAP

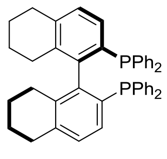
(*R*)-H_8-BINAP

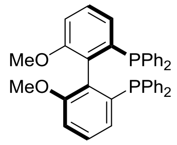
(*R*)-MeO-BIPHEP

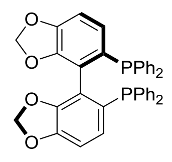
(*R*)-SEGPHOS

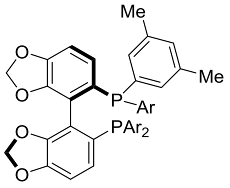
(*R*)-DM-SEGPHOS

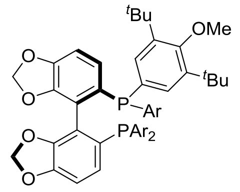
(*R*)-DTBM-SEGPHOS

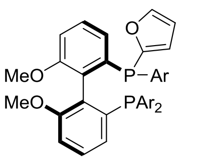
(*R*)-furyl-MeOBIPHEP

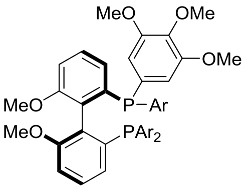
(*R*)-3,4,5-OMe-MeOBIPHEP

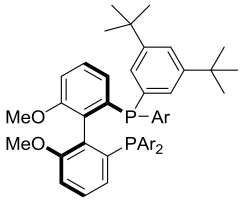
(*R*)-3,5-^{t}Bu-MeOBIPHEP

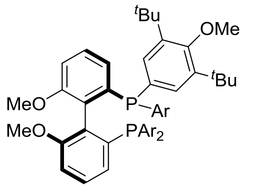
(*R*)-DTBM-MeOBIPHEP

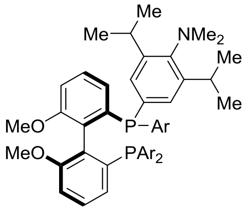
(*R*)-amino-MeOBIPHEP

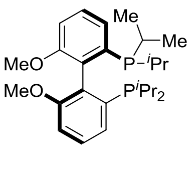
(*R*)-^{i}Pr-MeOBIPHEP

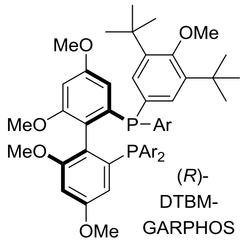
(*R*)-DTBM-GARPHOS

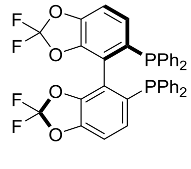
(*R*)-DIFLUOROPHOS

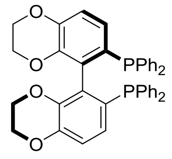
(*R*)-SYNPHOS

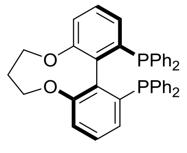
(*R*)-C3-TUNEPHOS

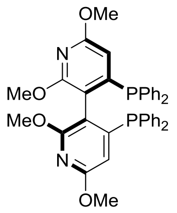
(*R*)-P-PHOS

3.2 Extended Optimization Table

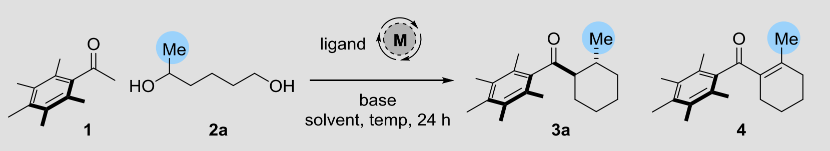

Under an air atmosphere, a 2–5 mL Biotage® microwave vial equipped with a stirrer bar, was sequentially charged with diol **2a** (71 mg, 0.60 mmol), pentamethylacetophenone **1** (57 mg, 0.30 mmol), *ligand*, *metal precatalyst*, *solvent* and *base*. The reaction vessel was sealed with a microwave vial cap (containing a Reseal™ septum) and the vial was heated to *the indicated temperature* in a preheated oil bath for 24 h. The mixture was cooled to RT and filtered through a SiO_2 plug (eluting with Et_2O) and the filtrate was concentrated under reduced pressure. The residue was dissolved in MeCN (50 mL) and durene (40 mg, 0.30 mmol) was added as an internal standard. The yields of **1**, **3a** and **4** were determined by calibrated reverse phase HPLC analysis (Agilent InfinityLab Poroshell 120 EC-C18 column with guard, 75% MeCN, 25% H_2O, 1.0 mL/min, 25 °C, λ = 254 nm, 5 µL injection). An analytical sample of **3a** (~1 mg) was obtained by small scale preparative TLC (95:5 pentane/Et_2O) and the enantioselectivity was determined by normal phase HPLC analysis (Chiralpak OD column with guard, 0.3 % IPA, 99.7 % hexane, 0.7 mL/min, 25 °C, λ = 254 nm, 10 µL injection).

Entry	[M] (mol%)[a]	Ligand (mol%)	Base (equiv.)	Solvent	T/°C	Yield 3a[b]	d.r.[c]	e.r.[d]
1	$[IrCp^*Cl_2]_2$ (4)	–	KO^{t}Bu (4)	PhMe (3M)	110	75	91:9	–
2	$[Ir(cod)Cl]_2$ (4)	(*R*)-BINAP (5)	KOH (4)	PhMe (3M)	110	27 (45% **4**)	92:8	56:44
3	$[Ir(cod)Cl]_2$ (4)	(*R*)-Tol-BINAP (5)	NaO^{t}Bu (4)	PhMe (3M)	110	48 (38% **4**)	90:10	59:41
4	$[Ir(cod)Cl]_2$ (4)	(*R*)-Tol-BINAP (5)	KO^{t}Bu (4)	PhMe (3M)	110	76	96:4	67:33
5	$[Ir(cod)Cl]_2$ (4)	(*R*)-Tol-BINAP (5)	KO^{t}Bu (4)	PhMe (3M)	90	58	96:4	65:35
6	$[Ir(cod)Cl]_2$ (4)	(*S*)-Tol-BINAP (5)	KO^{t}Bu (4)	PhMe (3M)	110	77	95:5	34:66
7	$[Ir(cod)Cl]_2$ (4)	(*R*,*R*)-^{i}Pr-DUPHOS (5)	KO^{t}Bu (4)	PhMe (3M)	110	60 (14% **4**)	94:6	34:66
8	$[Ir(cod)Cl]_2$ (4)	(*R*,*S$_P$*)-JOSIPHOS (5)	KO^{t}Bu (4)	PhMe (3M)	110	73	95:5	49:51
9	$[Ir(cod)Cl]_2$ (4)	sl-j005 (5)	KO^{t}Bu (4)	PhMe (3M)	110	61	91:9	42:58

10	$[Ir(cod)Cl]_2$ (4)	(R_P,*S*)-Me-PPFA (5)	KO^{t}Bu (4)	PhMe (3M)	110	36 (25% **4**)	93:7	51:49
11	$[Ir(cod)Cl]_2$ (4)	(R_P,*R*)-TANIAPHOS (5)	KO^{t}Bu (4)	PhMe (3M)	110	36 (28% **4**)	91:9	45:55
12	$[Ir(cod)Cl]_2$ (4)	DUANPHOS (5)	KO^{t}Bu (4)	PhMe (3M)	110	78	96:4	52:48
13	$[Ir(cod)Cl]_2$ (4)	(*S*,*S*)-Ph-BPE (5)	KO^{t}Bu (4)	PhMe (3M)	110	72	95:5	57:43
14	$[Ir(cod)Cl]_2$ (4)	(*R*,*R*)-DIPAMP (5)	KO^{t}Bu (4)	PhMe (3M)	110	77	97:3	53:47
15	$[Ir(cod)Cl]_2$ (4)	(*S*,*S*)-BDPP (5)	KO^{t}Bu (4)	PhMe (3M)	110	66	97:3	51:49
16	$[Ir(cod)Cl]_2$ (4)	(*S*)-SDP (5)	KO^{t}Bu (4)	PhMe (3M)	110	42 (28% **4**)	92:8	53:47
17	$[Ir(cod)Cl]_2$ (4)	(*S*)-PHANEPHOS (5)	KO^{t}Bu (4)	PhMe (3M)	110	76	93:7	38:62
18	$[Ir(cod)Cl]_2$ (4)	(*R*)-Monophos (5)	KO^{t}Bu (4)	PhMe (3M)	110	43 (23% **4**)	93:7	47:53
19	$[Ir(cod)Cl]_2$ (4)	(*S*)-5-CF_3-Pyrox-^{t}Bu (5)	KO^{t}Bu (4)	PhMe (3M)	110	41 (23% **4**)	93:7	48:52
20	$[Ir(cod)Cl]_2$ (4)	(R)-BINAP (5)	KO^{t}Bu (4)	PhMe (3M)	110	76	95:5	68:32
21	$[Ir(cod)Cl]_2$ (4)	(R)-Xyl-BINAP (5)	KO^{t}Bu (4)	PhMe (3M)	110	82	95:5	70:30
22	$[Ir(cod)Cl]_2$ (4)	(*R*)-H_8-BINAP (5)	KO^{t}Bu (4)	PhMe (3M)	110	78	93:7	64:36
23	$[Ir(cod)Cl]_2$ (4)	(R)-MeO-BIPHEP (5)	KO^{t}Bu (4)	PhMe (3M)	110	79	96:4	69:31
24	$[Ir(cod)Cl]_2$ (4)	(*R*)-SEGPHOS (5)	KO^{t}Bu (4)	PhMe (3M)	110	78	96:4	67:33
25	$[Ir(cod)Cl]_2$ (4)	(*R*)-DM-SEGPHOS (5)	KO^{t}Bu (4)	PhMe (3M)	110	76	95:5	82:18
26	$[Ir(cod)Cl]_2$ (4)	(*R*)-DTBM-SEGPHOS (5)	KO^{t}Bu (4)	PhMe (3M)	110	81	92:8	88:12
27	$[Ir(cod)Cl]_2$ (4)	(*R*)-furyl-MeOBIPHEP (5)	KO^{t}Bu (4)	PhMe (3M)	110	74	91:9	54:45
28	$[Ir(cod)Cl]_2$ (4)	(*R*)-3,4,5-OMe-MeOBIPHEP (5)	KO^{t}Bu (4)	PhMe (3M)	110	76	91:9	73:27
29	$[Ir(cod)Cl]_2$ (4)	(*R*)-3,5-^{t}Bu-MeOBIPHEP (5)	KO^{t}Bu (4)	PhMe (3M)	110	75	91:9	86:14
30	$[Ir(cod)Cl]_2$ (4)	(*R*)-DTBM-MeOBIPHEP (5)	KO^{t}Bu (4)	PhMe (3M)	110	77	91:9	87:13
31	$[Ir(cod)Cl]_2$ (2)	(*R*)-DTBM-SEGPHOS (2.5)	KO^{t}Bu (4)	PhMe (3M)	110	72	90:10	86:14
32	$[Ir(cod)Cl]_2$ (4)	(*R*)-DTBM-SEGPHOS (5)	KO^{t}Bu (4)	none	110	64	95:5	85:15
33	$[Ir(cod)Cl]_2$ (4)	(*R*)-DTBM-SEGPHOS (5)	KO^{t}Bu (4)	^{t}BuOH (3M)	110	80	93:7	89:11
34	$[Ir(coe)Cl]_2$ (4)	(*R*)-DTBM-SEGPHOS (5)	KO^{t}Bu (4)	^{t}BuOH (3M)	110	91	92:8	86:14
35	$[IrCp^*Cl_2]_2$ (4)	(*R*)-DTBM-SEGPHOS (5)	KO^{t}Bu (4)	^{t}BuOH (3M)	110	81	95:5	63:37
36	$[Ir(cod)OMe]_2$ (4)	(*R*)-DTBM-SEGPHOS (5)	KO^{t}Bu (4)	^{t}BuOH (3M)	110	85	92:8	90:10
37	Ir(cod)acac (4)	(*R*)-DTBM-SEGPHOS (5)	KO^{t}Bu (4)	^{t}BuOH (3M)	110	85	92:8	90:10
38	$[Rh(cod)Cl]_2$ (4)	(*R*)-DTBM-SEGPHOS (5)	KO^{t}Bu (4)	^{t}BuOH (3M)	110	36 (30% **4**)	92:8	51:49
39	$[Rh(cod)OH]_2$ (4)	(*R*)-DTBM-SEGPHOS (5)	KO^{t}Bu (4)	^{t}BuOH (3M)	110	36 (31% **4**)	92:8	51:49
40	$RhCl_3$ (4)	(*R*)-DTBM-SEGPHOS (5)	KO^{t}Bu (4)	^{t}BuOH (3M)	110	27 (39% **4**)	93:7	50:50
41	$Rh_2(OAc)_4$ (4)	(*R*)-DTBM-SEGPHOS (5)	KO^{t}Bu (4)	^{t}BuOH (3M)	110	29 (48% **4**)	92:8	50:50
42	$[Ru(cod)Cl_2]_n$ (4)	(*R*)-DTBM-SEGPHOS (5)	KO^{t}Bu (4)	^{t}BuOH (3M)	110	56 (16% **4**)	92:8	70:30
43	$[RuCl_2(p\text{-cy})]_2$ (4)	(*R*)-DTBM-SEGPHOS (5)	KO^{t}Bu (4)	^{t}BuOH (3M)	110	60 (11% **4**)	92:8	41:59

44	$[RuCp^*Cl_2]_n$ (4)	(*R*)-DTBM-SEGPHOS (5)	KO^{t}Bu (4)	^{t}BuOH (3M)	110	20 (24% **4**)	92:8	50:50
45	$Ru(acac)_3$ (4)	(*R*)-DTBM-SEGPHOS (5)	KO^{t}Bu (4)	^{t}BuOH (3M)	110	71	92:8	78:22
46	Ir(cod)acac (2)	(*R*)-DTBM-SEGPHOS (5)	KO^{t}Bu (4)	^{t}BuOH (3M)	110	84	92:8	91:9
47	Ir(cod)acac (2)	(*R*)-DTBM-SEGPHOS (5)	KO^{t}Bu (4)	benzene (3M)	110	84	92:8	89:11
48	Ir(cod)acac (2)	(*R*)-DTBM-SEGPHOS (5)	KO^{t}Bu (4)	*m*-xylene (3M)	110	84	92:8	89:11
49	Ir(cod)acac (2)	(*R*)-DTBM-SEGPHOS (5)	KO^{t}Bu (4)	heptane (3M)	110	85	92:8	90:10
50	Ir(cod)acac (2)	(*R*)-DTBM-SEGPHOS (5)	KO^{t}Bu (4)	1,4-dioxane (3M)	110	81	91:9	89:11
51	Ir(cod)acac (2)	(*R*)-DTBM-SEGPHOS (5)	KO^{t}Bu (4)	DMF (3M)	110	<5 (11% **4**)	n.d.	n.d.
52	Ir(cod)acac (2)	(*R*)-DTBM-SEGPHOS (5)	KO^{t}Bu (4)	NMP (3M)	110	41 (31% **4**)	90:10	68:32
**53**	**Ir(cod)acac (2)**	**(*R*)-DTBM-SEGPHOS (5)**	**KO^{t}Bu (4)**	**^{t}BuOH (1M)**	**110**	**88 (87%)**	**91:9**	**92:8**
54	Ir(cod)acac (2)	(*R*)-DTBM-SEGPHOS (5)	KO^{t}Bu (4)	^{t}BuOH (0.5M)	110	88	91:9	91:9
55	Ir(cod)acac (2)	(*R*)-DTBM-SEGPHOS (5)	KO^{t}Bu (4)	^{t}BuOH (0.3M)	110	81 (7% **4**)	92:8	89:11
56	Ir(cod)acac (2)	(*R*)-DTBM-SEGPHOS (5)	KOH (4)	^{t}BuOH (1M)	110	94	90:10	89:11
57	Ir(cod)acac (2)	(*R*)-DTBM-SEGPHOS (5)	K_3PO_4 (4)	^{t}BuOH (1M)	110	<5 (82% **1a**)	n.d.	n.d.
58	Ir(cod)acac (2)	(*R*)-DTBM-SEGPHOS (5)	KHMDS (4)	^{t}BuOH (1M)	110	84	86:14	91:9
59	Ir(cod)acac (2)	(*R*)-DTBM-SEGPHOS (5)	NaO^{t}Bu (4)	^{t}BuOH (1M)	110	20 (43% **1a**)	90:10	51:49
60	Ir(cod)acac (2)	(*R*)-DTBM-SEGPHOS (5)	NaOH (4)	^{t}BuOH (1M)	110	26 (47% **4**)	90:10	65:35
61	Ir(cod)acac (2)	(*R*)-DTBM-SEGPHOS (5)	LiO^{t}Bu (4)	^{t}BuOH (1M)	110	<5 (88% **1a**)	n.d.	n.d.
62	Ir(cod)acac (2)	(*R*)-DTBM-SEGPHOS (5)	$CsOH.H_2O$ (4)	^{t}BuOH (1M)	110	76	91:9	83:17
63	Ir(cod)acac (2)	(*R*)-DTBM-SEGPHOS (5)	KO^{t}Bu (1)	^{t}BuOH (1M)	110	24 (53% **4**)	91:9	62:38
64	Ir(cod)acac (2)	(*R*)-DTBM-SEGPHOS (5)	KO^{t}Bu (8)	^{t}BuOH (1M)	110	78	84:16	91:9
65	Ir(cod)acac (2)	(*R*)-DTBM-SEGPHOS (5)	KO^{t}Bu (4)	^{t}BuOH (1M)	90	82	96:4	91:9
66	Ir(cod)acac (2)	(*R*)-DTBM-SEGPHOS (5)	KO^{t}Bu (4)	^{t}BuOH (1M)	70	26 (15% **4**)	93:7	88:12
67	Ir(cod)acac (2)	(*R*)-SEGPHOS (5)	KO^{t}Bu (4)	^{t}BuOH (1M)	110	49	93:7	62:38
68	Ir(cod)acac (2)	(*R*)-BINAP (5)	KO^{t}Bu (4)	^{t}BuOH (1M)	110	64	93:7	58:42
69	Ir(cod)acac (2)	(R)-MeO-BIPHEP (5)	KO^{t}Bu (4)	^{t}BuOH (1M)	110	64	92:8	64:36
70	Ir(cod)acac (2)	(*R*)-furyl-MeOBIPHEP (5)	KO^{t}Bu (4)	^{t}BuOH (1M)	110	68	92:8	62:38
71	Ir(cod)acac (2)	(*R*)-3,4,5-OMe-MeOBIPHEP (5)	KO^{t}Bu (4)	^{t}BuOH (1M)	110	86	92:8	72:28
72	Ir(cod)acac (2)	(*R*)-3,5-^{t}Bu-MeOBIPHEP (5)	KO^{t}Bu (4)	^{t}BuOH (1M)	110	82	92:8	88:12
73	Ir(cod)acac (2)	(*R*)-amino-MeOBIPHEP (5)	KO^{t}Bu (4)	^{t}BuOH (1M)	110	82	92:8	75:25
74	Ir(cod)acac (2)	(*R*)-^{i}Pr-MeOBIPHEP (5)	KO^{t}Bu (4)	^{t}BuOH (1M)	110	18 (59% **4**)	91:9	50:50
75	Ir(cod)acac (2)	(*R*)-DTBM-GARPHOS (5)	KO^{t}Bu (4)	^{t}BuOH (1M)	110	86	91:9	90:10
76	Ir(cod)acac (2)	(*R*)-DIFLUOROPHOS (5)	KO^{t}Bu (4)	^{t}BuOH (1M)	110	48 (21% **4**)	90:10	50:50
77	Ir(cod)acac (2)	(*R*)-SYNPHOS (5)	KO^{t}Bu (4)	^{t}BuOH (1M)	110	47 (12% **4**)	92:8	64:36

78	Ir(cod)acac (2)	(*R*)-C3-TUNEPHOS (5)	KO^{t}Bu (4)	^{t}BuOH (1M)	110	62	91:9	69:31
79	Ir(cod)acac (2)	(*R*)-P-PHOS (5)	KO^{t}Bu (4)	^{t}BuOH (1M)	110	46 (30% **4**)	92:8	51:49
80	Ir(cod)acac (2)	(*R*)-DTBM-SEGPHOS (5)	KO^{t}Bu (4)	anhyd. ^{t}BuOH (1M)	110	89	93:7	91:9
81[e]	Ir(cod)acac (2)	(*R*)-DTBM-SEGPHOS (5)	KO^{t}Bu (4)	anhyd. ^{t}BuOH (1M)	110	36 (53% **4**)	91:9	83:17
82[f]	Ir(cod)acac (2)	(*R*)-DTBM-SEGPHOS (5)	KO^{t}Bu (4)	^{t}BuOH (1M)	110	30	96:4	87:13
83	none	(*R*)-DTBM-SEGPHOS (5)	KO^{t}Bu (4)	^{t}BuOH (1M)	110	3 (8% **4**)	93:7	51:49

[a] Loading refers to mol% metal after dissociation of precursors. [b] Determined by reverse phase HPLC analysis *vs* durene as an internal standard [c] Determined by reverse phase HPLC analysis. [d] Determined by normal phase HPLC analysis using a chiral stationary phase. [e] Reaction carried out with 200 mg powdered 3Å molecular sieves. [f] Reaction carried out with an argon balloon.

4. *Experimental Procedures*

4.1 *Synthesis of Aryl Ketones*

1-(2,3,4,5,6-Pentamethylphenyl)ethan-1-one, 1

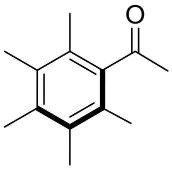

A solution of pentamethylbenzene (10.0 g, 67.4 mmol) and acetyl chloride (5.27 mL, 74.2 mmol) in CH_2Cl_2 (400 mL) was cooled to 0 ^{o}C and aluminium chloride (11.2 g, 84.3 mmol) was added in several portions over 10 mins. The resulting mixture was warmed to RT and stirred for 4 h and then poured onto crushed ice (c.a. 500 g). After the ice had melted, the layers were separated and the aqueous layer was extracted twice with CH_2Cl_2. The combined organic extracts were washed with brine, dried over anhydrous $MgSO_4$, filtered and concentrated *in vacuo*. Purification *via* column chromatography eluting with 95:5 pentane/Et_2O afforded the title compound **1** as a white solid (12.0 g, 93%). The spectral data matched that previously reported in the literature.[4]

m.p. = 83–85 ^{o}C [lit. 83–85 ^{o}C].[5]

IR (film) ν_{max}/cm^{-1} 2925, 1697, 1416, 1350, 1305, 1168, 998, 93.

^{1}H NMR ($CDCl_3$, 400 MHz) δ = 2.46 (3H, s), 2.24 (3H, s), 2.19 (6H, s), 2.14 (6H, s).

^{13}C NMR ($CDCl_3$, 101 MHz) δ = 210.1, 140.9, 135.4, 133.1, 127.0, 33.2, 17.1, 16.7, 16.0.

LRMS (ESI^{+}) Found [M+Na]$^{+}$ = 213; $C_{13}H_{18}NaO$ requires 213.

(2-Methylcyclohex-1-en-1-yl)(2,3,4,5,6-pentamethylphenyl)methanone, 4

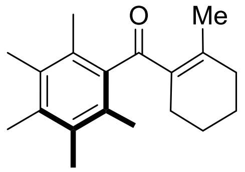

Crude reaction mixtures from several small scale screening experiments containing **4** were combined and purified *via* column chromatography eluting with 95:5 pentane/Et_2O followed by recrystallization from boiling MeOH to afford the title compound **4** as white solid.

m.p. = 138–140 °C.

IR (film) ν_{max}/cm^{-1} 2921, 1303, 1276, 1169, 1068, 905, 797, 708.

^{1}H NMR ($CDCl_3$, 400 MHz) δ = 2.22 (3H, s), 2.21 – 2.18 (2H, m), 2.17 (6H, s), 2.15 – 2.12 (2H, m), 2.05 (6H, s), 1.87 (3H, br s), 1.62 – 1.54 (4H, m).

^{13}C NMR ($CDCl_3$, 101 MHz) δ = 204.0, 148.9, 141.2, 134.8, 133.2, 132.8, 128.1, 35.0, 26.5, 22.7, 22.4, 22.1, 17.0, 16.7, 16.0.

HRMS (ESI$^+$) Found $[M+H]^+$ = 271.2056; $C_{19}H_{27}O$ requires 271.2056, Δ –0.17 ppm.

4.2 *Synthesis of Diols*

Diols **2a** and **2n** were purchased from Sigma Aldrich and used as received. The majority of other diols were synthesised by partial reduction of lactones with DIBAL-H followed by addition of a Grignard reagent. Experimental details and full characterization are provided below.

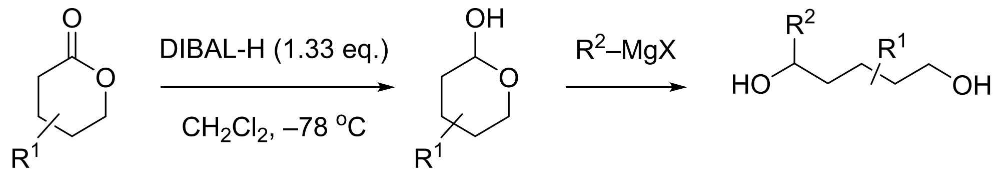

5,5-Dimethyltetrahydro-2*H*-pyran-2-one, S1

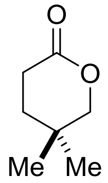

Based on a literature procedure,[6] a suspension of $NaBH_4$ (3.84 g, 103 mmol) in THF (50 mL) was stirred at 0 °C and a solution of 2,2-dimethylglutaric anhydride (4.82 g, 33.9 mmol) in THF (50 mL) was added dropwise. The resulting solution was warmed to RT and stirred for 48 h and then cooled to 0 °C and 3M HCl (100 mL) was added carefully. The resulting solution was concentrated under reduced pressure to remove the majority of the THF and the remaining aqueous phase was extracted three times with EtOAc. The combined organic extracts were washed with brine and then dried over anhydrous $MgSO_4$, filtered and concentrated under reduced pressure. The residue was dissolved in toluene (50 mL) and *p*-toluenesulfonic acid monohydrate (645 mg, 3.39 mmol) was added. The resulting solution was stirred at RT for 16 h and then diluted with EtOAc and sat. aq. $NaHCO_3$ was added. The organic layer was separated and the aqueous layer was extracted twice with ethyl acetate. The combined organic extracts were washed with brine and then dried over anhydrous $MgSO_4$, filtered and concentrated under reduced pressure. Purification *via* column

chromatography eluting with 70:30 pentane/Et_2O afforded the title compound **S1** as a colourless oil (1.18 g, 27%). The spectral data matched that previously reported in the literature.[7]

IR (film) ν_{max}/cm^{-1} 2961, 2876, 1733, 1460, 1380, 1345, 1291, 1225, 1197, 1166, 1056.

^{1}H NMR ($CDCl_3$, 400 MHz) δ = 3.96 (2H, s), 2.54 (2H, t, J=7.3 Hz), 1.68 (2H, t, J=7.3 Hz), 1.04 (6H, s).

^{13}C NMR ($CDCl_3$, 101 MHz) δ = 171.4, 78.8, 32.9, 29.4, 27.3, 24.8.

LRMS (EI^+) Found $[M]^+$ = 128; $C_7H_{12}O_2$ requires 128.

2,2-Dimethylhexane-1,5-diol, 2b

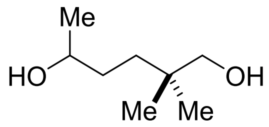

A solution of lactone **S1** (1.17 g, 9.12 mmol) in CH_2Cl_2 (18 mL) was stirred at –78 °C and diisobutylaluminum hydride (1 M in hexanes, 12.1 mL, 12.1 mmol) was added dropwise over 5 min. The resulting solution was stirred at –78 °C for 1.5 h and then sat. aq. Rochelle salt was added dropwise and the mixture was allowed to warm to RT with vigorous stirring. The organic layer was separated and the aqueous layer was extracted twice with CH_2Cl_2. The combined organic extracts were dried over anhydrous $MgSO_4$, filtered and concentrated *in vacuo*.

The resulting crude lactol was dissolved in THF (7 mL) and cooled to 0 °C and a solution of methylmagnesium bromide (3 M in Et_2O, 15.2 ml, 45.6 mmol) was added dropwise. The reaction mixture was then warmed to RT and stirred for 2 h and then cooled to 0 °C, diluted with Et_2O and quenched by dropwise addition of sat. aq. ammonium chloride. The organic layer was separated and the aqueous layer was extracted twice with EtOAc. The combined organic extracts were dried over anhydrous $MgSO_4$, filtered and concentrated *in vacuo*. Purification *via* column chromatography eluting with 97:3 CH_2Cl_2/MeOH afforded the title compound **2b** as a colourless oil (1.07 g, 80%).

IR (film) ν_{max}/cm^{-1} 3322 (br), 2960, 2869, 1472, 1365, 1124, 1039, 981, 941, 846.

^{1}H NMR ($CDCl_3$, 400 MHz) δ = 3.81 – 3.71 (1H, m), 3.34 (1H, dd, J=10.9, 4.1 Hz), 3.27 (1H, dd, J=11.0,

4.7 Hz), 2.73 (1H, br s), 2.54 (1H, br s), 1.49 – 1.33 (3H, m), 1.29 – 1.12 (4H, m), 0.88 (3H, s), 0.85 (3H, s).

^{13}C NMR ($CDCl_3$, 101 MHz) δ = 70.9, 68.7, 34.8, 33.9, 33.2, 24.5, 23.9, 23.6.

HRMS (ESI^+) Found $[M+Na]^+$ = 169.1200; $C_8H_{18}O_2Na$ requires 169.1199, Δ 0.74 ppm.

4,4-Dimethyltetrahydro-2*H*-pyran-2-one, S2

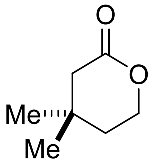

A stirred, neat mixture of 2,2-dimethylpentane-1,5-diol[3] (2.00 g, 15.1 mmol) and copper chromite (94 mg, 0.30 mmol) was heated to 200 °C for 16 h and then cooled to RT. The crude reaction mixture was directly purified *via* column chromatography eluting with 60:40 pentane/Et_2O to afford the title compound **S2** as a colourless gum (1.76 g, 91%). The spectral data matched that previously reported in the literature.[8]

IR (film) ν_{max}/cm^{-1} 2957, 1730, 1467, 1403, 1370, 1254, 1224, 1174, 1078, 1044.

^{1}H NMR ($CDCl_3$, 400 MHz) δ = 4.34 (2H, t, J=6.1 Hz), 2.30 (2H, s), 1.67 (2H, t, J=6.1 Hz), 1.06 (6H, s).

^{13}C NMR ($CDCl_3$, 101 MHz) δ = 171.6, 66.6, 44.2, 35.9, 29.8, 28.8.

LRMS (ESI^+) Found $[M+Na]^+$ = 151; $C_7H_{12}O_2Na$ requires 151.

3,3-Dimethylhexane-1,5-diol, 2c

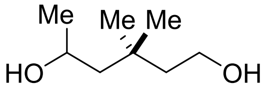

A solution of lactone **S2** (1.30 g, 10.1 mmol) in CH_2Cl_2 (20 mL) was stirred at –78 °C and diisobutylaluminum hydride (1 M in hexanes, 13.4 mL, 13.4 mmol) was added dropwise over 5 min. The resulting solution was stirred at –78 °C for 1.5 h and then sat. aq. Rochelle salt was added dropwise and the mixture was allowed to warm to RT with vigorous stirring. The organic layer was

separated and the aqueous layer was extracted twice with CH_2Cl_2. The combined organic extracts were dried over anhydrous $MgSO_4$, filtered and concentrated *in vacuo*.

The resulting crude lactol was dissolved in THF (7 mL) and cooled to 0 °C and a solution of methylmagnesium bromide (3 M in Et_2O, 11.8 ml, 35.4 mmol) was added dropwise. The reaction mixture was then warmed to RT and stirred for 2 h and then cooled to 0 °C, diluted with Et_2O and quenched by dropwise addition of sat. aq. ammonium chloride. The organic layer was separated and the aqueous layer was extracted twice with EtOAc. The combined organic extracts were dried over anhydrous $MgSO_4$, filtered and concentrated *in vacuo*. Purification *via* column chromatography eluting with 97:3 CH_2Cl_2/MeOH afforded the title compound **2c** as a colourless gum (680 mg, 46%). The spectral data matched that previously reported in the literature.[9]

IR (film) ν_{max}/cm^{-1} 3301 (br), 2959, 1470, 1367, 1124, 1050, 1023.

^{1}H NMR ($CDCl_3$, 400 MHz) δ = 3.97 (1H, dqd, *J*=8.6, 6.2, 2.2 Hz), 3.77 – 3.64 (2H, m), 3.08 (2H, br s), 1.82 (1H, ddd, *J*=14.4, 8.1, 6.3 Hz), 1.64 (1H, dd, *J*=14.8, 8.7 Hz), 1.42 (1H, dt, *J*=14.3, 5.6 Hz), 1.25 (1H, dd, *J*=14.8, 2.0 Hz), 1.17 (3H, d, *J*=6.2 Hz), 0.95 (3H, s), 0.93 (3H, s).

^{13}C NMR ($CDCl_3$, 101 MHz) δ = 65.1, 59.4, 50.1, 43.1, 32.1, 28.9, 28.8, 26.1.

LRMS (ESI^+) Found $[M+Na]^+$ = 169; $C_8H_{18}O_2Na$ requires 169.

8-Oxaspiro[4.5]decan-7-one, S3

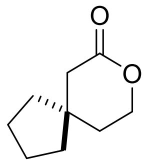

A stirred, neat mixture of 2,2'-(cyclopentane-1,1-diyl)bis(ethan-1-ol)[3] (2.39 g, 15.1 mmol) and copper chromite (94 mg, 0.30 mmol) was heated to 200 °C for 16 h and then cooled to RT. The crude reaction mixture was directly purified *via* column chromatography eluting with 50:50 pentane/Et_2O to afford the title compound **S3** as a colourless oil (2.24 g, 96%). The spectral data matched that previously reported in the literature.[10]

IR (film) ν_{max}/cm^{-1} 2951, 1735, 1402, 1256, 1223, 1166, 1074.

^{1}H NMR ($CDCl_3$, 400 MHz) δ = 4.36 (2H, t, *J*=6.1 Hz), 2.41 (2H, s), 1.76 (2H, t, *J*=6.0 Hz), 1.72 – 1.64 (4H, m), 1.59 – 1.44 (4H, m).

^{13}C NMR ($CDCl_3$, 101 MHz) δ = 171.5, 67.4, 42.5, 40.8, 38.7, 34.4, 23.8.

LRMS (EI^+) Found $[M]^+$ = 154; $C_9H_{14}O_2$ requires 154.

1-(1-(2-Hydroxyethyl)cyclopentyl)propan-2-ol, 2d

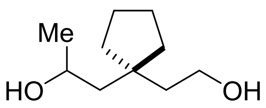

A solution of lactone **S3** (2.13 g, 13.8 mmol) in CH_2Cl_2 (28 mL) was stirred at –78 °C and diisobutylaluminum hydride (1 M in hexanes, 18.4 mL, 18.4 mmol) was added dropwise over 5 min. The resulting solution was stirred at –78 °C for 1.5 h and then sat. aq. Rochelle salt was added dropwise and the mixture was allowed to warm to RT with vigorous stirring. The organic layer was separated and the aqueous layer was extracted twice with CH_2Cl_2. The combined organic extracts were dried over anhydrous $MgSO_4$, filtered and concentrated *in vacuo*.

The resulting crude lactol was dissolved in THF (10 mL) and cooled to 0 °C and a solution of methylmagnesium bromide (3 M in Et_2O, 23.0 ml, 69.0 mmol) was added dropwise. The reaction mixture was then warmed to RT and then heated to 60 °C and stirred at this temperature for 20 h and then cooled to 0 °C, diluted with Et_2O and quenched by dropwise addition of sat. aq. ammonium chloride. The organic layer was separated and the aqueous layer was extracted twice with EtOAc. The combined organic extracts were dried over anhydrous $MgSO_4$, filtered and concentrated *in vacuo*. Purification *via* column chromatography eluting with 95:5 CH_2Cl_2/MeOH afforded the title compound **2d** as a white solid (2.20 g, 93%).

m.p. = 42–45 °C

IR (film) ν_{max}/cm^{-1} 3301 (br), 2948, 2869, 1455, 1372, 1126, 1055, 1015, 933.

^{1}H NMR ($CDCl_3$, 400 MHz) δ = 3.95 (1H, dqd, *J*=8.4, 6.2, 2.1 Hz), 3.75 – 3.67 (2H, m), 3.51 (2H, br s),

1.92 (1H, ddd, J=14.2, 7.6, 6.3 Hz), 1.74 (1H, dd, J=14.9, 8.5 Hz), 1.63 – 1.30 (10H, m), 1.17 (3H, d, J=6.3 Hz).

^{13}C NMR ($CDCl_3$, 101 MHz) δ = 66.0, 60.1, 47.4, 44.3, 40.2, 38.8, 38.7, 26.1, 24.1, 24.0.

HRMS (ESI$^+$) Found $[M+Na]^+$ = 195.1356; $C_{10}H_{20}O_2Na$ requires 195.1356, Δ 0.28 ppm.

3-Oxaspiro[5.5]undecan-2-one, S4

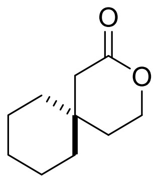

A stirred, neat mixture of 2,2'-(cyclohexane-1,1-diyl)bis(ethan-1-ol)[3] (1.34 g, 7.77 mmol) and copper chromite (48 mg, 0.15 mmol) was heated to 200 °C for 16 h and then cooled to RT. The crude reaction mixture was directly purified *via* column chromatography eluting with 50:50 pentane/Et_2O to afford the title compound **S4** as a colourless oil (1.27 g, 97%).

IR (film) ν_{max}/cm^{-1} 2924, 2853, 1732, 1453, 1258, 1228, 1199, 1103, 1082, 1064.

^{1}H NMR ($CDCl_3$, 400 MHz) δ = 4.30 (2H, t, J=6.0 Hz), 2.35 (2H, s), 1.72 (2H, t, J=6.0 Hz), 1.56 – 1.31 (10H, m).

^{13}C NMR ($CDCl_3$, 101 MHz) δ = 172.1, 65.9, 42.5, 37.6, 33.6, 32.7, 25.7, 21.6.

HRMS (ESI$^+$) Found $[M+H]^+$ = 169.1224; $C_{10}H_{17}O_2$ requires 169.1223, Δ 0.62 ppm.

1-(1-(2-Hydroxyethyl)cyclohexyl)propan-2-ol, 2e

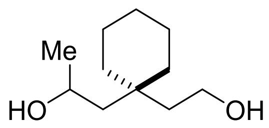

A solution of lactone **S4** (1.22 g, 7.25 mmol) in CH_2Cl_2 (14 mL) was stirred at −78 °C and diisobutylaluminum hydride (1 M in hexanes, 9.7 mL, 9.7 mmol) was added dropwise over 5 min. The resulting solution was stirred at −78 °C for 1.5 h and then sat. aq. Rochelle salt was added dropwise and the mixture was allowed to warm to RT with vigorous stirring. The organic layer was

separated and the aqueous layer was extracted twice with CH_2Cl_2. The combined organic extracts were dried over anhydrous $MgSO_4$, filtered and concentrated *in vacuo*.

The resulting crude lactol was dissolved in THF (5 mL) and cooled to 0 °C and a solution of methylmagnesium bromide (3 M in Et_2O, 12.0 ml, 36.0 mmol) was added dropwise. The reaction mixture was then warmed to RT and then heated to 60 °C and stirred at this temperature for 20 h and then cooled to 0 °C, diluted with Et_2O and quenched by dropwise addition of sat. aq. ammonium chloride. The organic layer was separated and the aqueous layer was extracted twice with EtOAc. The combined organic extracts were dried over anhydrous $MgSO_4$, filtered and concentrated *in vacuo*. Purification *via* column chromatography eluting with 97:3 CH_2Cl_2/MeOH afforded the title compound **2e** as a white solid (1.32 g, 98%).

m.p. = 50–52 °C.

IR (film) ν_{max}/cm^{-1} 3304 (br), 2923, 2851, 1455, 1125, 1055, 1018.

^{1}H NMR ($CDCl_3$, 400 MHz) δ = 3.99 (1H, dqd, *J*=8.4, 6.2, 2.1 Hz), 3.78 – 3.65 (2H, m), 3.05 (2H, br s), 1.83 (1H, ddd, *J*=14.5, 8.6, 5.7 Hz), 1.65 – 1.56 (2H, m), 1.47 – 1.28 (11H, m), 1.19 (3H, d, *J*=6.2 Hz).

^{13}C NMR ($CDCl_3$, 101 MHz) δ = 64.4, 58.7, 46.4, 39.0, 36.8, 34.2, 26.4, 26.4, 21.6, 21.5.

HRMS (ESI$^+$) Found $[M+Na]^+$ = 209.1512; $C_{11}H_{22}O_2Na$ requires 209.1512, Δ 0.06 ppm.

Nonane-1,5-diol, 2f

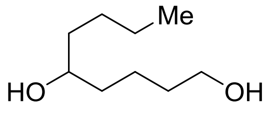

A stirred suspension of $LiAlH_4$ pellets (900 mg, 23.7 mmol) in THF (100 mL) was cooled to 0 °C and δ-nonalactone (3.36 mL, 19.2 mmol) was added dropwise. The resulting suspension was warmed to RT and stirred for 1 h. The resulting solution was cooled to 0 °C and diluted with Et_20 (~100 mL) and then quenched by sequential dropwise addition of H_2O (0.90 mL), aq. NaOH (15 % w/v, 0.90 mL) and H_2O (2.7 mL). $MgSO_4$ was added and the resulting suspension was warmed to RT and stirred vigorously for 30 min and then filtered and concentrated *in vacuo*. Purification *via* column

chromatography eluting with 95:5 CH_2Cl_2/MeOH afforded the title compound **2f** as a colourless oil (2.96 g, 96%). The spectral data matched that previously reported in the literature.[11]

IR (film) ν_{max}/cm^{-1} 3315 (br), 2930, 2860, 1458, 1377, 1340, 1129, 1102, 1055, 1018, 924.

^{1}H NMR ($CDCl_3$, 400 MHz) δ = 3.67 – 3.52 (3H, m), 2.56 (1H, br s), 2.26 (1H, br s), 1.64 – 1.19 (12H, m), 0.88 (3H, t, *J*=7.0 Hz).

^{13}C NMR ($CDCl_3$, 101 MHz) δ = 71.7, 62.5, 37.2, 36.9, 32.5, 27.9, 22.8, 21.8, 14.1.

LRMS (ESI$^+$) Found $[M+Na]^+$ = 183; $C_9H_{20}O_2Na$ requires 183.

7-Methyloctane-1,5-diol, 2g

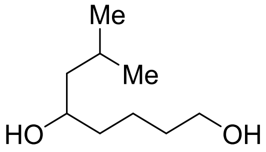

A stirred solution of isobutylmagnesium chloride (2 M in Et_2O, 16.5 mL, 33.0 mmol) was cooled to 0 °C and a solution of tetrahydro-2*H*-pyran-2-ol (1.12 g, 11.0 mmol) in THF (8 mL) was added dropwise. The reaction mixture was then warmed to RT and stirred for 2 h and then cooled to 0 °C, diluted with Et_2O and quenched by dropwise addition of sat. aq. ammonium chloride. The organic layer was separated and the aqueous layer was extracted twice with EtOAc. The combined organic extracts were dried over anhydrous $MgSO_4$, filtered and concentrated *in vacuo*. Purification *via* column chromatography eluting with 97:3 CH_2Cl_2/MeOH afforded the title compound **2g** as a colourless oil (1.47 g, 83%). The spectral data matched that previously reported in the literature.[12]

IR (film) ν_{max}/cm^{-1} 3315 (br), 2931, 2868, 1467, 1367, 1141, 1103, 1055, 1023, 917.

^{1}H NMR ($CDCl_3$, 400 MHz) δ = 3.71 – 3.59 (3H, m), 2.21 (1H, br s), 1.94 (1H, br s), 1.83 – 1.68 (1H, m), 1.66 – 1.31 (7H, m), 1.21 (1H, ddd, *J*=13.8, 8.8, 4.2 Hz), 0.90 (3H, d, J=6.6 Hz), 0.89 (3H, d, J=6.6 Hz).

^{13}C NMR ($CDCl_3$, 101 MHz) δ = 69.7, 62.6, 46.8, 37.5, 32.5, 24.6, 23.5, 22.1, 21.8.

LRMS (ESI$^+$) Found $[M+Na]^+$ = 183; $C_9H_{20}O_2Na$ requires 183.

7-Phenylheptane-1,5-diol, 2h

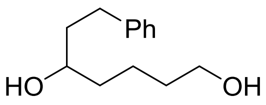

A stirred solution of phenethylmagnesium chloride (1 M in THF, 33.0 mL, 33.0 mmol) was cooled to 0 °C and a solution of tetrahydro-2*H*-pyran-2-ol (1.12 g, 11.0 mmol) in THF (8 mL) was added dropwise. The reaction mixture was then warmed to RT and stirred for 2 h and then cooled to 0 °C, diluted with Et_2O and quenched by dropwise addition of sat. aq. ammonium chloride. The organic layer was separated and the aqueous layer was extracted twice with EtOAc. The combined organic extracts were dried over anhydrous $MgSO_4$, filtered and concentrated *in vacuo*. Purification *via* column chromatography eluting with 97:3 CH_2Cl_2/MeOH afforded the title compound **2h** as a colourless oil (1.64 g, 72%). The spectral data matched that previously reported in the literature.[13]

IR (film) ν_{max}/cm^{-1} 3318 (br), 2934, 2862, 1495, 1454, 1057, 1031, 747, 699.

^{1}H NMR ($CDCl_3$, 400 MHz) δ = 7.23 – 7.17 (2H, m), 7.14 – 7.08 (3H, m), 3.59 – 3.51 (3H, m), 2.71 (1H, ddd, *J*=13.7, 9.2, 6.3 Hz), 2.58 (1H, ddd, *J*=13.7, 9.3, 7.0 Hz), 2.40 (1H, br s), 2.32 (1H, br s), 1.76 – 1.60 (2H, m), 1.57 – 1.28 (6H, m).

^{13}C NMR ($CDCl_3$, 101 MHz) δ = 142.2, 128.4, 128.4, 125.8, 71.1, 62.5, 39.2, 37.0, 32.4, 32.1, 21.8.

LRMS (ESI^+) Found $[M+Na]^+$ = 231; $C_{13}H_{20}O_2Na$ requires 231.

8-(Furan-2-yl)octane-1,5-diol, 2i

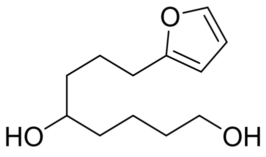

A suspension of magnesium turnings (656 mg, 27.0 mmol) in THF (15 mL) was stirred at RT and a single crystal of iodine was added followed by addition of 2-(3-bromopropyl)furan[14] (3.06 g, 16.2 mmol) at such a rate as to maintain gentle reflux. After the addition was complete the resulting suspension was heated to reflux for a further 10 min and then cooled to RT and decanted away from

the excess magnesium turnings by cannulation washing with THF (2 x 2 mL). The resulting Grignard solution was cooled to 0 °C and tetrahydro-2*H*-pyran-2-ol (551 mg, 5.40 mmol) was added dropwise. The reaction mixture was then warmed to RT and stirred for 2 h and then cooled to 0 °C, diluted with Et_2O and quenched by dropwise addition of sat. aq. ammonium chloride. The organic layer was separated and the aqueous layer was extracted twice with EtOAc. The combined organic extracts were dried over anhydrous $MgSO_4$, filtered and concentrated *in vacuo*. Purification *via* column chromatography eluting with 97:3 CH_2Cl_2/MeOH afforded the title compound **2i** as a colourless oil (996 mg, 87%).

IR (film) ν_{max}/cm^{-1} 3318 (br), 2936, 2864, 1596, 1508, 1458, 1434, 1338, 1147, 1056, 1005, 922, 729.

^{1}H NMR ($CDCl_3$, 400 MHz) δ = 7.28 (1H, dd, *J*=1.8, 0.7 Hz), 6.26 (1H, dd, *J*=3.1, 1.9 Hz), 5.98 (1H, dd, *J*=3.1, 0.8 Hz), 3.67 – 3.57 (3H, m), 2.63 (2H, t, *J*=7.5 Hz), 2.11 (2H, br s), 1.85 – 1.33 (10H, m).

^{13}C NMR ($CDCl_3$, 101 MHz) δ = 156.1, 140.8, 110.1, 104.8, 71.4, 62.6, 36.9, 36.9, 32.4, 27.9, 24.2, 21.8.

HRMS (ESI$^+$) Found $[M+Na]^+$ = 235.1304; $C_{12}H_{20}O_3Na$ requires 235.1305, Δ –0.27 ppm.

8-Methoxyoctane-1,5-diol, 2j

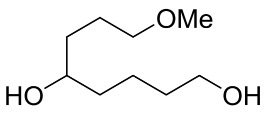

A suspension of magnesium turnings (1.33 g, 55.0 mmol) in THF (10 mL) was stirred at RT and a single crystal of iodine was added followed by addition of 1-bromo-3-methoxypropane (3.67 mL, 32.9 mmol) at such a rate as to maintain gentle reflux. After the addition was complete the resulting suspension was heated to reflux for a further 10 min and then cooled to RT, diluted with THF (10 mL) and decanted away from the excess magnesium turnings by cannulation washing with THF (2 x 5 mL). The resulting Grignard solution was cooled to 0 °C and tetrahydro-2*H*-pyran-2-ol (1.12 g, 11.0 mmol) was added dropwise. The reaction mixture was then warmed to RT and stirred for 2 h and then cooled to 0 °C, diluted with Et_2O and quenched by dropwise addition of sat. aq.

ammonium chloride. The organic layer was separated and the aqueous layer was extracted twice with EtOAc. The combined organic extracts were dried over anhydrous $MgSO_4$, filtered and concentrated *in vacuo*. Purification *via* column chromatography eluting with 97:3 CH_2Cl_2/MeOH afforded the title compound **2j** as a colourless oil (1.32 g, 68%). The spectral data matched that previously reported in the literature.[12]

IR (film) ν_{max}/cm^{-1} 3347 (br), 2931, 2863, 1452, 1387, 1112, 1058, 951, 915.

^{1}H NMR ($CDCl_3$, 400 MHz) δ = 3.65 – 3.52 (3H, m), 3.42 – 3.36 (2H, m), 3.32 (3H, s), 3.20 (1H, br s), 2.58 (1H, br s), 1.75 – 1.34 (10H, m).

^{13}C NMR ($CDCl_3$, 101 MHz) δ = 73.1, 71.3, 62.4, 58.6, 36.9, 34.8, 32.5, 26.2, 21.9.

LRMS (ESI^+) Found $[M+Na]^+$ = 199; $C_9H_{20}O_3Na$ requires 199.

8-(Methylthio)octane-1,5-diol, 2k

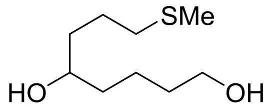

A suspension of magnesium turnings (1.33 g, 55.0 mmol) in THF (10 mL) was stirred at RT and a single crystal of iodine was added followed by addition of (3-bromopropyl)(methyl)sulfane[15] (5.58 g, 33.0 mmol) at such a rate as to maintain gentle reflux. After the addition was complete the resulting suspension was heated to reflux for a further 10 min and then cooled to RT, diluted with THF (10 mL) and decanted away from the excess magnesium turnings by cannulation washing with THF (2 x 5 mL). The resulting Grignard solution was cooled to 0 °C and tetrahydro-2*H*-pyran-2-ol (1.12 g, 11.0 mmol) was added dropwise. The reaction mixture was then warmed to RT and stirred for 2 h and then cooled to 0 °C, diluted with Et_2O and quenched by dropwise addition of sat. aq. ammonium chloride. The organic layer was separated and the aqueous layer was extracted twice with EtOAc. The combined organic extracts were dried over anhydrous $MgSO_4$, filtered and concentrated *in vacuo*. Purification *via* column chromatography eluting with 97:3 CH_2Cl_2/MeOH afforded the title compound **2j** as a colourless oil (2.09 g, 99%).

IR (film) ν_{max}/cm^{-1} 3324 (br), 2932, 2861, 1427, 1055.

^{1}H NMR ($CDCl_3$, 400 MHz) δ = 3.67 – 3.56 (3H, m), 2.50 (2H, t, *J*=7.2 Hz), 2.23 (2H, br s), 2.08 (3H, s), 1.80 – 1.36 (10H, m).

^{13}C NMR ($CDCl_3$, 101 MHz) δ = 71.3, 62.5, 37.0, 36.5, 34.3, 32.4, 25.2, 21.8, 15.5.

HRMS (ESI^+) Found $[M+Na]^+$ = 215.1078; $C_9H_{20}O_2NaS$ requires 215.1076, Δ 0.67 ppm.

7-(1,3-Dioxolan-2-yl)heptane-1,5-diol, 2l

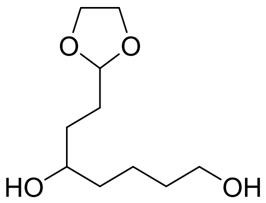

A suspension of magnesium turnings (968 mg, 39.8 mmol) in THF (20 mL) was stirred at RT and 2-(2-bromoethyl)-1,3-dioxolane (2.40 mL, 20.4 mmol) was added at such a rate as to maintain gentle reflux. After the addition was complete the resulting suspension was cooled to RT and decanted away from the excess magnesium turnings by cannulation washing with THF (2 x 5 mL). The resulting Grignard solution was cooled to 0 °C and tetrahydro-2*H*-pyran-2-ol (350 mg, 3.42 mmol) was added dropwise. The reaction mixture was then warmed to RT and stirred for 2 h and then cooled to 0 °C, diluted with Et_2O and quenched by dropwise addition of sat. aq. ammonium chloride. The organic layer was separated and the aqueous layer was extracted twice with EtOAc. The combined organic extracts were dried over anhydrous $MgSO_4$, filtered and concentrated *in vacuo*. Purification *via* column chromatography eluting with EtOAc afforded the title compound **2l** as a white solid (399 mg, 57%).

m.p. = 44–45 °C.

IR (film) ν_{max}/cm^{-1} 3372 (br), 2934, 2866, 1449, 1410, 1142, 1032, 946.

^{1}H NMR ($CDCl_3$, 400 MHz) δ = 4.87 (1H, t, *J*=4.5 Hz), 3.99 – 3.91 (2H, m), 3.88 – 3.80 (2H, m), 3.65 – 3.57 (3H, m), 2.76 (1H, br s), 2.25 (1H, br s), 1.87 – 1.71 (2H, m), 1.67 – 1.35 (8H, m).

^{13}C NMR ($CDCl_3$, 101 MHz) δ = 104.5, 71.3, 64.9, 64.9, 62.5, 36.9, 32.5, 31.4, 30.0, 21.8.

HRMS (ESI$^+$) Found $[M+Na]^+$ = 227.1255; $C_{10}H_{20}O_4Na$ requires 227.1254, Δ 0.61 ppm.

(1*S*,6*S*)-6-Isopropyl-2-methyl-3-oxabicyclo[4.1.0]heptan-4-one, S5

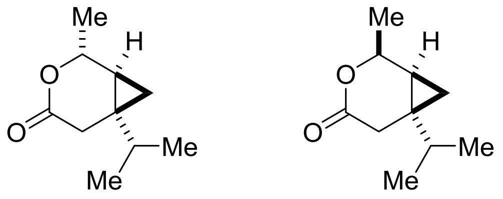

S5 (major) **S5 (minor)**

According to a modified literature procedure,[16] a stirred solution of α,β-thujone (technical grade, ~70% α-thujone, ~10% β-thujone, 5.00 mL, 24.3 mmol) in CH_2Cl_2 (5 mL) was stirred at RT in a 30 mL vial and 3-chloroperbenzoic acid (≤77%, 17.5 g, 71.0 mmol) was added in a single portion. The vial was capped and the resulting suspension was stirred at RT for 4 days. The reaction mixture was poured into a rapidly stirred mixture of CH_2Cl_2 and sat. aq. $Na_2S_2O_3$. The organic layer was separated and washed twice with sat. aq. $Na_2S_2O_3$ and once with sat. aq. K_2CO_3 and then dried over anhydrous $MgSO_4$, filtered and concentrated *in vacuo*. Purification *via* column chromatography eluting with 85:15 pentane/EtOAc afforded the title compound **S5** as an inseparable mixture of diastereoisomers as a colourless oil (2.87 g, 70%, 86:14 d.r.). The spectral data for both diastereoisomers matched that previously reported in the literature.[16]

IR (film) ν_{max}/cm^{-1} 2960, 1732, 1454, 1379, 1268, 1238, 1212, 1183, 1162, 1144, 1069, 1018, 967.

HRMS (ESI+) Found $[M+Na]^+$ = 191.1043; $C_{10}H_{16}O_2Na$ requires 191.1043, Δ 0.14 ppm.

$[\alpha]_D^{25}$ +1 (c = 1.00, $CHCl_3$).

<u>NMR data for **S5 (major)**:</u>

^{1}H NMR ($CDCl_3$, 400 MHz) δ = 4.39 (1H, qd, *J*=6.6, 3.7 Hz), 2.61 (1H, d, *J*=16.9 Hz), 2.37 (1H, d, *J*=16.8 Hz), 1.46 (3H, d, *J*=6.5 Hz), 1.11 (1H, sept, *J*=6.8 Hz), 0.97 – 0.89 (7H, m), 0.72 – 0.66 (1H, m), 0.60 (1H, t, *J*=5.1 Hz).

^{13}C NMR ($CDCl_3$, 101 MHz) δ = 172.0, 76.4, 35.0, 31.7, 24.4, 22.7, 22.2, 18.9, 18.5, 16.4.

<u>NMR data for **S5 (minor)**:</u>

^{1}**H NMR** ($CDCl_3$, 400 MHz) δ = 4.66 (1H, q, *J*=6.3 Hz), 2.67 (1H, d, *J*=16.5 Hz), 2.51 (1H, d, *J*=16.5 Hz), 1.37 (3H, d, *J*=6.2 Hz), 1.26 – 1.16 (1H, m), 1.02 (1H, dd, *J*=8.5, 4.7 Hz), 0.96 – 0.90 (6H, m), 0.53 (1H, t, *J*=5.3 Hz), 0.46–0.40 (1H, m).

^{13}**C NMR** ($CDCl_3$, 101 MHz) δ = 171.7, 72.9, 34.4, 30.5, 22.5, 22.3, 21.1, 18.9, 18.5, 8.8.

2-((1*S*,2*S*)-2-(1-hydroxyethyl)-1-isopropylcyclopropyl)ethan-1-ol, 2m

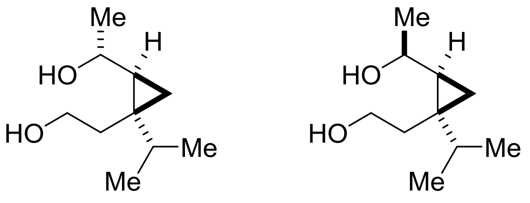

3m (major) **3m (minor)**

A stirred suspension of $LiAlH_4$ (1.69 g, 44.6 mmol) in THF (230 mL) was cooled to 0 °C and a solution of lactone **S5** (2.50 g, 14.9 mmol, 86:14 d.r.) in THF (20 mL) was added dropwise. The resulting suspension was warmed to RT and stirred for 1 h. The resulting solution was cooled to 0 °C and diluted with Et_2O (200 mL) and then quenched by sequential dropwise addition of H_2O (1.7 mL), aq. NaOH (15 % w/v, 1.7 mL) and H_2O (5.1 mL). $MgSO_4$ was added and the resulting suspension was warmed to RT and stirred vigorously for 30 min and then filtered and concentrated *in vacuo*. Purification *via* column chromatography eluting with 95:5 CH_2Cl_2/MeOH afforded the title compound **2m** as a mixture of diastereoisomers as a colourless oil (2.29 g, 89%, 85:15 d.r.). An analytical quantity of each diastereoisomer was obtained by column chromatography (EtOAc).

Data for **2m (major)**:

IR (film) ν_{max}/cm^{-1} 3278 (br), 2969, 1457, 1368, 1079, 972, 898.

HRMS (ESI+) Found $[M+Na]^+$ = 195.1357; $C_{10}H_{20}O_2Na$ requires 195.1356, Δ 0.51 ppm.

^{1}**H NMR** ($CDCl_3$, 400 MHz) δ = 4.75 (1H, br s), 3.97 (1H, br s), 3.88 (1H, td, *J*=11.1, 3.5 Hz), 3.66 (1H, ddd, *J*=10.7, 5.3, 3.1 Hz), 3.40 (1H, dq, *J*=10.1, 6.2 Hz), 1.98 (1H, dt, *J*=15.4, 3.4 Hz), 1.55 – 1.41 (2H, m), 1.27 (3H, d, *J*=6.1 Hz), 0.94 (3H, d, *J*=6.9 Hz), 0.87 (1H, td, *J*=9.3, 5.8 Hz), 0.82 (3H, d, *J*=6.9 Hz), 0.44 (1H, dd, *J*=9.2, 4.6 Hz), –0.18 (1H, t, *J*=5.2 Hz).

^{13}C NMR ($CDCl_3$, 101 MHz) δ = 68.2, 59.5, 32.8, 32.1, 31.1, 25.4, 22.6, 20.3, 18.4, 13.5.

$[\alpha]_D^{25}$ +16 (c = 1.00, $CHCl_3$).

<u>Data for **2m (minor)**:</u>

IR (film) ν_{max}/cm^{-1} 3319 (br), 2960, 2875, 1463, 1368, 1181, 1094, 1025, 975, 900.

HRMS (ESI+) Found $[M+Na]^+$ = 195.1357; $C_{10}H_{20}O_2Na$ requires 195.1356, Δ 0.74 ppm.

^{1}H NMR ($CDCl_3$, 400 MHz) δ = 3.72 (1H, td, *J*=9.8, 5.8 Hz), 3.68 – 3.60 (1H, m), 3.41 (1H, dt, *J*=12.5, 6.2 Hz), 1.86 (1H, ddd, *J*=14.6, 8.9, 5.9 Hz), 1.79 (2H, br s), 1.32 (1H, ddd, *J*=14.9, 9.2, 6.3 Hz), 1.27 (3H, d, *J*=6.2 Hz), 1.22 (1H, hept, *J*=6.8 Hz), 0.90 (3H, d, *J*=6.8 Hz), 0.79 (3H, d, *J*=6.9 Hz), 0.68 – 0.56 (2H, m), 0.36 (1H, t, *J*=4.9 Hz).

^{13}C NMR ($CDCl_3$, 101 MHz) δ = 69.0, 61.9, 34.5, 31.7, 29.9, 26.8, 23.6, 19.7, 18.7, 15.0.

$[\alpha]_D^{25}$ –20 (c = 1.00, $CHCl_3$).

4.3 Asymmetric Hydrogen Borrowing Catalysis

((1*R*,2*R*)-2-Methylcyclohexyl)(2,3,4,5,6-pentamethylphenyl)methanone, 3a

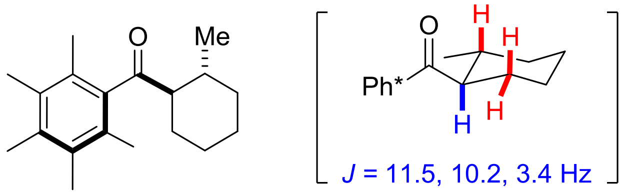

0.3 mmol scale reaction: Diol **2a** (71 mg, 0.60 mmol), pentamethylacetophenone **1** (57 mg, 0.30 mmol), (*R*)-DTBM-SEGPHOS (18 mg, 0.015 mmol), Ir(cod)acac (2.4 mg, 0.0060 mmol), *tert*-butanol (0.3 mL) and KO^{t}Bu (135 mg, 1.20 mmol) were subjected to **General Procedure A.** Purification *via* column chromatography eluting with 98:2 pentane/Et_2O afforded the title compound **3a** as a white solid (71 mg, 87%, 91:9 d.r., 92:8 e.r.). The relative stereochemistry was determined by *J*-coupling constant analysis.

m.p. = 137–139 °C

IR (film) ν_{max}/cm^{-1} 2925, 2877, 1682, 1445, 1381, 1314, 1261, 1162, 1142, 1109, 920.

^{1}H NMR ($CDCl_3$, 400 MHz, 299 K) δ = 2.45 (1H, ddd, *J*=11.5, 10.2, 3.4 Hz), 2.24 (3H, s), 2.18 (6H, s), 2.13 (6H, s), 2.02–1.90 (1H, m), 1.89–1.64 (4H, m), 1.33–1.13 (3H, m), 1.09 (3H, d, *J* = 6.9 Hz), 1.03 (1H, qd, *J* = 12.6, 3.6 Hz). The minor diastereoisomer displays a diagnostic peak at δ = 2.77 (1H, dt, *J* = 11.6, 3.1 Hz).

^{13}C NMR ($CDCl_3$, 101 MHz, 299 K) δ = 213.1, 139.7, 135.5, 133.0, 59.3, 34.6, 32.5, 29.5, 26.6, 25.9, 21.0, 17.9, 16.8, 16.1. [*N.B*. The signal for the *ortho*-quaternary aromatic carbon of the Ph* group was not observed, presumably due to restricted rotation about the Ar-CO axis. Upon warming to 318 K a broad signal for this carbon appeared at 128.8 ppm - see data below for full details].

^{1}H NMR ($CDCl_3$, 500 MHz, 318 K) δ = 2.46 (1H, ddd, *J*=11.6, 10.2, 3.4 Hz), 2.25 (3H, s), 2.19 (6H, s), 2.14 (6H, s), 2.02 – 1.91 (1H, m), 1.88 – 1.61 (4H, m), 1.33 – 1.13 (3H, m), 1.12 – 1.00 (4H, m).

^{13}C NMR ($CDCl_3$, 126 MHz, 298 K) δ = 212.8, 139.8, 135.4, 133.0, 128.8, 59.3, 34.7, 32.5, 29.5, 26.6, 25.9, 20.9, 17.8, 16.7, 16.0.

HRMS (ESI^+) Found $[M+H]^+$ = 273.2213; $C_{19}H_{29}O$ requires 273.2213, Δ 0.17 ppm.

$[\alpha]_D^{25}$ See large scale hydrogen borrowing reaction (*vide infra*)

Chiral HPLC (Chiralpak OD with guard, 0.3 % IPA, 99.7 % hexane, 0.7 mL/min, 25 °C, λ = 254 nm, 10 µL injection) [*N.B*. the peaks at 16.7 and 17.3 min correspond to the minor diasteroisomer].

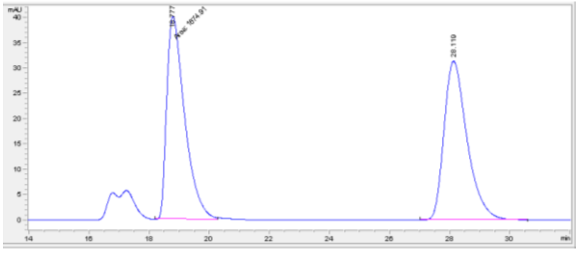

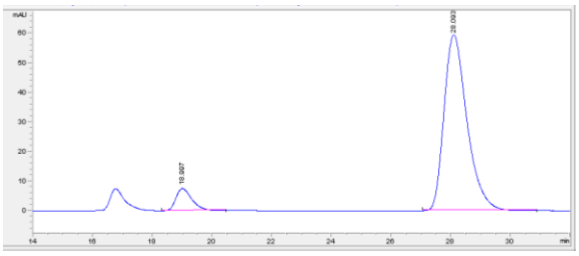

#	Time	Type	Area	Height	Width	Area%	Symmetry
1	18.777	MF	1674.9	40.1	0.6963	50.005	0.512
2	28.119	BB	1674.6	31.3	0.8095	49.995	0.66

#	Time	Type	Area	Height	Width	Area%	Symmetry
1	18.997	BB	280.2	7.5	0.5504	8.042	0.646
2	28.093	BB	3203.9	59.8	0.8176	91.958	0.689

(2,3,4,5,6-Pentamethylphenyl)((1*R*,2*R*)-2,5,5-trimethylcyclohexyl)methanone, 3b

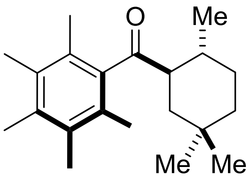

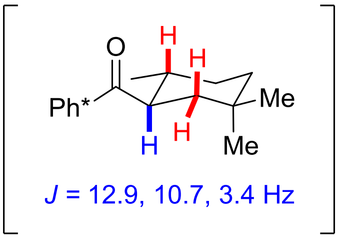

Diol **2b** (88 mg, 0.60 mmol), pentamethylacetophenone **1** (57 mg, 0.30 mmol), (*R*)-DTBM-SEGPHOS (18 mg, 0.015 mmol), Ir(cod)acac (4.8 mg, 0.012 mmol), *tert*-butanol (0.1 mL) and KO^tBu (135 mg, 1.20 mmol) were subjected to **General Procedure A.** Purification *via* column chromatography eluting with 97.5:2.5 pentane/Et_2O afforded the title compound **3b** as a white solid (60 mg, 67%, 90:10 d.r., 94:6 e.r.). The relative stereochemistry was determined by *J*-coupling constant analysis.

m.p. = 83–85 °C

IR (film) ν_{max}/cm^{-1} 2918, 1689, 1458, 1384, 1304, 1259, 1117, 1067, 1000, 912, 699.

^{1}H NMR ($CDCl_3$, 400 MHz) δ = 2.67 (1H, ddd, *J*=12.9, 10.7, 3.4 Hz), 2.24 (3H, s), 2.18 (6H, s), 2.12 (6H,

s), 1.92 – 1.77 (1H, m), 1.64 – 1.56 (1H, m), 1.45 (1H, dt, J=12.7, 2.9 Hz), 1.37 – 1.18 (3H, m), 1.14 – 1.06 (4H, m), 0.89 (3H, s), 0.80 (3H, s). The minor diastereoisomer displays a diagnostic peak at δ = 3.02 (1H, dt, J=13.3, 3.2 Hz).

^{13}C NMR ($CDCl_3$, 101 MHz) δ = 213.6, 139.7, 135.4, 133.0, 55.4, 41.7, 38.8, 32.9, 32.6, 30.9, 30.7, 23.8, 20.7, 18.0, 16.8, 16.1. [*N.B.* The signal for the *ortho*-quaternary aromatic carbon of the Ph* group was not observed, presumably due to restricted rotation about the Ar-CO axis].

$[\alpha]_D^{25}$ +6.9 (c = 1.00, $CHCl_3$).

HRMS (ESI^+) Found $[M+H]^+$ = 301.2527; $C_{21}H_{33}O$ requires 301.2526, Δ 0.51 ppm.

Chiral HPLC (Chiralpak OD with guard, 0.3 % IPA, 99.7 % hexane, 1.0 mL/min, 25 °C, λ = 254 nm, 10 µL injection) [*N.B.* the peaks at 7.6 and 8.0 min correspond to the minor diasteroisomer].

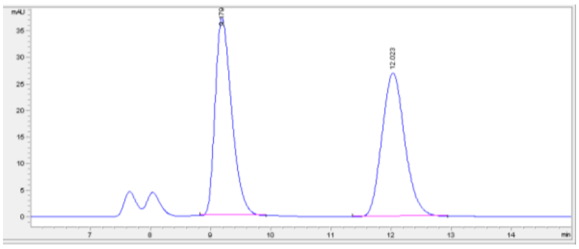

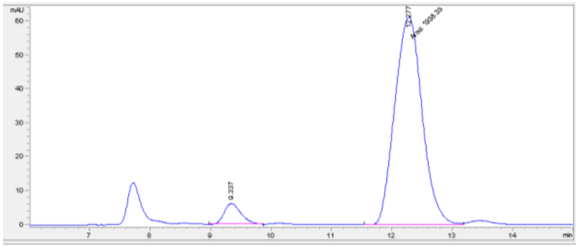

#	Time	Type	Area	Height	Width	Area%	Symmetry
1	9.179	BB	709.3	37.1	0.2949	49.863	0.653
2	12.023	BB	713.2	26.9	0.4129	50.137	0.888

#	Time	Type	Area	Height	Width	Area%	Symmetry
1	9.337	BB	119.1	6.1	0.2988	5.789	0.706
2	12.277	MF	1938.4	61.3	0.5268	94.211	0

(2,3,4,5,6-Pentamethylphenyl)((1*R*,2*R*)-2,4,4-trimethylcyclohexyl)methanone, 3c

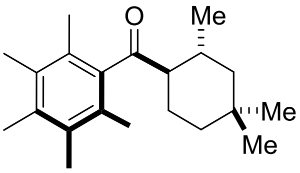

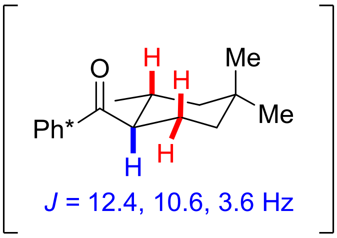

Diol **2c** (88 mg, 0.60 mmol), pentamethylacetophenone **1** (57 mg, 0.30 mmol), (*R*)-DTBM-SEGPHOS (18 mg, 0.015 mmol), Ir(cod)acac (4.8 mg, 0.012 mmol), *tert*-butanol (0.1 mL) and KO^{t}Bu (135 mg, 1.20 mmol) were subjected to **General Procedure A.** Purification *via* column chromatography eluting

with 97.5:2.5 pentane/Et_2O afforded the title compound **3c** as a white solid (72 mg, 80%, >95:5 d.r., 92:8 e.r.). The relative stereochemistry was determined by *J*-coupling constant analysis.

m.p. = 95–98 °C

IR (film) ν_{max}/cm^{-1} 2947, 1691, 1458, 1385, 1364, 1315, 1267, 1168, 1108, 920, 908.

^{1}H NMR ($CDCl_3$, 400 MHz) δ = 2.35 (1H, ddd, J=12.4, 10.6, 3.6 Hz), 2.24 (3H, s), 2.19 (6H, s), 2.17 – 2.09 (7H, m), 1.66 (1H, dq, J=12.6, 3.0 Hz), 1.49 – 1.35 (3H, m), 1.12 – 1.02 (4H, m), 0.98 – 0.87 (7H, m).

^{13}C NMR ($CDCl_3$, 101 MHz) δ = 213.4, 139.7, 135.5, 133.1, 59.6, 47.9, 39.3, 33.0, 30.5, 28.4, 25.8, 24.6, 21.0, 18.0, 16.8, 16.1. [*N.B*. The signal for the *ortho*-quaternary aromatic carbon of the Ph* group was not observed, presumably due to restricted rotation about the Ar-CO axis].

$[\alpha]_D^{25}$ +9.7 (c = 1.00, $CHCl_3$).

HRMS (ESI^+) Found $[M+H]^+$ = 301.2525; $C_{21}H_{33}O$ requires 301.2526, Δ –0.40 ppm.

Chiral HPLC (Chiralpak IA with guard, 0.3 % IPA, 99.7 % hexane, 1.0 mL/min, 25 °C, λ = 254 nm, 10 µL injection).

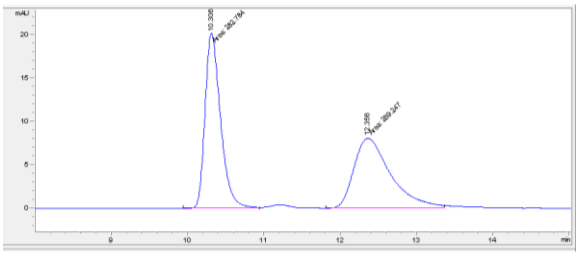

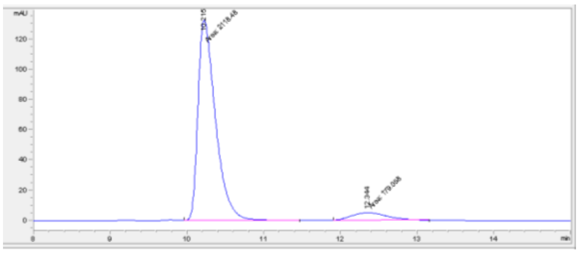

#	Time	Type	Area	Height	Width	Area%	Symmetry
1	10.306	MF	282.8	20.1	0.2341	51.226	0.675
2	12.356	MF	269.2	8	0.5599	48.774	0.588

#	Time	Type	Area	Height	Width	Area%	Symmetry
1	10.215	FM	2118.5	132.9	0.2657	92.207	0.529
2	12.344	MF	179.1	5.2	0.578	7.793	0.751

((7*R*,8*R*)-7-Methylspiro[4.5]decan-8-yl)(2,3,4,5,6-pentamethylphenyl)methanone, 3d

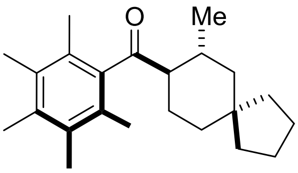

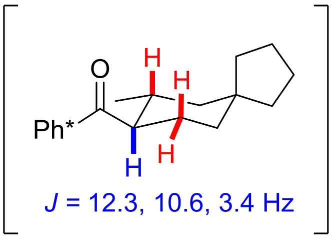

Diol **2d** (103 mg, 0.60 mmol), pentamethylacetophenone **1** (57 mg, 0.30 mmol), (*R*)-DTBM-SEGPHOS (18 mg, 0.015 mmol), Ir(cod)acac (4.8 mg, 0.012 mmol), *tert*-butanol (0.1 mL) and KO^{t}Bu (135 mg, 1.20 mmol) were subjected to **General Procedure A.** Purification *via* column chromatography eluting with 97.5:2.5 pentane/Et_2O afforded the title compound **3d** as a white solid (80 mg, 82%, >95:5 d.r., 93:7 e.r.). The relative stereochemistry was determined by *J*-coupling constant analysis.

m.p. = 112–114 °C

IR (film) ν_{max}/cm^{-1} 2930, 1690, 1446, 1382, 1310, 1263, 1148, 1108, 1002, 910.

^{1}**H NMR** ($CDCl_3$, 400 MHz) δ = 2.40 (1H, ddd, *J*=12.3, 10.6, 3.4 Hz), 2.24 (3H, s), 2.19 (6H, s), 2.17 – 2.02 (7H, m), 1.72 (1H, dq, *J*=12.7, 3.4 Hz), 1.66 – 1.26 (11H, m), 1.15 (1H, td, *J*=13.0, 3.6 Hz), 1.09 – 1.01 (4H, m).

^{13}**C NMR** ($CDCl_3$, 101 MHz) δ = 213.4, 139.7, 135.5, 133.1, 59.4, 46.7, 42.6, 42.1, 38.0, 34.8, 29.7, 27.0, 25.1, 24.0, 21.0, 18.0, 16.8, 16.1. [*N.B*. The signal for the *ortho*-quaternary aromatic carbon of the Ph* group was not observed, presumably due to restricted rotation about the Ar-CO axis].

[α]$_D^{25}$ –0.9 (c = 1.00, $CHCl_3$).

HRMS (ESI$^+$) Found $[M+H]^+$ = 327.2680; $C_{23}H_{35}O$ requires 327.2682, Δ –0.63 ppm.

Chiral HPLC (Chiralpak OD with guard, 0.3 % IPA, 99.7 % hexane, 0.5 mL/min, 25 °C, λ = 254 nm, 10 μL injection).

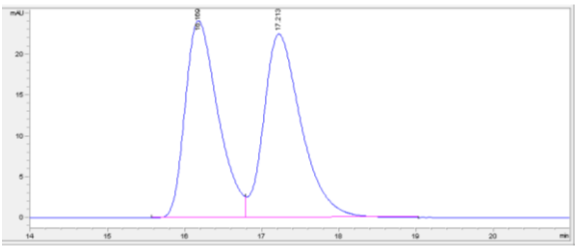

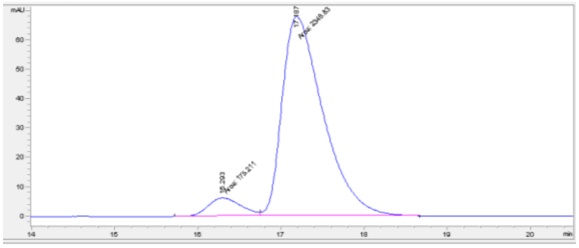

#	Time	Type	Area	Height	Width	Area%	Symmetry
1	16.169	BV	718.5	24.2	0.448	48.579	0.633
2	17.213	VB	760.5	22.6	0.5091	51.421	0.634

#	Time	Type	Area	Height	Width	Area%	Symmetry
1	16.293	MF	175.2	6.3	0.4663	6.947	0.748
2	17.187	MF	2346.8	67.9	0.5757	93.053	0.537

((2*R*,3*R*)-2-Methylspiro[5.5]undecan-3-yl)(2,3,4,5,6-pentamethylphenyl)methanone, 3e

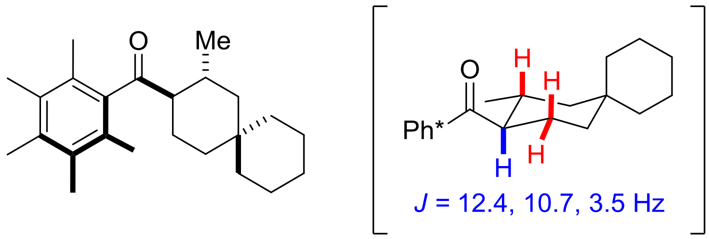

Diol **2e** (112 mg, 0.60 mmol), pentamethylacetophenone **1** (57 mg, 0.30 mmol), (*R*)-DTBM-SEGPHOS (18 mg, 0.015 mmol), Ir(cod)acac (4.8 mg, 0.012 mmol), *tert*-butanol (0.1 mL) and KO^{t}Bu (135 mg, 1.20 mmol) were subjected to **General Procedure A.** Purification *via* column chromatography eluting with 97.5:2.5 pentane/Et_2O afforded the title compound **3e** as a white solid (82 mg, 80%, >95:5 d.r., 89:11 e.r.). The relative stereochemistry was determined by *J*-coupling constant analysis.

m.p. = 112–114 °C

IR (film) ν_{max}/cm^{-1} 2924, 1689, 1448, 1382, 1303, 1257, 1110, 999, 904, 732.

^{1}H NMR ($CDCl_3$, 400 MHz) δ = 2.30 (1H, ddd, *J*=12.4, 10.7, 3.5 Hz), 2.16 (3H, s), 2.11 (6H, s), 2.09 – 1.99 (7H, m), 1.65 – 1.50 (3H, m), 1.42 – 1.24 (9H, m), 1.14 – 1.04 (2H, m), 0.98 (3H, d, *J*=6.4 Hz), 0.83 (1H, td, *J*=13.3, 3.7 Hz), 0.71 (1H, t, *J*=13.0 Hz).

^{13}C NMR ($CDCl_3$, 101 MHz) δ = 213.4, 139.8, 135.5, 133.0, 60.1, 45.5, 41.8, 36.7, 32.8, 32.3, 27.4, 26.9, 24.8, 21.7, 21.6, 21.2, 18.0, 16.8, 16.1. [*N.B.* The signal for the *ortho*-quaternary aromatic carbon of the Ph* group was not observed, presumably due to restricted rotation about the Ar-CO axis].

$[\alpha]_D^{25}$ –2.4 (c = 1.00, $CHCl_3$).

HRMS (ESI$^+$) Found $[M+H]^+$ = 341.2838; $C_{24}H_{37}O$ requires 341.2839, Δ –0.32 ppm.

Chiral HPLC (Chiralpak IA with guard, 1.0 % IPA, 99.0 % hexane, 1.0 mL/min, 25 °C, λ = 230 nm, 10 µL injection).

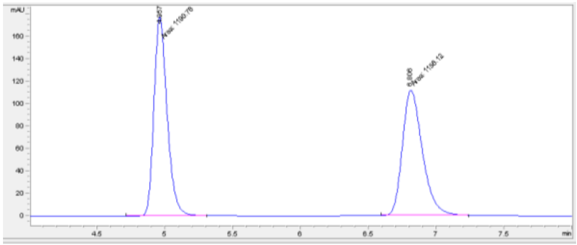

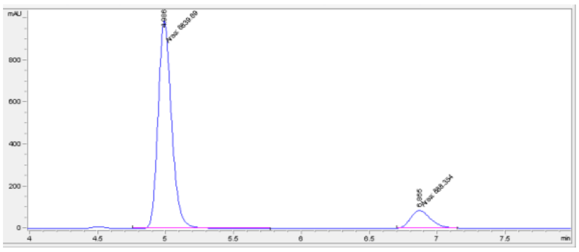

#	Time	Type	Area	Height	Width	Area%	Symmetry
1	4.957	MF	1190.8	178.1	0.1114	50.738	0.748
2	6.806	MF	1156.1	112.1	0.1718	49.262	0.719

#	Time	Type	Area	Height	Width	Area%	Symmetry
1	4.986	FM	6839.7	990.4	0.1151	88.505	0.811
2	6.865	MF	888.4	84.8	0.1747	11.495	0.735

((1*R*,2*R*)-2-Butylcyclohexyl)(2,3,4,5,6-pentamethylphenyl)methanone, 3f

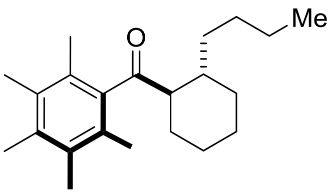

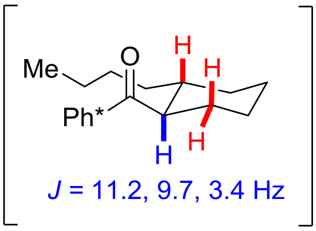

Diol **2f** (96 mg, 0.60 mmol), pentamethylacetophenone **1** (57 mg, 0.30 mmol), (*R*)-DTBM-SEGPHOS (18 mg, 0.015 mmol), Ir(cod)acac (4.8 mg, 0.012 mmol), *tert*-butanol (0.1 mL) and KO^{t}Bu (135 mg, 1.20 mmol) were subjected to **General Procedure A.** Purification *via* column chromatography eluting with 98:2 pentane/Et_2O afforded the title compound **3f** as a white solid (82 mg, 87%, 89:11 d.r., 91:9 e.r.). The relative stereochemistry was determined by *J*-coupling constant analysis.

m.p. = 95–98 °C

IR (film) ν_{max}/cm^{-1} 2926, 2854, 1682, 1447, 1380, 1305, 1261, 1137, 928, 907, 695.

^{1}H NMR ($CDCl_3$, 400 MHz) δ = 2.57 (1H, ddd, *J*=11.2, 9.7, 3.4 Hz), 2.24 (3H, s), 2.18 (6H, s), 2.14 (6H, s), 2.05 – 1.97 (1H, m), 1.88 – 1.77 (3H, m), 1.75 – 1.65 (2H, m), 1.45 – 1.00 (8H, m), 0.97 – 0.86 (4H, m). The minor diastereoisomer displays a diagnostic peak at δ = 2.80 (1H, dt, *J*=11.2, 3.4 Hz).

^{13}C NMR ($CDCl_3$, 101 MHz) δ = 213.2, 139.7, 135.5, 133.0, 58.0, 37.1, 34.1, 30.6, 29.5, 29.3, 26.5, 25.6, 22.9, 18.0, 16.8, 16.1, 14.2. [*N.B*. The signal for the *ortho*-quaternary aromatic carbon of the Ph* group was not observed, presumably due to restricted rotation about the Ar-CO axis].

$[\alpha]_D^{25}$ +6.3 (c = 1.00, $CHCl_3$).

HRMS (ESI^+) Found $[M+H]^+$ = 315.2683; $C_{22}H_{35}O$ requires 315.2682, Δ 0.31 ppm.

Chiral HPLC (Chiralpak OD with guard, 0.3 % IPA, 99.7 % hexane, 0.7 mL/min, 25 °C, λ = 320 nm, 10 µL injection). [*N.B*. the peak at 15.6 min corresponds to the minor diasteroisomer].

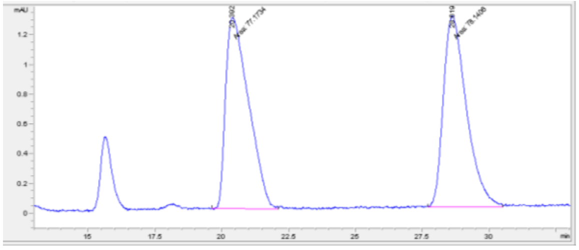

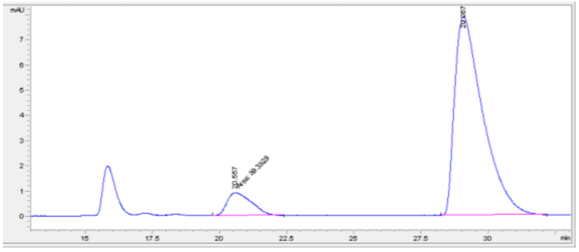

#	Time	Type	Area	Height	Width	Area%	Symmetry
1	20.392	MM	77.2	1.3	0.9992	49.689	0.421
2	28.619	MM	78.1	1.3	1.0123	50.311	0.622

#	Time	Type	Area	Height	Width	Area%	Symmetry
1	20.557	MM	59.4	9.1E-1	1.0854	9.309	0.468
2	29.067	BB	578.2	7.9	1.0038	90.691	0.428

((1*R*,2*S*)-2-Isobutylcyclohexyl)(2,3,4,5,6-pentamethylphenyl)methanone, 3g

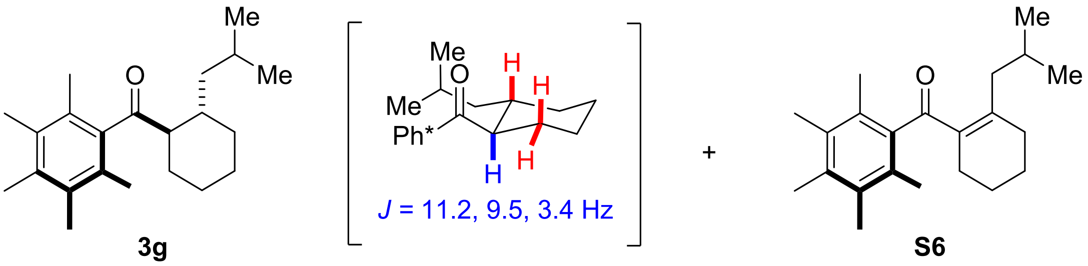

Diol **2g** (96 mg, 0.60 mmol), pentamethylacetophenone **1** (57 mg, 0.30 mmol), (*R*)-DTBM-SEGPHOS (18 mg, 0.015 mmol), Ir(cod)acac (4.8 mg, 0.012 mmol), *tert*-butanol (0.1 mL) and KO^tBu (135 mg, 1.20 mmol) were subjected to **General Procedure A.** Purification *via* column chromatography eluting with 98:2 pentane/Et_2O afforded the title compound **3g** as a white solid (23 mg, 24%, 92:8 d.r., 90:10 e.r.) along with enone **S6** as an off white gum (45 mg, 48%). The relative stereochemistry of **3g** was determined by *J*-coupling constant analysis.

<u>Data for **3g**:</u>

m.p. = 115–117 °C

IR (film) ν_{max}/cm^{-1} 2926, 2852, 1684, 1464, 1447, 1382, 1306, 1262, 1112, 925, 901.

^{1}H NMR ($CDCl_3$, 400 MHz) δ = 2.54 (1H, ddd, *J*=11.2, 9.5, 3.4 Hz), 2.24 (3H, s), 2.18 (6H, s), 2.14 (6H, s), 2.04 – 1.89 (2H, m), 1.85 – 1.64 (4H, m), 1.57 (1H, ddd, *J*=13.1, 10.6, 2.8 Hz), 1.30 – 1.11 (3H, m), 1.08 – 0.81 (8H, m). The minor diastereoisomer displays a diagnostic peak at δ = 2.80 (1H, dt, *J*=11.1, 3.5 Hz).

^{13}C NMR ($CDCl_3$, 101 MHz) δ = 213.0, 139.6, 135.5, 133.0, 58.2, 43.9, 34.8, 30.5, 29.5, 26.5, 25.5, 24.9, 24.5, 21.0, 18.0, 16.8, 16.1. [*N.B*. The signal for the *ortho*-quaternary aromatic carbon of the Ph* group was not observed, presumably due to restricted rotation about the Ar-CO axis].

$[\alpha]_D^{25}$ –1.0 (c = 1.00, $CHCl_3$).

HRMS (ESI^+) Found $[M+H]^+$ = 315.2684; $C_{22}H_{35}O$ requires 315.2682, Δ 0.41 ppm.

Chiral HPLC (Chiralpak IA with guard, 0.3 % IPA, 99.7 % hexane, 0.7 mL/min, 25 °C, λ = 254 nm, 10 µL injection). [*N.B*. the peak at 8.5 min corresponds to the minor diasteroisomer].

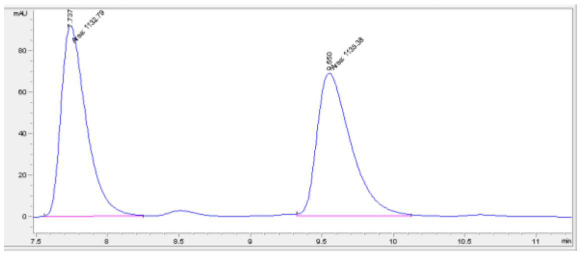

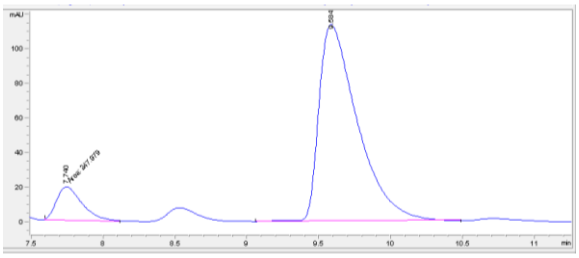

#	Time	Type	Area	Height	Width	Area%	Symmetry
1	7.737	MF	1132.8	92	0.2053	49.943	0.578
2	9.55	MF	1135.4	68.8	0.2751	50.057	0.542

#	Time	Type	Area	Height	Width	Area%	Symmetry
1	7.74	MM	248	19.4	0.2132	10.434	0.582
2	9.584	BB	2128.7	113.7	0.2804	89.566	0.428

<u>Data for **S6**:</u>

IR (film) ν_{max}/cm^{-1} 2927, 1666, 1638, 1591, 1449, 1383, 1304, 1263, 1164, 1068, 909, 739.

^{1}H NMR ($CDCl_3$, 400 MHz) δ = 2.36 (2H, d, *J*=7.5 Hz), 2.25 – 2.19 (5H, m), 2.17 (6H, s), 2.13 – 2.03 (8H, m), 1.96 (1H, non, *J*=6.8 Hz), 1.60 – 1.53 (4H, m), 0.87 (6H, d, *J*=6.6 Hz).

^{13}C NMR ($CDCl_3$, 101 MHz) δ = 203.9, 152.4, 141.0, 134.7, 133.4, 132.7, 128.2, 43.6, 33.0, 27.6, 27.5, 22.8, 22.7, 22.2, 17.1, 16.7, 16.0.

HRMS (ESI^+) Found $[M+H]^+$ = 313.2529; $C_{22}H_{33}O$ requires 313.2526, Δ 0.88 ppm.

(2,3,4,5,6-Pentamethylphenyl)((1*R*,2*S*)-2-phenethylcyclohexyl)methanone, 3h

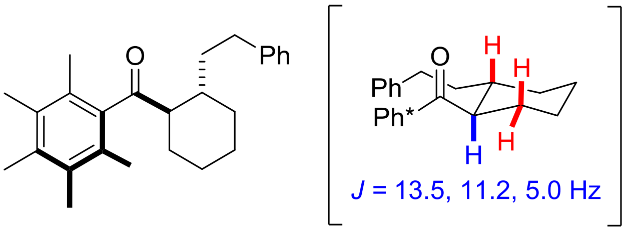

Diol **2h** (125 mg, 0.60 mmol), pentamethylacetophenone **1** (57 mg, 0.30 mmol), (*R*)-DTBM-SEGPHOS (18 mg, 0.015 mmol), Ir(cod)acac (4.8 mg, 0.012 mmol), *tert*-butanol (0.1 mL) and KO^{t}Bu (135 mg, 1.20 mmol) were subjected to **General Procedure A.** Purification *via* column chromatography eluting with 97:3 pentane/Et_2O afforded the title compound **3h** as a white solid (65 mg, 60%, >95:5 d.r., 87:13 e.r.). The relative stereochemistry was determined by *J*-coupling constant analysis.

m.p. = 155–158 °C

IR (film) ν_{max}/cm^{-1} 2930, 2854, 1675, 1496, 1449, 1308, 1262, 1117, 932, 913, 746, 697.

^{1}H NMR ($CDCl_3$, 400 MHz) δ = 7.23 – 7.06 (5H, m), 2.70 (1H, ddd, *J*=13.5, 11.2, 5.0 Hz), 2.61 – 2.49 (2H, m), 2.20 – 1.96 (17H, m), 1.85 (1H, qt, *J*=10.0, 3.4 Hz), 1.74 (1H, br d, *J*=9.6 Hz), 1.69 – 1.59 (2H, m), 1.35 – 1.03 (4H, m), 0.95 (1H, qd, *J*=12.6, 3.1 Hz).

^{13}C NMR ($CDCl_3$, 101 MHz) δ = 213.0, 142.8, 139.6, 135.6, 133.1, 128.5, 128.3, 125.6, 57.8, 37.3, 36.2, 33.6, 30.7, 29.3, 26.4, 25.5, 18.1, 16.9, 16.1. [*N.B*. The signal for the *ortho*-quaternary aromatic carbon of the Ph* group was not observed, presumably due to restricted rotation about the Ar-CO axis].

$[\alpha]_D^{25}$ +17.3 (c = 1.00, $CHCl_3$).

HRMS (ESI$^+$) Found $[M+Na]^+$ = 385.2500; $C_{26}H_{34}ONa$ requires 385.2502, Δ –0.41 ppm.

Chiral HPLC (Chiralpak IA with guard, 0.3 % IPA, 99.7 % hexane, 1.0 mL/min, 25 °C, λ = 254 nm, 10 μL injection).

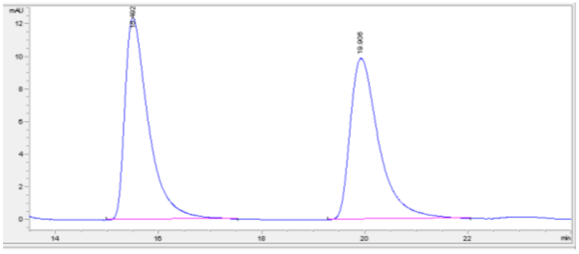

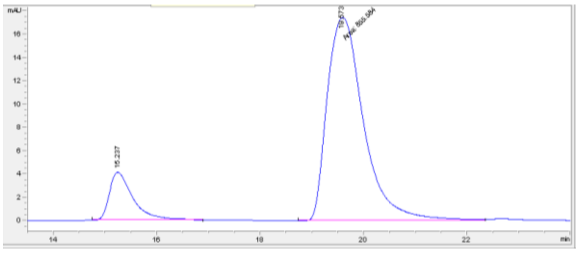

#	Time	Type	Area	Height	Width	Area%	Symmetry
1	15.492	BB	395.2	12.4	0.4692	50.578	0.465
2	19.906	BB	386.1	9.9	0.5707	49.422	0.547

#	Time	Type	Area	Height	Width	Area%	Symmetry
1	15.237	BB	131.7	4.1	0.4632	13.339	0.508
2	19.573	MF	855.6	17.5	0.8167	86.661	0

((1*R*,2*S*)-2-(3-(Furan-2-yl)propyl)cyclohexyl)(2,3,4,5,6-pentamethylphenyl)methanone, 3i

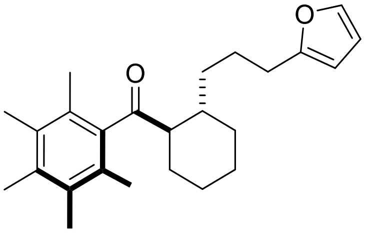

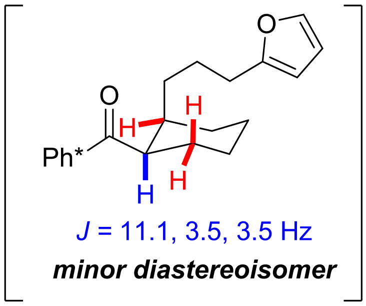

Diol **2i** (128 mg, 0.60 mmol), pentamethylacetophenone **1** (57 mg, 0.30 mmol), (*R*)-DTBM-SEGPHOS (18 mg, 0.015 mmol), Ir(cod)acac (4.8 mg, 0.012 mmol), *tert*-butanol (0.1 mL) and KO^{t}Bu (135 mg, 1.20 mmol) were subjected to **General Procedure A.** Purification *via* column chromatography eluting with 97.5:2.5 pentane/Et_2O afforded the title compound **3i** as a white solid (75 mg, 68%, 87:13 d.r., 88:12 e.r.). The relative stereochemistry of the major diastereoisomer could not be determined directly, but was assigned as *trans* on the basis of J-coupling constant analysis of the minor diastereoisomer which revealed a *cis*-arrangement.

m.p. = 85–87 °C

IR (film) ν_{max}/cm^{-1} 2926, 1688, 1507, 1447, 1382, 1305, 1261, 1146, 1003, 916, 798, 726.

^{1}H NMR ($CDCl_3$, 400 MHz) δ = 7.30 (1H, dd, *J*=1.9, 0.9 Hz), 6.28 (1H, dd, *J*=3.2, 1.8 Hz), 6.00 (1H, dd, *J*=3.1, 1.0 Hz), 2.75 – 2.54 (3H, m), 2.24 (3H, s), 2.19 (6H, s), 2.13 (6H, s), 2.07 – 1.99 (1H, m), 1.94 –

1.53 (7H, m), 1.30 – 1.07 (4H, m), 1.00 – 0.88 (1H, m). The minor diastereoisomer displays a diagnostic peak at δ = 2.80 (1H, dt, J=11.1, 3.5 Hz).

^{13}C NMR ($CDCl_3$, 101 MHz) δ = 213.1, 156.6, 140.6, 139.6, 135.6, 133.1, 110.1, 104.6, 57.9, 37.0, 34.0, 30.6, 29.4, 28.2, 26.4, 25.7, 25.5, 18.0, 16.8, 16.1. [*N.B*. The signal for the *ortho*-quaternary aromatic carbon of the Ph* group was not observed, presumably due to restricted rotation about the Ar-CO axis].

$[\alpha]_D^{25}$ +6.0 (c = 1.00, $CHCl_3$).

HRMS (ESI^+) Found $[M+H]^+$ = 367.2631; $C_{25}H_{35}O_2$ requires 367.2632, Δ –0.18 ppm.

Chiral HPLC (Chiralpak OD with guard, 0.5 % IPA, 99.5 % hexane, 1.0 mL/min, 25 °C, λ = 210 nm, 10 µL injection). [*N.B*. the peaks 12.0 and 12.5 min correspond to the minor diasteroisomer].

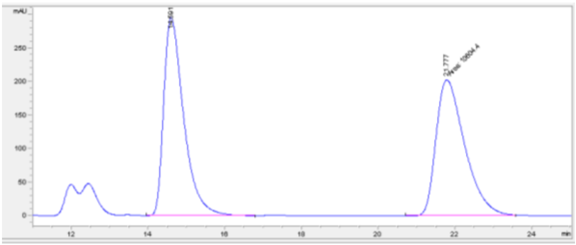

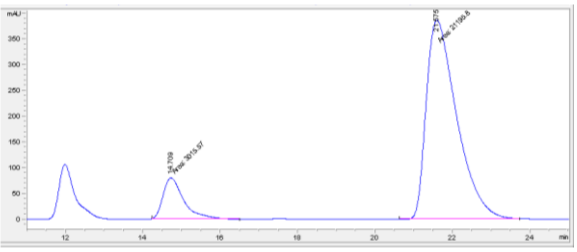

#	Time	Type	Area	Height	Width	Area%	Symmetry
1	14.591	BB	10699.5	295.2	0.5488	50.223	0.548
2	21.777	MM	10604.4	202.7	0.872	49.777	0.585

#	Time	Type	Area	Height	Width	Area%	Symmetry
1	14.709	MF	3015.6	80.1	0.6276	12.455	0.587
2	21.575	MF	21195.8	386.2	0.9146	87.545	0.514

((1*R*,2*S*)-2-(3-Methoxypropyl)cyclohexyl)(2,3,4,5,6-pentamethylphenyl)methanone, 3j

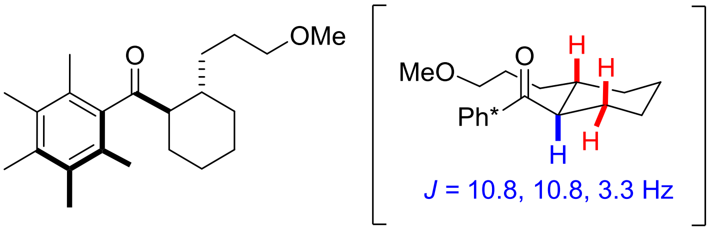

Diol **2j** (106 mg, 0.60 mmol), pentamethylacetophenone **1** (57 mg, 0.30 mmol), (*R*)-DTBM-SEGPHOS (18 mg, 0.015 mmol), Ir(cod)acac (4.8 mg, 0.012 mmol), *tert*-butanol (0.1 mL) and KO^{t}Bu (135 mg, 1.20 mmol) were subjected to **General Procedure A** [*N.B*. in this case *tert*-butyl hydroperoxide was not added to the crude filtrate]**.** Purification *via* column chromatography eluting with 90:10

pentane/Et_2O afforded the title compound **3j** as a white solid (85 mg, 86%, 88:12 d.r., 90:10 e.r.). The relative stereochemistry was determined by *J*-coupling constant analysis.

m.p. = 77–80 °C

IR (film) ν_{max}/cm^{-1} 2925, 2854, 1689, 1447, 1384, 1260, 1117.

^{1}H NMR ($CDCl_3$, 400 MHz) δ = 3.44 (1H, ddd, *J*=9.1, 7.5, 5.6 Hz), 3.39 – 3.32 (4H, m), 2.56 (1H, td, *J*=10.8, 3.3 Hz), 2.23 (3H, s), 2.18 (6H, s), 2.12 (6H, s), 2.07 – 1.98 (1H, m), 1.89 – 1.53 (7H, m), 1.29 – 1.06 (4H, m), 1.00 – 0.88 (1H, m). The minor diastereoisomer displays a diagnostic peak at δ = 2.79 (1H, dt, J=11.2, 3.4 Hz).

^{13}C NMR ($CDCl_3$, 101 MHz) δ = 213.1, 139.5, 135.5, 133.1, 73.2, 58.5, 57.8, 37.0, 30.7, 30.5, 29.4, 27.2, 26.4, 25.5, 18.0, 16.8, 16.1. [*N.B*. The signal for the *ortho*-quaternary aromatic carbon of the Ph* group was not observed, presumably due to restricted rotation about the Ar-CO axis].

$[\alpha]_D^{25}$ +7.7 (c = 1.00, $CHCl_3$).

HRMS (ESI^+) Found $[M+H]^+$ = 331.2628; $C_{22}H_{35}O_2$ requires 331.2632, Δ –1.02 ppm.

Chiral HPLC (Chiralpak OD with guard, 1 % IPA, 99 % hexane, 1.0 mL/min, 25 °C, λ = 254 nm, 10 µL injection). [*N.B*. the peak at 7.2 min corresponds to the minor diasteroisomer].

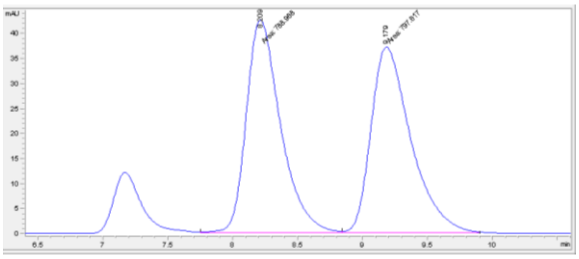

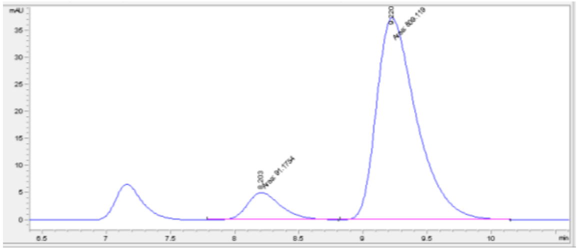

#	Time	Type	Area	Height	Width	Area%	Symmetry
1	8.209	FM	789	42.5	0.3093	49.727	0.677
2	9.179	MF	797.6	37.1	0.3582	50.273	0.615

#	Time	Type	Area	Height	Width	Area%	Symmetry
1	8.203	MM	91.2	5	0.3033	10.127	0.712
2	9.22	MM	809.1	37.4	0.3604	89.873	0.611

((1*R*,2*S*)-2-(3-(Methylthio)propyl)cyclohexyl)(2,3,4,5,6-pentamethylphenyl)methanone, 3k

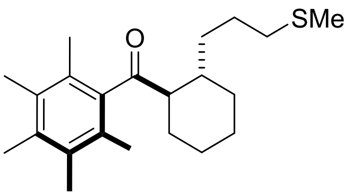

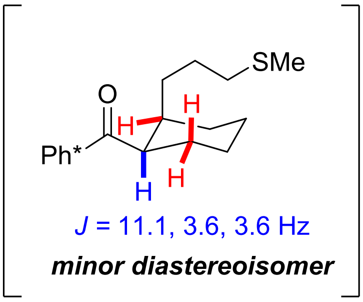

Diol **2k** (115 mg, 0.60 mmol), pentamethylacetophenone **1** (57 mg, 0.30 mmol), (*R*)-DTBM-SEGPHOS (18 mg, 0.015 mmol), Ir(cod)acac (4.8 mg, 0.012 mmol), *tert*-butanol (0.1 mL) and KO^{t}Bu (135 mg, 1.20 mmol) were subjected to **General Procedure A** [*N.B*. in this case *tert*-butyl hydroperoxide was not added to the crude filtrate]. Purification *via* column chromatography eluting with 98:2 pentane/Et_2O afforded the title compound **3k** as a white solid contaminated with 2% of (*R*)-DTBM-SEGPHOS (79 mg, 71%, 88:12 d.r., 89:11 e.r.). The relative stereochemistry of the major diastereoisomer could not be determined directly, but was assigned as *trans* on the basis of J-coupling constant analysis of the minor diastereoisomer which revealed a *cis*-arrangement.

m.p. = 81–83 °C

IR (film) ν_{max}/cm^{-1} 2922, 2853, 1688, 1446, 1382, 1303, 1260, 1233, 1111, 913.

^{1}H NMR ($CDCl_3$, 400 MHz) δ = 2.61 – 2.42 (3H, m), 2.24 (3H, s), 2.18 (6H, s), 2.13 (6H, s), 2.11 (3H, s), 2.05 – 1.97 (1H, m), 1.94 – 1.57 (7H, m), 1.28 – 1.10 (4H, m), 0.95 (1H, qd, *J*=11.9, 3.4 Hz). The minor diastereoisomer displays a diagnostic peak at δ = 2.78 (1H, dt, J=11.1, 3.6 Hz).

^{13}C NMR ($CDCl_3$, 101 MHz) δ = 213.0, 139.5, 135.6, 133.1, 57.8, 36.9, 34.5, 33.8, 30.7, 29.4, 27.0, 26.4, 25.5, 18.0, 16.8, 16.1, 15.6. [*N.B*. The signal for the *ortho*-quaternary aromatic carbon of the Ph* group was not observed, presumably due to restricted rotation about the Ar-CO axis].

$[\alpha]_D^{25}$ +1.9 (c = 1.00, $CHCl_3$).

HRMS (ESI$^+$) Found $[M+Na]^+$ = 369.2220; $C_{22}H_{34}ONaS$ requires 369.2223, Δ –0.66 ppm.

Chiral HPLC (Chiralpak IA with guard, 1 % IPA, 99 % hexane, 1.0 mL/min, 25 °C, λ = 254 nm, 10 µL injection). [*N.B.* the peaks at 7.4 and 8.4 min correspond to the minor diasteroisomer (~88:12 e.r.)].

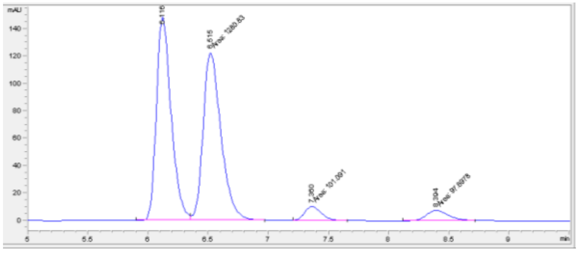

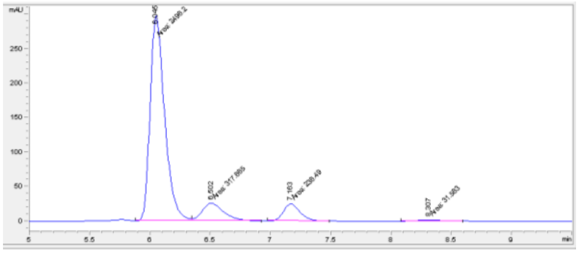

#	Time	Type	Area	Height	Width	Area%	Symmetry
1	6.116	BV	1272.8	148.8	0.1298	46.243	0.676
2	6.515	MF	1280.8	123	0.1736	46.535	0.668
3	7.36	FM	101.1	10.4	0.1614	3.673	0.774
4	8.394	MF	97.7	7.5	0.217	3.550	0.735

#	Time	Type	Area	Height	Width	Area%	Symmetry
1	6.045	MF	2496.2	296.6	0.1403	80.995	0.678
2	6.502	FM	317.7	25.7	0.2061	10.307	0.677
3	7.163	MF	236.5	24.5	0.1606	7.673	0.755
4	8.307	MF	31.6	1.8	0.2894	1.024	0.632

((1*R*,2*S*)-2-(2-(1,3-Dioxolan-2-yl)ethyl)cyclohexyl)(2,3,4,5,6-pentamethylphenyl)methanone, 3l

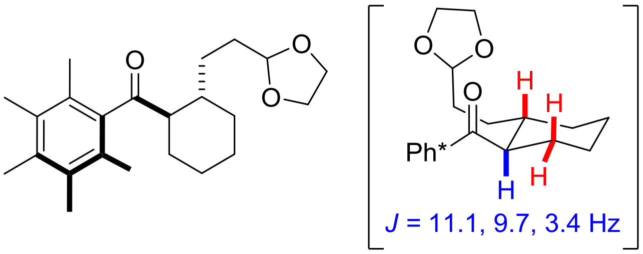

Diol **2l** (123 mg, 0.60 mmol), pentamethylacetophenone **1** (57 mg, 0.30 mmol), (*R*)-DTBM-SEGPHOS (18 mg, 0.015 mmol), Ir(cod)acac (4.8 mg, 0.012 mmol), *tert*-butanol (0.1 mL) and KO^{t}Bu (135 mg, 1.20 mmol) were subjected to **General Procedure A** [*N.B.* in this case *tert*-butyl hydroperoxide was not added to the crude filtrate]**.** Purification *via* column chromatography eluting with 85:15 pentane/Et_2O afforded the title compound **3l** as a white solid (86 mg, 80%, 87:13 d.r., 86:14 e.r.). The relative stereochemistry was determined by *J*-coupling constant analysis.

m.p. = 95–98 °C

IR (film) ν_{max}/cm^{-1} 2926, 1688, 1447, 1409, 1384, 1307, 1260, 1127, 942, 915, 732.

^{1}H NMR ($CDCl_3$, 400 MHz) δ = 4.88 (1H, dd, *J*=5.5, 4.2 Hz), 4.00 – 3.92 (2H, m), 3.88 – 3.82 (2H, m), 2.57 (1H, ddd, *J*=11.1, 9.7, 3.4 Hz), 2.23 (3H, s), 2.18 (6H, s), 2.13 (6H, s), 2.04 – 1.92 (2H, m), 1.91 –

1.62 (6H, m), 1.31 – 1.11 (4H, m), 0.96 (1H, qd, *J*=12.1, 3.4 Hz). The minor diastereoisomer displays a diagnostic peak at δ = 2.80 (1H, dt, *J*=11.1, 3.5 Hz).

^{13}C NMR ($CDCl_3$, 101 MHz) δ = 212.9, 139.5, 135.5, 133.0, 105.0, 64.9, 64.8, 57.7, 37.1, 31.7, 30.4, 29.2, 28.7, 26.3, 25.4, 18.0, 16.8, 16.1. [*N.B*. The signal for the *ortho*-quaternary aromatic carbon of the Ph* group was not observed, presumably due to restricted rotation about the Ar-CO axis].

$[\alpha]_D^{25}$ +4.8 (c = 1.00, $CHCl_3$).

HRMS (ESI^+) Found $[M+H]^+$ = 359.2579; $C_{23}H_{35}O_3$ requires 359.2581, Δ –0.38 ppm.

Chiral HPLC (Chiralpak IA with guard, 1 % IPA, 99 % hexane, 1.0 mL/min, 25 ^{o}C, λ = 230 nm, 10 µL injection). [*N.B*. the peaks at 16.6 and 17.2 min correspond to the minor diastereoisomer].

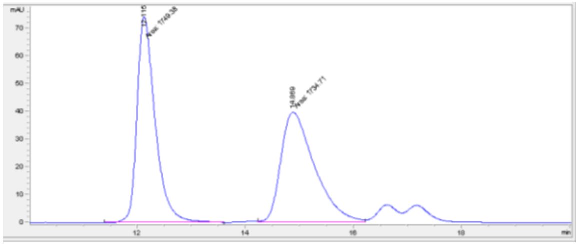

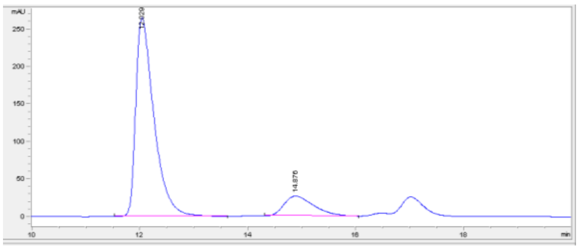

#	Time	Type	Area	Height	Width	Area%	Symmetry
1	12.115	MF	1749.4	74.3	0.3925	50.210	0.62
2	14.869	FM	1734.7	39.8	0.7267	49.790	0.552

#	Time	Type	Area	Height	Width	Area%	Symmetry
1	12.029	BB	6218.2	264.4	0.3543	85.979	0.48
2	14.876	BB	1014	26.4	0.5845	14.021	0.591

6-Isopropyl-2-methylbicyclo[4.1.0]heptan-3-yl)(2,3,4,5,6-pentamethylphenyl)methanone, 3m

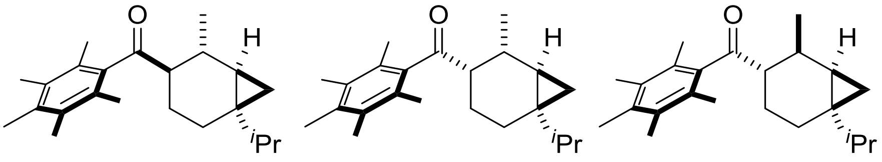

3m' **3m''** **3m'''**

With an achiral Ir(III) catalyst:[3] Under an air atmosphere, a 2–5 mL Biotage® microwave vial equipped with a stirrer bar, was sequentially charged with diol **2m** (207 mg, 1.20 mmol), pentamethylacetophenone **1** (114 mg, 0.60 mmol), $[Cp^*IrCl_2]_2$ (9.6 mg, 0.012 mmol), PhMe (0.15 mL) and KOH (135 mg, 2.40 mmol). The reaction vessel was sealed with a microwave vial cap (containing

a Reseal™ septum) and the vial was heated to 115 °C in a preheated oil bath for 24 h. The mixture was cooled to RT and filtered through a SiO_2 plug (eluting with ~50 mL Et_2O) and the filtrate was concentrated under reduced pressure. Purification *via* column chromatography eluting with 96:4→90:10 pentane/Et_2O afforded the title compound **3m** as a white solid (85 mg, 44%, 51:7:42 **3m'**:**3m''**:**3m'''**). An analytical quantity of each diastereoisomer was obtained by preparative TLC (3:97 Et_2O/pentane). The relative stereochemistry of each diastereoisomer was established by nOe and J-coupling constant analysis (see details below).

<u>With (*R*)-DTBM-SEGPHOS (matched):</u> Diol **2m** (103 mg, 0.60 mmol), pentamethylacetophenone **1** (57 mg, 0.30 mmol), (*R*)-DTBM-SEGPHOS (18 mg, 0.015 mmol), Ir(cod)acac (4.8 mg, 0.012 mmol), *tert*-butanol (0.1 mL) and KO^tBu (135 mg, 1.20 mmol) were subjected to **General Procedure A**. Purification *via* column chromatography eluting with 97.5:2.5 pentane/Et_2O afforded the title compound **3m** as a white solid (82 mg, 84%, 90:10:<5 **3m'**:**3m''**:**3m'''**). The d.r. was determined by quantitative ^{13}C NMR ($CDCl_3$, 126 MHz, relaxation delay = 60 s) by integration of the signals at 57.7, 50.6 and 54.5 ppm.

<u>With (*S*)-DTBM-SEGPHOS (mismatched):</u> Diol **2m** (103 mg, 0.60 mmol), pentamethylacetophenone **1** (57 mg, 0.30 mmol), (*S*)-DTBM-SEGPHOS (18 mg, 0.015 mmol), Ir(cod)acac (4.8 mg, 0.012 mmol), *tert*-butanol (0.1 mL) and KO^tBu (135 mg, 1.20 mmol) were subjected to **General Procedure A**. Purification *via* column chromatography eluting with 97.5:2.5 pentane/Et_2O afforded the title compound **3m** as a white solid (81 mg, 83%, 58:5:37 **3m'**:**3m''**:**3m'''**). The d.r. was determined by quantitative ^{13}C NMR ($CDCl_3$, 126 MHz, relaxation delay = 60 s) by integration of the signals at 57.7, 50.6 and 54.5 ppm.

<u>Data for **3m'**:</u>

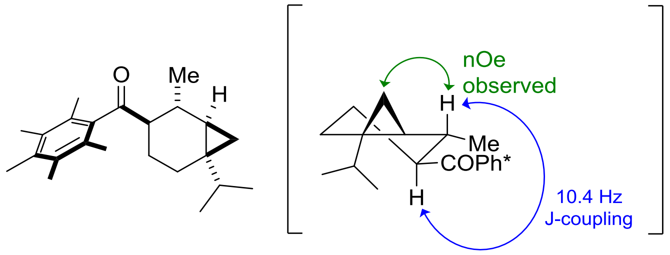

IR (film) ν_{max}/cm^{-1} 2954, 2870, 1690, 1463, 1382, 1304, 1129, 1084, 1005, 880.

^{1}H NMR ($CDCl_3$, 500 MHz) δ = 2.32 – 2.22 (4H, m), 2.18 (6H, s), 2.15 – 2.02 (6H, br s), 2.12 (1H, ddd, *J*=12.5, 10.4, 2.6 Hz), 1.73 – 1.63 (2H, m), 1.48 – 1.40 (1H, m), 1.19 (3H, d, *J*=6.8 Hz), 1.02 (1H, qd, *J*=13.2, 4.8 Hz), 0.93 – 0.85 (7H, m), 0.38 (1H, dd, *J*=9.0, 4.0 Hz), 0.31 (1H, ddd, *J*=9.0, 5.0, 1.3 Hz), 0.26 (1H, t, *J*=4.7 Hz).

^{13}C NMR ($CDCl_3$, 126 MHz) δ = 213.6, 139.8, 135.4, 133.0, 129.1 (br), 57.7, 37.4, 30.6, 26.0, 25.0, 23.6, 23.5, 23.0, 19.1, 18.7, 17.9, 16.9, 16.8, 16.1.

HRMS (ESI+) Found $[M+H]^+$ = 327.2684; $C_{23}H_{35}O$ requires 327.2682, Δ 0.4 ppm.

$[\alpha]_D^{25}$ +34.7 (c = 0.62, $CHCl_3$).

Data for **3m''**:

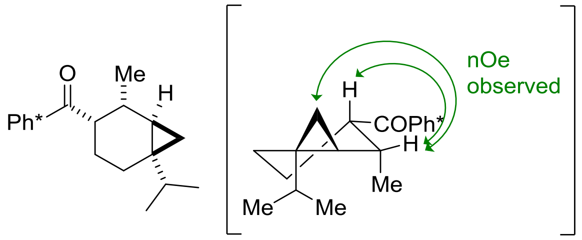

IR (film) ν_{max}/cm^{-1} 2954, 2871, 1691, 1464, 1381, 1128, 1065, 1019, 860.

^{1}H NMR ($CDCl_3$, 500 MHz) δ = 2.53 – 2.44 (2H, m), 2.23 (3H, s), 2.17 (6H, s), 2.07 (6H, s), 1.89 (1H, dd, *J*=13.8, 6.6 Hz), 1.64 – 1.49 (1H, m), 1.44 – 1.32 (2H, m), 1.18 (3H, d, *J*=6.6 Hz), 0.90 (3H, d, *J*=6.7 Hz), 0.87 (3H, d, *J*=6.7 Hz), 0.77 (1H, sept, *J*=6.6 Hz), 0.62 (1H, ddd, *J*=9.2, 5.7, 1.5 Hz), 0.34 (1H, dd, *J*=9.1, 4.8 Hz), 0.16 (1H, t, *J*=5.3 Hz).

^{13}C NMR ($CDCl_3$, 126 MHz) δ = 215.3, 140.7, 135.4, 133.1, 128.3 (br), 50.7, 38.6, 29.9, 28.9, 27.2, 24.3, 23.3, 19.1, 19.0, 18.4, 17.9, 17.4, 16.9, 16.2.

HRMS (ESI+) Found $[M+H]^+$ = 327.2685; $C_{23}H_{35}O$ requires 327.2682, Δ 0.82 ppm.

$[\alpha]_D^{25}$ –13.6 (c = 0.62, $CHCl_3$).

Data for **3m'''**:

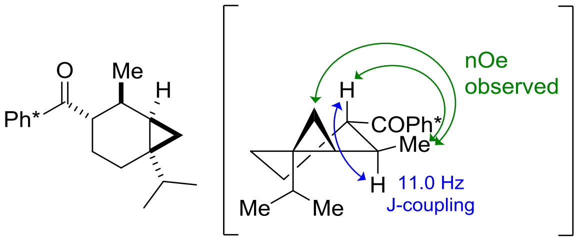

IR (film) ν_{max}/cm^{-1} 2953, 2870, 1693, 1464, 1382, 1364, 1304, 1123, 1070, 860.

^{1}H NMR ($CDCl_3$, 500 MHz) δ = 2.55 (1H, dqd, *J*=11.0, 6.5, 5.9 Hz), 2.23 (3H, s), 2.17 (6H, s), 2.16 – 2.04 (7H, m), 1.88 (1H, ddd, *J*=14.2, 5.2, 2.8 Hz), 1.59 (1H, ddt, *J*=12.9, 5.5, 2.8 Hz), 1.19 – 1.13 (1H, m), 1.12 (3H, d, *J*=6.5 Hz), 1.02 (1H, qd, *J*=12.4, 5.2 Hz), 0.90 – 0.85 (7H, m), 0.82 (1H, dt, *J*=9.2, 5.9 Hz), 0.32 (1H, dd, *J*=9.2, 4.4 Hz), -0.04 (1H, dd, *J*=5.8, 4.5 Hz).

^{13}C NMR ($CDCl_3$, 126 MHz) δ = 212.9, 139.9, 135.5, 133.1, 128.8 (br), 54.5, 38.3, 28.8, 27.6, 26.5, 25.7, 25.4, 20.2, 18.9, 18.3, 17.9, 16.8, 16.1, 16.1.

HRMS (ESI+) Found $[M+H]^+$ = 327.2687; $C_{23}H_{35}O$ requires 327.2682, Δ 1.33 ppm.

$[\alpha]_D^{25}$ –2.9 (c = 0.63, $CHCl_3$).

((1*R*,2*R*)-2-Methylcyclopentyl)(2,3,4,5,6-pentamethylphenyl)methanone, 3n

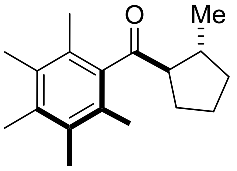

1,4-Pentanediol **2n** (63 mg, 0.60 mmol), pentamethylacetophenone **1** (57 mg, 0.30 mmol), (*R*)-DTBM-SEGPHOS (18 mg, 0.015 mmol), Ir(cod)acac (4.8 mg, 0.012 mmol), *tert*-butanol (0.1 mL) and KO^{t}Bu (135 mg, 1.20 mmol) were subjected to **General Procedure A.** Purification *via* column chromatography eluting with 97:3 pentane/Et_2O afforded the title compound **3n** as a white solid (33 mg, 43%, >95:5 d.r., 90:10 e.r.). The spectral data matched that previously reported in the literature.[3]

m.p. = 58–60 °C

IR (film) ν_{max}/cm^{-1} 2954, 2868, 1686, 1573, 1450, 1405, 1379, 1360, 1344, 1329, 1305, 1259, 1234,

1163, 1121, 1093, 1081, 1067, 1019, 1001, 972.

^{1}H NMR ($CDCl_3$, 400 MHz) δ = 2.78 (1H, q, J = 8.0 Hz), 2.43–2.31 (1H, m), 2.23 (3H, s), 2.18 (6H, s), 2.12 (6H, s), 1.99–1.82 (3H, m), 1.74–1.60 (2H, m), 1.30–1.20 (1H, m), 1.01 (3H, d, J = 6.7 Hz).

^{13}C NMR ($CDCl_3$, 101 MHz) δ = 214.5, 141.1, 135.3, 133.1, 127.9, 61.8, 37.2, 35.2, 30.1, 24.9, 20.7, 17.9, 16.8, 16.1.

$[\alpha]_D^{25}$ –27.2 (c = 1.00, $CHCl_3$).

HRMS (ESI^+) Found $[M+H]^+$ = 259.2058; $C_{18}H_{27}O$ requires 259.2056, Δ 0.77 ppm.

Chiral HPLC (Chiralpak OD with guard, 0.1 % IPA, 99.9 % hexane, 1.0 mL/min, 25 °C, λ = 254 nm, 10 μL injection).

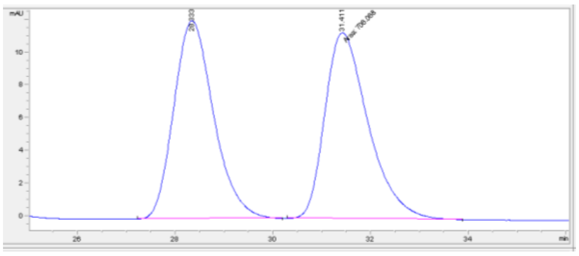

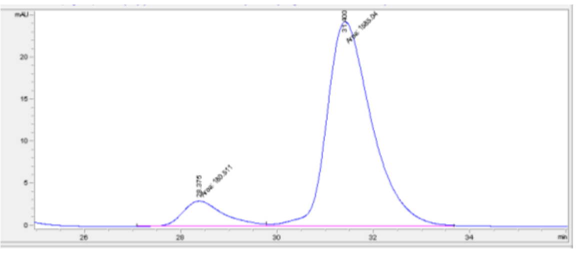

#	Time	Type	Area	Height	Width	Area%	Symmetry
1	28.333	BB	689.8	12.1	0.8384	49.418	0.797
2	31.411	MF	706.1	11.4	1.0305	50.582	0.655

#	Time	Type	Area	Height	Width	Area%	Symmetry
1	28.375	MF	180.5	3	0.9988	10.224	0.658
2	31.4	MF	1585	24.4	1.0831	89.776	0.665

4.4 *Experiments from Scheme 2 and Additional Studies*

(a) Gram scale hydrogen borrowing reaction

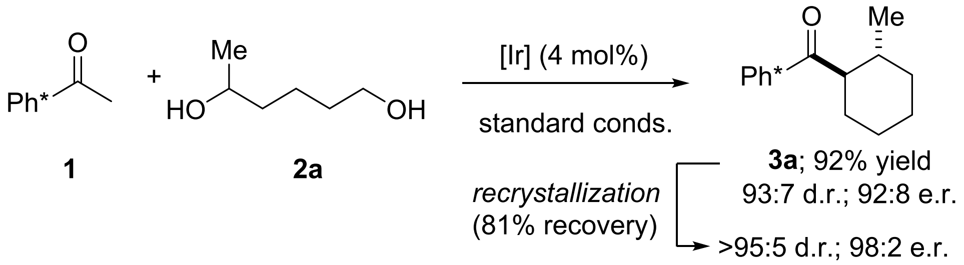

Under an air atmosphere, a 100 mL ACE pressure tube equipped with a stirrer bar, was sequentially charged with diol **2a** (1.24 g, 10.5 mmol), pentamethylacetophenone **1** (1.00 g, 5.25 mmol), (*R*)-DTBM SEGPHOS (310 mg, 0.26 mmol), Ir(cod)acac (42 mg, 0.11 mmol), *tert*-butanol (5.3 mL) and KO^tBu (2.35 g, 21.0 mmol). The reaction vessel was sealed and the vial was heated to 110 ^oC in a preheated oil bath for 24 h. The mixture was cooled to RT and filtered through a SiO_2 plug (eluting with Et_2O). For ease of purification from residual (*R*)-DTBM-SEGPHOS, the crude ethereal solution was treated with *tert*-butyl hydroperoxide (5-6 M in decane, 0.5 mL, ~2.75 mmol), swirled and allowed to stand at RT for 10 min and then concentrated under reduced pressure. Purification *via* column chromatography eluting with 95:5 pentane/Et_2O to afforded the title compound **3a** as a white solid (1.32 g, 92%, 93:7 d.r., 92:8 e.r.). Recrystallization from boiling methanol afforded the title compound **3a** as a white needles (1.07 g, 81% recovery, >95:5 d.r.; 98:2 e.r.). [*N.B.* concentration of the mother liquor under reduced pressure afforded **3a** (238 mg, 75:25 d.r.; 50:50 e.r.).]. The spectral data for **3a** was identical to that described above.

$[\alpha]_D^{25}$ +19.1 (c = 1.00, $CHCl_3$, >95:5 d.r., 98:2 e.r.).

Chiral HPLC (Chiralpak OD with guard, 0.3 % IPA, 99.7 % hexane, 0.7 mL/min, 25 °C, λ = 254 nm, 10 µL injection) [*N.B.* the peaks at 16.7 and 17.3 min correspond to the minor diasteroisomer].

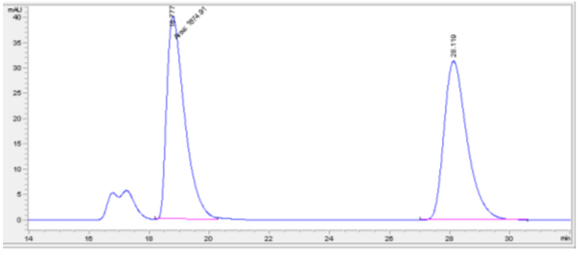

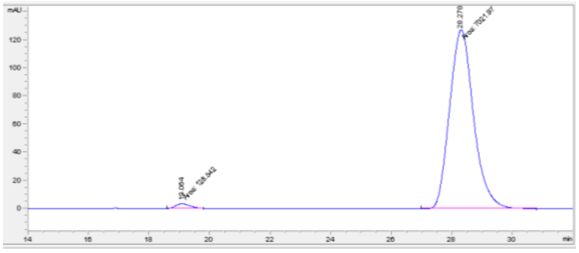

#	Time	Type	Area	Height	Width	Area%	Symmetry
1	18.777	MF	1674.9	40.1	0.6963	50.005	0.512
2	28.119	BB	1674.6	31.3	0.8095	49.995	0.66

#	Time	Type	Area	Height	Width	Area%	Symmetry
1	19.064	FM	128.5	3.6	0.5957	1.798	0.706
2	28.278	MF	7022	127.7	0.9166	98.202	0.902

(b) Resubjection of a cyclic enone

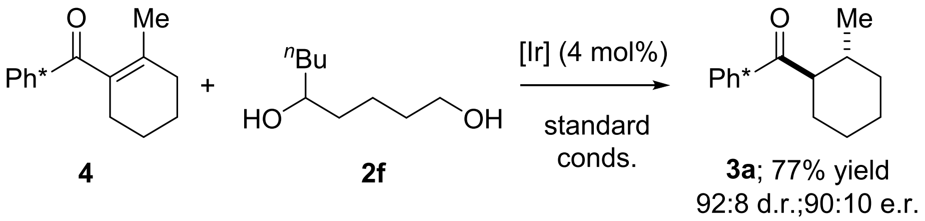

Under an air atmosphere, a 2–5 mL Biotage® microwave vial equipped with a stirrer bar, was sequentially charged with diol **2f** (96 mg, 0.60 mmol), enone **4** (81 mg, 0.30 mmol), (*R*)-DTBM SEGPHOS (18 mg, 0.015 mmol), Ir(cod)acac (2.4 mg, 0.006 mmol), *tert*-butanol (0.3 mL) and KO^{t}Bu (135 mg, 1.20 mmol). The reaction vessel was sealed with a microwave vial cap (containing a Reseal™ septum) and the vial was heated to 110 °C in a preheated oil bath for 24 h. The mixture was cooled to RT and filtered through a SiO_2 plug (eluting with Et_2O). The resulting solution was concentrated under reduced pressure. A combination of reverse and normal phase HPLC analysis (see optimization section for details) indicated **3a** to be formed in 77% yield, 92:8 d.r. and 90:10 e.r. The spectral data for **3a** was identical to that described above.

(c) Synthesis of ((1*R*,2*R*)-2-Methylcyclohexyl)methanol, (*R*,*R*)-6

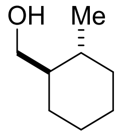

A stirred solution of ketone **3a** (109 mg, 0.40 mmol, >95:5 d.r., 98:2 e.r.) in CH_2Cl_2 (2 mL) was cooled to –17 °C (ice/NaCl bath). Following this, Br_2 (41 μL, 0.80 mmol) was added dropwise and the resulting solution was stirred at –17 °C for 15 min. The reaction mixture was then warmed to RT (by placing in a RT water bath) and the majority of the volatiles were removed under a stream of nitrogen and the resulting solid was dried *in vacuo* (0.2 mmHg) for 2 min. The residue was dissolved in THF (2 mL) and the resulting stirred solution was cooled to 0 °C. $LiAlH_4$ (76 mg, 2.00 mmol) was added in a single portion and the reaction mixture was warmed to RT and stirred for 1 h and then diluted with Et_2O (2 mL) and then quenched by sequential dropwise addition of H_2O (76 μL), aq. NaOH (76 μL) and H_2O (228 μL). $MgSO_4$ was added and the resulting suspension was stirred vigorously for 30 min and then filtered and concentrated *in vacuo.* Purification by column chromatography (Pentane:Et_2O, 60:40) afforded the title compound (*R*,*R*)-**6** as a colourless oil (46 mg, 90%, >95:5 d.r.). The spectral data matched that previously reported in the literature.[17]

IR (film) ν_{max}/cm^{-1} 3334 (br), 2919, 2853, 1447, 1094, 1047, 1016, 993.

^{1}H NMR ($CDCl_3$, 400 MHz) δ = 3.64 (1H, dd, *J*=10.8, 2.7 Hz), 3.45 (1H, dd, *J*=10.7, 5.8 Hz), 1.80 – 1.56 (4H, m), 1.26 – 0.88 (7H, m), 0.86 (3H, d, *J*=6.4 Hz).

^{13}C NMR ($CDCl_3$, 101 MHz) δ = 66.0, 46.6, 35.7, 33.6, 29.6, 26.4, 26.2, 20.1.

$[\alpha]_D^{23}$ –41.3 (c = 1.00, MeOH) [Lit. $[\alpha]_D^{23}$ for (*S*,*S*)-**6** = +41.7 (c = 1.00, MeOH)].[17]

(d) Attempted formation of a 7-membered ring

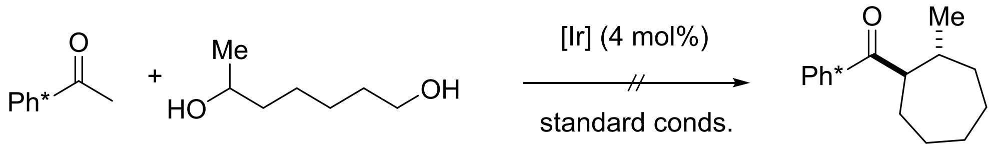

1 **2a**

Heptane-1,6-diol[18] (79 mg, 0.60 mmol), pentamethylacetophenone **1** (57 mg, 0.30 mmol), (*R*)-DTBM-SEGPHOS (18 mg, 0.015 mmol), Ir(cod)acac (4.8 mg, 0.012 mmol), *tert*-butanol (0.1 mL) and KO^{t}Bu (135 mg, 1.20 mmol) were subjected to **General Procedure A.** The desired 7-ring product could not be observed by ^{1}H-NMR or isolated by column chromatography.

5. *Computational Modelling*

5.1 Computational Methods

All calculations were performed in ORCA v. 4.1.1.[19] Geometry optimisations were performed with the hybrid PBE0[20] functional, D3BJ[21] dispersion correction, the def2-TZVP basis set on Ir (which includes the ECP60MWB effective core potential)[22] and def2-SVP[23] on all other atoms. The resolution of identity (RI) technique for Coulomb type terms (J) and the chain of spheres (COS) algorithm for exchange, RIJCOSX, was used to accelerate the calculations (default fitting basis)[24]. Vibrational frequencies were calculated at the same level of theory to assign stationary points as either minima or transition structures, the latter characterised by the presence of a large imaginary (negative) frequency, and to calculate the zero-point vibrational energy and thermal corrections at 383 K. Solvent effects were accounted for with the SMD implicit solvent model using parameters appropriate for toluene at the same level of theory.[25] Single point energies were calculated using the same PBE0-D3BJ functional and the larger def2-TZVPP[23] basis set on all atoms.

5.2 Binding mode and conformational analysis

(a) Model [Ir(MVK)] Complex

To assess the possible binding modes of enone **4** to [Ir] (where [Ir] = [IrH(*R*-BINAP)]), first a model system was constructed in which **4** was reduced to methyl vinyl ketone (MVK) and BINAP to $H_2P(CH_2)_4PH_2$. While this system removes the steric demand of the ligand, which is key to induce stereoselectivity, it allowed us to systematically and accurately explore the energetics of the possible binding modes (*vide infra* for discussion of computational methodology). For the model complex ($[IrH(H_2P(CH_2)_4PH_2)(MVK)]$, **MS**) five different isomers were located (Figure S1). The lowest energy

structure corresponds to η^4 coordination of the enone forming a pseudo-octahedral geometry (**MS-anti- η^4**).

Furthermore, in the presence of diol and base (KO^{t}Bu) there is also the possibility of forming 18 electron, coordinatively saturated complexes (**MS-EtOH-η^2**, **MS-EtO$^-$$\eta^2$**). In this model the diol was reduced to ethanol and both [IrH($H_2P(CH_2)_4PH_2$)(MVK)(ethanol)] and [IrH($H_2P(CH_2)_4PH_2$)(MVK)(ethoxide)] complexes calculated to be disfavoured over the lowest energy **MS** isomer (Figure S1). In all cases the bidentate phosphine ligand is assumed to remain coordinated.

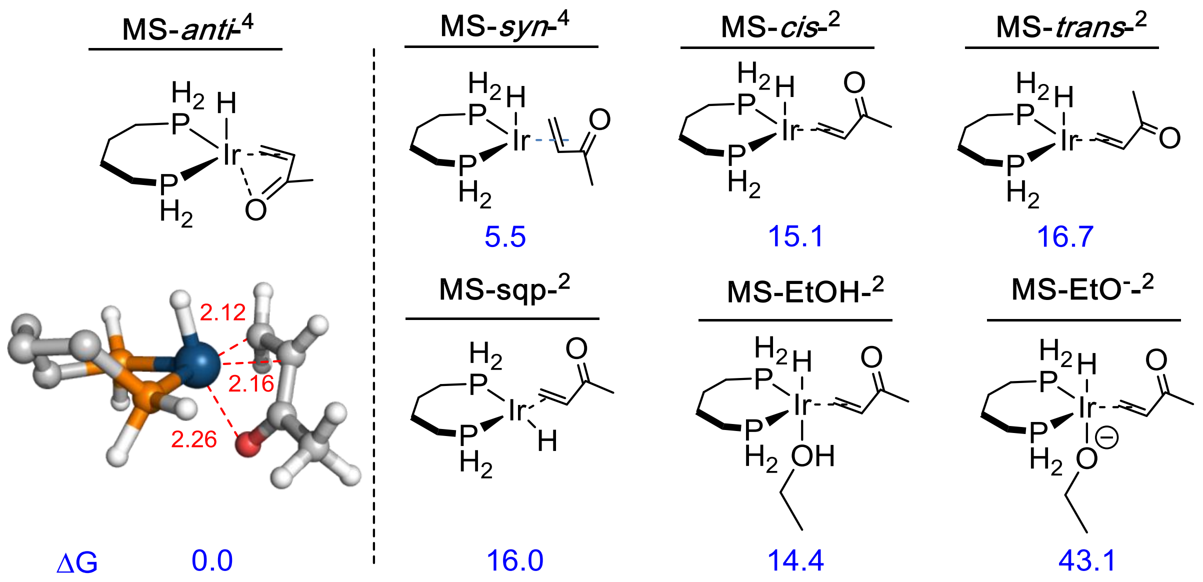

Figure S1. Relative free energy (Δ*G*) for the different binding modes of methyl vinyl ketone (MVK) to [IrH($H_2P(CH_2)_4PH_2$)(MVK)] (**MS**); **MS-EtOH** and **MS-EtO$^-$**. Δ*G* for MS-EtO$^-$-η^2 is calculated by balancing against free KOEt in solution. Relative free energies (in kcal mol^{-1}) were calculated at DLPNO-CCSD(T)/ma-def2-TZVPP, def2-TZVP(Ir)//PBE0-D3BJ/def2-SVP,def2-TZVP(Ir) level of theory. Solvent and thermodynamic corrections were calculated at the PBE0-D3BJ/def2-SVP,def2-TZVP(Ir) level using the SMD solvent model and at 383 K respectively.

(b) Conformation of enone (4)

The two rotatable bonds in enone **4**, (O=)C—C(Ph*) and (C=)C–C(=O), give rise to several different conformers, all of them very close in energy. In all cases, the *S-cis* and *S-trans* configurations were

found to be almost isoenergetic, suggesting that in principle both orientations are populated in solution (Figure S2). However, as discussed below, the *S-trans* conformer was disregarded as it leads to prohibitive steric clashes between Ph* and [Ir] complex.

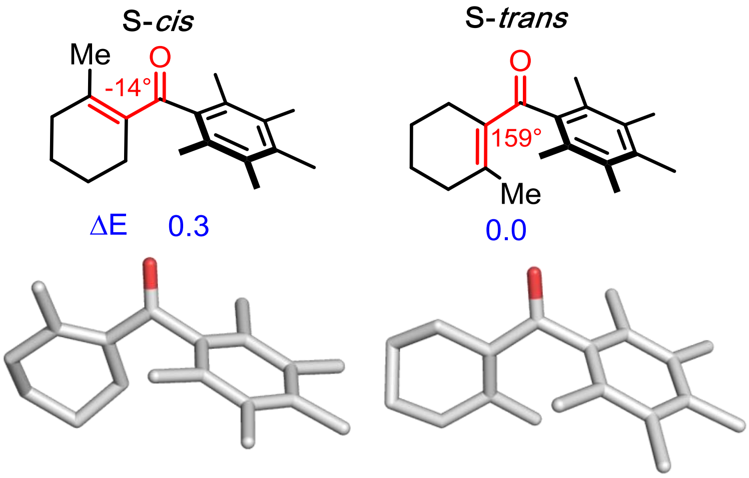

Figure S2. Geometries for the *S-cis* and *S-trans* configurations of enone **4** calculated at the PBE0-D3BJ/def2-TZVPP//PBE0-D3BJ/def2-SVP level of theory.

(c) IrH(R-BINAP) Complex

As a starting point, the coordinatively unsaturated [Ir] complex was generated by deleting the appropriate ligands from the reported complex $[IrH(Cl)_2(CO)(R\text{-BINAP})]$.[26] As graphically shown in Figure S3, two phenyl groups of the BINAP ligand are ‘pinned back’ by the *R*-binaphthyl unit, such that the other two phenyl moieties are free to rotate. This chiral [Ir] environment resembles similar models reported in the literature.[27] From this model, a clear preference for *Si*-face coordination is observed ($\Delta E_{Re/Si}$ = 8.3 kcal mol^{-1} at the PBE0-D3BJ/def2-SVP,def2-TZVP(Ir) level of theory), which arises from the steric clash between the enone methyl group and the (P)Ph moiety in the top left quadrant (Figure S3).

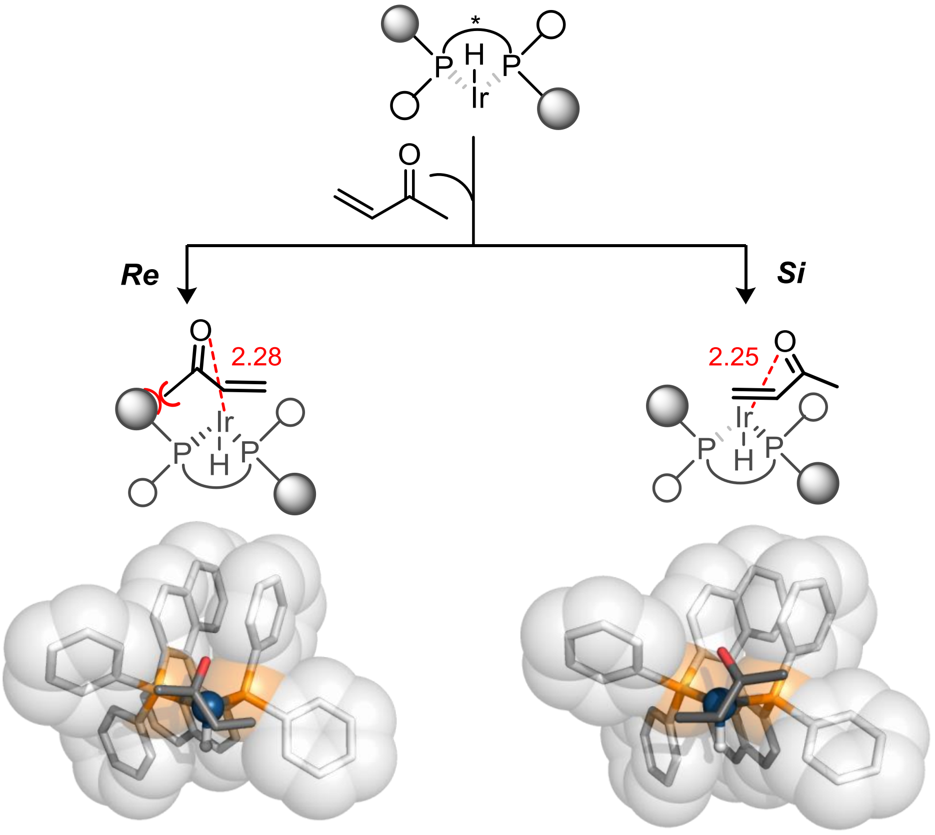

Figure S3. Stereochemical model of [IrH(*R*-BINAP)] and [IrH(*R*-BINAP)(MVK)] complexes where methyl vinyl ketone (MVK) is coordinated from the pseudo *Re*- and *Si*- faces to the chiral iridium fragment. Geometries were optimised at the PBE0-D3BJ/def2-SVP,def2-TZVP(Ir) level of theory.

Building on the knowledge gained from this model complex, the full [IrH(*R*-BINAP)(**4**)] complex was studied. For this system, three different conformers were characterised for each *Si* and *Re* face coordination. In all cases, the C(=O)–C_α(Ph*) dihedral is ~120° as observed for the isolated **4**. Moreover, coordination of the *S-trans* conformer of **4** was disregarded as it is leads to prohibitive steric clashes between Ph* and [Ir] complex. The lowest energy species (*Si/Re*-INT0, Figure S4) are those in which the carbonyl is coordinated to the vacant site *trans* to the hydride ligand. Following the trend observed in the model system, *Si*-coordination is favoured, although the difference in energy between *Si/Re* coordination is less marked ($\Delta E_{Re/Si}$ = 1.2 kcal mol^{-1}). This preference for *Si*-coordination is once again due to the steric clash between the Ph* group of **4** and the forward projecting phenyl groups of *R*-BINAP.

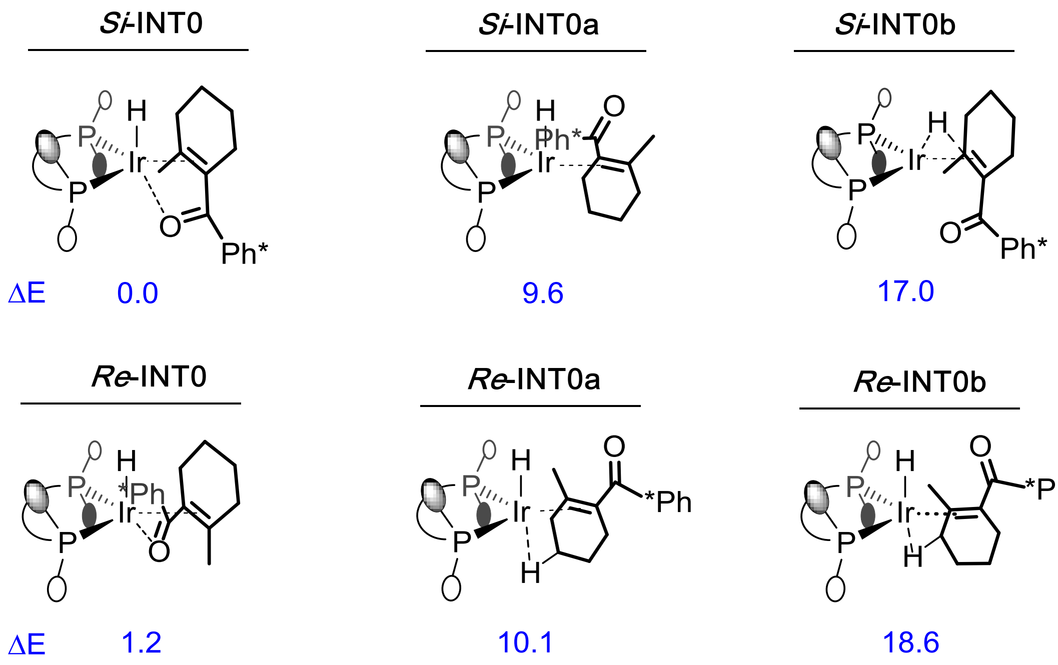

Figure S4. Isomers of [IrH(*R*-BINAP)(**4**)]. Relative energies (kcal mol^{-1}) are calculated at the PBE0-D3BJ/def2-TZVPP//PBE0-D3BJ/def2-SVP,def2-TZVP(Ir) level of theory.

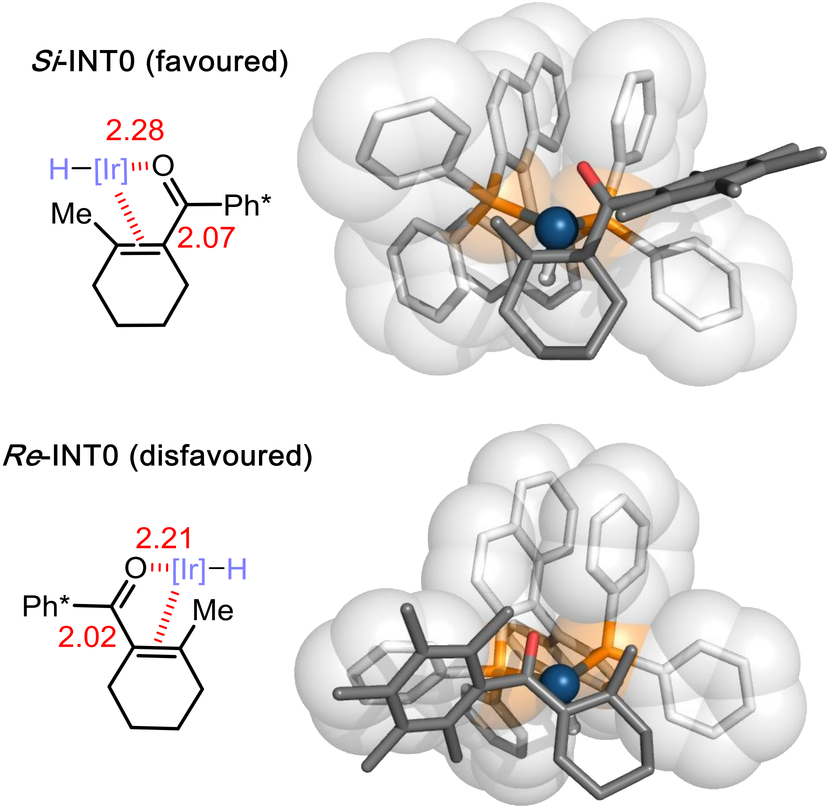

Figure S5. Intermediate geometries of [IrH(*R*-BINAP)**4**] optimised at the PBE0-D3BJ/def2-SVP,def2-TZVP(Ir) level of theory.

5.3 Origin of selectivity

Transition states for the *Si*- and *Re*- 1,3-insertion pathways were confirmed as such by visualization of the transition vector (with a single negative frequency -444.32 cm^{-1} and -456.58 cm^{-1}, respectively, Figure S6). In both TSs dissociation of O(carbonyl) is required to facilitate the pseudo square-planar geometry observed in the TS. As expected from previous analyses, the *Si*-TS is preferred by 0.8 kcal mol^{-1} (Table S1). For this pathway, a flip from the half-chair to full chair is observed for the cyclohexane motif.

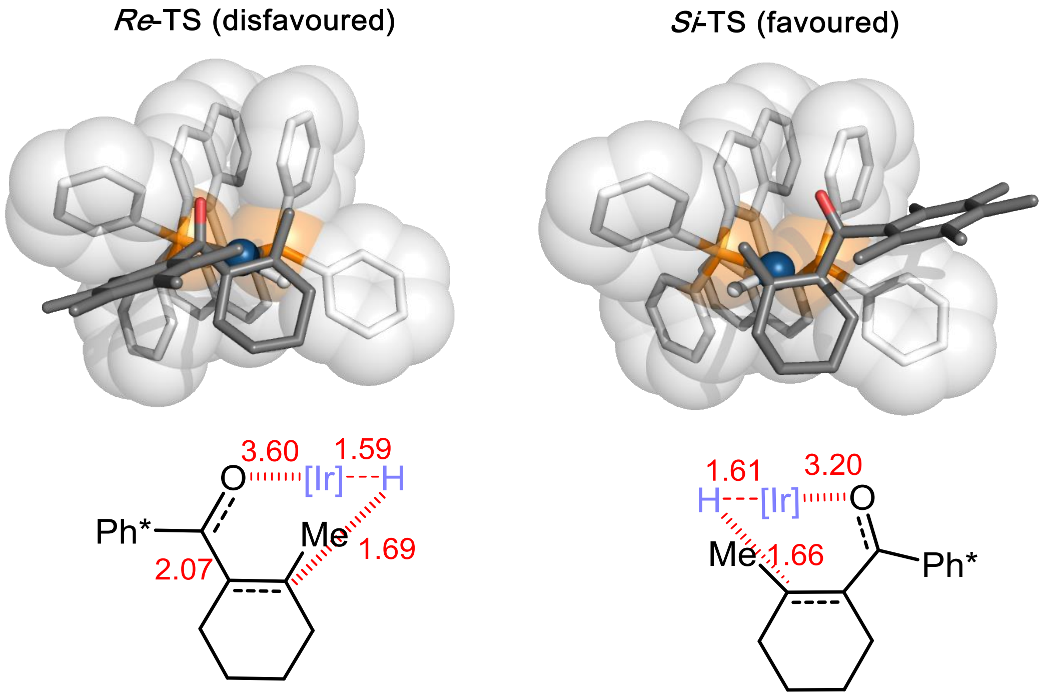

Figure S6. Transition state geometries of [IrH(*R*-BINAP)**4**] optimised at the PBE0-D3BJ/def2-SVP,def2-TZVP(Ir) level of theory. Key bond distance (in Å) are presented.

Table S1. Energy differences calculated at PBE0-D3BJ/def2-ZVPP//PBE0-D3BJ/def2-SVP,def2-TZVP(Ir) for [IrH(R-BINAP)**4**]. Solvent and thermodynamic corrections calculated using SMD with parameters appropriate for toluene and at 383 K respectively at the PBE0-D3BJ/def2-SVP,def2-TZVP(Ir) level of theory.

Species	Δ*E* / kcal mol^{-1}	Δ*H* / kcal mol^{-1}	Δ*G* / kcal mol^{-1}
***Si*-INT0**	0.0	0.0	0.0
***Si*-TS**	21.3	19.1	19.7
***Si*-INT1**	1.1	2.7	1.6
***Re*-INT0**	3.2	2.4	4.8
***Re*-TS**	23.9	22.3	20.5
***Re*-INT1**	1.2	1.4	3.7

5.4 Methodology Validation

To validate our computational methodology, single point energy calculations with different basis sets were carried out for the species outlined in Figure S1 and compared to those obtained at the DLPNO-CCSD(T)/ma-def2-TZVP level of theory (Table S2). Overall, a similar trend is observed, with the major error seen with the double-ζ def2-SVP basis. This is associated to the basis set superposition error (BSSE) which leads to overestimation of the complexation energies (Table S2, Column 1). Convergence is already achieved with def-TZVPP basis which compares well with the larger augmented ma-def2-TZVPP analogue. These results demonstrate that single point methodology used throughout (PBE0-D3BJ/def2-TZVPP) is accurate *cf*. coupled cluster energies (Mean Absolute Deviation (MAD) = 1.2 kcal mol^{-1} for all species in Table S2). Geometries were optimised at the PBE0-D3BJ/def2-SVP, def2-TZVP(Ir) level of theory.

Table S2. Absolute potential energy deviations (ΔE, in kcal mol^{-1}) to DLPNO-CCSD(T)/ ma-def2-TZVPP,def2-TZVP(Ir) reference values.

Species	PBE0-D3BJ/def2-SVP	PBE0-D3BJ/def2-TZVPP	PBE0-D3BJ/ ma-def2-TZVPP
**MS-*cis*-η^2**	1.0	1.6	1.6
**MS-*syn*-η^4**	1.0	0.1	0.1
**MS-*anti*-η^4**	0.0	0.0	0.0
**MS-sqp-η^2**	0.9	3.6	3.6
**MS-*trans*-η^2**	0.6	1.7	1.7
**MS-EtOH-η^2**	4.8	0.4	0.5
**MS-EtO$^-$-η^2**	10.3	0.7	1.8

Furthermore, we found that $\Delta E \approx \Delta G_{solv}$ for isomeric species and intermediates/TSs as shown in Table S1. This is also confirmed by the small MAD (1.4 kcal mol^{-1}) between ΔG_{solv} and ΔE for isomeric species in Figure S1. These results validate the use of potential energies as a proxy for free energies, provided required differences are > 2 kcal mol^{-1}.

5.5 Cartesian Coordinates and Energies

Cartesian coordinates and energies for species outlined in the manuscript. All energies are quoted in electronic Hartrees. Free energies were obtained directly from ORCA calculations at 383 K and do not include a 1 M standard state correction.

Species: ***Si*-INT0**
E(PBE0-D3BJ/def2-SVP, def2-TZVP(Ir)) = -3293.697191376938
E(PBE0-D3BJ/def2-SVP, def2-TZVP(Ir)/SMD(Toluene)) = -3293.746285215600
E(PBE0-D3BJ/def2-TZVPP)) = -3296.545558674753
H(PBE0-D3BJ/def2-SVP, def2-TZVP(Ir)) = -3292.55344432
G(PBE0-D3BJ/def2-SVP, def2-TZVP(Ir)) = -3292.77617900

Ir	12.04267688807625	7.08609648022819	4.58171160398627
C	13.94092098373161	6.16528126912230	4.60517266385141
C	13.57131080357588	6.36173964785486	3.17554866705174
C	13.99876192326118	4.71528602809421	5.08019076496725
C	15.15288151134900	6.91082002717961	5.13471930069994
C	13.49331147640762	5.23142543660680	2.16876740480414
C	13.78011305935782	3.85098942916764	2.76058727525499
H	14.19316572820929	5.42566729055589	1.34078392403300
H	12.49933235146755	5.23742636681981	1.69586459701916
C	13.28417301418415	3.71117977952199	4.19284429854461
H	14.87019411839870	3.66938129500792	2.75992779802825
H	13.34213006670319	3.07686770948760	2.11109211113037
H	13.48243430734598	2.69129057953223	4.56327621182058
H	12.19078721594766	3.86019598145941	4.24034992724477
H	13.63133987656818	4.66078962230489	6.11846507513592
H	15.06298117754267	4.41163855024381	5.12596313775315
C	13.55134858454474	7.72800045575705	2.71927089864557
H	15.16741406386948	7.96904071785614	4.85131392472584
H	16.07466776007539	6.43523773636951	4.74958376329865
H	15.19953824619754	6.85426575388520	6.23341739824857
O	13.36343036794980	8.65467522163506	3.57442646854044
C	13.78675929033472	8.09148643928440	1.27673405429852
C	12.77370015559157	8.66930796274640	0.49743348663259
C	13.03671963595396	9.07656555491834	-0.82682489012004
C	14.31708033750987	8.88627268585048	-1.37402789419933
C	15.32719816253656	8.28081983692431	-0.59496085694735
C	15.07432109536912	7.89062883447226	0.73172352923484
C	11.38873420068829	8.82928733043523	1.03544748371149
C	11.89775142729632	9.69180042803543	-1.59363668045752
C	14.66966104339742	9.30191922517380	-2.77871662383443
C	16.68003829432020	8.05818136547223	-1.21827262826167
C	16.18753384693376	7.29679826304693	1.55624862147425
H	11.31549558483212	8.42700447426706	2.04819187916060
H	10.65483318034456	8.29599508674915	0.41307141567565
H	11.07395147359013	9.88358564814137	1.07024287747189

H	16.57754399713020	6.36957688997797	1.10765378993658
H	15.86645271187286	7.05672588561386	2.57443113868112
H	17.03465039372734	7.99523938165871	1.63876485465415
H	16.61158060638227	7.38422474046530	-2.08848635177178
H	17.39854052994519	7.61543157682349	-0.52075689931003
H	17.11627719278707	8.99833303680954	-1.59312097664073
H	13.82483707561956	9.72342941619869	-3.33358483360106
H	15.05339594514127	8.44896086636390	-3.36084543692625
H	15.47138281851965	10.05891608002805	-2.78099235615834
H	11.42815574822420	10.50338052545980	-1.01720948164049
H	11.10334552821503	8.95088545864200	-1.78017655895776
H	12.19570277008685	10.10719382817068	-2.56180009899045
P	11.70110429637693	7.73114010919307	6.72924033678007
P	9.84089779178020	7.61011767136711	4.01199884945158
C	10.18367726123578	4.33393330432411	8.48746837043574
H	9.93449045783037	3.31871631165174	8.16989480959726
C	8.71837429712413	6.93962078541448	5.32405271732389
C	13.24474191636997	8.36216872395140	7.49012889964310
C	7.79072217301358	6.96127199920308	7.58838933696915
C	10.77986363785481	5.20877199417563	7.58173959432222
H	11.00027593074670	4.88713736972468	6.56331526917592
C	6.51332183520616	12.28620103346290	6.97548402639744
H	5.83662290741730	13.14302059520063	7.00882030009093
C	7.58910325909767	7.57531939919692	8.85359622019018
H	8.05871121469596	8.53946812449354	9.05192723057665
C	10.21841792256679	10.35235190942478	3.74851986440868
H	11.27816847549475	10.08953502975768	3.81773866960025
C	6.81862828897504	6.97563574341814	9.82200961750127
H	6.66929551768974	7.47505462004317	10.78210842425778
C	9.86211601372497	5.56330035745468	2.13559877098407
H	10.67980060231510	5.20047868429600	2.76320826739561
C	11.05992613549997	6.52541559488821	7.95322127718308
C	10.14589355814748	11.52107471529756	7.29453758188887
H	10.54288333511407	12.51881988901829	7.49915456871489
C	10.50966240667457	9.13378328830325	6.97866846133439
C	8.19461957273119	7.23718595162272	1.67154064178194
H	7.69640773268543	8.17704051913106	1.90560137604786
C	9.25700511475414	9.33823248226931	3.81361293251361
C	9.14045940399288	8.93392906732767	6.80660843897206
C	8.56107069566586	7.58343349792914	6.54940448247700
C	9.82544092016238	11.68031554381967	3.58910413426558
H	10.58360850963938	12.46521748956927	3.54192752090207
C	6.42019848900922	5.08175712618480	8.38397907776324
H	5.97065864846768	4.10460765971067	8.19117287207974
C	10.99070266353599	10.44258621083567	7.24394090270360
H	12.05446952428520	10.59714094910489	7.42076041331353
C	15.01037969872539	8.31108322230981	9.15055029375285
H	15.43666110710166	7.87326485658643	10.05608309677195
C	7.89958582056090	9.67874921675915	3.74150959142030
H	7.13767766842179	8.90446632830529	3.85501027755262
C	9.21888696923051	6.75531957696975	2.49366937517282

C	8.75428910922355	11.36043932478024	7.10435470924379
C	8.24081279917420	10.04734694633149	6.88351921418350
C	8.12813052341180	5.66485171021679	5.12438587040074
H	8.25983004790363	5.16351366528562	4.16430839368024
C	6.83727250770079	9.89957635349787	6.72036550167289
H	6.42497635788998	8.90573598765335	6.54418061556967
C	15.67177922082961	9.34741826400896	8.49513297907915
H	16.60820523496805	9.74219987579275	8.89585846029329
C	6.22706316217901	5.71516835632282	9.58898976916568
H	5.61990807048416	5.24679156710446	10.36717087795231
C	7.19504695373142	5.68439550352157	7.36065367017132
C	13.80149695051648	7.82284497501860	8.65467141948949
H	13.29289984593128	7.01213278380027	9.17892854685158
C	10.75688628891509	6.94816702268672	9.25505668215029
H	10.94946757755651	7.98288303229594	9.55071575630327
C	10.18099030030652	6.06714234669945	10.16451858983386
H	9.93222318592594	6.41363826362254	11.17011373995119
C	8.45889086150250	5.35126421971372	0.18198952966590
H	8.16164824525913	4.80838809303947	-0.71825774087866
C	8.47392118607588	12.00756439119668	3.49140811577902
H	8.16580634826342	13.04816782715840	3.36397745220194
C	9.88259572652157	4.75999731794919	9.77909074257693
H	9.40098803582685	4.07890233481840	10.48415986322698
C	7.51024798554776	11.00402272475593	3.56911492558359
H	6.44892334855464	11.25878860099434	3.52047242879530
C	7.39174608469216	5.05538501104769	6.10743381142752
H	6.93520146033060	4.07898587292767	5.92676430464515
C	15.13679318079605	9.87195592372062	7.31831323043393
H	15.66623640837523	10.66041916997884	6.77842559854460
C	7.81772951743601	6.53983112129707	0.52501904642992
H	7.02073714627764	6.93635024739885	-0.10883073688050
C	7.86596325001610	12.46487541179676	7.13895123071946
H	8.28247159252244	13.46117030496436	7.30798511068867
C	5.99767821200050	10.98690973566864	6.77098141257997
H	4.92166342012632	10.84351552543174	6.64245969089698
C	13.94614176136305	9.36763943023218	6.80450368892668
H	13.56955088856080	9.73545194304817	5.84715648056600
C	9.48650021670498	4.86590348518417	0.98988910255708
H	10.00720496549047	3.94417197306752	0.71933671749870
H	11.35209367607276	5.73438020385400	4.94372652840984

Species: ***Re*-INT0***

E(PBE0-D3BJ/def2-SVP, def2-TZVP(Ir)) = --3293.6983864373
E(PBE0-D3BJ/def2-SVP, def2-TZVP(Ir)/SMD(Toluene)) = -3293.744208253680
E(PBE0-D3BJ/def2-TZVPP)) = -3296.543719886039
H(PBE0-D3BJ/def2-SVP, def2-TZVP(Ir)) = -3292.55593715
G(PBE0-D3BJ/def2-SVP, def2-TZVP(Ir)) = -3292.77489850

Ir	12.34056025248887	7.85607659042135	5.06148577479799
C	13.45470108151327	8.27066747577261	3.21286435420351

* Contained one imaginary mode with frequency -39.99 cm^{-1}

C	14.27319382902976	8.20812017643702	4.46491909590014
C	13.43046730194480	9.59154757012561	2.43208855212786
C	15.49718139279714	7.33012890570616	4.54262392842481
C	14.40626895947426	9.56977659440306	5.13966780997917
C	14.80527877928481	10.70318023499385	4.21024501863313
H	15.05493889343510	9.47392446831280	6.02342789699854
H	13.40448267160767	9.88015238964335	5.55125022409031
C	13.68041794760097	10.87752047095304	3.20919889793406
H	15.74769616056672	10.45006712980081	3.69298569767522
H	14.98352865269724	11.63124616356415	4.77819885041412
H	13.89958991858159	11.68709595227145	2.49516064764973
H	12.77398813349221	11.18697871796445	3.75989553080160
H	12.49655156995186	9.68839831290937	1.85932043403560
H	14.22009748270996	9.52106237272513	1.66211645357959
C	13.49911124153657	7.08068028843316	2.31120048396763
H	15.88242847281739	7.25901868354339	5.56934319849466
H	16.31598724850311	7.74833246807752	3.92903159205181
H	15.27966808837765	6.31644485693024	4.18387780592093
O	13.50966155003300	5.92414121799845	2.70643874843643
C	13.60454162100754	7.31098264336231	0.80709186891711
C	14.85459523787289	7.13065032542638	0.18556888748568
C	14.96696705032091	7.32574081356820	-1.20267026097340
C	13.82852470571946	7.63256088496948	-1.96916190638541
C	12.58880382834569	7.83451109480447	-1.33953398688268
C	12.47755928888419	7.67722039762103	0.05451611972863
C	16.06990437951239	6.74990860920500	0.98828995262324
C	16.29973548005826	7.19958248277799	-1.89168849837470
C	13.95504470234530	7.73196336831229	-3.46531696345530
C	11.37698091395778	8.21206705888800	-2.15035853024028
C	11.15154492602235	7.89327942074860	0.72493490892362
H	16.65062093561706	5.96222482716965	0.48754329254379
H	16.74671808912817	7.60955274789861	1.13244509022594
H	15.79541291890630	6.36798399425792	1.97507768678995
H	10.75629145403108	8.90407048492490	0.53086552059587
H	11.22585890478448	7.77337100116457	1.81031889473056
H	10.39127433646047	7.17886932756957	0.37382904309064
H	11.63020939716391	8.89069086531265	-2.97617439513501
H	10.61678946560825	8.71271591610059	-1.53874846851424
H	10.89293056173086	7.32499641872809	-2.59648842201149
H	14.65352251373987	6.98104703232493	-3.85991476039364
H	14.33716876373640	8.71768655117883	-3.78597581666432
H	12.99495794400422	7.57464017704404	-3.97153814836774
H	17.13635673207192	7.22236089994626	-1.18368803142815
H	16.46133200859265	8.01679135001759	-2.61115696822324
H	16.38053519368175	6.25778056371099	-2.46280522080893
P	12.08386783724675	7.68179444161308	7.32562497263880
P	10.00832759825528	7.83537625115999	4.78826271403308
C	10.74485017952944	3.86408132827956	8.01081084172792
H	10.61335130209160	2.95762618006564	7.41536830788357
C	8.94309330737421	7.19821643201912	6.17411345185143
C	13.53592058639259	7.95565007070568	8.42052674557895

C	8.26203694810548	7.17717480405896	8.52728159395127
C	11.20739016974670	5.02971480718523	7.40037372897871
H	11.44259562255739	5.04416464465585	6.33377593281107
C	7.42404304084032	12.71744003600200	7.93833022820532
H	6.85017362602940	13.64514222541530	7.99399878686866
C	8.28194275181958	7.73356540836086	9.83349182249987
H	8.80229621162139	8.67879995361507	9.99588389666335
C	9.96845653520127	10.31710630622852	3.56522497967321
H	10.98697569396009	10.04165565496789	3.28660887237776
C	7.67304794268108	7.09012023888107	10.88487007847293
H	7.70405325851259	7.53318555865673	11.88323729034255
C	10.19728189480104	5.64007707792407	3.05751880450836
H	11.15016160186425	5.45561425183451	3.55822246736171
C	11.37760896681490	6.19812205481856	8.14719214384722
C	10.97008994483599	11.57038227963446	7.81120470078752
H	11.49035084564306	12.53107537889740	7.84174943571216
C	11.02642649944936	9.13942423574166	7.70521925321076
C	8.25497379782086	7.02755762584907	2.69965068282078
H	7.65558488251242	7.90509794768501	2.94933520543127
C	9.22303406628662	9.43754027764556	4.35799261490482
C	9.63919781149111	9.09219911511272	7.65967964733834
C	8.93262510409537	7.80192401674865	7.43109688214517
C	9.41444803137212	11.51619875930961	3.12494330056672
H	10.00565584271971	12.18960660364192	2.49911237068508
C	6.97258347982303	5.29410909786342	9.43221901828542
H	6.46687028094205	4.34001001943528	9.26579029405059
C	11.68218368523907	10.40087097410530	7.77036696597137
H	12.77355251305705	10.43455456027993	7.77418615007268
C	15.89087126863499	7.51753417664157	8.80087958870328
H	16.82673556656036	7.06384962912597	8.46591302528846
C	7.91805688485803	9.78682863434167	4.71947949014192
H	7.32721221153364	9.10988824739984	5.34061835208873
C	9.43274728682864	6.75963676005548	3.40733663446075
C	9.55372284138952	11.55580489642493	7.82975630456760
C	8.87744891418876	10.29989699522000	7.78051641237326
C	8.23761798033369	5.97903378843950	5.99475832505986
H	8.22389451864474	5.51032165823348	5.01087298573690
C	7.45897181967373	10.29854916280586	7.84491715315294
H	6.92991346538087	9.34485199416651	7.83331846719482
C	15.84098781289358	8.24820735574819	9.98726341220928
H	16.74174307362898	8.38402602523569	10.59067912767489
C	7.00932507273286	5.85883202038966	10.68473684928516
H	6.52696733297338	5.35811446936312	11.52702942892857
C	7.59461940549356	5.93229405699889	8.32964105950466
C	14.74323332327908	7.37497626123278	8.02467728275682
H	14.77273654994789	6.82166832933017	7.08488999837648
C	11.09457938050149	6.18108003966965	9.51913839976582
H	11.23151013687466	7.08168738692205	10.11988962145654
C	10.62295345714547	5.02162057762155	10.12404355089047
H	10.38021457816445	5.02627563953495	11.18870561096781
C	8.60378995256572	5.06419285854252	1.33336751426405

H	8.28474046172597	4.40733680136839	0.52037170008233
C	8.11407788965639	11.86017735444460	3.49398336561062
H	7.67842320369025	12.80303146489181	3.15482949836055
C	10.44615048346109	3.86016095365483	9.37127953766728
H	10.07164178202811	2.95250258365809	9.85100099736782
C	7.37073189866733	10.99734667924305	4.29767061527483
H	6.35947489665662	11.27170205167562	4.60557017474560
C	7.58850420861964	5.36492787722137	7.03514560955069
H	7.06513451031819	4.41976306158814	6.87348425133725
C	14.63440114634837	8.81031005812766	10.40411218211748
H	14.58618265038620	9.37439401822345	11.33848933315772
C	7.84510515964186	6.18633240169630	1.66766680834852
H	6.93000233192719	6.41553498603970	1.11667364448956
C	8.79761708392674	12.75297977486981	7.89473985974189
H	9.33116637545945	13.70667921871550	7.91776230037092
C	6.75241115204445	11.47545975132164	7.92678375812849
H	5.66140095855931	11.44924685452530	7.98373209544327
C	13.48522329719905	8.66094941961049	9.63033573478909
H	12.55053382758985	9.11613203121101	9.96749872848498
C	9.78113467443195	4.79527840778462	2.02971250289725
H	10.40029654865092	3.93882503046225	1.75514444503882
H	12.43395978230176	6.32596933980460	4.91961819597420

Species: ***Si*-TS***
E(PBE0-D3BJ/def2-SVP, def2-TZVP(Ir)) = -3293.658416301381
E(PBE0-D3BJ/def2-SVP, def2-TZVP(Ir)/SMD(Toluene)) = -3293.708233723551
E(PBE0-D3BJ/def2-TZVPP)) = -3296.510928811212
H(PBE0-D3BJ/def2-SVP, def2-TZVP(Ir)) = -3292.51815037
G(PBE0-D3BJ/def2-SVP, def2-TZVP(Ir)) = -3292.73997767
Imaginary Frequency = -444.32 cm^{-1}

Ir	12.21923990255928	7.01897786623393	4.97111249574733
C	14.27960699729122	6.55412591791171	4.34860582349440
C	13.46816855167705	6.81669465583770	3.14848117409254
C	14.77585950729482	5.12118870378909	4.49250130230399
C	15.35158627082327	7.52344505844310	4.80973177485532
C	13.21086208232773	5.68617088437844	2.14818265102378
C	13.39352880011865	4.23670017217798	2.63480795842335
H	13.87628889520091	5.83667912721918	1.28610867897647
H	12.20334884444000	5.81919914676189	1.73012222298762
C	13.72647117111606	4.10217756387032	4.11260051087957
H	14.21450638164549	3.77438124926631	2.06294618948409
H	12.49976540558582	3.64057971363662	2.38969950420501
H	14.07975926354438	3.08190950457913	4.33572630169539
H	12.83130713362901	4.29071280192344	4.73457877847109
H	15.13678264942275	4.96481847549932	5.52176374577158
H	15.65937109462259	5.01282351348312	3.83362208612332
C	13.62070369904200	8.15798285611081	2.51082366263182
H	15.04161619459912	8.56847468781696	4.72959978186901
H	16.25134303922635	7.38286547444892	4.18584505923171

* Contained a second imaginary mode with frequency -24.40 cm^{-1}

H	15.63522642772066	7.31019946994001	5.85019428486906
O	13.67616661090496	9.20582064333404	3.13626582281356
C	13.75936185331379	8.26272634610148	0.99808597108880
C	12.70381913162398	8.81498479456497	0.26297094796793
C	12.86748359964196	9.10494415995610	-1.10641476918806
C	14.08877614462222	8.81253840820133	-1.73797639717842
C	15.13046488079901	8.20605781402273	-1.00257335810214
C	14.97138308838878	7.92802538069639	0.36562183493509
C	11.37853311157888	9.07229019521794	0.91267823313377
C	11.69457356360085	9.71162341902790	-1.82887135606614
C	14.34395381042906	9.11566943640300	-3.19050293758825
C	16.41513874269938	7.87446438527457	-1.71384129985584
C	16.09552637605694	7.29751903744009	1.14584943214983
H	11.33643825226521	8.64829863365122	1.91939510052118
H	10.56578315297628	8.61470387880385	0.33209324571429
H	11.15312299062041	10.14741753776437	1.00124057459403
H	16.50071012422796	6.41389616202439	0.63164227040587
H	15.76910131945747	6.97079137481813	2.13886597966811
H	16.93011366540177	8.00203202005767	1.29254567158994
H	16.24449241917170	7.16391036945385	-2.53979452825730
H	17.16261922714236	7.42925395071024	-1.04858348988770
H	16.86857829640085	8.77064028819063	-2.16853623396537
H	13.49029195456442	9.57994770515658	-3.69542227259299
H	14.59978389705763	8.20283459630803	-3.75269668836269
H	15.20172620317850	9.79755539075686	-3.30957648358950
H	11.26254880790700	10.54444027266413	-1.25434499155796
H	10.88588901533955	8.97296629496836	-1.95951422390766
H	11.94778374819209	10.09813955047395	-2.82195584117015
P	11.65639365279667	7.55523879368145	7.07209660652609
P	10.05978299633834	7.43385928120739	4.27377802645172
C	9.78452465433729	4.19621826743440	8.46204598894245
H	9.52223019891242	3.21508901008643	8.05855582490355
C	8.77580820623849	6.85238417843673	5.49133514565462
C	13.15009672557854	8.09903850798863	7.99822478525742
C	7.60938733900425	7.02098752461407	7.63812028937027
C	10.54983305525620	5.07523877963995	7.69919141425160
H	10.90320587904364	4.80791620956916	6.69920321300204
C	6.70747180979787	12.36910729306392	6.81981482961699
H	6.07792163695377	13.26067966482396	6.77515968445142
C	7.33901452688879	7.68052945891666	8.86738025569264
H	7.86940220075616	8.60541335065249	9.09730663985805
C	10.46524859296326	10.17145789777483	3.94736374829882
H	11.51839828583616	9.91590383367346	4.10607492404616
C	6.42921643236458	7.17191295101993	9.76402032260132
H	6.23240203819612	7.70410736720125	10.69739348602023
C	10.02181672156966	5.20926372853335	2.61588068611399
H	10.65866325665407	4.79179406029774	3.40261230896789
C	10.83887791820331	6.35379167477650	8.18521546155942
C	10.25893392090971	11.42578692600325	7.43115913487623
H	10.69228953377051	12.40828211196725	7.63548509416640
C	10.51657245208718	9.01442932629872	7.21648755451295

C	8.78615438401887	7.07482653260474	1.72272819455804
H	8.43248433049799	8.10390280379877	1.79185993740004
C	9.50680126269890	9.15378074649212	3.96220322538732
C	9.15620441296633	8.87759687648935	6.94632280275349
C	8.52843179502186	7.55127660354622	6.67241977560955
C	10.07401752633617	11.48922665448383	3.71315646031237
H	10.82675660909769	12.28050789966675	3.70524566241409
C	6.00685309322527	5.28538865836511	8.32329678224743
H	5.49602969223864	4.34490019026761	8.10280128453154
C	11.04601252364163	10.30448998270357	7.47735583866134
H	12.09988846121472	10.41033734182823	7.73014971788779
C	14.71325459410815	8.00129505134787	9.84720838001510
H	15.01385322236349	7.56583129965575	10.80305197930274
C	8.15884544410046	9.47071109358701	3.74984710330682
H	7.40085850365435	8.68471853319729	3.80296822196216
C	9.56307298583120	6.52697767304326	2.74906581240417
C	8.87864687624178	11.33002094765968	7.14045185540095
C	8.31284002122737	10.03853657638078	6.91940835611380
C	8.09056094160902	5.63370960330454	5.25238617468783
H	8.26669965501697	5.09490554238560	4.32178181560431
C	6.91850384820297	9.96157968754959	6.65470620116632
H	6.46357851727683	8.98791949100855	6.47298108654771
C	15.49998315814554	8.98330160166643	9.24978492503104
H	16.40861769552476	9.33682378024856	9.74276075829468
C	5.75342261617597	5.96143112810605	9.49281751852937
H	5.03549750344599	5.56496282381289	10.21480378638983
C	6.93045531301064	5.79407743212504	7.37381341776869
C	13.53904918692906	7.56584066788450	9.23069880511506
H	12.93074081342754	6.79750731677344	9.71181688338008
C	10.38877881183155	6.72837735132897	9.45662391874922
H	10.59306522512917	7.73517448566926	9.83100751917129
C	9.64425199562691	5.83976363331735	10.22676041096749
H	9.27744973856116	6.14738332422041	11.20829021155416
C	8.91528607879418	5.00577000303822	0.47808649503079
H	8.66097743376984	4.41399678603748	-0.40425658465782
C	8.73367263623739	11.79762275202178	3.48727146995859
H	8.42850122100253	12.83040888264018	3.30184907679810
C	9.32794839311261	4.57810761326243	9.72323769814489
H	8.71333988849858	3.89400004271801	10.31337380019751
C	7.77467380999297	10.78533798973032	3.50338826256355
H	6.72091246737376	11.02602480638262	3.34353111739912
C	7.19413551338768	5.12337062066164	6.15717417853225
H	6.66812027524022	4.19001369901520	5.94050774102096
C	15.12759673368287	9.50765729535550	8.01155117916881
H	15.75156473505873	10.25912437972678	7.52169796584274
C	8.47102931230188	6.32143370451271	0.59276981445520
H	7.87302429282415	6.77020360267866	-0.20413739287642
C	8.05259235000298	12.48080770154808	7.07690196554836
H	8.51097696376499	13.45851463757221	7.24581489128120
C	6.13867147126462	11.09287037937082	6.61437718686618
H	5.06972974902447	11.00281190959720	6.40572063654655

C	13.97291715956621	9.05434724055428	7.38196263486299
H	13.70776140452074	9.42774985000519	6.38891663343905
C	9.69006451032870	4.45033919819492	1.49575550173432
H	10.05801168500818	3.42551495171456	1.40611722255422
H	13.45493082293565	6.37913853210966	5.78226305677213

Species: ***Re*-TS**
E(PBE0-D3BJ/def2-SVP, def2-TZVP(Ir)) = -3293.651508335040
E(PBE0-D3BJ/def2-SVP, def2-TZVP(Ir)/SMD(Toluene)) = -3293.702722770602
E(PBE0-D3BJ/def2-TZVPP)) = -3296.505396811773
H(PBE0-D3BJ/def2-SVP, def2-TZVP(Ir)) = -3292.51027872
G(PBE0-D3BJ/def2-SVP, def2-TZVP(Ir)) = -3292.73586789
Imaginary Frequency = -415.16 cm^{-1}

H	11.83114408445364	6.74497967070873	2.90149221080657
H	9.68346102032854	3.78442697568639	0.97037789606168
C	9.23146664413209	4.74984666837630	1.20988491497079
H	13.75083442766604	10.04708136003874	5.65735000041325
C	14.01823669470426	9.82257945624161	6.69223491951758
H	4.78611457194902	10.38226648413929	7.09059235235110
C	5.84862953582928	10.63384316118351	7.14128510500412
H	7.90822547853103	13.32321181791450	7.53321256046510
C	7.58287410349855	12.29010191897467	7.38594402250999
H	6.87155621352077	6.92355744116299	0.08178078118640
C	7.65950956388092	6.50323047123877	0.71153708712274
H	15.65098290823045	11.21969371315848	6.70164293696261
C	15.09907010291266	10.47610310041598	7.27996272534488
H	7.27385480530239	3.89813183382114	6.22496704600252
C	7.67974974404659	4.90195627085237	6.37143163286919
H	6.09628570282706	10.98665443003047	3.85996757502137
C	7.17388025402704	10.81205909987961	3.81748671208773
H	10.89791394161287	3.35119936657620	9.65520260113181
C	11.08941356188250	4.25559344765218	9.07287800358642
H	7.64906738878753	12.89275617424659	3.50700208417855
C	8.04702451654092	11.88226822433032	3.62726851479291
H	7.85273578867436	4.69795016601255	-0.45639172048020
C	8.21139564261227	5.26229392580910	0.40757099123594
H	10.33560493670315	5.58172967771141	10.60420589354400
C	10.78075979291305	5.50543377187851	9.61008320192696
H	10.75071031606643	7.63504916104745	9.28666779040666
C	11.01164397321375	6.66025829265254	8.86834489273344
H	13.21744348196236	7.70360331679402	9.23808197723356
C	13.72768163195087	8.49705937083209	8.68989063712257
C	7.54377505699129	5.51835097876075	7.63806037288064
H	6.26057584278082	4.96296109368649	10.76202843962169
C	6.76205622527256	5.47673954224664	9.93871516020919
H	16.35360833816232	10.64485863672632	9.03158879693015
C	15.49713929465114	10.14361865020413	8.57455061577246
H	6.46122059553591	8.60783756802934	6.87231036641232
C	6.78720742203800	9.63685643285643	7.02354877190573
H	8.37240900812371	5.06330552944873	4.36653746414323

C	8.29374437378562	5.55607279399043	5.33587631202240
C	8.17773304932963	9.92411420390436	7.08419605480988
C	8.57229004324545	11.28188592158275	7.26999262061189
C	9.12819825195974	6.71861523518953	2.63332958317323
H	6.98954956544308	8.69549072361386	4.17458779575520
C	7.67595275024572	9.52394748467003	3.98331798386777
H	15.14780429953553	8.84833174020009	10.27025748261790
C	14.81584377904651	9.14584461427870	9.27254465467660
H	11.94689662442114	10.86706001931079	7.25362039679112
C	10.89201936125174	10.60100590393450	7.19256601936942
H	6.51828885453832	3.84983882780793	8.55920407611085
C	6.90590212306351	4.85888061174900	8.71959411993291
H	10.11041180814816	12.49696838296696	3.45936651042913
C	9.42254295579511	11.66032053852714	3.59833025943262
C	8.69793705168685	7.50673243389676	6.72604831210630
C	9.17601415477377	8.90410614662798	6.93172580364891
C	9.05711193372959	9.29089264967009	3.93699432451210
H	7.67837420264657	8.20194458394876	2.02396228307767
C	8.11322451155012	7.22503030215102	1.81570451308711
C	10.52671455803434	9.24642837882345	6.97202614572672
H	10.26210418449469	12.62027635405566	7.50112483368114
C	9.95123279720763	11.58599673043811	7.33343914078011
C	11.54179485075384	6.57444536497597	7.57658134506196
H	10.49520445491204	5.07497162970088	2.92964121228236
C	9.68587390004011	5.47195985428314	2.30999388643261
H	7.13399608128813	7.28126883293646	11.09004945496202
C	7.25763415199042	6.78686085201768	10.12336815313525
H	11.00094446298910	10.16996291761327	3.73072243864224
C	9.92548967241372	10.36895587451134	3.74593899521553
H	8.26281094744338	8.45856675629194	9.25165696972869
C	7.88550250792952	7.44782801378748	9.09380371096657
H	5.49250692743317	12.76137296864886	7.41623515693638
C	6.24642424867554	11.97661127534939	7.32184348497050
H	12.23803647409145	5.26763291789740	6.02253148516679
C	11.84449379191027	5.31714783911475	7.04193333470832
C	8.04984614763656	6.83980846426330	7.81889529842476
C	13.31336630685832	8.83936950940572	7.39844500190279
C	8.81542656475925	6.86591722709044	5.49530182053334
H	11.86941521617830	3.18749026849006	7.36020697186985
C	11.62521956373007	4.16247200915504	7.78942419721448
P	9.78996104623801	7.61786557677166	4.11200346000755
P	11.88307839540958	8.04994397787893	6.55205401780093
H	20.69659420904435	8.30616732007667	2.37278808675730
H	20.30380820804466	6.63727614531749	1.93836806828305
H	19.39074784662169	7.97851877345057	1.23185446990870
H	21.18356586429773	7.59523452665251	5.64538404374310
H	20.96353174898367	5.90496451316130	5.20218945009510
H	21.42687366698672	7.09376606396023	3.96727843452555
H	19.75035482404198	7.43630789139585	7.32997933657381
H	18.17261555939131	6.77692991389016	7.78518846002585
H	19.40615935027999	5.73175714333677	7.04524575876384

H	16.19222171153556	7.86258535144549	7.35663926818378
H	15.04335392590099	7.18895949108143	6.19803514654048
H	16.19603463113204	6.12456259381272	7.01516934525359
H	17.55132979556143	9.15612491059519	1.36656904168737
H	17.19555680179733	7.46225164132948	0.98850217002907
H	15.96115306675694	8.49354576172568	1.73802416867503
C	16.07723515035039	7.11849223032003	6.55503871085577
C	18.95589622592688	6.73936634772259	7.01984159591782
C	20.79818026274405	6.93755266984951	4.85074088237339
C	19.86081489762129	7.62379841175193	2.15510958991685
C	17.03129873302329	8.25780848461499	1.73376502090225
C	17.06662009600243	7.32848280122488	5.44411149392692
C	18.43516711044553	7.09216384847744	5.65417706850806
C	19.34211233874460	7.20258924389498	4.58531466566193
C	18.88652964408070	7.55258147458357	3.30149783054077
C	17.52475857252541	7.84894635540021	3.09898200034009
C	16.63132397263536	7.75234574861277	4.17924031538836
O	15.04385816370532	9.45388014278644	3.98818239229719
H	12.15164525141777	9.03024793970936	1.42739925615661
H	13.06411540455347	9.70436966423613	2.82031906907796
H	13.91496487683498	9.23815797027813	1.33737299706394
C	15.20495607601297	8.24302788872345	3.97191905636393
H	15.39219195457477	5.68876374894567	4.50557610270969
H	13.71185971965502	5.22880601669102	4.29535186715803
H	15.16148332887371	4.03546692502155	2.69157933080243
H	15.73209071858078	5.57481422669006	2.02749571490908
H	12.80938919212738	4.63537617735962	2.06342745926954
H	13.85314302509860	4.63459516141706	0.63488178835194
C	14.86684809966101	5.08173123702142	2.50548582343857
H	12.34350868191777	6.67361229177250	0.75991457561719
H	14.06369168513667	7.02443382880268	0.62609709642432
C	13.66040020913251	5.15530989844461	1.58703097344345
C	13.29852011243856	6.60274739984109	1.30493735899315
C	13.08167154534394	8.96854875007576	2.01180549208697
C	14.54284197797952	5.77581317966035	3.81801691408239
C	13.26181770910534	7.54789157627973	2.51186228498439
C	14.14866963768272	7.24888832597633	3.65570969537693
Ir	12.02797085676126	7.51087707973566	4.28388537184491

Species: ***Si*-INT1**

E(PBE0-D3BJ/def2-SVP, def2-TZVP(Ir)) = -3293.689592077024

E(PBE0-D3BJ/def2-SVP, def2-TZVP(Ir)/SMD(Toluene)) = -3293.739811574409

E(PBE0-D3BJ/def2-TZVPP)) = -3296.542602494011

H(PBE0-D3BJ/def2-SVP, def2-TZVP(Ir)) = -3292.54339894

G(PBE0-D3BJ/def2-SVP, def2-TZVP(Ir)) = -3292.76785952

Ir	14.33444812002684	6.61385001368240	4.61869285971686
C	13.57494493213898	4.20990057762794	2.79346088534406
C	12.89480098177473	5.47955115908542	3.34404414271674
C	12.76646352437458	3.68072678732360	1.59297552852504
C	15.05336380229806	4.27172304993683	2.43391756914752

C	11.45755977028891	5.17236326542711	3.71777017882140
C	10.65290159006275	4.63981202984328	2.53247461154206
H	10.95800237635478	6.05385906414725	4.13626076317454
H	11.45570989366075	4.40780769087064	4.51220648643330
C	11.31108237261790	3.40457915938578	1.93784741985795
H	10.57831595236577	5.42958208361879	1.76324710071036
H	9.62487715498611	4.40849400505672	2.85883201197262
H	10.75945315767868	3.05604291749703	1.04888001229091
H	11.26133505812225	2.58038587091170	2.67316098280883
H	13.25043975451364	2.76600776501484	1.20959396343321
H	12.82356369880875	4.42381920724155	0.77820751730949
C	13.16844648273302	6.79975469894601	2.83800190390628
H	15.66520757444803	4.55726022826754	3.30194417930599
H	15.24712767946048	5.00323884684400	1.63771090995864
H	15.38898101409156	3.28431058151867	2.07641439893636
O	14.42036954288800	7.10052484317851	2.59671224207744
C	12.15937164953037	7.85294170105484	2.45989948923294
C	11.39955409030823	8.61931752606930	3.36129400460912
C	10.57166152852674	9.65808488766160	2.87921953658782
C	10.47895108839310	9.89849155061276	1.49975167515589
C	11.22914019391377	9.11347122958741	0.60112491153736
C	12.07981744349703	8.10373912277350	1.07170173388777
C	11.45045763645889	8.39777108892057	4.84471068353801
C	9.82935180821255	10.48836909938108	3.89057517364037
C	9.59774065912843	10.96631934306783	0.91662832312333
C	11.09911142073330	9.38589616198263	-0.87055466570155
C	12.89056878306076	7.30444218878836	0.08439264716478
H	12.25170943581964	7.68682604119152	5.11402451253236
H	10.49164304322821	8.01789634219795	5.23586592432769
H	11.64624725361962	9.33608042117129	5.38626278853575
H	12.24058738361361	6.81413149721059	-0.65698368501722
H	13.49572479477128	6.53147485532951	0.56506110204064
H	13.59082010586880	7.95213686500267	-0.46714297363518
H	10.06046089195476	9.24180993108957	-1.21189916764316
H	11.73284185554149	8.73516115509418	-1.47989474080568
H	11.37076754870490	10.42584617088582	-1.11516150006517
H	9.04215826676288	11.53562074312197	1.66848811929320
H	8.85854165928042	10.53040851854162	0.22477258676392
H	10.18621328734732	11.68797322209850	0.32708639226692
H	10.53236058892623	11.02696247456005	4.54888017918169
H	9.20640861432502	9.86480594878034	4.55100214722288
H	9.17386849731148	11.23886371344794	3.43681517840779
P	16.10790560688276	7.93513681160076	5.18549390362994
P	14.18196016352550	5.81831065616855	6.67666776846056
C	18.88219306688188	6.14749542592286	2.78432959693167
H	18.86022915858420	5.58059202972199	1.85103852995450
C	15.87188911402233	5.13773763466893	6.90809239983273
C	15.92703578730243	9.58935019299731	4.40745486554486
C	18.25620993595254	5.36360199759662	7.37970635236194
C	17.69580906217402	6.64395236808210	3.31761401659274
H	16.74415035321346	6.48511272159598	2.80713561223613

C	16.84769398904053	8.24432623717703	12.02123475214401
H	16.87230660328499	8.48217689825918	13.08772986502570
C	19.35783498113299	6.03905908129834	7.96763711167856
H	19.18576225844407	6.98212216146568	8.48730843750011
C	13.42662626600548	8.21438990157871	7.92717456367625
H	13.44303901995077	8.62292986715009	6.91425949786929
C	20.63104350166643	5.52215888058746	7.89118657023556
H	21.46002984464585	6.05401632176366	8.36334869588911
C	11.66682373884169	4.78036985894758	6.99489875458119
H	11.38185953542700	5.74582395594647	6.56967875863571
C	17.71241807799142	7.35649550184325	4.52159554844240
C	16.45925050965653	9.96605729699933	8.73985530083316
H	16.36126727565743	11.00341714846489	9.07030749708311
C	16.48427973693923	8.31501233714718	6.95498862312557
C	13.38099747639779	3.24558467845979	7.71966827462616
H	14.43105637648731	2.99163852371311	7.87970627478123
C	13.79786970216486	6.88368881734084	8.12442256531668
C	16.72063350432025	7.29595271835077	7.87992294861256
C	16.92230311356094	5.89161185333667	7.41535103101899
C	13.05351963401178	9.00624370562572	9.01168838530564
H	12.76499283535573	10.04656266563820	8.84616219727047
C	19.82835102950235	3.63433495982205	6.62543096539432
H	20.00084734448404	2.69828754102479	6.08892138518894
C	16.36845348086938	9.65370461314778	7.40698680833980
H	16.18801484978958	10.44638635394356	6.68001780110593
C	16.83555207647824	11.65629975631310	3.53662815560901
H	17.69606854747581	12.29800880499308	3.33354833887023
C	13.77219697779701	6.34676576986655	9.41812210549460
H	14.03777032574254	5.29906908393434	9.57987859338182
C	13.02510099919992	4.46333306710947	7.12978847907837
C	16.64960679402949	8.95467164169735	9.70861863175500
C	16.79279239077984	7.60296089013539	9.27293170644444
C	16.13894896526100	3.90992542167254	6.23927340593419
H	15.31510787041964	3.37213488747589	5.76659119999762
C	16.96473951279607	6.59207369021377	10.25486784452886
H	17.06931639814817	5.55576287451532	9.92871677067331
C	15.55574104210522	12.06291683861439	3.15426290189531
H	15.41416018907001	13.02450003487473	2.65503578640294
C	20.87452363622189	4.31454295089773	7.20252147537640
H	21.89092668886508	3.92005860226021	7.12964699470213
C	18.50313072546544	4.12583381684394	6.71296534032582
C	17.02403461809786	10.42226578275369	4.15451343886494
H	18.03098362210869	10.09903076818889	4.43008600973148
C	18.93097355611648	7.59831180414001	5.16278985913880
H	18.95037066796105	8.15265790562372	6.10305495610252
C	20.11708624646943	7.10457811112613	4.62436870853304
H	21.06034524266400	7.28705269505962	5.14416239524585
C	11.04661223620838	2.67312208405653	7.99272015772594
H	10.27776100178384	1.97713278298600	8.33443435099669
C	13.04668514077886	8.47187962755324	10.29807029863055
H	12.75322451604631	9.09241824828214	11.14725305482664

C	20.09320780389212	6.36823193795792	3.44100106778205
H	21.01959467852802	5.96325065010176	3.02615936365571
C	13.40689732814463	7.13910447543350	10.50012957338953
H	13.40146890997442	6.71304468600742	11.50626119606432
C	17.41530102242444	3.42899030118237	6.12902546317595
H	17.61010670781299	2.50512700380304	5.57941062707665
C	14.46298611563263	11.23201752791293	3.38975503086690
H	13.46182285835019	11.52573452623339	3.06396853777236
C	12.39386136285508	2.35219786589709	8.13896231843685
H	12.68695670897464	1.40626621582441	8.60081266580556
C	16.68710602632422	9.24614820080149	11.09532276444008
H	16.57766560829805	10.28600784600767	11.41397621469280
C	16.98761599066988	6.90422879604716	11.59396593187979
H	17.11610930155789	6.11070463038700	12.33365513635892
C	14.65018669484270	10.00092093832031	4.01413708964083
H	13.80891113461874	9.32605224437618	4.17806205373061
C	10.68441720623931	3.89570858575337	7.42559216544480
H	9.63075274681686	4.16921284340478	7.32547680909399
H	13.46314090925165	3.45956542092419	3.60008332702837

Species: ***Re*-INT1***

E(PBE0-D3BJ/def2-SVP, def2-TZVP(Ir)) = -3293.687097658445
E(PBE0-D3BJ/def2-SVP, def2-TZVP(Ir)/SMD(Toluene)) = -3293.739071573045
E(PBE0-D3BJ/def2-TZVPP)) = -3296.540767942845
H(PBE0-D3BJ/def2-SVP, def2-TZVP(Ir)) = -3292.54296118
G(PBE0-D3BJ/def2-SVP, def2-TZVP(Ir)) = -3292.76217051

H	13.22312581877444	9.78479579139257	3.06025510662256
H	11.11567902373099	2.92976277541375	2.22021239284796
C	10.61057814167131	3.89032131071172	2.09316953917124
H	13.23296397161800	10.71622903094653	5.09073070820613
C	13.54861920080042	10.94331971473620	6.11155379263882
H	4.43491054311174	9.06863375956497	6.35188327715285
C	5.37370066439297	9.62407855993352	6.41516598565636
H	6.54770235158512	12.82597683215174	6.41278541504758
C	6.54139014627901	11.73301353091273	6.44625558456206
H	8.67758399657293	5.62153963194590	-0.10534172913507
C	9.24632004609649	5.39147676243618	0.79881228026793
H	14.82714103838795	12.57771944552229	5.53373258288058
C	14.44413341942867	11.97866829904446	6.36290722763662
H	8.44140188046777	3.58696009628258	6.96044034725899
C	8.62743697043438	4.66389728930382	6.93808459051781
H	5.92192629898468	9.21538173777903	3.02976766684527
C	6.98603733606794	9.42910370971307	3.15377896675984
H	12.11114720767614	5.64984399894575	11.46498209821466
C	12.02606557524922	6.24475364327005	10.55234310294270
H	6.80986903009047	11.49648850454590	2.55706534862279
C	7.48493082484722	10.70600969784003	2.89159639962197
H	9.78793270747270	3.40128338670364	0.15262957311219
C	9.87022675095029	4.15393606223317	0.94079409904600

* * Contained two imaginary modes with frequencies -45.67 and -9.66 cm^{-1}

H	10.83436701401047	7.79691328489329	11.47850763380730
C	11.31912760608939	7.44751175733140	10.56378230845694
H	10.67676666901887	9.15304040056725	9.40986362902935
C	11.22793544686351	8.20949919619231	9.40242216767302
H	13.13772696380770	9.79611429057649	9.29206328780320
C	13.48834758251785	10.41543885867322	8.46500509820989
C	8.25063332003525	5.44782670271694	8.05224898617500
H	6.82093004144011	5.18943241637113	11.15087911035009
C	7.27737098532265	5.64748485097182	10.26988990747887
H	15.57681792286834	13.04204524295216	7.86774403873470
C	14.86586414475515	12.23658600677406	7.66698904620573
H	6.56003961189131	7.85042253281594	6.53934436772825
C	6.56179178228802	8.94087170633912	6.52340877596557
H	9.45404065481395	4.60787094123016	4.98670326446932
C	9.19901938129219	5.24121521718675	5.83392270743235
C	7.80092340692808	9.62979130805893	6.61547013956768
C	7.78087574070191	11.05548455169488	6.56724069411757
C	10.07884745760687	6.09760671602791	2.96879112129923
H	7.45030710925855	7.42975355074383	3.81013175565332
C	7.84426615511972	8.42570323542618	3.58954767131497
H	14.72520836842748	11.64718078054446	9.73610581872349
C	14.38717169222207	11.45328829713753	8.71503802593680
H	11.12911524215426	11.64761900300657	6.81252056839055
C	10.19502252655063	11.08846227487317	6.75245042096641
H	7.50499896652479	3.78602094637431	9.22561620591451
C	7.66010362926448	4.86818237667660	9.20521826144418
H	9.23722297600347	11.97062689540122	2.86014391316903
C	8.84279014185535	10.97198040616851	3.06222610254412
C	9.01557191988885	7.45136295402819	6.81172095508814
C	9.05323180070958	8.94171322454414	6.73468476761969
C	9.21027603165182	8.68404653061067	3.75694861709229
H	8.86621572503496	7.32227471884560	1.65701756707599
C	9.34704279017478	6.35507319857408	1.80232568466615
C	10.23714409333968	9.67184743403636	6.78806916178449
H	8.98607436415210	12.85327902483592	6.62926757106136
C	9.00404250261583	11.76033277844585	6.64936280563656
C	11.84687832379166	7.77561163978653	8.22411506647900
H	11.32690358310984	4.65375030586112	3.96737834062236
C	10.72025905652136	4.85755375707262	3.08696852693304
H	7.15735093212729	7.66893679802655	11.05895701855383
C	7.46592590787828	7.04717913558993	10.21531330802110
H	10.77035238020190	10.15350218492948	3.64516323849789
C	9.70423882438568	9.96351450066453	3.49141939408672
H	8.16036505979653	8.71728582945735	9.07813582452805
C	8.02873207269970	7.63519181236214	9.10671257087646
H	4.41018868082062	11.57090171245906	6.29282797479686
C	5.35902794219118	11.03604459847342	6.37823633240128
H	12.99955243756307	6.22892304818583	7.27915123611693
C	12.53557540990049	6.56019648792317	8.21154179131530
C	8.44196704454454	6.86015770673354	7.98877899826765
C	13.05603495877070	10.15506724801799	7.16035394931809

C	9.42285830902032	6.64017614305028	5.75213849042882
H	13.16576952554332	4.84926479275457	9.36372198774383
C	12.62393444789844	5.79746535894871	9.37477445429424
P	10.35446791944368	7.35275739338026	4.29962266767686
P	11.86827289962369	8.81604933385194	6.72094918675943
H	19.81788154380334	6.10715308923257	4.41031226319813
H	19.68731602635921	5.07885240986755	2.97671504377982
H	19.29776870509319	6.79902016324584	2.86747428976812
H	19.34906887334282	3.56132004336372	4.67355102540969
H	18.11593557317309	2.30141895758270	4.65977755049730
H	18.77110505822312	2.91623511612054	3.13345967926836
H	16.31350785823996	1.83030348600052	4.35385764998610
H	15.23174433462753	2.40007836419613	5.64385660133217
H	14.68209264682623	2.37593634870799	3.97148611031773
H	13.63720243671675	4.13442128297304	5.75725638129298
H	13.05772046253834	5.59480586218911	4.90948441762310
H	13.22167088312383	4.03526832159764	4.04480327550234
H	18.21862944847352	8.25914809983577	4.23096280064483
H	17.48323281153962	8.28800370826750	2.61937801544485
H	16.54583977299938	8.78421638595007	4.04909711058838
C	13.68196819324730	4.68645866595832	4.80441285734410
C	15.55763915068096	2.58178877497957	4.60557736910901
C	18.45008848459561	3.21062093441212	4.14748832506273
C	19.21021389849340	5.91285563020034	3.50855198378060
C	17.28619112478786	8.06669650759893	3.68153152625775
C	15.09211870140610	5.03026710847000	4.43862870347565
C	16.04387988024681	3.99057967054676	4.39543049717265
C	17.39048682590529	4.28067968212432	4.12339417960714
C	17.77531381966471	5.60767256761605	3.84968521029553
C	16.83106150730243	6.64638208298856	3.88919914009933
C	15.47685276271128	6.34775843480847	4.14891729365948
O	14.47296914513617	8.40848936605647	4.93548273879130
H	14.64635994055257	10.59382004050217	1.11003898251142
H	15.55105338277427	9.89422367214653	2.47095470097199
H	15.32519654458256	8.95846179926885	0.97994199461072
C	14.52025158448577	7.50253187328963	4.00341971341210
H	14.81545211494468	6.65863704448807	1.31320770045436
H	13.77053257256135	5.61518339322962	2.25334343894315
H	11.78607401648453	6.39369515682564	1.10764174830062
H	12.98718019590701	5.95253888223906	-0.10138962581290
H	11.78342034888955	8.12261526181706	-0.61249115423353
H	13.52643843901646	8.34449451858237	-0.48086532569895
C	12.75537002934672	6.68721028335292	0.68581116792369
H	11.46087831344358	8.86024154216065	1.72718698780854
H	12.28674246918572	10.11260647293203	0.79106912963036
C	12.62391475799505	8.08455835812774	0.10056176894240
C	12.40431295410320	9.09961885272877	1.21164564330084
C	14.83719848460550	9.66417327494567	1.66979978521955
C	13.81130478548052	6.60433209145801	1.77852607747782
C	13.53108956592940	9.10605279405005	2.24865277937176
C	13.73476456260585	7.69647862850835	2.82840252248101

Ir 12.45095458012472 7.92497802395048 4.74249305227411

Species: **MS-*anti*-η^4**
E(PBE0-D3BJ/def2-SVP, def2-TZVP(Ir)) = -1177.470565388960
E(PBE0-D3BJ/def2-SVP, def2-TZVP(Ir)/SMD(Toluene)) = -1177.483398117948
E(PBE0-D3BJ/def2-TZVPP)) = -1178.137576330334
H(PBE0-D3BJ/def2-SVP, def2-TZVP(Ir)) = -1177.19024959
G(PBE0-D3BJ/def2-SVP, def2-TZVP(Ir)) = -1177.27471654

Ir	-0.34370030936651	0.05111949401062	-0.75632766235838
H	-0.44325884467247	0.25040027457703	0.80480205064617
H	2.35646724265824	1.30857918459064	-1.82251527404744
H	-1.92123175406423	2.62594505052533	-2.00294396519381
C	0.49848841673167	-1.88460370787221	-0.57679765119984
C	-0.91542176700209	-2.02816218735146	-0.86138666014114
H	2.43819299045001	1.17574436233888	0.31857901941348
H	0.70325288541345	3.52720861069547	-1.46491323401189
H	2.24595563119530	3.68008319776177	-0.62536825223340
P	1.47147772854918	1.37942501218788	-0.70806012472602
H	1.04477790716664	4.51304454871095	1.12524803859753
H	0.59234811101597	2.86010339285417	1.48941966415216
C	1.25494563387432	3.20523930227677	-0.56601949326728
H	-1.04545906578748	4.66875270279903	-0.37076786971117
H	-1.22333743974561	4.70709070156147	1.37359766680024
C	0.51579069537130	3.64071636524770	0.71145716641374
H	-3.00183219553738	3.40478660237093	0.46858528525371
H	-1.95774230932751	2.32928607841155	1.41722554058054
C	-0.95081973551572	4.04360833234075	0.53602223469770
H	-3.26937172865571	1.34805611063080	-0.98820617360032
C	-2.00420948287613	2.93830485071598	0.49970669553028
P	-1.89504521880902	1.72194977800461	-0.89534103265849
C	-1.35547654572581	-1.43475493925536	-2.09254417433025
H	-1.63738482096673	-2.47931146555111	-0.17674449366105
O	-0.48299110146184	-0.84033620686013	-2.82677794671371
C	-2.78468868683269	-1.52738136660595	-2.54930871564086
H	-3.45951050574907	-1.85078983024951	-1.74519709840641
H	-2.84492297968186	-2.26310009684813	-3.36724683742191
H	-3.12248811711493	-0.56389511859404	-2.95699911391635
H	1.18747997005834	-2.08176571069452	-1.40701200476416
H	0.85120539640837	-2.24039332272991	0.39589441591832

Species: **MS-*syn*-η^4**
E(PBE0-D3BJ/def2-SVP, def2-TZVP(Ir)) = -1177.458233744902
E(PBE0-D3BJ/def2-SVP, def2-TZVP(Ir)/SMD(Toluene)) = -1177.473166832284
E(PBE0-D3BJ/def2-TZVPP)) = -1178.127114711211
H(PBE0-D3BJ/def2-SVP, def2-TZVP(Ir)) = -1177.17837209
G(PBE0-D3BJ/def2-SVP, def2-TZVP(Ir)) = -1177.26229282

Ir	0.02857108724711	-0.16864731066383	-0.79967702570052
H	-0.62906500920269	-0.02600352573386	0.67749793303821
H	3.31752714670038	-0.99564140184492	-1.94308114421256
H	3.00421711677102	-2.53426909930369	-2.76172679758015

H	2.49413085803238	-1.00999152296675	-3.53992499390489
C	2.59395887981603	-1.52842567653922	-2.57624574220741
O	1.24578968976385	-1.93172972292627	-0.58996431554093
H	2.91415287827806	1.25896306316086	-1.04353952076135
H	-0.96579200239202	2.38996829004373	-2.39207336993529
H	-1.10645668754908	-2.60128617550919	-0.98070273914687
H	-2.11529376125678	-1.52522942466809	-2.07681182587929
H	-0.04215557937721	-1.22415571197979	-3.56277714228296
C	1.27854960136518	-1.65354886629631	-1.87020583452314
C	0.02099483708499	-1.53479163605027	-2.51671283420375
C	-1.13101742624093	-1.72300844655567	-1.63677900035509
H	2.39438108238627	1.02336276529154	1.00785494606966
H	1.19639018018182	3.42150069758869	-1.13164304745906
H	2.41801625601968	3.54010528957335	0.13146913562299
P	1.73730420588093	1.25018027645585	-0.23385209442885
H	0.73877823250941	4.40906193905722	1.39408939263810
H	0.26273962360755	2.74464882030856	1.68527179221554
C	1.45404266257338	3.07094041213686	-0.11722228288535
H	-0.85140116824595	4.48404069946888	-0.58447815394785
H	-1.54078098807295	4.44758045953710	1.02770285193270
C	0.37591990832189	3.50008140167860	0.88899805242443
H	-2.87105663237478	3.10297587416474	-0.45119220892248
H	-2.16082598673714	2.06108963110077	0.80247286341013
C	-0.99370228149090	3.83091834866400	0.29547175917517
H	-2.57699234113896	1.06490778038294	-1.90752801693125
C	-1.92455317348953	2.67930069913580	-0.07838610750304
P	-1.33531120897101	1.48056207328839	-1.35203452821487

Species: **MS-*cis*-η^2**

E(PBE0-D3BJ/def2-SVP, def2-TZVP(Ir)) = -1177.444730789749

E(PBE0-D3BJ/def2-SVP, def2-TZVP(Ir)/SMD(Toluene)) = -1177.457804506649

E(PBE0-D3BJ/def2-TZVPP)) = -1178.112715864227

H(PBE0-D3BJ/def2-SVP, def2-TZVP(Ir)) = -1177.16626839

G(PBE0-D3BJ/def2-SVP, def2-TZVP(Ir)) = -1177.2519623

Ir	0.03984774351983	0.07062591513162	-0.92235885697750
H	-0.35423363871244	0.10762474583550	0.55464346912830
H	3.09893869006241	-1.45578823926918	0.98861197640403
H	2.88598148435474	-3.15176773242952	1.45155763516000
H	3.17776218935281	-2.71367599708868	-0.27035728069165
C	2.67458779584972	-2.41947667809538	0.66157520922766
O	0.45284405511488	-2.35908647234052	1.48875889261589
H	2.84837872323180	1.61887198400337	-1.39343993648708
H	-1.67590798305457	2.45641358901080	-2.28939412913346
H	-1.43439926645307	-2.16220248477125	-0.29250720860512
H	-1.12485352723921	-2.14501126593095	-2.09839736229915
H	1.33712152507954	-2.23531917215569	-1.66806064792773
C	1.17153879178618	-2.27066091512088	0.50983415849589
C	0.66363306402687	-1.96367191950509	-0.84494093408970
C	-0.76315519606778	-1.85474780120229	-1.10258473040688
H	2.58069614738986	1.16094307008274	0.66573564574150

H	1.22914157175165	3.75187393728792	-1.07960366537410
H	2.44603861648824	3.73159901527875	0.19932546995024
P	1.77281481921327	1.48972510727213	-0.46253338673644
H	0.73729738547863	4.46728590820724	1.49611313405665
H	0.33059414405056	2.77304867009079	1.66632440445684
C	1.48898641085503	3.28648906970793	-0.11376311801224
H	-0.90309475372930	4.48946041695233	-0.55255972693889
H	-1.47182168932522	4.61446511766479	1.09871537599719
C	0.40001261128642	3.58854268405793	0.92455584619046
H	-2.99314414011017	3.20975198367780	0.00150668811659
H	-2.07843966378817	2.17430450547966	1.10878918668234
C	-0.99888837625346	3.91718552032647	0.38785141108518
H	-2.96814832912002	1.06157177966288	-1.34142765398198
C	-1.99647809877689	2.77915711904020	0.19011373919468
P	-1.64638941450365	1.56879167225280	-1.16845962760453

Species: **MS-*trans*-η^2**
E(PBE0-D3BJ/def2-SVP, def2-TZVP(Ir)) = -1177.442994395325
E(PBE0-D3BJ/def2-SVP, def2-TZVP(Ir)/SMD(Toluene)) = -1177.455890036432
E(PBE0-D3BJ/def2-TZVPP)) = -1178.111819810679
H(PBE0-D3BJ/def2-SVP, def2-TZVP(Ir)) = -1177.16581313
G(PBE0-D3BJ/def2-SVP, def2-TZVP(Ir)) = -1177.24904586

Ir	-0.13511253317602	0.06322380415695	-0.85473744825559
H	-0.59735508208372	0.11194317208705	0.60522513432592
O	2.58777947161336	-2.21983034403561	0.40095327211462
C	0.61083672906815	-2.32262589859170	1.69804475751238
H	2.74150596765225	1.47371021861874	-1.32295870713014
H	-1.84507577669246	2.32933882240503	-2.33560212849976
H	-1.47155804390319	-2.27441732608089	-0.22968420942073
H	-1.20353471203268	-2.20979445475994	-2.02226531188605
H	1.25937021774742	-2.09311744046400	-1.73441695381658
C	1.36985658644855	-2.17293459620876	0.39167197693126
C	0.62277424959503	-1.91332933948352	-0.85656150625626
C	-0.82398580359643	-1.91100457844493	-1.03568665142578
H	2.47103452528872	0.86327623725363	0.69570482269426
H	1.54849527364282	3.75120912169314	-0.81425751941551
H	2.49864535264810	3.43918386954267	0.63867938715246
P	1.68350614782916	1.38100422659494	-0.37227444303780
H	0.65101513205279	4.35949790472037	1.63180748829337
H	0.11272536007280	2.69423648489316	1.67197238826948
C	1.56699629241582	3.16645013327797	0.11914844128940
H	-0.64492350520258	4.34151920267463	-0.76649574226813
H	-1.34741466478884	4.81714821450790	0.76192760871541
C	0.35136549548903	3.52240533492009	0.98233620698844
H	-2.98206622871597	3.46235581340945	-0.18753521790177
H	-2.24999336716290	2.45053302160939	1.06431861454545
C	-0.91990839134123	3.95268649617062	0.23018124535649
H	-3.15387089838006	1.11075869988778	-1.18494077367772
C	-2.05451339877143	2.93864339874521	0.09448002247283
P	-1.80318145852195	1.57077006562542	-1.12561795959083

H -0.42205441415055 -2.67502820683524 1.57542212081816
H 0.57951737497874 -1.34290670466707 2.20096240554024
H 1.17095410197725 -3.00870535322248 2.34642867956249

Species: **MS-sqp-η²**
E(PBE0-D3BJ/def2-SVP, def2-TZVP(Ir)) = -1177.442293093595
E(PBE0-D3BJ/def2-SVP, def2-TZVP(Ir)/SMD(Toluene)) = -1177.457053967805
E(PBE0-D3BJ/def2-TZVPP)) = -1178.113605314014
H(PBE0-D3BJ/def2-SVP, def2-TZVP(Ir)) = -1177.16353071
G(PBE0-D3BJ/def2-SVP, def2-TZVP(Ir)) = -1177.24865387
Ir -0.16687737659281 0.02853917312500 -1.07244228952622
H -1.42998438579390 -0.59409391679333 -1.86071038331767
H 3.18569892864237 -0.57270579193125 1.05627785074315
H 2.94670684702662 -1.80859239457853 2.34314528659545
H 3.54490875655538 -2.26751318659788 0.71198415172805
C 2.86406654538516 -1.60529004173082 1.26768282242362
O 0.56985425796535 -2.14664597017520 1.59062544460614
H 2.56554494477201 1.45352930417525 -1.65241544118377
H -1.68116158390216 2.72369051697199 -2.04733014335812
H -0.73958828860012 -2.55710152573661 -0.51426736820806
H -0.06111834725035 -2.44913080323065 -2.23423189137440
H 2.08516845959270 -1.57924673514912 -1.27719256277180
C 1.43731721815760 -1.81233228071120 0.81176576325209
C 1.18954936074711 -1.62299020121812 -0.64610841834167
C -0.03462556599493 -2.08200378536648 -1.20469202458602
H 2.41392649964762 1.35887531744150 0.47234101034608
H 0.66395504884753 3.59806161577857 -1.43383779996542
H 2.20050784354460 3.80810906376260 -0.60085324137230
P 1.54428873489016 1.47672618418653 -0.65707870562819
H 0.92300120285256 4.64258493106062 1.11002318163393
H 0.67974092633376 2.96389627759977 1.54777563409733
C 1.22977767728075 3.29464597522901 -0.53731364711874
H -1.21065495619242 4.60128511829477 -0.23439629140839
H -1.36532101631370 4.45772452530313 1.50753822795063
C 0.48648459203884 3.69906043063705 0.74621355887142
H -2.95403199914090 2.99308744012249 0.51432899556539
H -1.73157325176651 1.98099842309636 1.30723479554752
C -1.01781346357622 3.91632338529329 0.61158717924521
H -3.03518923968462 1.27546231070693 -1.29555936165432
C -1.89849550766020 2.67941076802248 0.47179345725527
P -1.69170286181125 1.67071587241184 -1.07625779004618

Species: **MS-EtOH-η²**
E(PBE0-D3BJ/def2-SVP, def2-TZVP(Ir)) = -1332.223574372495
E(PBE0-D3BJ/def2-SVP, def2-TZVP(Ir)/SMD(Toluene)) = -1332.241076276084
E(PBE0-D3BJ/def2-TZVPP)) = -1333.064863283505
H(PBE0-D3BJ/def2-SVP, def2-TZVP(Ir)) = -1331.85363578
G(PBE0-D3BJ/def2-SVP, def2-TZVP(Ir)) = -1331.95555282
Ir 0.00486513295111 0.07626887418439 -0.90674598241792

H	3.04089056149476	-1.33942099127839	1.18161674693455
H	2.87623525435659	-3.03905709957563	1.64439371577203
H	3.11176161709940	-2.59195548617361	-0.08508308607080
C	2.62855681146513	-2.31050691346564	0.86101246078732
O	0.44497931985682	-2.23611592164441	1.76882123900248
H	2.83181355519480	1.61952906805249	-1.48012640343762
H	-1.82739414422248	2.50375650200381	-2.16601480744388
H	-1.50615851705133	-2.01532561648628	0.06663619100007
H	-1.27996189381254	-2.25043985008941	-1.73483099390372
H	1.18733471480238	-2.32486517644540	-1.41895004518251
C	1.11886081983890	-2.18607400010420	0.75668471884575
C	0.56297236054048	-1.95985169768729	-0.59490752242628
C	-0.86871670399833	-1.83393879176435	-0.80568208232743
H	2.62921050346341	1.07161841848732	0.55623840852448
H	1.29524780969235	3.75647750181262	-1.03808806622214
H	2.52582444042922	3.64218346733049	0.22463380866798
P	1.77730478243009	1.45253163149166	-0.52282634404432
H	0.84218676477174	4.43299387029033	1.52201127504826
H	0.43992821222318	2.73871547598179	1.71466111485377
C	1.55046480571034	3.24342284156758	-0.09514499705068
H	-0.85980180997989	4.43078470168172	-0.48508393973734
H	-1.36877596697757	4.59340648521810	1.18171275076974
C	0.48780897429923	3.54994979899818	0.96710518836004
H	-2.94584924210230	3.17057562332475	0.23272311788067
H	-1.93664076709339	2.11043964061253	1.23362925587329
C	-0.92644957429892	3.87754131371579	0.46967660937627
H	-3.01323922677202	1.06305040946584	-1.17577920122356
C	-1.93561065678425	2.74103459987969	0.32918095260466
P	-1.68163284421420	1.57208053475811	-1.08410447033643
O	0.39267339905303	-0.28339200294660	-3.18040057137717
H	1.06458180823109	0.34588314948649	-3.46475497645722
C	-0.70818167131778	-0.23400177552430	-4.07914680879497
H	-1.41836089661571	-0.98374481344277	-3.70462526787111
C	-0.28821258479458	-0.53121645756137	-5.49939675619158
H	-1.20790649491839	0.74883478403782	-4.01374499833428
H	0.18685986634255	-1.52108941255428	-5.57116936809685
H	0.42787529492964	0.21899092687419	-5.87194065614604
H	-1.16242743969279	-0.51217949994315	-6.16741498124736
H	-0.26639637452980	0.17366588743135	0.61749477203988

Species: **MS-EtO^{-}-η^{2}**

E(PBE0-D3BJ/def2-SVP, def2-TZVP(Ir)) = -1331.667001966963
E(PBE0-D3BJ/def2-SVP, def2-TZVP(Ir)/SMD(Toluene)) = -1331.716273071967
E(PBE0-D3BJ/def2-TZVPP)) = -1332.511785401621
H(PBE0-D3BJ/def2-SVP, def2-TZVP(Ir)) = -1331.31323275
G(PBE0-D3BJ/def2-SVP, def2-TZVP(Ir)) = -1331.41171907

Ir	-0.15791948820559	-0.27033587847360	-0.46485567403819
H	2.67177262140535	-1.90997875468785	1.28878118232567
H	2.54910277007617	-3.61671399833234	1.81438993647463
H	2.99376887368454	-3.20473170858405	0.11780262587330

C	2.37039464746869	-2.92369316469912	0.98101233635624
O	0.09078014175283	-3.39718804231290	1.43376727638539
H	2.71283443208828	0.86852776616290	-1.04237515393446
H	-2.71022792221841	1.62382161296051	-1.30284768954893
H	-1.60951907983765	-2.64983814662823	-0.31682670003280
H	-1.15900170152237	-2.23535667534557	-2.05606233153871
H	1.25633733171720	-2.34296547534891	-1.44018414267020
C	0.88438727954150	-2.91939662448242	0.63670487957520
C	0.50680043904124	-2.30112898719669	-0.64090729264237
C	-0.89698815766650	-2.16483919069606	-0.99251335056052
H	2.20528153571187	1.81576153181868	0.78235145066321
H	0.61057612666663	2.56431846749324	-2.13081476065255
H	2.15918732439595	3.24943606624595	-1.61525961436677
P	1.52399532655297	1.25019874100337	-0.35394561971626
H	0.69747098257231	4.89958565041322	-0.92956911840680
H	0.94734445767421	4.01383751352251	0.56613907425715
C	1.19986225393275	2.85163769658463	-1.24566462102294
H	-1.49389549511464	3.79838697502412	-1.28478099480595
H	-1.35349803620321	4.82967019580149	0.13036184724879
C	0.48683016006154	3.93395305250956	-0.43640872579741
H	-2.59379505704354	3.10679432038816	1.01383842810218
H	-0.97091835989248	2.56312969945803	1.47456539303492
C	-1.03205722437697	3.85681137202099	-0.28188300233572
H	-2.96524519049768	0.65420793573760	0.55773415161003
C	-1.62598180702707	2.76380101341110	0.61025064637112
P	-1.87548996885512	1.12110894228946	-0.24134171543170
O	0.47576332981278	-0.00712749968879	-2.51780609163656
C	-0.38674701251767	0.53366885595923	-3.41416561520196
H	-1.27891938670748	-0.11219953683969	-3.62124084167326
C	0.31554418938633	0.82792957612050	-4.73977324059731
H	-0.84167406792823	1.50384424059918	-3.06046096385829
H	0.73732239747948	-0.10056882354974	-5.15875338799137
H	1.15895552217210	1.51807338671979	-4.56978345096196
H	-0.36464500886578	1.27965388580285	-5.48361910415001
H	-0.44021917871437	-0.54460599118113	1.08140397529519

Species: [IrH(*R*-BINAP)(*Si*-MVK)]
E(PBE0-D3BJ/def2-SVP, def2-TZVP(Ir)) = -2711.093207724845

Ir	12.59139857752807	7.19844149929525	4.73468876447276
C	14.59760240103155	6.55886968037870	4.39925499957624
C	13.93928796076844	6.69669343874173	3.11928633592553
C	13.40300530744549	7.99448766850391	2.83345757154462
O	13.59639671452942	8.93181951572942	3.71277203999571
C	12.70992618513980	8.31066936528305	1.53923452424978
P	12.58741094471777	7.67779961643827	6.95188524156293
P	10.35765056692239	7.68460240549380	4.46998492903121
C	11.33029592046333	4.21461442545725	8.75974505872028
H	11.11414345470949	3.18940567706008	8.44982941731064
C	9.38648167940000	7.07895937271780	5.91829553030534
C	14.25694016905505	8.18235112764980	7.52516519426215
C	8.80701059358596	7.15454319303463	8.29203093126364

C	11.79294858088301	5.13352401489084	7.82064476162472
H	11.95009016638584	4.83931835998829	6.78157945292207
C	7.80681285394517	12.59868223288030	7.65910609551238
H	7.20414795756277	13.50624524094485	7.73732965834914
C	8.81047799021247	7.78925206837029	9.56292276016055
H	9.31124163957461	8.75169392653703	9.66933913882207
C	10.76678350868912	10.39352450315228	3.98120280142033
H	11.82107425014354	10.10551519995855	3.89868427460758
C	8.19928172081738	7.20836257376902	10.64866443149537
H	8.21705803520296	7.71689913343257	11.61559733532864
C	10.20474414612028	5.66334488469727	2.55101240825215
H	11.09322442514918	5.29499785456653	3.07006446209472
C	12.03786939152344	6.45704987036752	8.19505664528731
C	11.38474373157054	11.57017610122395	7.50911268931113
H	11.87314643432012	12.54537068605557	7.58195900780778
C	11.52948096831872	9.15165185015776	7.31002064449436
C	8.48703017360782	7.33731929738604	2.32183500598359
H	8.01607952788101	8.26629619261171	2.64110320460258
C	9.80724252461003	9.42020474428478	4.28217082839197
C	10.13900105833571	9.04926572931329	7.30511004172547
C	9.44556978061351	7.73533057924383	7.14767499929177
C	10.37492439841971	11.72015230670361	3.79972768114800
H	11.12856814176111	12.47586814664679	3.56822076341102
C	7.54078864013318	5.31243897658148	9.31166114941796
H	7.04829749948364	4.34320998541747	9.20293870225956
C	12.13759081420835	10.42739736370220	7.43013966154003
H	13.22505506654006	10.50118656562165	7.44916953594199
C	16.11405805837988	8.15398934041795	9.08189497332321
H	16.56126877042420	7.77726143884188	10.00480582813271
C	8.46350875339965	9.79489449998559	4.42104364981864
H	7.71713564664491	9.05020610050159	4.70937989770467
C	9.59644208989568	6.83835896853348	3.01407017004005
C	9.97204788755644	11.50770950570860	7.51932759393594
C	9.33919368541132	10.23001112191879	7.45031461918840
C	8.70742720532231	5.83892546325814	5.81348683515845
H	8.65780205281660	5.34140338107917	4.84302609494252
C	7.92051608940269	10.18451469254266	7.50965710956313
H	7.41797067634659	9.21764406370889	7.46686749296898
C	16.80886321012652	9.06145001546851	8.28563147465348
H	17.79385305220050	9.42044779860656	8.59341689740648
C	7.55930468886221	5.95563777200331	10.52603814278350
H	7.07941484609303	5.50075763265534	11.39559533468198
C	8.15140618008627	5.89287786333035	8.17062622312360
C	14.84335385453445	7.71738363796188	8.70689276065525
H	14.31028732265101	7.00815150641667	9.34202388609519
C	11.83811152457127	6.84372225595300	9.52644285145012
H	12.01051239944080	7.88146952232611	9.82359206511165
C	11.39401878646807	5.91968008732934	10.46655189504169
H	11.22897990835763	6.23579847521145	11.49895732730561
C	8.59112163550517	5.49500620402139	0.76088581141953
H	8.19911577347141	4.97437088302756	-0.11597916799045

C	9.03444370602356	12.08282095473120	3.91359748007072
H	8.72907165642199	13.12143456506449	3.76532137898235
C	11.12726754225705	4.60589549274091	10.08191334378789
H	10.75223195373943	3.88823552282247	10.81477396497669
C	8.07825732939161	11.11768508321958	4.22723703643449
H	7.03031780398005	11.40335726894931	4.34255646671795
C	8.12013202370330	5.25583667709337	6.90736877533317
H	7.60842280127787	4.29541686237945	6.81303035629519
C	16.24358611784448	9.50264185894493	7.08868483720458
H	16.79320104871791	10.19132901613727	6.44209097426874
C	7.98849879807035	6.67042292194026	1.20432451490397
H	7.12864016779941	7.08198054616749	0.67069271567604
C	9.17770117996504	12.67872037520509	7.60864421169189
H	9.68221608361031	13.64758402307081	7.64812580906688
C	7.17670050646616	11.33567327250565	7.62091816281759
H	6.08708320073006	11.27146572917607	7.67427633822371
C	14.98595007338716	9.05219115198397	6.69639053911920
H	14.56699907593672	9.36240026692673	5.73333709004296
C	9.70267520291126	4.99377097862861	1.43689914424603
H	10.19390515931665	4.08341082119929	1.08513099214819
H	12.07999730231359	5.75112113329070	5.07053125412405
H	15.29477972604133	7.35309533470849	4.69000755467521
H	14.91146202542895	5.55632472376260	4.70448365397064
H	13.75712933894681	5.86848766101254	2.42940691122466
H	12.38476460058187	7.40161176495037	1.01568867011921
H	13.41014268740559	8.86088335007400	0.88912002148402
H	11.83819488845265	8.95705503821695	1.71165935645461

Species: [IrH(*R*-BINAP)(*Re*-MVK)]
E(PBE0-D3BJ/def2-SVP, def2-TZVP(Ir)) = -2711.079981334530

Ir	12.53312637309980	7.01352316607225	4.62236351023341
C	14.09410140968668	6.36560465142373	3.22719452143117
C	12.81395336709565	6.54531642067544	2.58509716104059
H	12.68747052825183	7.45160550526675	1.97852849880048
P	12.63902423282425	7.53729163699317	6.88454499975431
P	10.34650303052571	7.56457259925150	4.46851635791267
C	11.01762427084437	4.20972012978885	8.67516172582400
H	10.66635805873778	3.22596520222071	8.35505874293203
C	9.34709523063792	7.04601529199138	5.93210717569821
C	14.28602601097071	7.99534723573827	7.56491916880577
C	8.80589638664893	7.21121194616687	8.31089335239979
C	11.59102980222871	5.07254415664531	7.74339457245442
H	11.70409720779842	4.77168809701190	6.70030410715209
C	7.99759434545541	12.62555269884619	7.60322316364631
H	7.42162523156661	13.55077222347390	7.67768321916158
C	8.88354212323872	7.86541054417302	9.56947020892832
H	9.46305467461029	8.78543518571141	9.64988940977671
C	10.94604760731602	10.23663509563780	3.98561943161562
H	11.97915661192725	9.87861684534684	3.92527834198154
C	8.25122175154773	7.35200862612253	10.67695474452207

H	8.33387779005082	7.87183869236109	11.63445614944287
C	9.78385206643362	5.37861927632800	2.80823059679094
H	10.45387927375705	4.84189640365179	3.48450013844171
C	11.99866512154732	6.35231545612951	8.12637196077043
C	11.53985520166740	11.47822934980714	7.52051662935566
H	12.06128828520954	12.43240770513732	7.63200323376545
C	11.61864638282627	9.05402340709131	7.26544672928226
C	8.70694091479478	7.42504153728729	2.13468285380672
H	8.50598536475509	8.48755510418658	2.27550214783922
C	9.91868237820353	9.32564783554551	4.24928159187801
C	10.22460656071959	9.00142828934419	7.26573181697264
C	9.46754065185754	7.72048586473204	7.14540982673884
C	10.64480357414988	11.58789209426449	3.81842470430756
H	11.45135097053861	12.29816581817460	3.62510335819953
C	7.41654704465740	5.49236403730764	9.38734542379398
H	6.84838191572527	4.56262884658416	9.30457641210363
C	12.25401423070273	10.31231262212566	7.42772894532055
H	13.33930489690171	10.36160009933367	7.48620373248964
C	16.10555362764757	7.76772682795957	9.15604880124987
H	16.53940651682748	7.25836778247305	10.01983295317616
C	8.59499495238943	9.77566247692817	4.33516425617949
H	7.79467623237903	9.06791802089440	4.56581996538761
C	9.51912519892077	6.73696804279330	3.04189408069431
C	10.12759129346098	11.46562936980350	7.49700917952090
C	9.45949208972875	10.20854089706843	7.40536063143677
C	8.56891560250169	5.86238648622547	5.86406239415557
H	8.45736733471021	5.35283988027098	4.90659337222061
C	8.03880348824637	10.20929107402706	7.43751099363919
H	7.50598657420650	9.26019202896816	7.37658214507472
C	16.78329740588932	8.82684518647332	8.55446718739773
H	17.73892548696347	9.16974062695698	8.95778988491510
C	7.51054236504099	6.15252290271713	10.58955308622410
H	7.01467568856374	5.75000925127539	11.47592607894769
C	8.05090067198296	6.00302777095227	8.22608824681237
C	14.86553719749717	7.35639749122287	8.66895263932135
H	14.34136085111832	6.53782126646921	9.16417692447756
C	11.85593881392725	6.74459125171448	9.46414326527802
H	12.15496373600408	7.75024621657970	9.77086897853751
C	11.30856234671288	5.87241296170184	10.39863013970799
H	11.18896417265269	6.19704036125275	11.43478143710465
C	8.41677502023083	5.41910346018029	0.81557385654975
H	7.99258441431151	4.90727423224468	-0.05144417405315
C	9.32578345642314	12.03160220832772	3.89852122612834
H	9.09232672330821	13.09082408918530	3.76611905427307
C	10.87291358010888	4.60795331182337	10.00261136756730
H	10.41074258053874	3.93655060992045	10.72946327177896
C	8.29944801451333	11.12247711699669	4.15203434888430
H	7.26693665168844	11.47069084232681	4.22882343624616
C	7.94938891958261	5.34942937203993	6.97546388493060
H	7.35881641532974	4.43316077182529	6.90302366286723
C	16.23727406649034	9.43886977575164	7.42709164569261

H	16.77813365526732	10.24269977755474	6.92151352128884
C	8.15965324812057	6.76950615609146	1.03011268699674
H	7.53720437807996	7.32919733153734	0.32762617854066
C	9.37095921677064	12.66170875317002	7.58248674979212
H	9.90622691130245	13.61290049006667	7.64334608603112
C	7.32978214212401	11.38314843892275	7.54045312953726
H	6.23770715146835	11.35382056661360	7.56837934313370
C	15.01188749328437	9.01132334339246	6.91947123774516
H	14.62250630924873	9.44854804719955	5.99528257358780
C	9.23468755940714	4.72554671341181	1.70941798264701
H	9.46789412091923	3.67237920571536	1.53383305376246
H	11.98114232628109	5.59175690501464	4.98926462723777
C	14.66175311950621	7.52018210820786	3.83807574250332
H	14.61718766537810	5.40870950711555	3.29166629921117
H	12.33618974734740	5.65801337885453	2.16277102003231
O	13.96893998087277	8.61242749579952	3.86291114092240
C	16.08243743300028	7.50588379657438	4.31669363635331
H	16.56952429970923	6.54490064006711	4.10419148235696
H	16.16915046523485	7.72974857133649	5.38655996148334
H	16.62028040726893	8.29993511005418	3.77629665914135

Species: ***Si*-INT0a**
E(PBE0-D3BJ/def2-SVP, def2-TZVP(Ir)) = -3293.682434849341
E(PBE0-D3BJ/def2-TZVPP)) = -3296.530338984674

Ir	12.28534541717746	7.33970717474794	4.43974338617679
C	12.86096109005393	6.43800007233302	2.57894373509257
C	14.01966161544144	7.00242125168314	3.29499416430576
C	12.40620382871090	7.09392782103533	1.27847842285620
C	12.72741645730382	4.93474155310017	2.46461151079839
C	14.04016085002882	8.50861871260464	3.19016961347425
C	13.82795483881690	9.05751260404092	1.77986443128816
H	14.91930896164229	8.92871236926667	3.69384056943829
H	13.15268918071862	8.98144084706869	3.79492946960736
C	12.47885831150918	8.60733256280026	1.25408962765430
H	14.62549644085313	8.65967820743214	1.13068865010333
H	13.92421282363750	10.15601775380497	1.76880907872365
H	12.29703363829513	8.98987334581962	0.23715765275866
H	11.68168917437460	9.02896665298290	1.89185193704149
H	11.38019144724176	6.76129570214264	1.05517001870525
H	13.03739925985233	6.69489031392809	0.45888556863915
C	15.14326505482404	6.22535090629339	3.85301436288977
H	12.88187983476551	4.41297288465753	3.41364316047652
H	13.47564695892748	4.53448385034644	1.76078125332204
H	11.73569844213531	4.68424198522929	2.05913019548102
O	15.19316770741287	5.00613715554119	3.79217595272170
C	16.28176992896344	6.92705657284329	4.57611492183277
C	16.51470621013311	6.58939434944190	5.92781929644444
C	17.63720597897034	7.12015895779875	6.58243249986592
C	18.50161801305207	8.01335281049251	5.90468529727451
C	18.28459040178211	8.31447693389743	4.55090777989725

C	17.18848553448523	7.73197004976818	3.87660935441064
C	15.58116052454689	5.62398566799206	6.59780981823209
C	17.99653406442591	6.72798855759308	7.99131893733581
C	19.65929002358011	8.58974609042038	6.67414055133394
C	19.20796752588168	9.19670278968109	3.75299588540798
C	17.07114553462045	7.91404990006230	2.38858625545677
H	14.54431572741130	5.80119805987351	6.27718265377921
H	15.60494244945274	5.69946033791118	7.69050213323642
H	15.82599855266795	4.59159461432064	6.30400051026537
H	16.88188868243528	8.96120794036193	2.09883546461962
H	16.27281529616592	7.29730860320432	1.96138622512482
H	18.00650898937437	7.61554908095124	1.88916667531566
H	19.87480572670799	9.80284699561140	4.37626022844826
H	18.64333644230216	9.89070881970254	3.11280384011889
H	19.84841090133694	8.60165159913702	3.07785757619737
H	19.30531794076227	9.21749293880173	7.50944066199456
H	20.32165831687023	9.20755281732665	6.05854873662794
H	20.27782941047001	7.79930049018902	7.12806729141977
H	17.19479054625309	6.18221893275150	8.49757495664509
H	18.23840565189410	7.60231302149992	8.61151807895310
H	18.88865781051388	6.07831580858777	8.00277831457300
P	12.44132911752137	7.90920759829257	6.70385149707941
P	10.05658798040635	7.82064992668884	4.35375568138299
C	11.43603620612132	4.30480779886944	8.42961915546512
H	11.30190088895258	3.27421395844158	8.09218764313004
C	9.15189428219045	7.16801455925810	5.82978136143320
C	14.04759955551623	8.54502513612691	7.35954708338440
C	8.69529734725224	7.19010745647705	8.23413816448658
C	11.82359147396884	5.27955543610134	7.51349234144965
H	11.99466491419333	5.01179863182850	6.47007091658793
C	7.41989476428309	12.50747220548946	7.92852273707101
H	6.77346953814113	13.36873567845756	8.11039207624984
C	8.75068184578020	7.80253777436857	9.51524135064526
H	9.24482912043099	8.76838764787465	9.61757036788088
C	10.42372037438596	10.59501255487343	4.15140963694734
H	11.48699659172172	10.34758714009107	4.10670474235479
C	8.19653540945470	7.19768099913539	10.61824253451440
H	8.25563933173995	7.69081713849349	11.59163351148778
C	9.37239359223242	5.71073454233297	2.64219182246529
H	9.97900418539432	5.11038910977587	3.32217380783212
C	11.97413267648159	6.61055344269324	7.91479191658893
C	11.04321065124515	11.68874717671257	7.61395645133988
H	11.48617874854252	12.67515265592078	7.77566700872837
C	11.30977224364019	9.30149344811142	7.19529648749477
C	8.50031300008805	7.84793647203496	1.96005901029064
H	8.38285820210945	8.92184411287357	2.10885567413637
C	9.49346417968605	9.56360547046337	4.28706966914747
C	9.92616683311803	9.12956163044204	7.23180857574657
C	9.27213615544533	7.79712860966828	7.06881854297882
C	10.00360561736533	11.92319904013866	4.10564612530619
H	10.74153100133294	12.72312365931292	4.01487293016758

C	7.49156935794192	5.32004700034522	9.28179917476945
H	6.99919512310547	4.35033607935705	9.17654153259214
C	11.84883961549984	10.59953067933588	7.40216928076463
H	12.93060044065123	10.73512198805099	7.40005079742068
C	15.72176769331859	8.83066820047417	9.09169574694484
H	16.10872023201021	8.55412190654053	10.07565942857731
C	8.13041634155874	9.87978083846423	4.37175061227180
H	7.39506856254046	9.08018142895144	4.49560694104354
C	9.20392121245411	7.08025728515065	2.89636950152105
C	9.63628221180783	11.55366581087326	7.64992320547273
C	9.07071367977069	10.25555415091148	7.47319476332900
C	8.46157425209497	5.93291488181761	5.74037349047681
H	8.33382872906423	5.45979548927517	4.76751781658081
C	7.65713933743433	10.13053861331788	7.53769337951023
H	7.20457271962371	9.14816217471340	7.40052392324381
C	16.39148031654525	9.77147301148941	8.31292842096040
H	17.29241168125754	10.25913259665219	8.69305865004190
C	7.56302457419656	5.94078180461732	10.50563882535878
H	7.12703314637907	5.46694400504225	11.38804696437697
C	8.04258265831835	5.92702212198313	8.12475103516520
C	14.56334616803723	8.21743646691857	8.61841794516834
H	14.06322023646303	7.47328417397412	9.23938904283827
C	11.74432512215892	6.94207329347118	9.25658987284752
H	11.82346479299453	7.98280692209555	9.57965370021411
C	11.37858925260014	5.96449696703709	10.17634657913827
H	11.19344973798143	6.24355786917702	11.21571421579095
C	8.09361311684699	5.88845362477538	0.59990780891743
H	7.66022659823846	5.42412851954548	-0.28908074286289
C	8.64673369316856	12.22964081324565	4.18513034327713
H	8.31397395467832	13.26985937598447	4.15312609838021
C	11.21380589626222	4.64359903150264	9.76258648522500
H	10.90439422281222	3.87970355632190	10.47915845751934
C	7.70988936317782	11.20493478603952	4.31293116320882
H	6.64645512646727	11.44328797446486	4.38757358370701
C	7.93725773293337	5.32327861903035	6.85056603027785
H	7.41384775415728	4.36901977357998	6.75896945656256
C	15.92065069410215	10.06371870406348	7.03469758452702
H	16.46183357315261	10.76284123312184	6.39352064775637
C	7.95043810557035	7.25529542305147	0.82241631883050
H	7.40929576746380	7.87457265181092	0.10271801513126
C	8.78405564250386	12.66513954818299	7.86949914652787
H	9.23861876331130	13.64979540778529	8.00610984991910
C	6.85533086293426	11.22300558977369	7.76795905064515
H	5.77171698412665	11.09418677347193	7.82520196102687
C	14.76484453314577	9.44823098048247	6.56275297044176
H	14.40287718382137	9.68578341634859	5.56316163264020
C	8.81450584787120	5.11833646803454	1.51301202087109
H	8.96593327321655	4.05164894434440	1.33007476773525
H	11.73580076670472	5.95019554657244	4.87511697317609

Species: ***Si*-INT0b**

E(PBE0-D3BJ/def2-SVP, def2-TZVP(Ir)) = -3293.665702365079

E(PBE0-D3BJ/def2-TZVPP)) = -3296.518401969054

Ir	12.57782439798099	7.40633009924729	4.83089208839798
C	13.66221532915726	6.67890192613943	2.89221724758024
C	14.58012090559903	7.18473457321646	3.98741887933927
C	13.84251363494734	7.29978625264025	1.49113931037137
C	13.44794406046384	5.17800093346537	2.72103150932019
C	15.04555339064869	8.64073481393867	3.79364616282810
C	15.39407471806503	9.01608167708573	2.35827610220034
H	15.87903135507771	8.84605788674395	4.47589921433290
H	14.23245777741501	9.32751751500776	4.09509664369127
C	14.19243723074212	8.77312231280404	1.46868476338584
H	16.23915077731384	8.41391115957581	1.98603282580214
H	15.71426671295279	10.07066997696582	2.31728846306639
H	14.39339305442098	9.09342244846249	0.43342815707892
H	13.33552616285998	9.37085584744221	1.82846493200523
H	12.93899052747854	7.09855092439196	0.89403725655155
H	14.65833680177199	6.73113640525921	1.01140059408207
C	15.52869264572714	6.22276483404984	4.61134295224408
H	13.23311662036251	4.67468943948318	3.66939746111121
H	14.34922178329200	4.71121318621692	2.30556649181780
H	12.61175429438445	5.01762354022146	2.02276042699428
O	15.54067096334146	5.02267484283996	4.37505701850584
C	16.53122935227286	6.69219618057601	5.65684965123828
C	16.36814288509201	6.23406875815940	6.98035256530523
C	17.34374394722157	6.54401107442776	7.94078752303693
C	18.44161945598028	7.36659390326483	7.60700436279103
C	18.62511025363377	7.77597018348280	6.27718033437249
C	17.69847229272453	7.37478753463612	5.29173325956214
C	15.20195463689125	5.35982202859309	7.33737364647551
C	17.27103519442671	5.97719132684121	9.33111516837654
C	19.40552937730122	7.73286194023041	8.70169280156578
C	19.80518619308124	8.59523753354721	5.83035892731033
C	18.03753933127740	7.60390345198818	3.84621282975881
H	14.40640766419239	5.41637262190403	6.58553242383538
H	14.76077417612996	5.63573876046463	8.30377962714630
H	15.51456805288353	4.30470305465702	7.39812976547342
H	18.06339128620524	8.67006422613060	3.56778076106609
H	17.33006423929185	7.09726161023266	3.18048910632320
H	19.03514649451916	7.19556801269021	3.62102269403774
H	20.35714357904283	9.04746627296994	6.66159631635168
H	19.48813592948006	9.41419348501268	5.16711718455632
H	20.52219867770798	7.98776656469180	5.25146557848574
H	18.87787112450368	8.20900467203892	9.54383714570096
H	20.19109440663094	8.42296273308830	8.37610780848387
H	19.90532067620474	6.84202198278698	9.11648535765551
H	16.34169356643580	5.42936203807788	9.51804196347039
H	17.35362146256087	6.76113392346418	10.09843680741522
H	18.10366402916440	5.27638025597305	9.51316689377063
P	12.50254110666853	7.83263736477188	7.02388001535359

P	10.34915222877518	7.70829980224686	4.68475487584575
C	11.22507897228437	4.13645781003150	8.24258573573876
H	11.07700834832366	3.16082449288485	7.77299357500387
C	9.28619717577001	7.10561291065136	6.07358998711868
C	14.03569171610415	8.41299075519750	7.87665949157236
C	8.63044606047196	7.19578999019080	8.42680072324721
C	11.71111100087747	5.20000541711229	7.48672000809661
H	11.94810999210187	5.08628714516384	6.42381691676873
C	7.51882280079488	12.57235032505683	7.78285911985850
H	6.88797457582514	13.46054368989821	7.86099124732106
C	8.62509938702001	7.82138917790528	9.70233156821267
H	9.11950333453069	8.78627517404392	9.81879307616053
C	10.67879199671184	10.43133313172857	4.17007803397261
H	11.73892663961813	10.16032392311519	4.14029383795140
C	8.01975929676584	7.22457849949064	10.78318711317192
H	8.03382250095862	7.72305806953168	11.75552708550779
C	10.05234527840017	5.51014602009541	2.99810420712347
H	10.75885366172679	5.04820925734730	3.69314574633903
C	11.88038339315031	6.46169778786139	8.06753051532764
C	11.12778233880941	11.65505087746543	7.64098754634895
H	11.58608792521642	12.64433982562900	7.72029369868940
C	11.35098339214323	9.23963615631327	7.41899308204492
C	8.76466486515741	7.42783746551807	2.30865288514305
H	8.44756758962261	8.46140351614151	2.44964419868135
C	9.74798553958297	9.42641473186030	4.44367809513499
C	9.96561540317416	9.09471803084760	7.44612914441545
C	9.29641336314148	7.77075883985658	7.29663098399331
C	10.25582268503086	11.74429149710913	3.96749125348910
H	10.99037005695408	12.52634231411091	3.76427680895623
C	7.36337549858725	5.34239153201495	9.42498490339924
H	6.87243795976220	4.37363160431846	9.30578556194720
C	11.91418220048505	10.53813163418086	7.53740092316122
H	12.99845320322471	10.65042871373335	7.54429064361492
C	15.47765276536765	8.72696400819675	9.79877673824767
H	15.71620285195032	8.49971156945243	10.84072509110552
C	8.38653682652477	9.75237637469590	4.51096675405390
H	7.65293489420289	8.97845647243513	4.75169792272906
C	9.64299762397565	6.83323609107836	3.22067182647099
C	9.71765029710632	11.54896196253698	7.65911300960325
C	9.12728066027605	10.25247558552253	7.58453072120820
C	8.58177524068831	5.88176227330193	5.94732149635988
H	8.54481969768392	5.38344333927599	4.97823299370978
C	7.70987749983423	10.16404353740792	7.61930622415877
H	7.23499281146547	9.18437105127190	7.55918246462451
C	16.30310953639595	9.57278792308023	9.06190573993775
H	17.18187731503463	10.02481070890927	9.52688212501824
C	7.38452881771902	5.97014734396486	10.64745194276028
H	6.90911766766891	5.50355141196220	11.51326313931178
C	7.97537559281499	5.93547651448693	8.29128339841073
C	14.35421657989700	8.14616184786854	9.21185663889647
H	13.73719720992464	7.47043564174221	9.80543550161553

C	11.54449883491092	6.64296856977902	9.41421559543746
H	11.60269957758590	7.63657898275541	9.86392879685850
C	11.07607336452468	5.57523373536879	10.17391674572073
H	10.80083551134578	5.73639365876560	11.21824884481828
C	8.69347637016027	5.39428079562064	1.00300542619510
H	8.32031780650276	4.83377779917488	0.14269476596881
C	8.89983664877468	12.06035420525478	4.02797471275154
H	8.56539710565373	13.08825915807631	3.86870745806147
C	10.91431129097239	4.32023583771828	9.58952923507368
H	10.52733816769786	3.48838986734597	10.18230964078970
C	7.96479487637749	11.06156122895837	4.29706462135228
H	6.90254928231468	11.30939775426170	4.35869349646165
C	7.95308911176039	5.30902229530486	7.02318840079336
H	7.42494866298301	4.35926231761422	6.91251220697859
C	16.01376018393481	9.81770419881473	7.72196118222762
H	16.67360656935762	10.44830966219992	7.12246215681363
C	8.29263118766950	6.71230162095862	1.20842036641122
H	7.60938252529157	7.19449180649703	0.50484645888259
C	8.88742136939315	12.69408504691275	7.75089650418308
H	9.36177910827049	13.67735341739315	7.80390026558879
C	6.92872871036412	11.29144176842257	7.72266287072150
H	5.84074028773932	11.19243132278158	7.75441531773949
C	14.89233294754778	9.23713241512026	7.13663711720144
H	14.66532223781114	9.42472263149855	6.08841654501181
C	9.57914267047173	4.79621943928395	1.89991714506352
H	9.91357233483745	3.76908469457779	1.73534863957756
H	12.53735645058550	7.11251990379760	3.08201014211026

Species: ***Re*-INT0a**

E(PBE0-D3BJ/def2-SVP, def2-TZVP(Ir)) = -3293.696953425736

E(PBE0-D3BJ/def2-TZVPP)) = -3296.540767942845

H	11.87005358779953	5.51690592717194	4.92572310250132
H	9.46167764649681	3.31807756158985	1.56734287530703
C	9.49230374081337	4.40796193093310	1.49438424747753
H	14.24600532866579	9.23016171049942	6.66930935565044
C	14.29757085233458	8.80870684914766	7.67592370607827
H	5.97179875492650	11.91134904535096	5.63940950889761
C	7.02665192496705	11.84851752131422	5.91683115759738
H	9.69864020431698	13.85819955792499	6.57406970339646
C	9.10390911029432	12.95320871614844	6.42603544541778
H	9.04807900898235	6.93488511623984	-0.74206207578938
C	9.26336669369768	6.42843303452074	0.20219380909968
H	16.13237705537408	9.82553464458684	8.13224343763501
C	15.37129164573025	9.13385628171392	8.50000583214755
H	6.35356624449048	5.02554013784311	5.69493248480466
C	7.04507122231106	5.84855483459120	5.89439707261012
H	7.61068638156993	11.41197540578866	2.62797304460981
C	8.61189003084191	11.04670155256859	2.86792616488819
H	8.22931591776199	4.35251388827741	9.92588624709320
C	8.97005510949957	4.94176857380579	9.38016127235042

H	9.54817033031163	12.96466261022400	2.55336318172438
C	9.70015196868851	11.91920117960067	2.83196966582441
H	8.96188817248028	4.44478540036004	-0.60048684975851
C	9.21527562137780	5.03820592686763	0.28099168222407
H	8.99190247867677	6.54386717187599	10.83067173807461
C	9.39955981189545	6.16631706452925	9.89068784909052
H	10.60632643291287	7.91969183756056	9.55071174169398
C	10.30821176081811	6.93834669948507	9.17316373490309
H	12.71486875369716	6.57680931179328	9.70370604675912
C	13.45130018009993	7.30416308494411	9.36016527789377
C	6.90534071706103	6.59283814734608	7.08893213154803
H	5.01493234956480	6.78431038712385	9.93504122885058
C	5.77890390238675	7.03665120034502	9.19572381175716
H	16.34425766191574	8.78282922049485	10.39806626719883
C	15.49686895690998	8.53779868490922	9.75297403241370
H	7.02627151228619	9.71465097162685	5.90662021343541
C	7.61742985959068	10.61584322668856	6.06688484753667
H	8.08262602253137	5.58053899118210	4.06321523511405
C	8.02399155906145	6.15757920734977	4.98401740564082
C	8.98930798799685	10.49369127071275	6.41663096887548
C	9.73580021081366	11.69556926059552	6.59699443339249
C	9.85893424839411	6.56379859497095	2.55077397777191
H	7.94879285088069	9.03401184551790	3.26733317891435
C	8.80322317052799	9.71523057164378	3.22218111429094
H	14.64467816907155	7.11918340259255	11.14222071022272
C	14.54247006642326	7.61060045624075	10.17196413156394
H	12.72933242205854	10.33933145804048	7.44214035331949
C	11.69066740940277	10.37176805899598	7.12605698809048
H	5.26708180223530	5.41662783559183	7.88079460600922
C	5.91801442528047	6.27626394424945	8.05861738808850
H	11.82931638333233	12.13754884532288	3.11475028688587
C	10.97584709617302	11.45674564294909	3.14785901489351
C	8.80053046804814	8.00494960902503	6.34809301887229
C	9.63631880157142	9.21907777188545	6.57290841464415
C	10.08463372617577	9.25066503564484	3.54848106796935
H	9.59409529902509	8.27625814974236	1.25648678795480
C	9.57274558009527	7.18750009193714	1.33067268954934
C	10.98404685032688	9.15806871718869	6.92507618693970
H	11.67095712677125	12.50765856417305	7.13604805964657
C	11.09655479349536	11.59449640486704	6.96228445057460
C	10.81725244656222	6.47993277067084	7.95133238869368
H	10.09859810644477	4.65878348335385	3.54473737005321
C	9.83249840971285	5.16226075661099	2.61235031013298
H	6.51437935504053	8.75001827063089	10.31094242848243
C	6.62712522159658	8.14680474621924	9.40737930433380
C	11.17419114794892	10.12572065443351	3.51301353846294
H	8.26175463318256	9.31634753791369	8.67159909389652
C	7.59956563313196	8.46865987863453	8.48985351528290
H	7.29083084800793	14.00490333228819	5.96294906818141
C	7.77307706271671	13.03314707348672	6.09384713506327
H	10.80836253947803	4.88857033372623	6.50795991015676

C	10.40995171580507	5.23563719647782	7.46332618540590
C	7.77657181688164	7.70162303760978	7.30617487216869
C	13.30483257557747	7.91544905269795	8.10734865009621
C	8.94309975259113	7.21640337486898	5.20726657142190
H	9.13205294259868	3.52547259767964	7.75646756896539
C	9.47959985990982	4.47578982693289	8.16902846513880
P	10.35792528796489	7.50982878875337	4.04265362879253
P	11.92170816750538	7.54556746230913	6.95180915981371
H	19.08090588352401	11.16432081573161	5.90931743823294
H	19.36467031909906	10.53811329860363	4.28000572731772
H	17.88571184802968	11.44392109641743	4.63927117209268
H	19.54297063672942	9.73505720267799	7.94027986198476
H	20.18132383488676	8.12654780675529	7.58135373123059
H	20.39167269362844	9.43965950772255	6.41570663967846
H	17.44140844272532	6.02054989160129	8.47699169065638
H	19.07473660322888	6.10950020592473	7.80075884577443
H	18.49234833814561	7.37432108014102	8.89662542616523
H	15.47157412737510	6.10428529799448	7.89473979985315
H	14.70804178614057	5.85332356604890	6.32159040601460
H	16.24196244039050	5.03019285686244	6.71591981124359
H	15.53236105823053	10.62168766938041	3.82441078366640
H	17.08566045950828	10.27607412149774	3.02348291927705
H	15.70960217110877	9.17254763446164	2.84828606826755
C	15.67696901736448	5.97092395324877	6.82270382994207
C	18.18943622699024	6.71007757771516	8.07316160758213
C	19.68621403498020	8.99622216574616	7.13130739800847
C	18.58533598031826	10.70215104013000	5.04484108610553
C	16.23953784488749	9.82185775284002	3.55606418997883
C	16.45366163384214	7.13994330174795	6.28187397917780
C	17.66826300180713	7.49791811042479	6.90143563450746
C	18.38194598475840	8.62824447205373	6.47129972528298
C	17.87579009555621	9.42828314887475	5.42971357225203
C	16.69981327886198	9.04603922744216	4.76393937549493
C	15.97545547531219	7.91721377944214	5.21119042828175
O	13.82741692747573	8.68066490197636	4.46607607796204
H	12.22992809944940	7.83070615762398	1.36565823797093
H	13.52652094998438	8.67446408637372	2.25036684847982
H	13.92334767973662	7.49929777899116	0.96696705748997
C	14.67297407151024	7.68796875703789	4.49783269115210
H	16.26817036922169	5.83941855729126	3.02512349161701
H	15.90567851752120	5.20286585043405	4.61067457183804
H	14.32226225081251	3.58083227247921	3.69887813230324
H	15.82502068895635	3.48991086513085	2.76798480515714
H	13.84874765646591	3.51369345955639	1.22355030234877
H	14.78192150640408	5.00447593968063	1.02153100566125
C	14.96034699454418	4.13657515528944	2.98884032978078
H	12.32823348106838	4.61911861894759	2.80268098528236
H	12.29068294913889	5.43933495995863	1.24793962754040
C	14.15565236472206	4.44060488634660	1.73634547615880
C	12.92499007916606	5.24763762563269	2.12628347407589
C	13.23344913185755	7.71815817741950	1.80269468296033

C	15.44878025932172	5.42152179894821	3.64062621904152
C	13.26156182730220	6.57579466208549	2.80426324508979
C	14.41725397118212	6.52262038975120	3.72417809788474
Ir	12.44219902737315	6.97612893574889	4.73462962561356
H	12.16714305545806	9.76334126030012	3.79148123981147

Species: ***Re*-INT0b**
E(PBE0-D3BJ/def2-SVP, def2-TZVP(Ir)) = -3293.664854025692
E(PBE0-D3BJ/def2-TZVPP)) = -3296.503493601328

Ir	12.34056025248887	7.85607659042135	5.06148577479799
C	13.45470108151327	8.27066747577261	3.21286435420351
C	14.27319382902976	8.20812017643702	4.46491909590014
C	13.43046730194480	9.59154757012561	2.43208855212786
C	15.49718139279714	7.33012890570616	4.54262392842481
C	14.40626895947426	9.56977659440306	5.13966780997917
C	14.80527877928481	10.70318023499385	4.21024501863313
H	15.05493889343510	9.47392446831280	6.02342789699854
H	13.40448267160767	9.88015238964335	5.55125022409031
C	13.68041794760097	10.87752047095304	3.20919889793406
H	15.74769616056672	10.45006712980081	3.69298569767522
H	14.98352865269724	11.63124616356415	4.77819885041412
H	13.89958991858159	11.68709595227145	2.49516064764973
H	12.77398813349221	11.18697871796445	3.75989553080160
H	12.49655156995186	9.68839831290937	1.85932043403560
H	14.22009748270996	9.52106237272513	1.66211645357959
C	13.49911124153657	7.08068028843316	2.31120048396763
H	15.88242847281739	7.25901868354339	5.56934319849466
H	16.31598724850311	7.74833246807752	3.92903159205181
H	15.27966808837765	6.31644485693024	4.18387780592093
O	13.50966155003300	5.92414121799845	2.70643874843643
C	13.60454162100754	7.31098264336231	0.80709186891711
C	14.85459523787289	7.13065032542638	0.18556888748568
C	14.96696705032091	7.32574081356820	-1.20267026097340
C	13.82852470571946	7.63256088496948	-1.96916190638541
C	12.58880382834569	7.83451109480447	-1.33953398688268
C	12.47755928888419	7.67722039762103	0.05451611972863
C	16.06990437951239	6.74990860920500	0.98828995262324
C	16.29973548005826	7.19958248277799	-1.89168849837470
C	13.95504470234530	7.73196336831229	-3.46531696345530
C	11.37698091395778	8.21206705888800	-2.15035853024028
C	11.15154492602235	7.89327942074860	0.72493490892362
H	16.65062093561706	5.96222482716965	0.48754329254379
H	16.74671808912817	7.60955274789861	1.13244509022594
H	15.79541291890630	6.36798399425792	1.97507768678995
H	10.75629145403108	8.90407048492490	0.53086552059587
H	11.22585890478448	7.77337100116457	1.81031889473056
H	10.39127433646047	7.17886932756957	0.37382904309064
H	11.63020939716391	8.89069086531265	-2.97617439513501
H	10.61678946560825	8.71271591610059	-1.53874846851424
H	10.89293056173086	7.32499641872809	-2.59648842201149
H	14.65352251373987	6.98104703232493	-3.85991476039364

H	14.33716876373640	8.71768655117883	-3.78597581666432
H	12.99495794400422	7.57464017704404	-3.97153814836774
H	17.13635673207192	7.22236089994626	-1.18368803142815
H	16.46133200859265	8.01679135001759	-2.61115696822324
H	16.38053519368175	6.25778056371099	-2.46280522080893
P	12.08386783724675	7.68179444161308	7.32562497263880
P	10.00832759825528	7.83537625115999	4.78826271403308
C	10.74485017952944	3.86408132827956	8.01081084172792
H	10.61335130209160	2.95762618006564	7.41536830788357
C	8.94309330737421	7.19821643201912	6.17411345185143
C	13.53592058639259	7.95565007070568	8.42052674557895
C	8.26203694810548	7.17717480405896	8.52728159395127
C	11.20739016974670	5.02971480718523	7.40037372897871
H	11.44259562255739	5.04416464465585	6.33377593281107
C	7.42404304084032	12.71744003600200	7.93833022820532
H	6.85017362602940	13.64514222541530	7.99399878686866
C	8.28194275181958	7.73356540836086	9.83349182249987
H	8.80229621162139	8.67879995361507	9.99588389666335
C	9.96845653520127	10.31710630622852	3.56522497967321
H	10.98697569396009	10.04165565496789	3.28660887237776
C	7.67304794268108	7.09012023888107	10.88487007847293
H	7.70405325851259	7.53318555865673	11.88323729034255
C	10.19728189480104	5.64007707792407	3.05751880450836
H	11.15016160186425	5.45561425183451	3.55822246736171
C	11.37760896681490	6.19812205481856	8.14719214384722
C	10.97008994483599	11.57038227963446	7.81120470078752
H	11.49035084564306	12.53107537889740	7.84174943571216
C	11.02642649944936	9.13942423574166	7.70521925321076
C	8.25497379782086	7.02755762584907	2.69965068282078
H	7.65558488251242	7.90509794768501	2.94933520543127
C	9.22303406628662	9.43754027764556	4.35799261490482
C	9.63919781149111	9.09219911511272	7.65967964733834
C	8.93262510409537	7.80192401674865	7.43109688214517
C	9.41444803137212	11.51619875930961	3.12494330056672
H	10.00565584271971	12.18960660364192	2.49911237068508
C	6.97258347982303	5.29410909786342	9.43221901828542
H	6.46687028094205	4.34001001943528	9.26579029405059
C	11.68218368523907	10.40087097410530	7.77036696597137
H	12.77355251305705	10.43455456027993	7.77418615007268
C	15.89087126863499	7.51753417664157	8.80087958870328
H	16.82673556656036	7.06384962912597	8.46591302528846
C	7.91805688485803	9.78682863434167	4.71947949014192
H	7.32721221153364	9.10988824739984	5.34061835208873
C	9.43274728682864	6.75963676005548	3.40733663446075
C	9.55372284138952	11.55580489642493	7.82975630456760
C	8.87744891418876	10.29989699522000	7.78051641237326
C	8.23761798033369	5.97903378843950	5.99475832505986
H	8.22389451864474	5.51032165823348	5.01087298573690
C	7.45897181967373	10.29854916280586	7.84491715315294
H	6.92991346538087	9.34485199416651	7.83331846719482
C	15.84098781289358	8.24820735574819	9.98726341220928

H	16.74174307362898	8.38402602523569	10.59067912767489
C	7.00932507273286	5.85883202038966	10.68473684928516
H	6.52696733297338	5.35811446936312	11.52702942892857
C	7.59461940549356	5.93229405699889	8.32964105950466
C	14.74323332327908	7.37497626123278	8.02467728275682
H	14.77273654994789	6.82166832933017	7.08488999837648
C	11.09457938050149	6.18108003966965	9.51913839976582
H	11.23151013687466	7.08168738692205	10.11988962145654
C	10.62295345714547	5.02162057762155	10.12404355089047
H	10.38021457816445	5.02627563953495	11.18870561096781
C	8.60378995256572	5.06419285854252	1.33336751426405
H	8.28474046172597	4.40733680136839	0.52037170008233
C	8.11407788965639	11.86017735444460	3.49398336561062
H	7.67842320369025	12.80303146489181	3.15482949836055
C	10.44615048346109	3.86016095365483	9.37127953766728
H	10.07164178202811	2.95250258365809	9.85100099736782
C	7.37073189866733	10.99734667924305	4.29767061527483
H	6.35947489665662	11.27170205167562	4.60557017474560
C	7.58850420861964	5.36492787722137	7.03514560955069
H	7.06513451031819	4.41976306158814	6.87348425133725
C	14.63440114634837	8.81031005812766	10.40411218211748
H	14.58618265038620	9.37439401822345	11.33848933315772
C	7.84510515964186	6.18633240169630	1.66766680834852
H	6.93000233192719	6.41553498603970	1.11667364448956
C	8.79761708392674	12.75297977486981	7.89473985974189
H	9.33116637545945	13.70667921871550	7.91776230037092
C	6.75241115204445	11.47545975132164	7.92678375812849
H	5.66140095855931	11.44924685452530	7.98373209544327
C	13.48522329719905	8.66094941961049	9.63033573478909
H	12.55053382758985	9.11613203121101	9.96749872848498
C	9.78113467443195	4.79527840778462	2.02971250289725
H	10.40029654865092	3.93882503046225	1.75514444503882
H	12.43395978230176	6.32596933980460	4.91961819597420

6. *References*

[1] A. B. Pangborn, M. A. Gairdello, R. H. Grubbs, R. K. Rosen, F. J. Timmers, *Organometallics*, **1996**, *15*, 1518–1520.
[2] *Purification of Laboratory Chemicals*, 3rd edition. D.D. Perrin, W. L. F. Armarego, *Pergamon Press*, Oxford, **1988**.
[3] W. M. Akhtar, R. J. Armstrong, J. R. Frost, N. G. Stevenson, T. J. Donohoe, *J. Am. Chem. Soc.* **2018**, *140*, 11916–11920.
[4] P. Kiprof, J. Li, C. L. Renish, E. K. Kalombo, V. G. Young, *J. Organomet. Chem.* **2001**, *620*, 113–118.
[5] H. Suzuki, M. Hashihama, T. Mishina, *Bull. Chem. Soc. Jpn.* **1981**, *54*, 1186–1190.
[6] (a) K. Mitachi, T. Yamamoto, F. Kondo, T. Shimizu, M. Miyashita, K. Tanino, *Chem. Lett.* **2010**, *39*, 630–632; (b) D. M. Bailey, R. E. Johnson, *J. Org. Chem.* **1970**, *35*, 3574–3576.
[7] X. Xie, S. S. Stahl, *J. Am. Chem. Soc.* **2015**, *137*, 3767–3770.
[8] J. Pícha, V. Vaněk, M. Buděšínský, J. Mládková, T. A. Garrow, J. Jiráček, *Eur. J. Med. Chem.* **2013**, *65*, 256–275.
[9] F. Boratyński, G. Kiełbowicz, C. Wawrzeńczyk, *J. Mol. Catal. B Enzym.* **2010**, *65*, 30–36.
[10] I. R. Hazelden, R. C. Carmona, T. Langer, P. G. Pringle, J. F. Bower, *Angew. Chem. Int. Ed.* **2018**, *57*, 5124–5128.
[11] B. D. Schwartz, C. S. P. McErlean, M. T. Fletcher, B. E. Mazomenos, M. A. Konstantopoulou, W. Kitching, J. J. De Voss, *Org. Lett.* **2005**, *7*, 1173–1176.
[12] X.-H. Yang, K. Wang, S.-F. Zhu, J.-H. Xie, Q.-L. Zhou, *J. Am. Chem. Soc.* **2014**, *136*, 17426–17429.
[13] F. Foubelo, S. A. Saleh, M. Yus, *J. Org. Chem.* **2000**, *65*, 3478–3483.
[14] J. Mao, F. Liu, M. Wang, L. Wu, B. Zheng, S. Liu, J. Zhong, Q. Bian, P. J. Walsh, *J. Am. Chem. Soc.* **2014**, *136*, 17662–17668.
[15] O. Linnenberg, A. Kondinski, C. Stöcker, K. Y. Monakhov, *Dalton Trans.* **2017**, *46*, 15636–15640.
[16] R. Gniłka, A. Szumny, A. Białońska, C. Wawrzeńczyk, *Phytochem. Lett.* **2012**, *5*, 340–345.
[17] H. C. Brown, T. Imai, M. C. Desai, B. Singaram, *J. Am. Chem. Soc.* **1985**, *107*, 4980–4983.
[18] M. J. Schultz, S. S. Hamilton, D. R. Jensen, M. S. Sigman, *J. Org. Chem.* **2005**, *70*, 3343–3352.
[19] F. Neese, *WIREs Comput. Mol. Sci.* **2018**, *8*, e1327.
[20] C. Adamo, V. Barone, *J. Chem. Phys.* **1999**, *110*, 6158–6170.
[21] (a) S. Grimme, S. Ehrlich, L. Goerigk, *J. Comput. Chem.* **2011**, *32*, 1456–1465; (b) S. Grimme, J. Antony, S. Ehrlich, H. Krieg, *J. Chem. Phys.* **2010**, *132*, 154104.
[22] D. Andrae, U. Häußermann, M. Dolg, H. Stoll, H. Preuß, *Theoret. Chim. Acta* **1990**, *77*, 123–141.
[23] F. Weigend, R. Ahlrichs, *Phys. Chem. Chem. Phys.* **2005**, *7*, 3297–3305.
[24] F. Weigend, *Phys. Chem. Chem. Phys.* **2006**, *8*, 1057–1065.
[25] A. V. Marenich, C. J. Cramer, D. G. Truhlar, *J. Phys. Chem. B* **2009**, *113*, 6378–6396.
[26] E. P. K. Olsen, T. Singh, P. Harris, P. G. Andersson, R. Madsen, *J. Am. Chem. Soc.* **2015**, *137*, 834–842.
[27] (a) H.-L. Qin, X.-Q. Chen, Y.-Z. Huang, E. A. B. Kantchev, *Chem. Eur. J.* **2014**, *20*, 12982–12987; (b) T. Korenaga, R. Maenishi, K. Hayashi, T. Sakai, *Adv. Synth. Catal.* **2010**, *352*, 3247–3254.

7. NMR Spectra

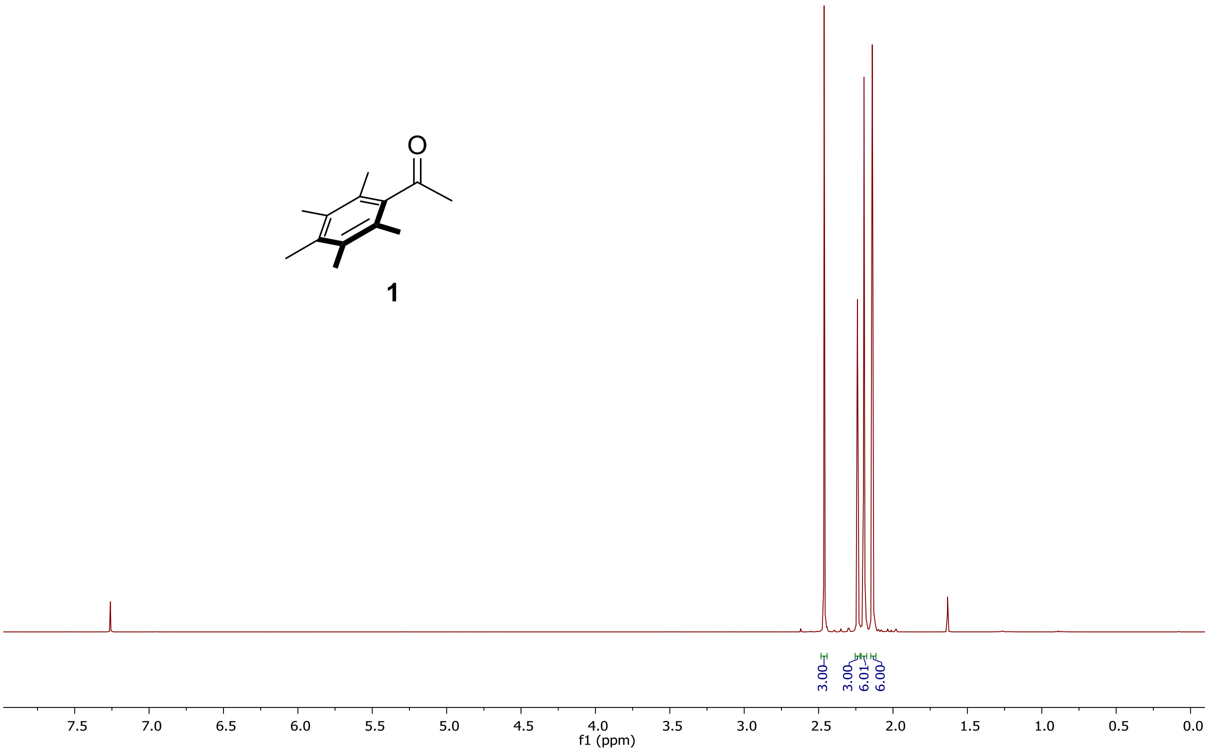

1

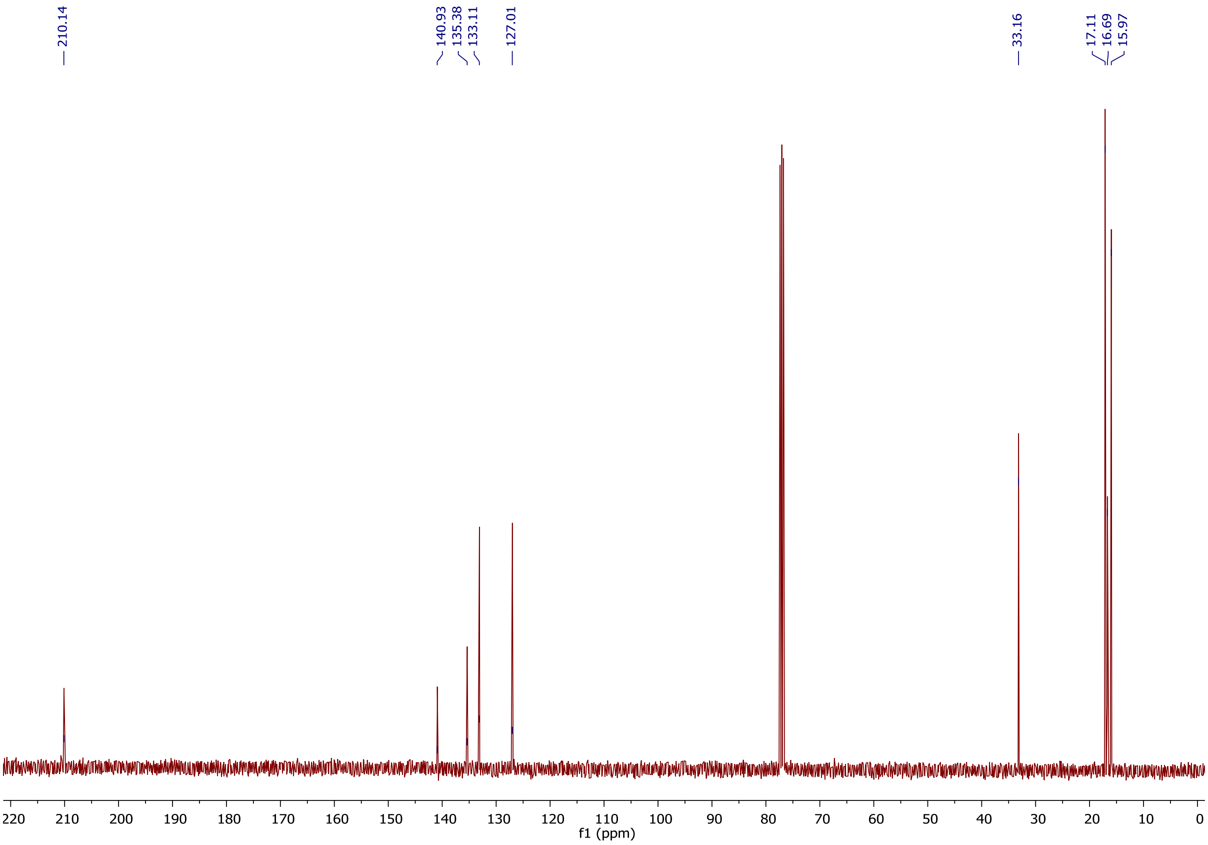

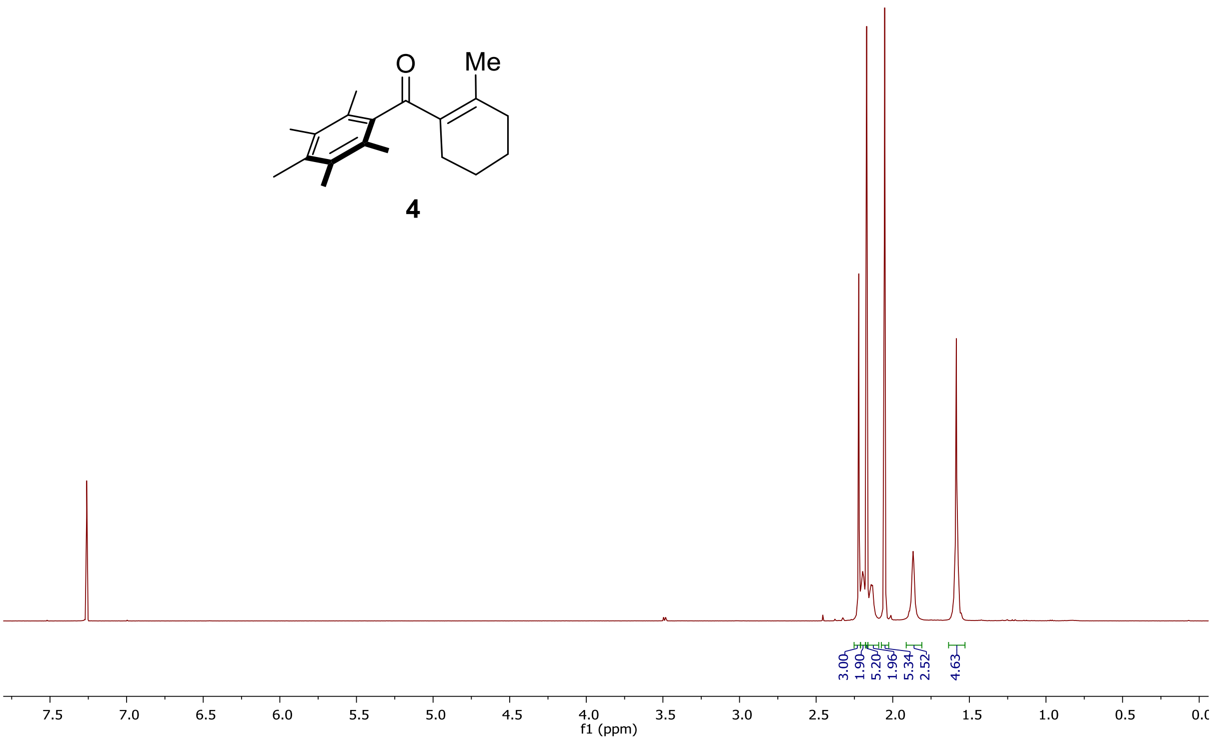

O
Me
4
3.00
1.90
5.20
1.96
5.34
2.52
4.63
7.5
7.0
6.5
6.0
5.5
5.0
4.5
4.0
3.5
3.0
2.5
2.0
1.5
1.0
0.5
f1 (ppm)

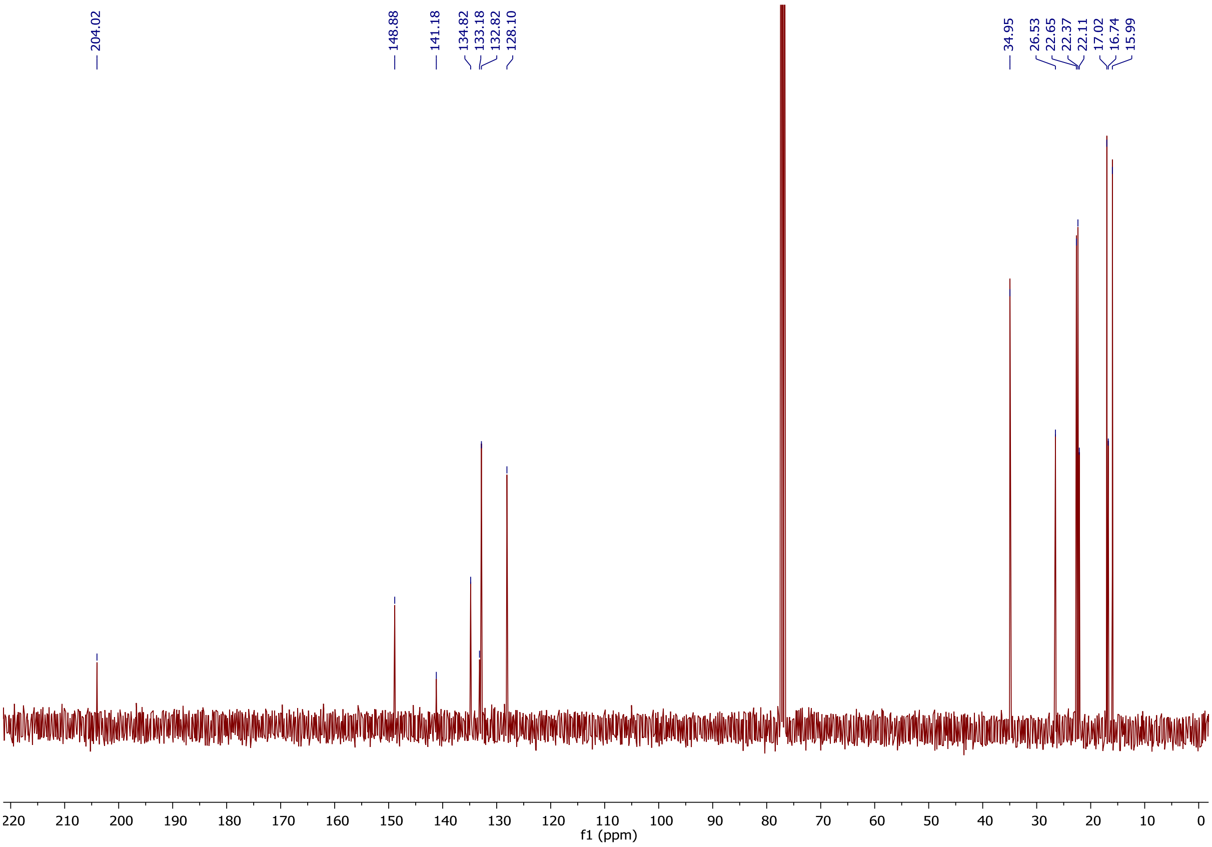

204.02
148.88
141.18
134.82
133.18
132.82
128.10
34.95
26.53
22.65
22.37
22.11
17.02
16.74
15.99
220
210
200
190
180
170
160
150
140
130
120
110
100
90
80
70
60
50
40
30
20
10
0
f1 (ppm)

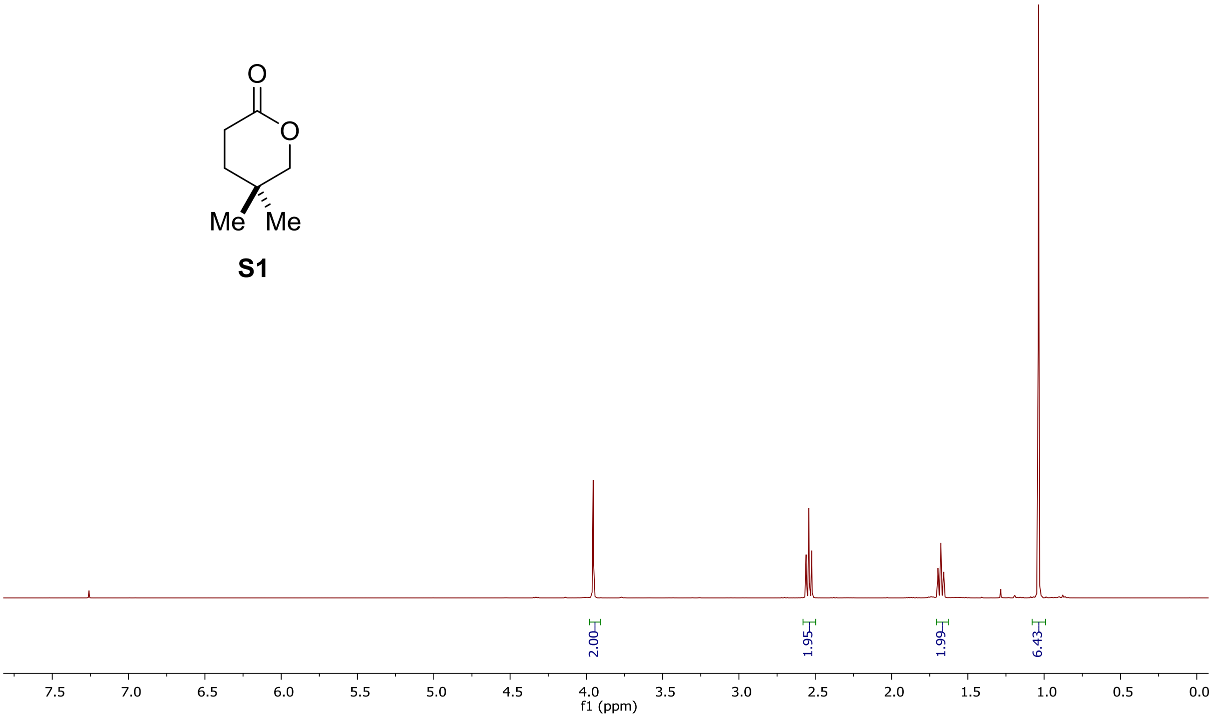
O
O
Me
Me
S1
2.00
1.95
1.99
6.43
7.5
7.0
6.5
6.0
5.5
5.0
4.5
4.0
3.5
3.0
2.5
2.0
1.5
1.0
0.5
0.0
f1 (ppm)

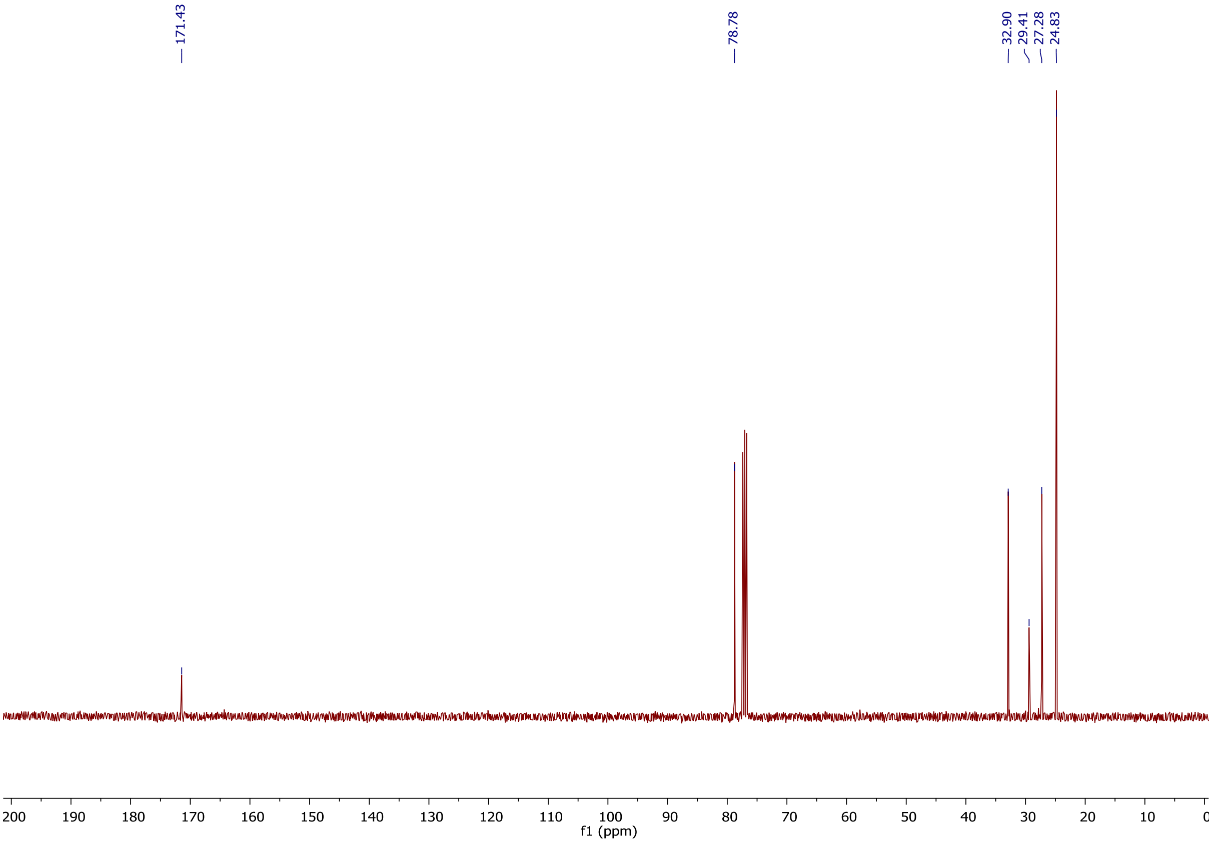
171.43
78.78
32.90
29.41
27.28
24.83
200
190
180
170
160
150
140
130
120
110
100
90
80
70
60
50
40
30
20
10
f1 (ppm)

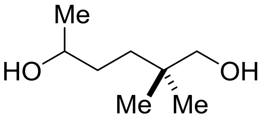
Me
HO
OH
Me
Me

2b

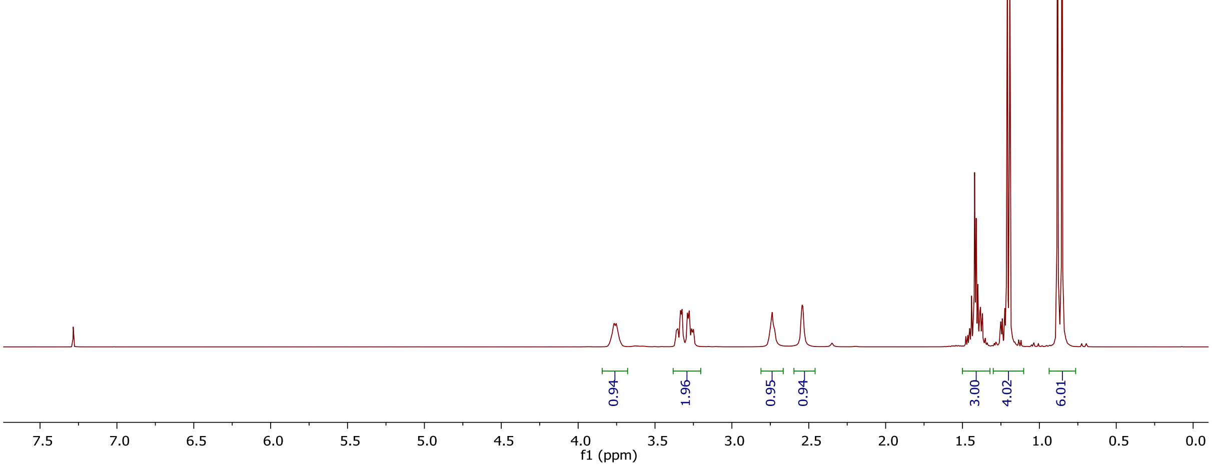
0.94
1.96
0.95
0.94
3.00
4.02
6.01
7.5 7.0 6.5 6.0 5.5 5.0 4.5 4.0 3.5 3.0 2.5 2.0 1.5 1.0 0.5 0.0
f1 (ppm)

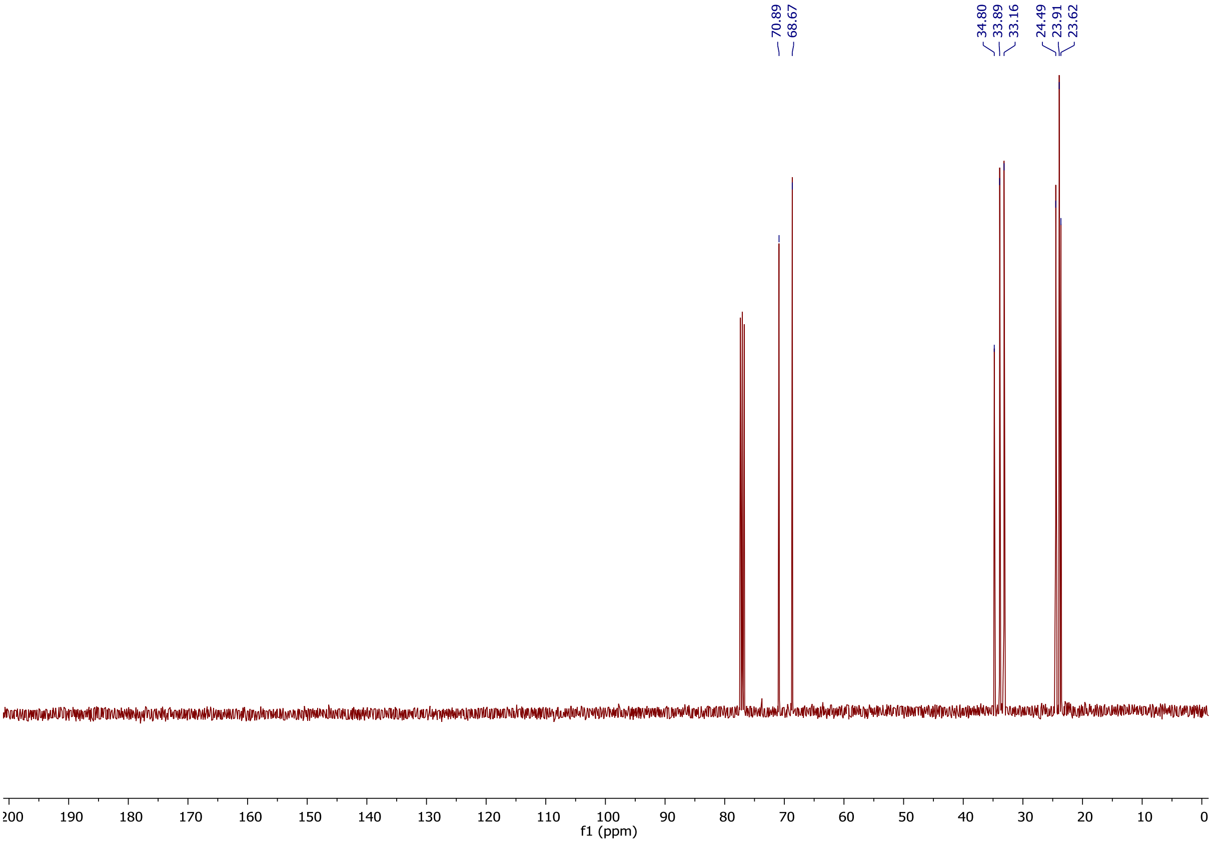
70.89
68.67
34.80
33.89
33.16
24.49
23.91
23.62
200 190 180 170 160 150 140 130 120 110 100 90 80 70 60 50 40 30 20 10 0
f1 (ppm)

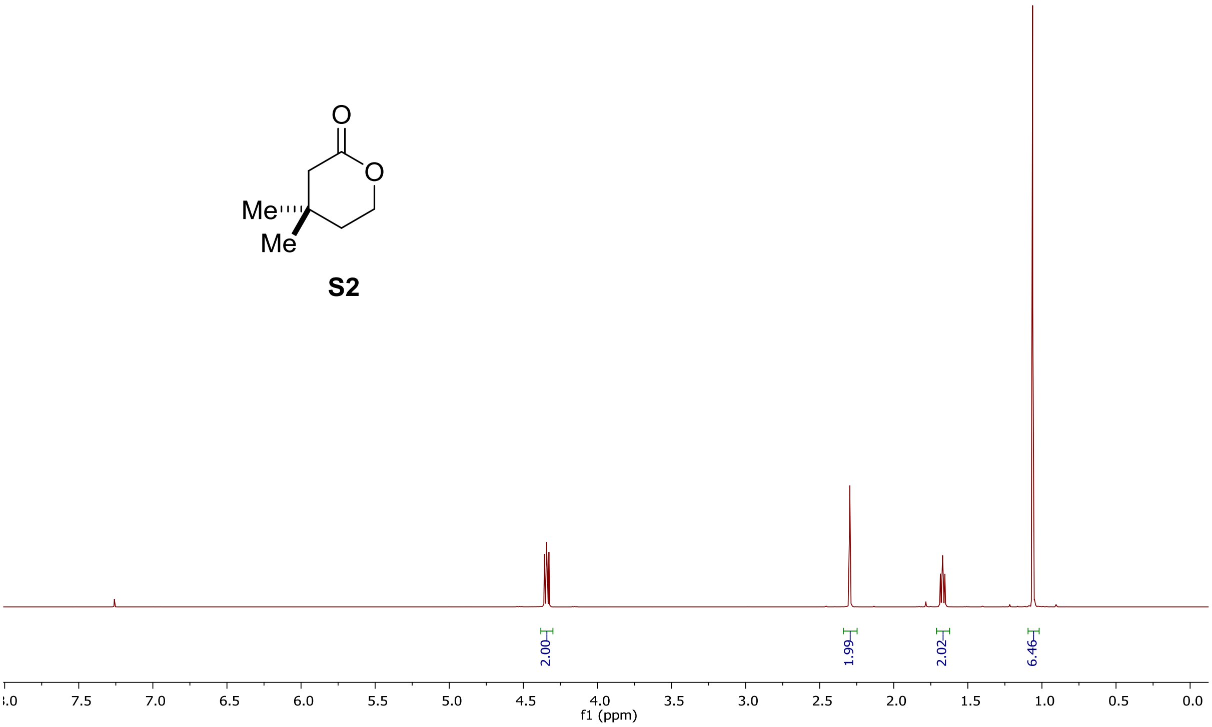

Me
Me
O
O
S2
2.00
1.99
2.02
6.46
7.5
7.0
6.5
6.0
5.5
5.0
4.5
4.0
3.5
3.0
2.5
2.0
1.5
1.0
0.5
0.0
f1 (ppm)

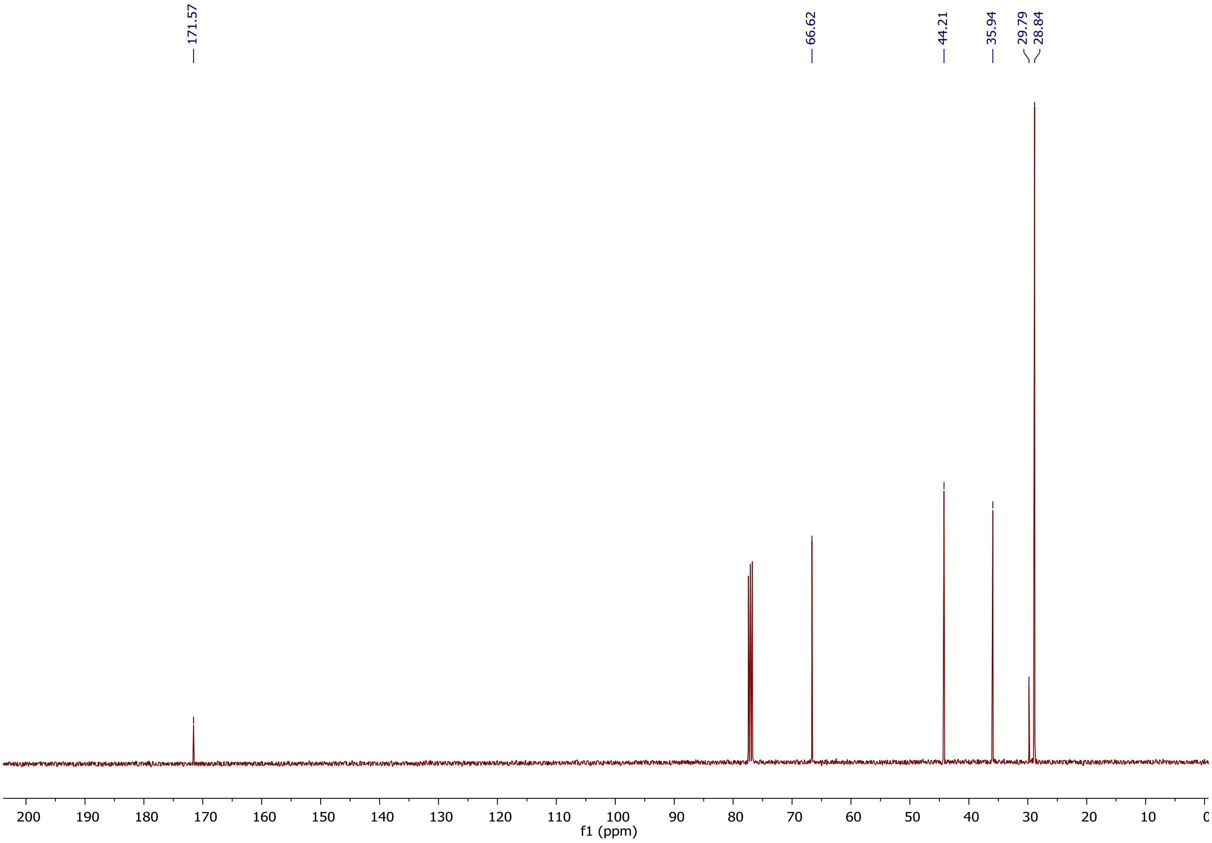

171.57
66.62
44.21
35.94
29.79
28.84
200
190
180
170
160
150
140
130
120
110
100
90
80
70
60
50
40
30
20
10
f1 (ppm)

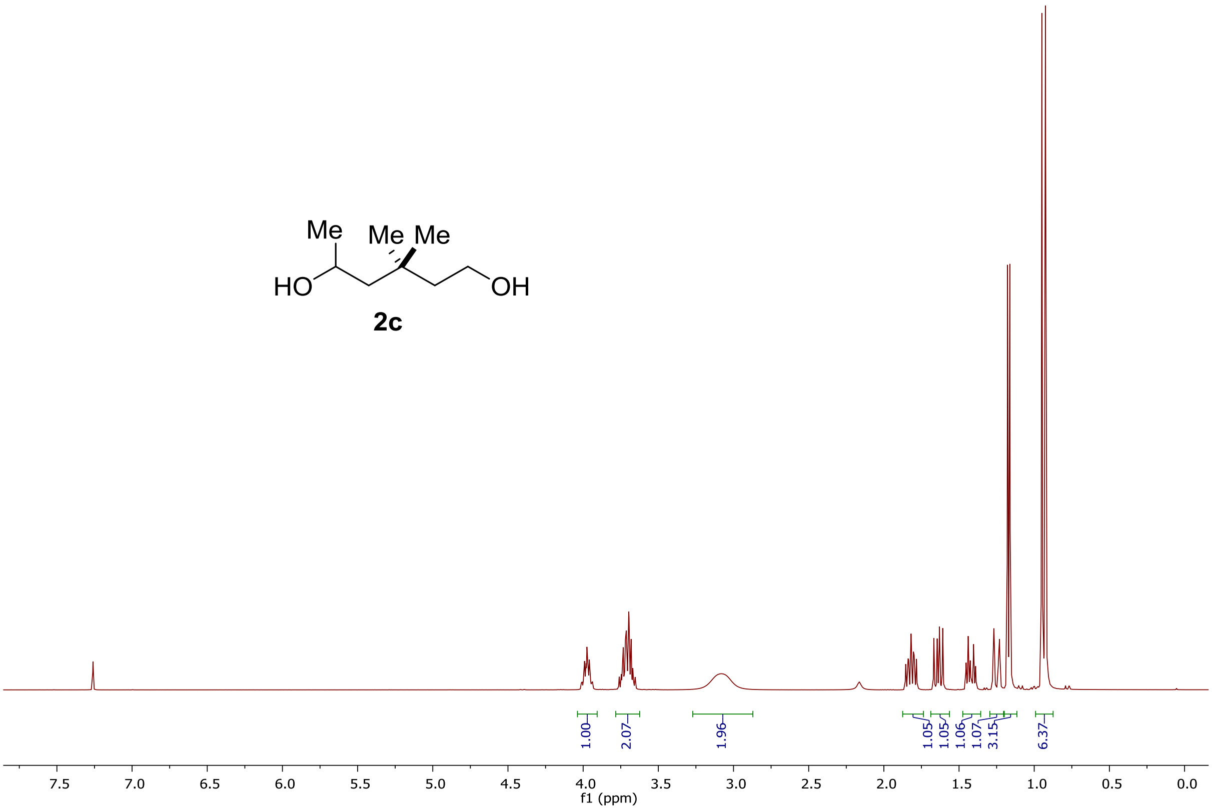
Me
Me Me
HO
OH
2c
1.00
2.07
1.96
1.05
1.05
1.06
1.07
3.15
6.37
7.5 7.0 6.5 6.0 5.5 5.0 4.5 4.0 3.5 3.0 2.5 2.0 1.5 1.0 0.5 0.0
f1 (ppm)

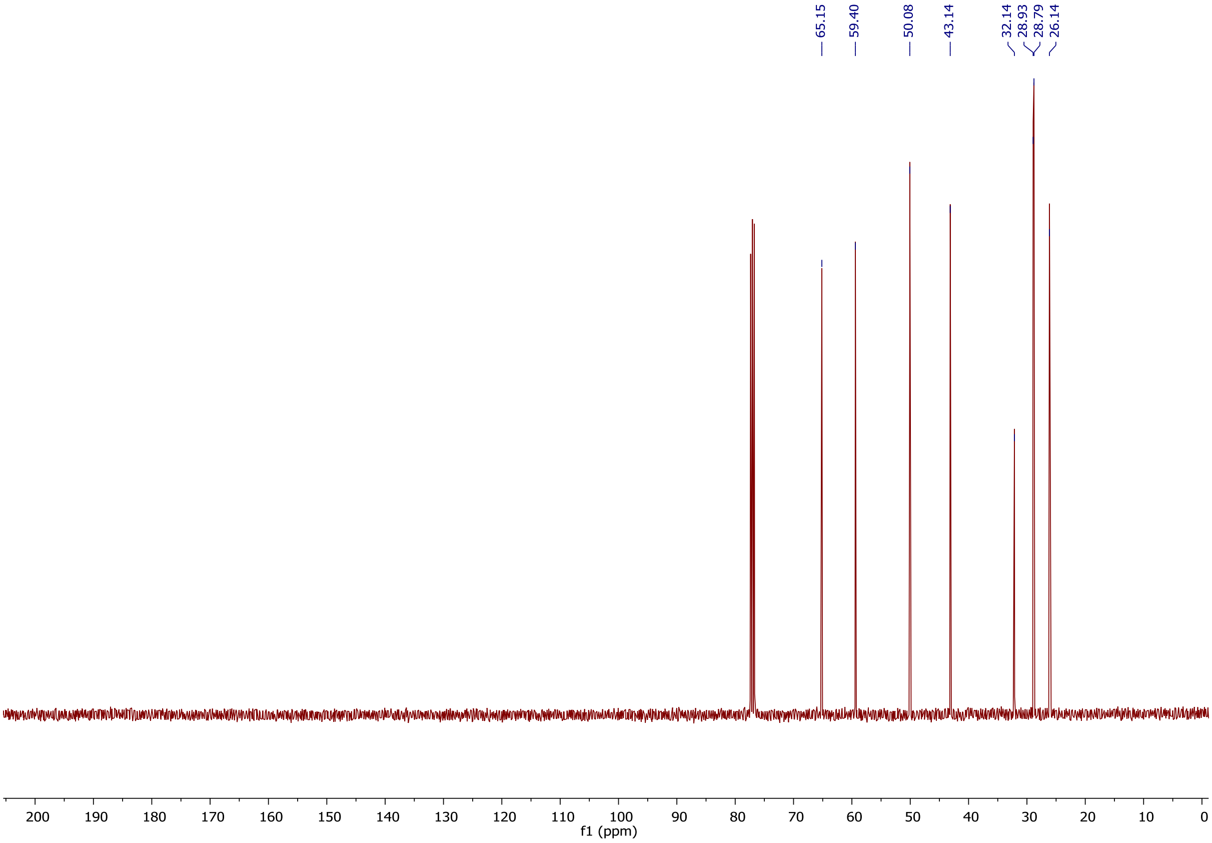
65.15
59.40
50.08
43.14
32.14
28.93
28.79
26.14
200 190 180 170 160 150 140 130 120 110 100 90 80 70 60 50 40 30 20 10 0
f1 (ppm)

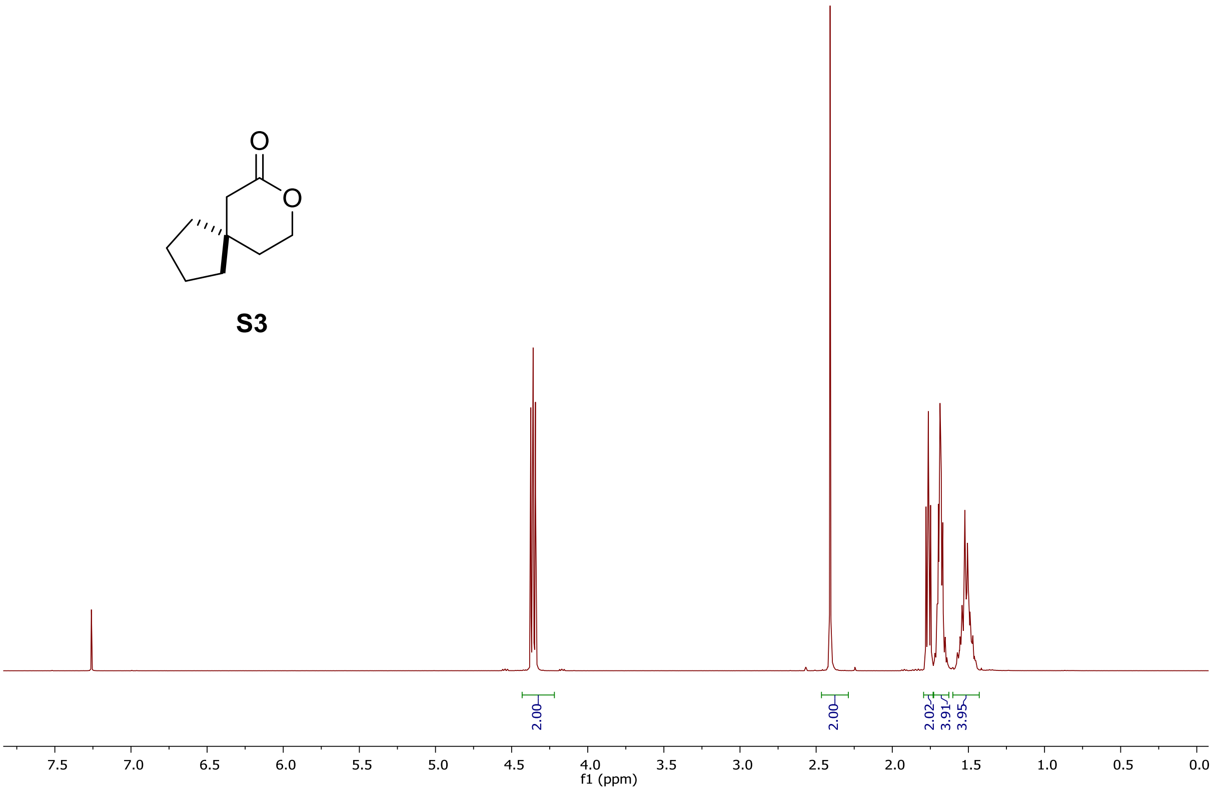

S3
2.00
2.00
2.02
3.91
3.95
7.5 7.0 6.5 6.0 5.5 5.0 4.5 4.0 3.5 3.0 2.5 2.0 1.5 1.0 0.5 0.0
f1 (ppm)

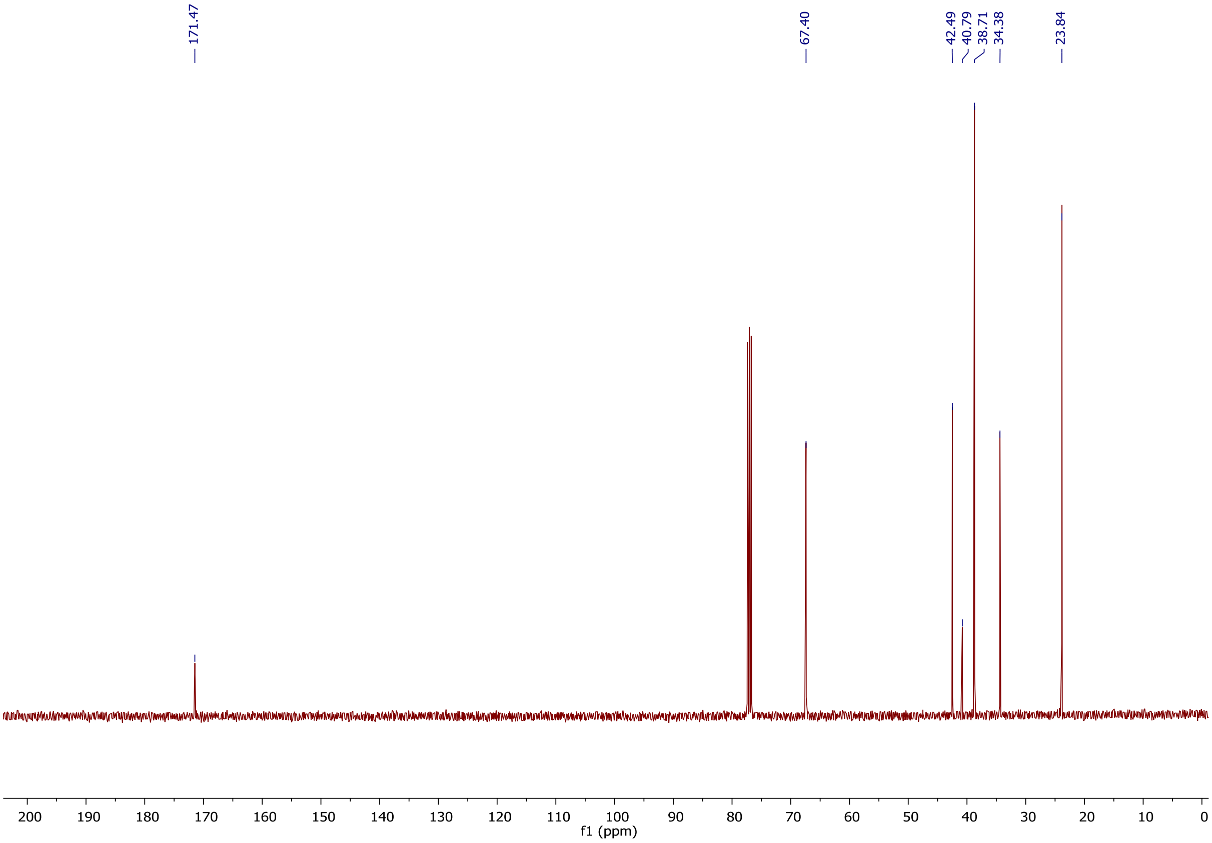

171.47
67.40
42.49
40.79
38.71
34.38
23.84
200 190 180 170 160 150 140 130 120 110 100 90 80 70 60 50 40 30 20 10 0
f1 (ppm)

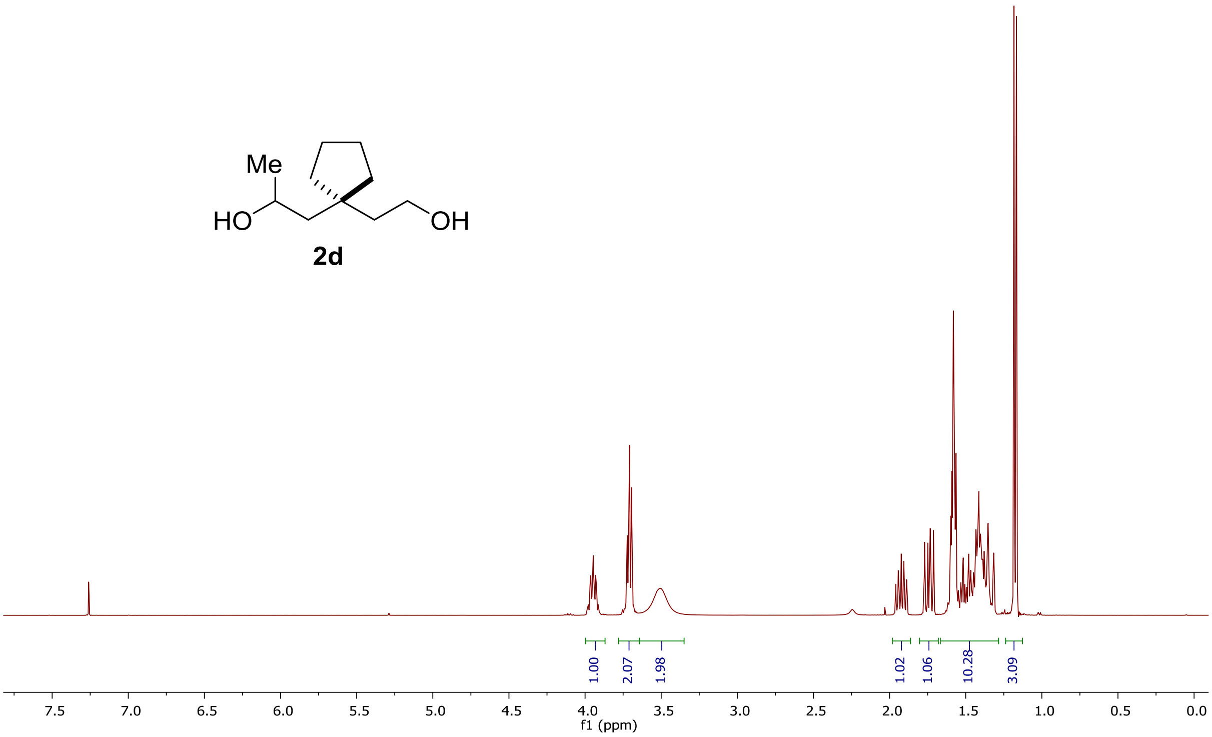
Me
HO
OH
2d
1.00
2.07
1.98
1.02
1.06
10.28
3.09
7.5 7.0 6.5 6.0 5.5 5.0 4.5 4.0 3.5 3.0 2.5 2.0 1.5 1.0 0.5 0.0
f1 (ppm)

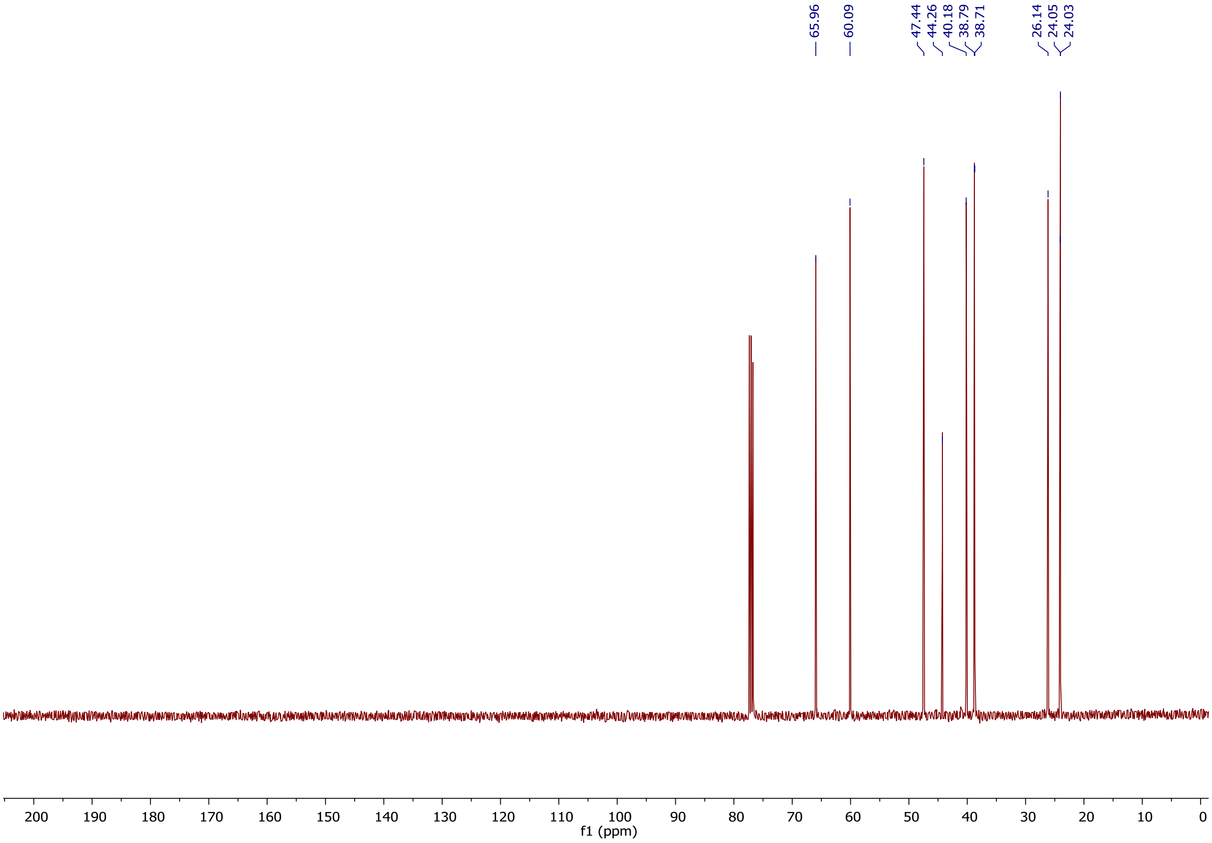
65.96
60.09
47.44
44.26
40.18
38.79
38.71
26.14
24.05
24.03
200 190 180 170 160 150 140 130 120 110 100 90 80 70 60 50 40 30 20 10 0
f1 (ppm)

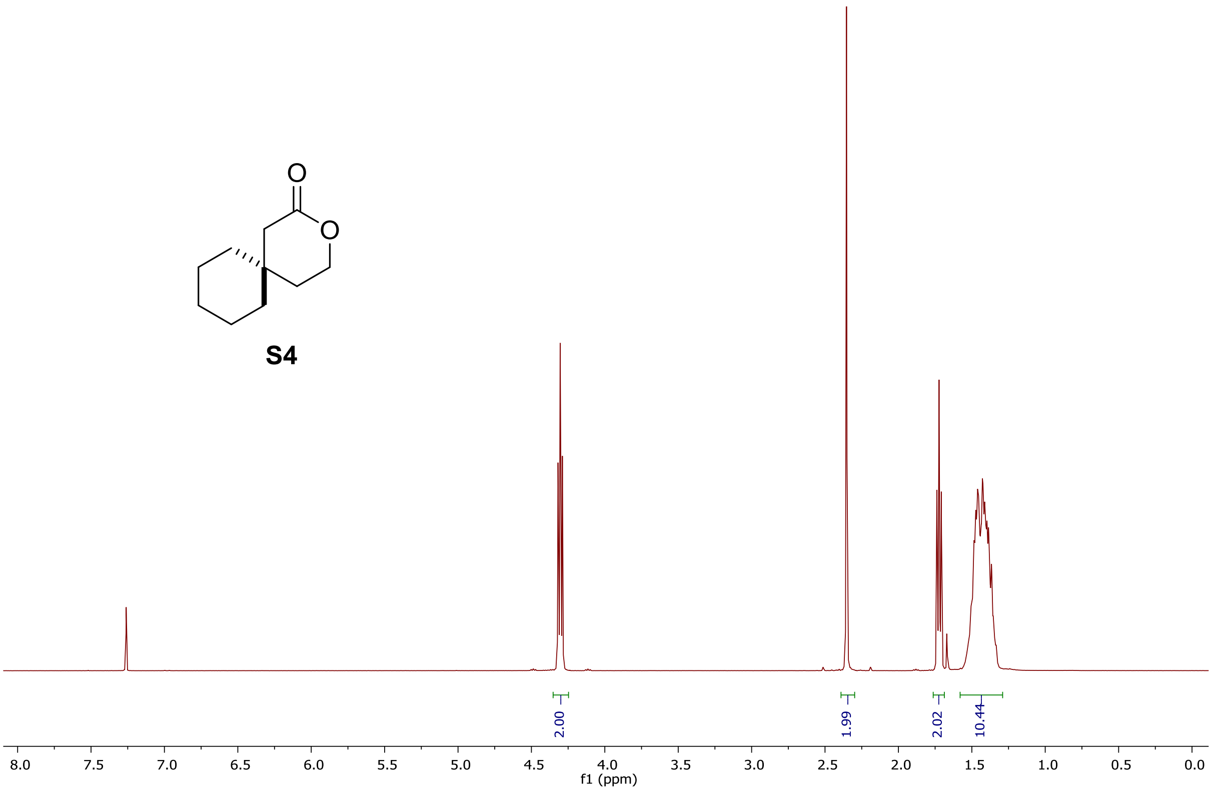

S4

2.00
1.99
2.02
10.44
8.0 7.5 7.0 6.5 6.0 5.5 5.0 4.5 4.0 3.5 3.0 2.5 2.0 1.5 1.0 0.5 0.0
f1 (ppm)

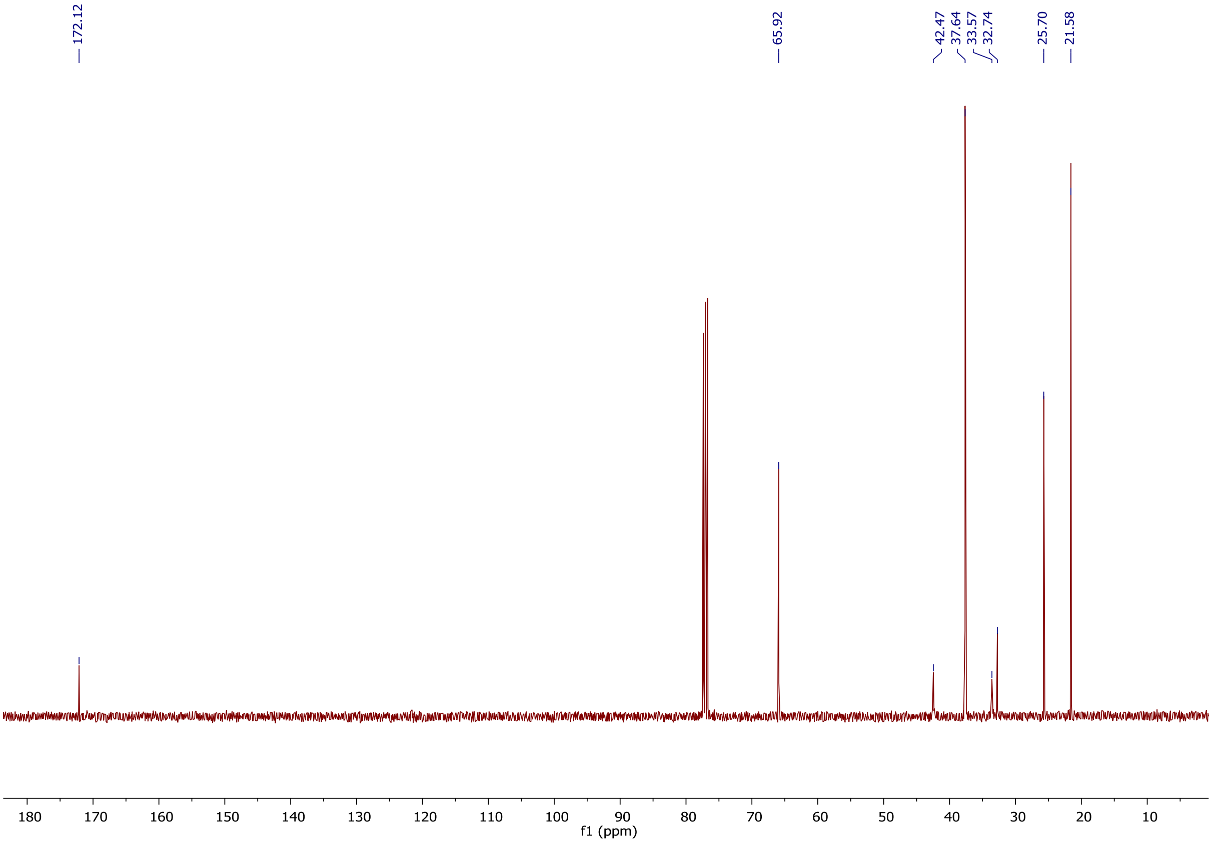

172.12
65.92
42.47
37.64
33.57
32.74
25.70
21.58
180 170 160 150 140 130 120 110 100 90 80 70 60 50 40 30 20 10
f1 (ppm)

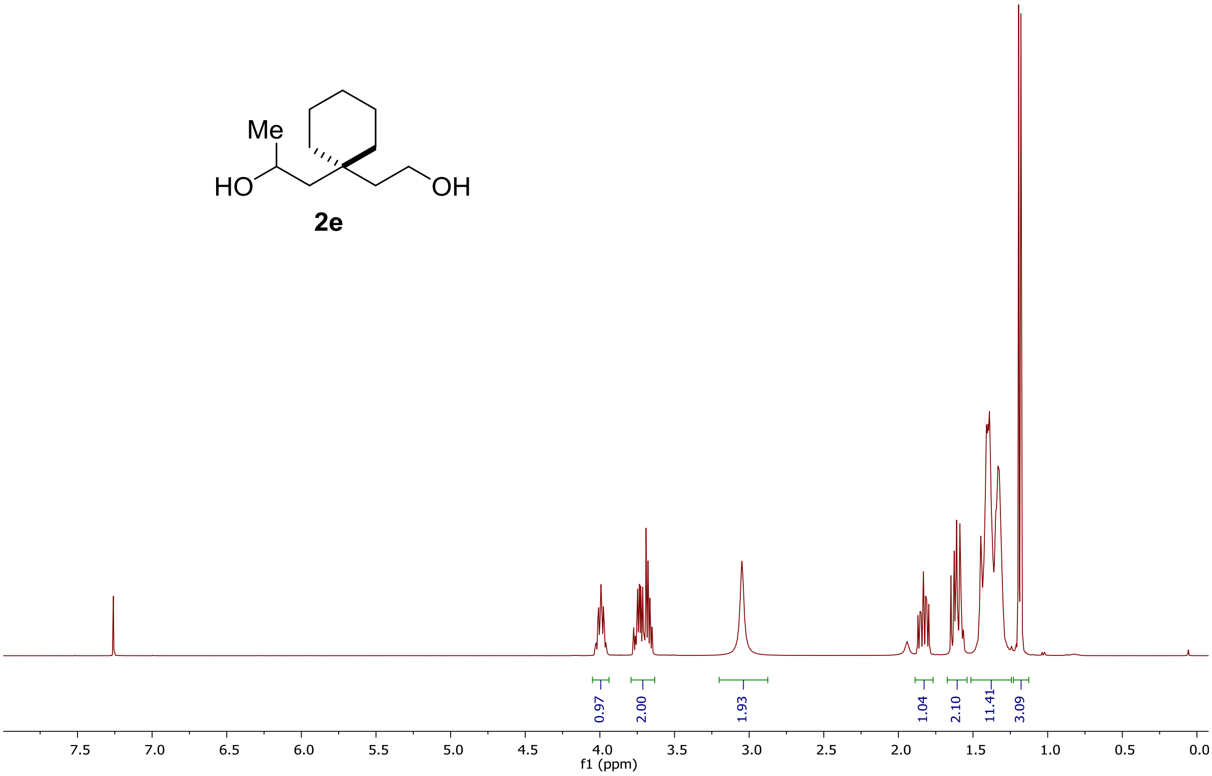

Me
HO
OH
2e
0.97
2.00
1.93
1.04
2.10
11.41
3.09
7.5
7.0
6.5
6.0
5.5
5.0
4.5
4.0
3.5
3.0
2.5
2.0
1.5
1.0
0.5
0.0
f1 (ppm)

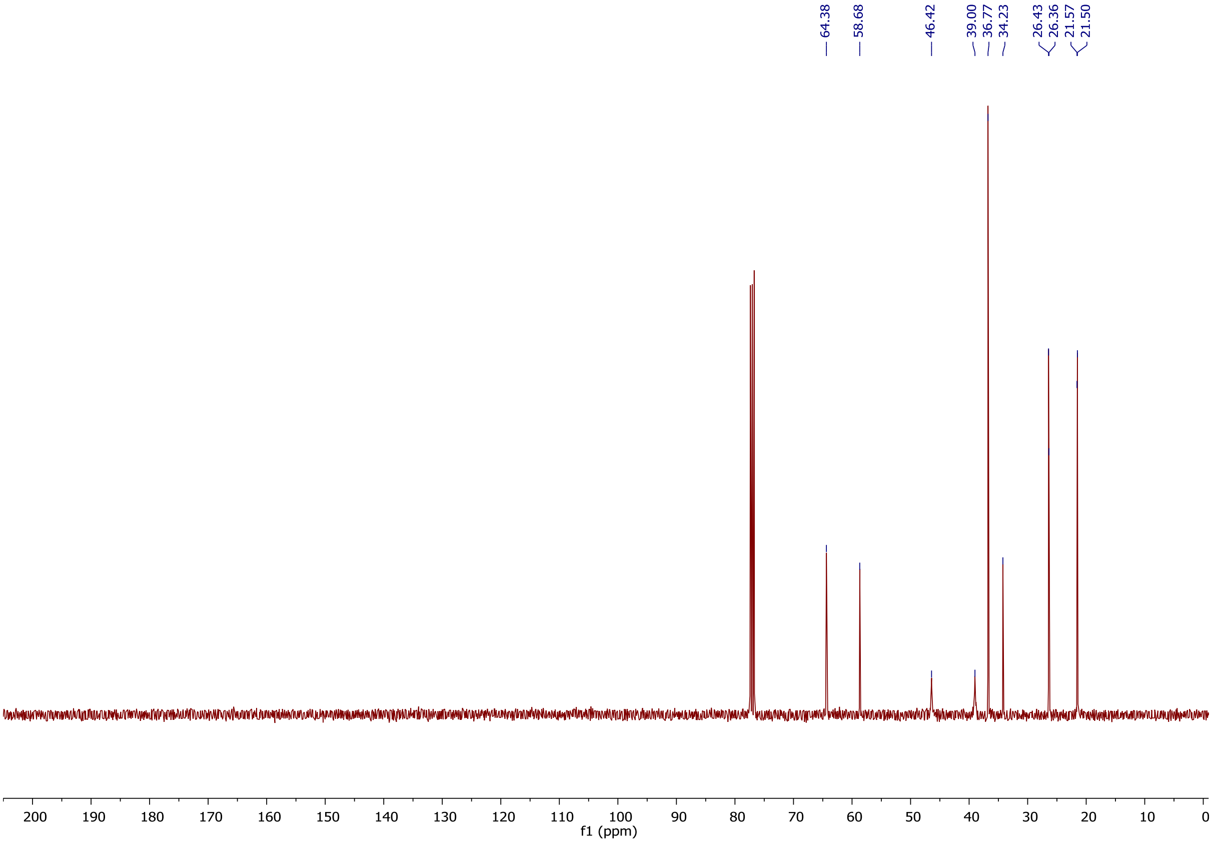

64.38
58.68
46.42
39.00
36.77
34.23
26.43
26.36
21.57
21.50
200
190
180
170
160
150
140
130
120
110
100
90
80
70
60
50
40
30
20
10
0
f1 (ppm)

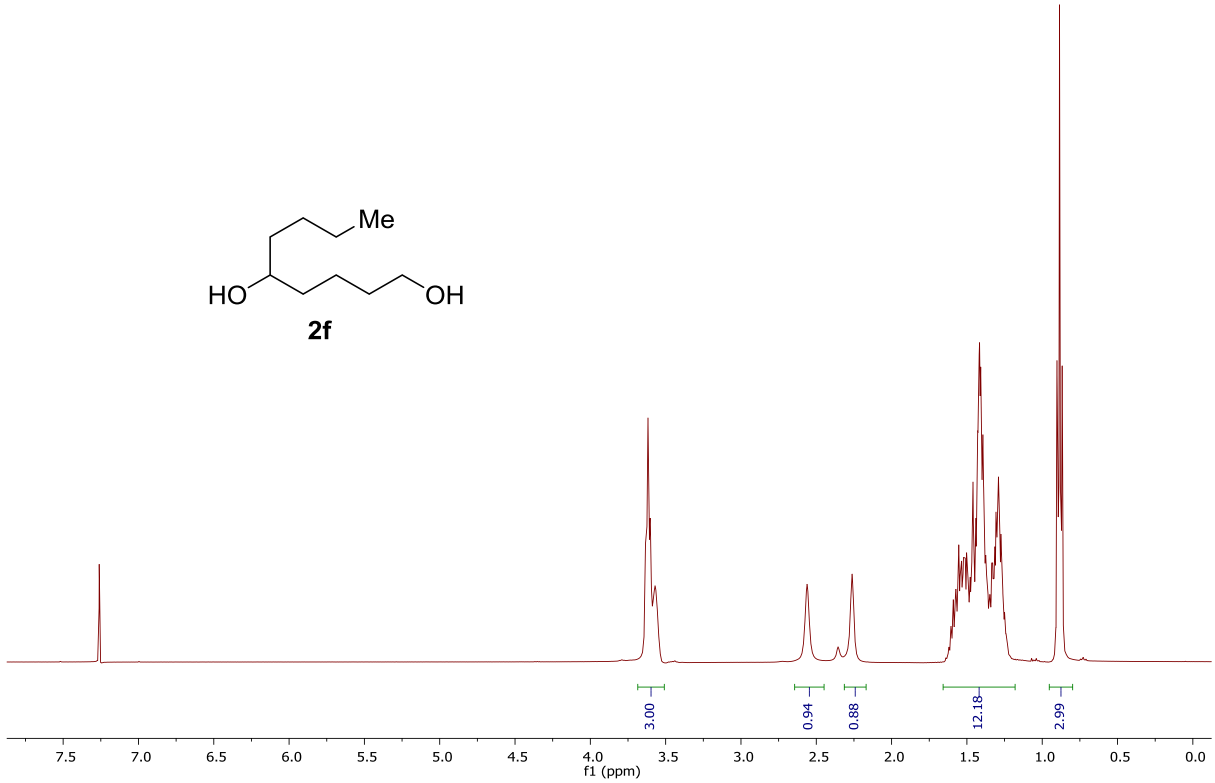

Me
HO
OH
2f
3.00
0.94
0.88
12.18
2.99
f1 (ppm)

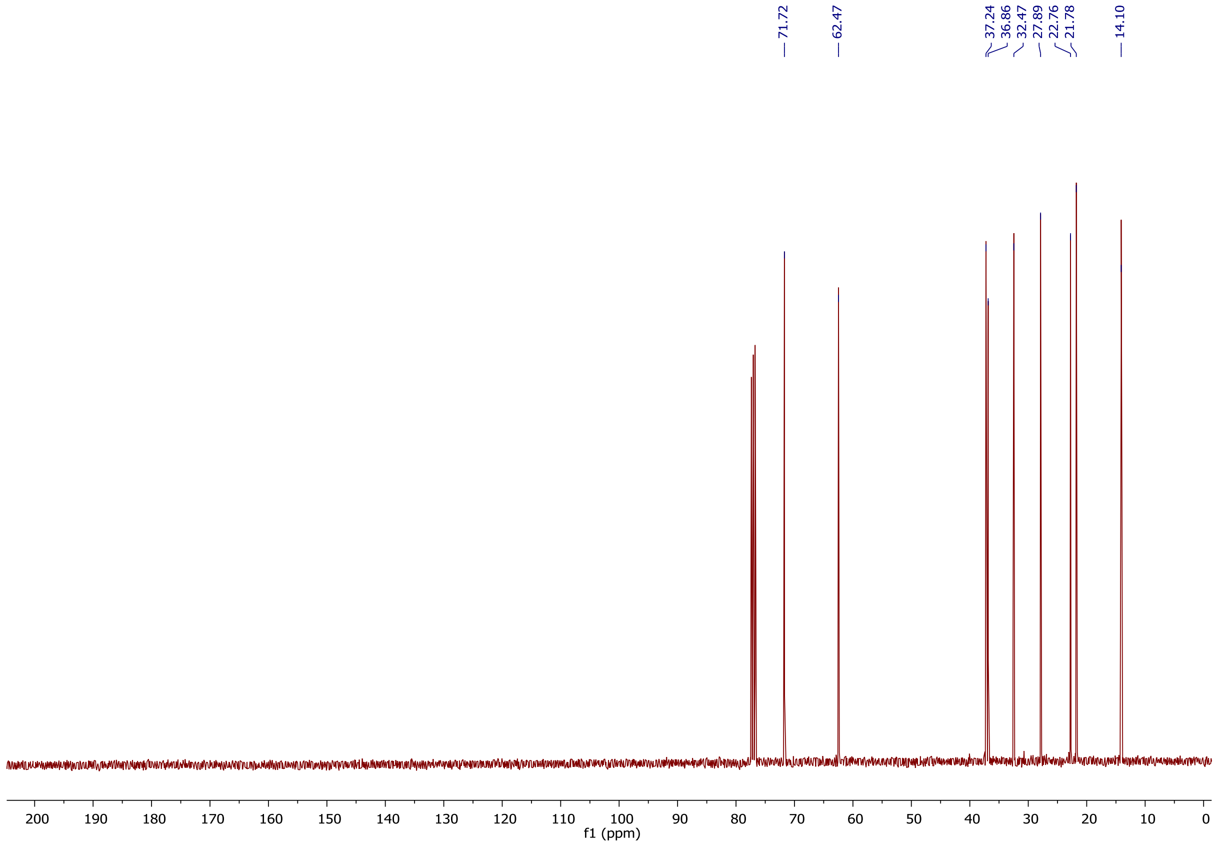

71.72
62.47
37.24
36.86
32.47
27.89
22.76
21.78
14.10
f1 (ppm)

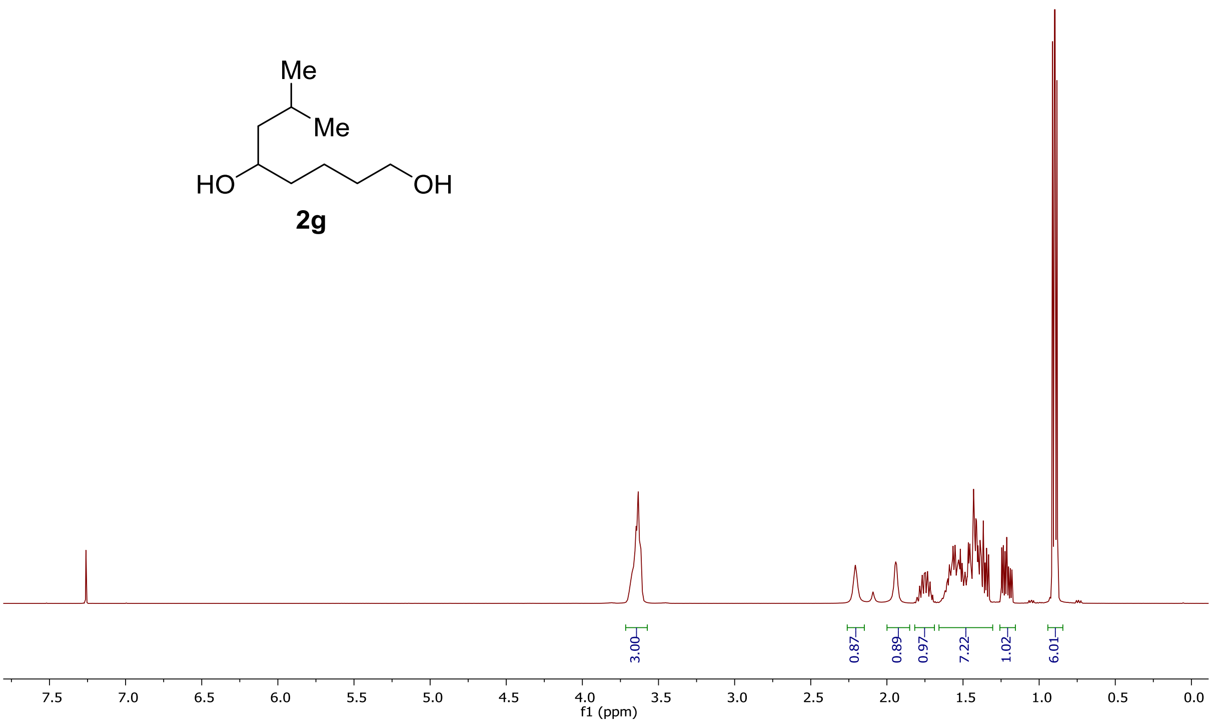
Me
Me
HO
OH
2g
3.00
0.87
0.89
0.97
7.22
1.02
6.01
7.5 7.0 6.5 6.0 5.5 5.0 4.5 4.0 3.5 3.0 2.5 2.0 1.5 1.0 0.5 0.0
f1 (ppm)

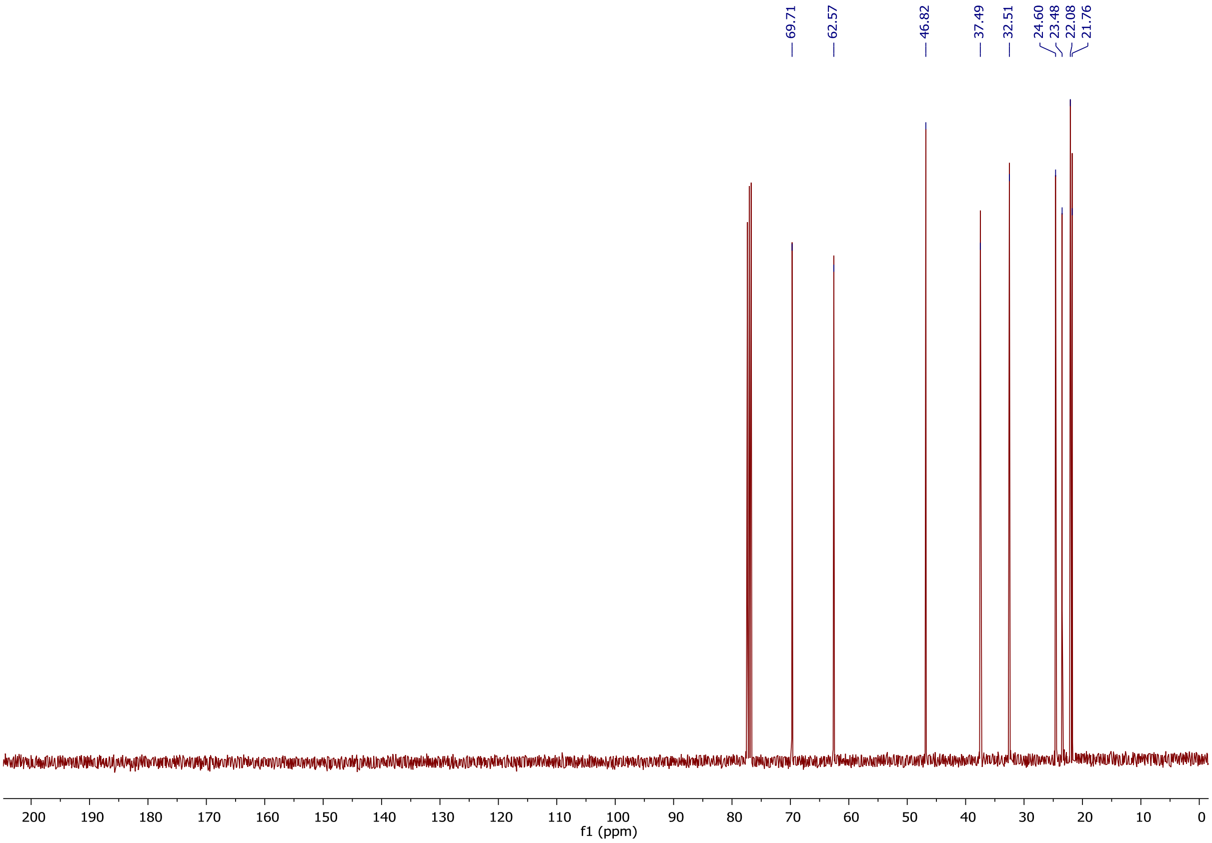
69.71
62.57
46.82
37.49
32.51
24.60
23.48
22.08
21.76
200 190 180 170 160 150 140 130 120 110 100 90 80 70 60 50 40 30 20 10 0
f1 (ppm)

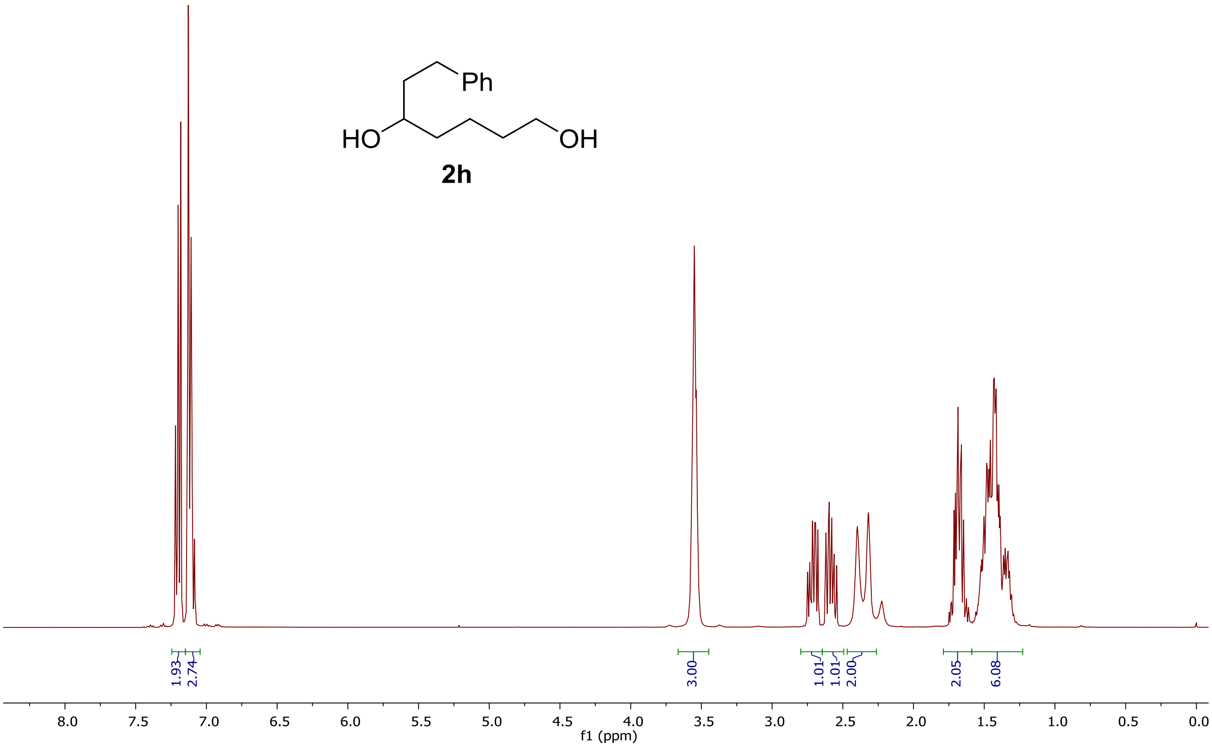
Ph
HO
OH
2h
1.93
2.74
3.00
1.01
1.01
2.00
2.05
6.08
8.0 7.5 7.0 6.5 6.0 5.5 5.0 4.5 4.0 3.5 3.0 2.5 2.0 1.5 1.0 0.5 0.0
f1 (ppm)

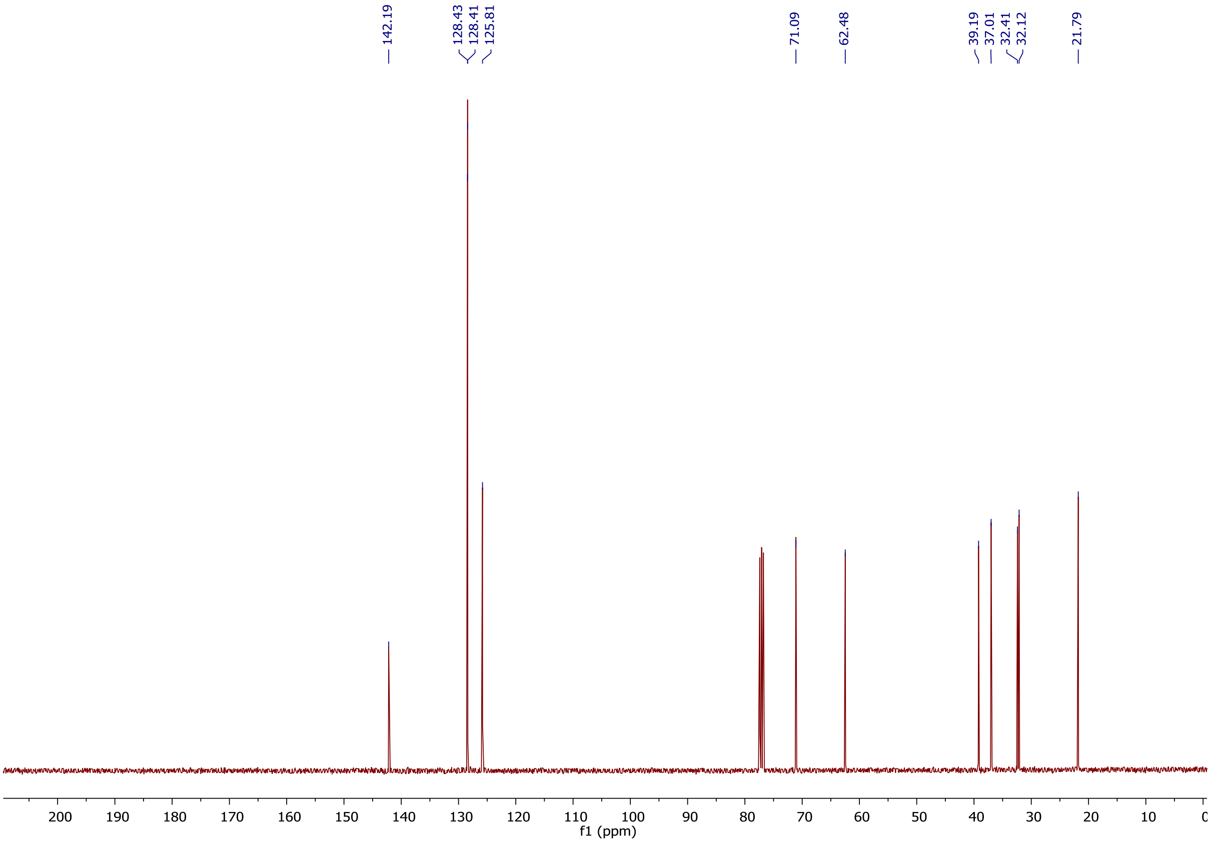
142.19
128.43
128.41
125.81
71.09
62.48
39.19
37.01
32.41
32.12
21.79
200 190 180 170 160 150 140 130 120 110 100 90 80 70 60 50 40 30 20 10 0
f1 (ppm)

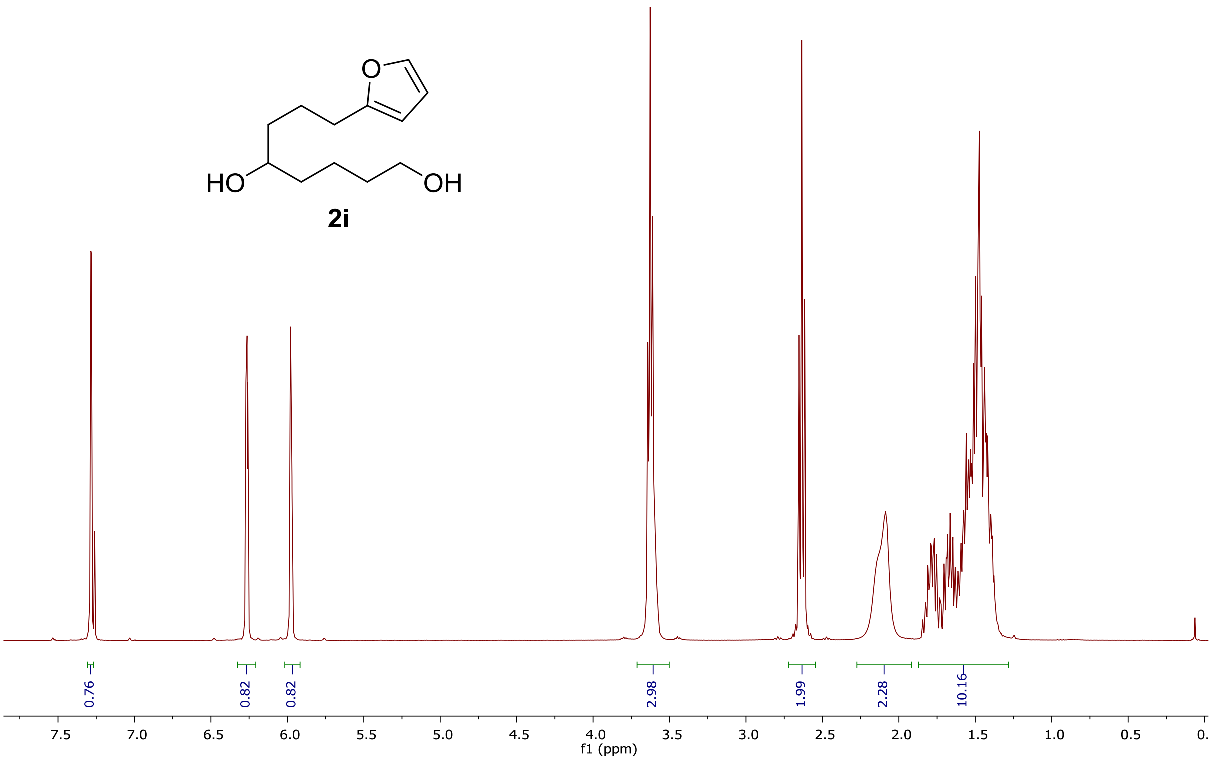
HO
OH
O
2i
0.76
0.82
0.82
2.98
1.99
2.28
10.16
7.5 7.0 6.5 6.0 5.5 5.0 4.5 4.0 3.5 3.0 2.5 2.0 1.5 1.0 0.5 0.
f1 (ppm)

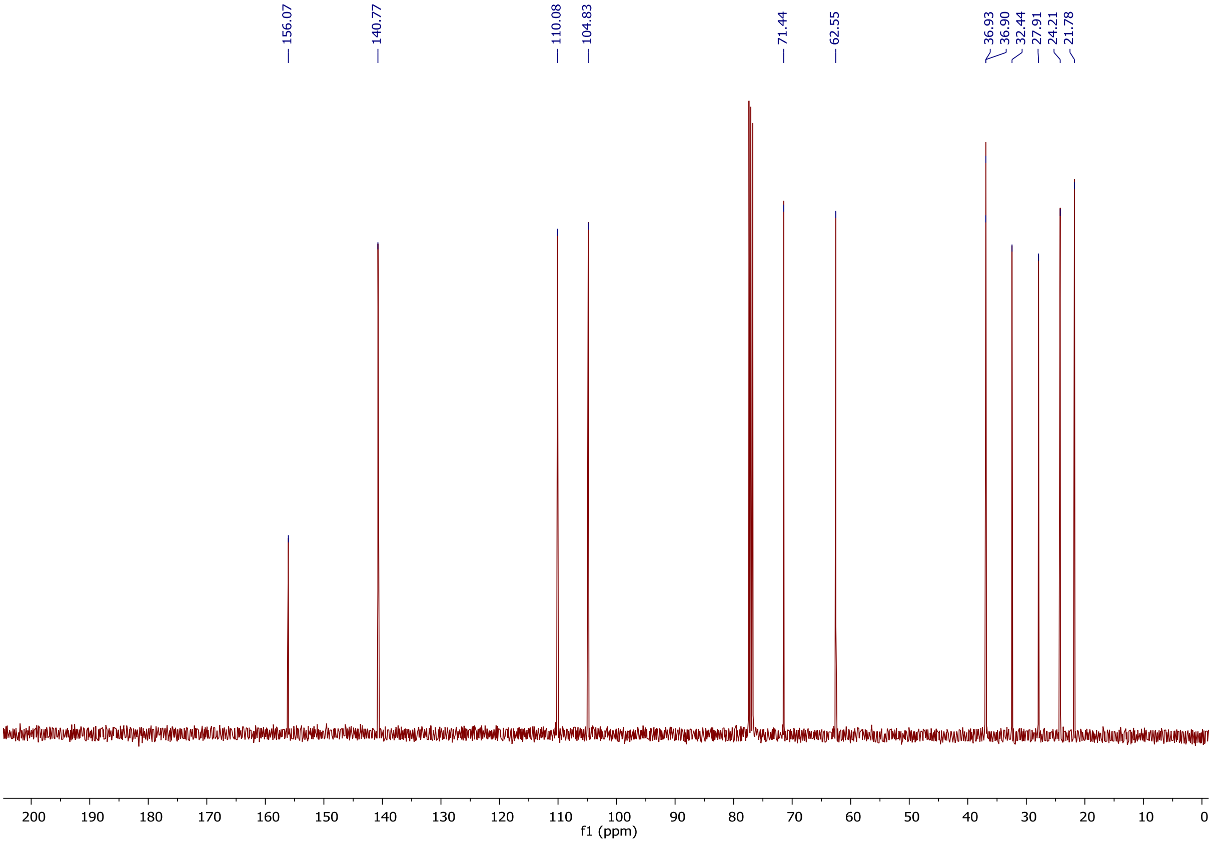
156.07
140.77
110.08
104.83
71.44
62.55
36.93
36.90
32.44
27.91
24.21
21.78
200 190 180 170 160 150 140 130 120 110 100 90 80 70 60 50 40 30 20 10 0
f1 (ppm)

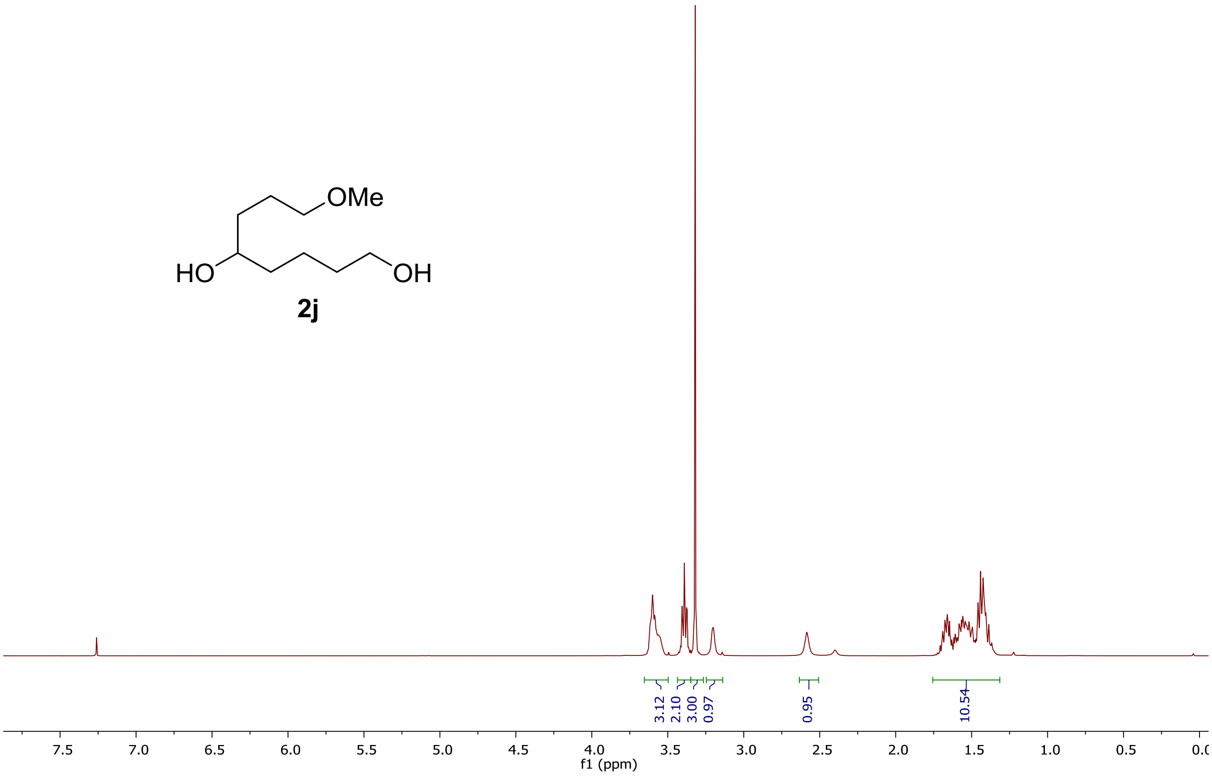
OMe
HO
OH
2j
3.12
2.10
3.00
0.97
0.95
10.54
7.5 7.0 6.5 6.0 5.5 5.0 4.5 4.0 3.5 3.0 2.5 2.0 1.5 1.0 0.5 0.0
f1 (ppm)

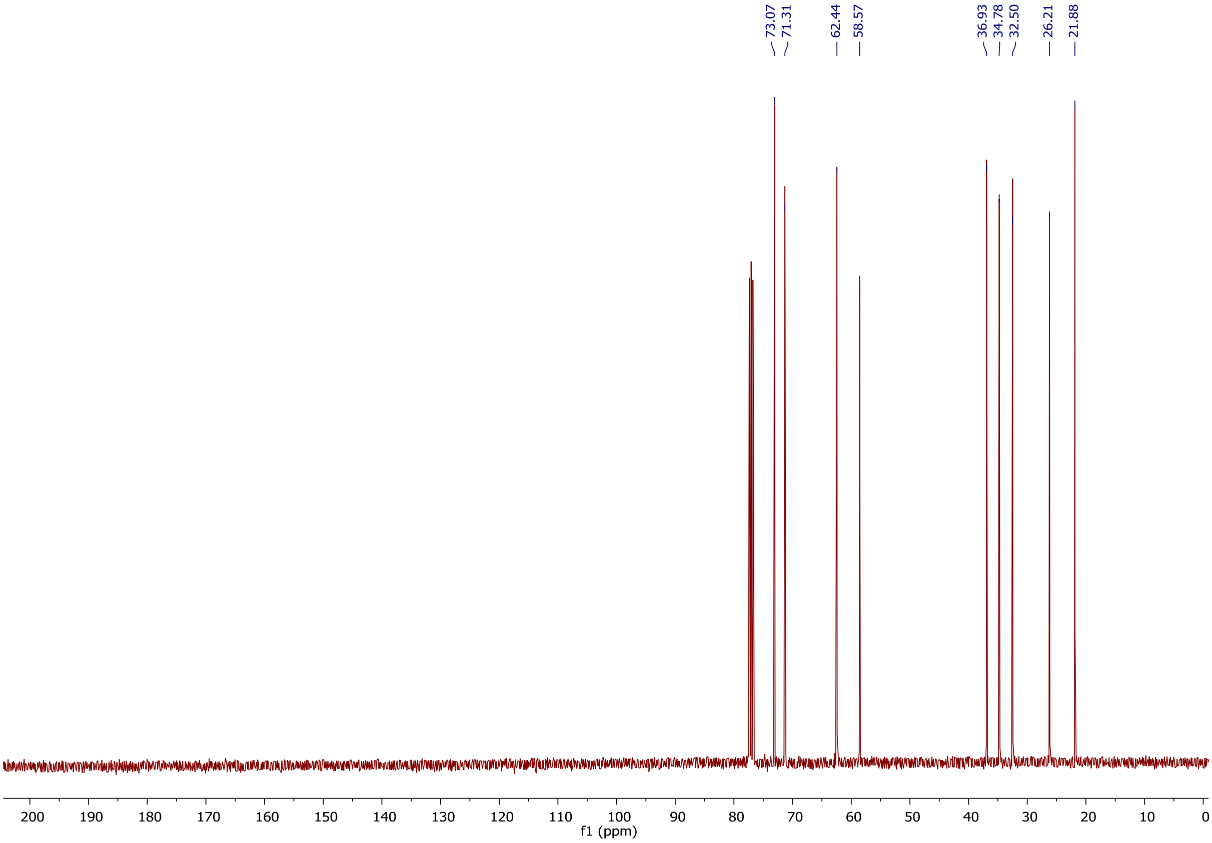
73.07
71.31
62.44
58.57
36.93
34.78
32.50
26.21
21.88
200 190 180 170 160 150 140 130 120 110 100 90 80 70 60 50 40 30 20 10 0
f1 (ppm)

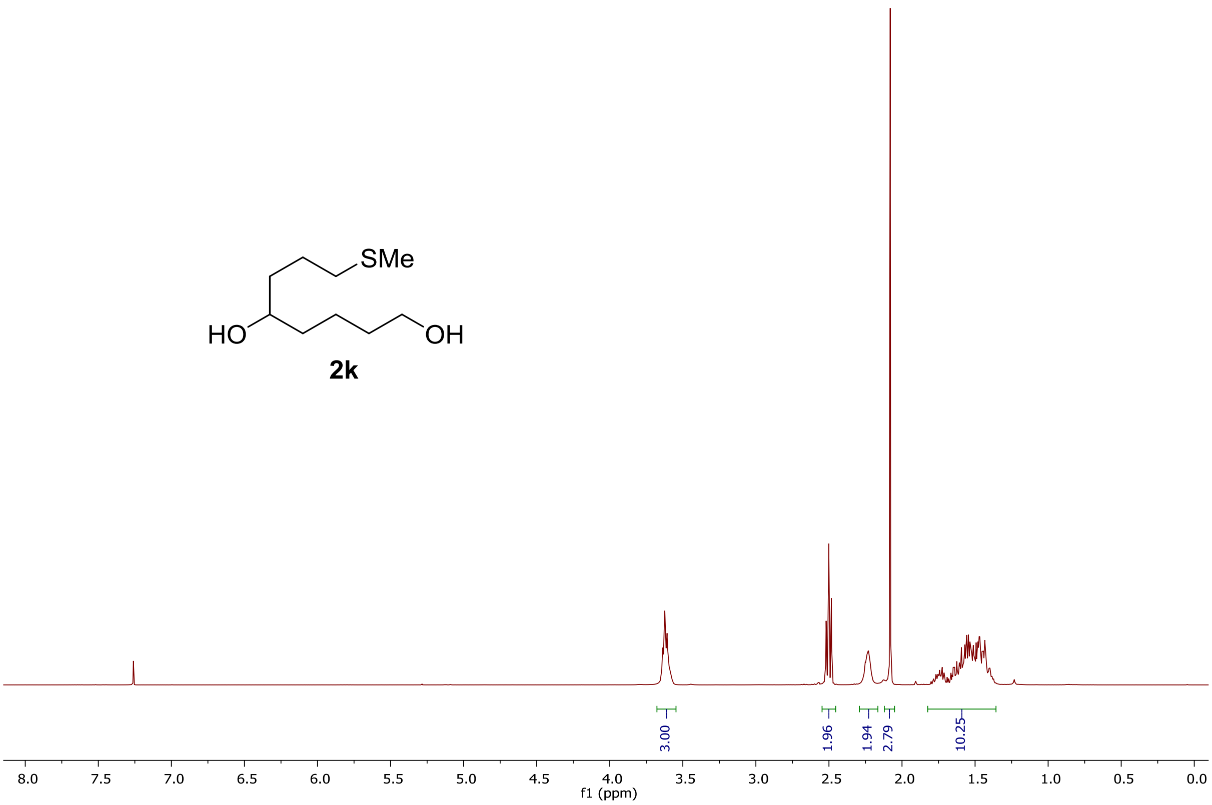
SMe
HO
OH
3.00
1.96
1.94
2.79
10.25
8.0
7.5
7.0
6.5
6.0
5.5
5.0
4.5
4.0
3.5
3.0
2.5
2.0
1.5
1.0
0.5
0.0
f1 (ppm)

2k

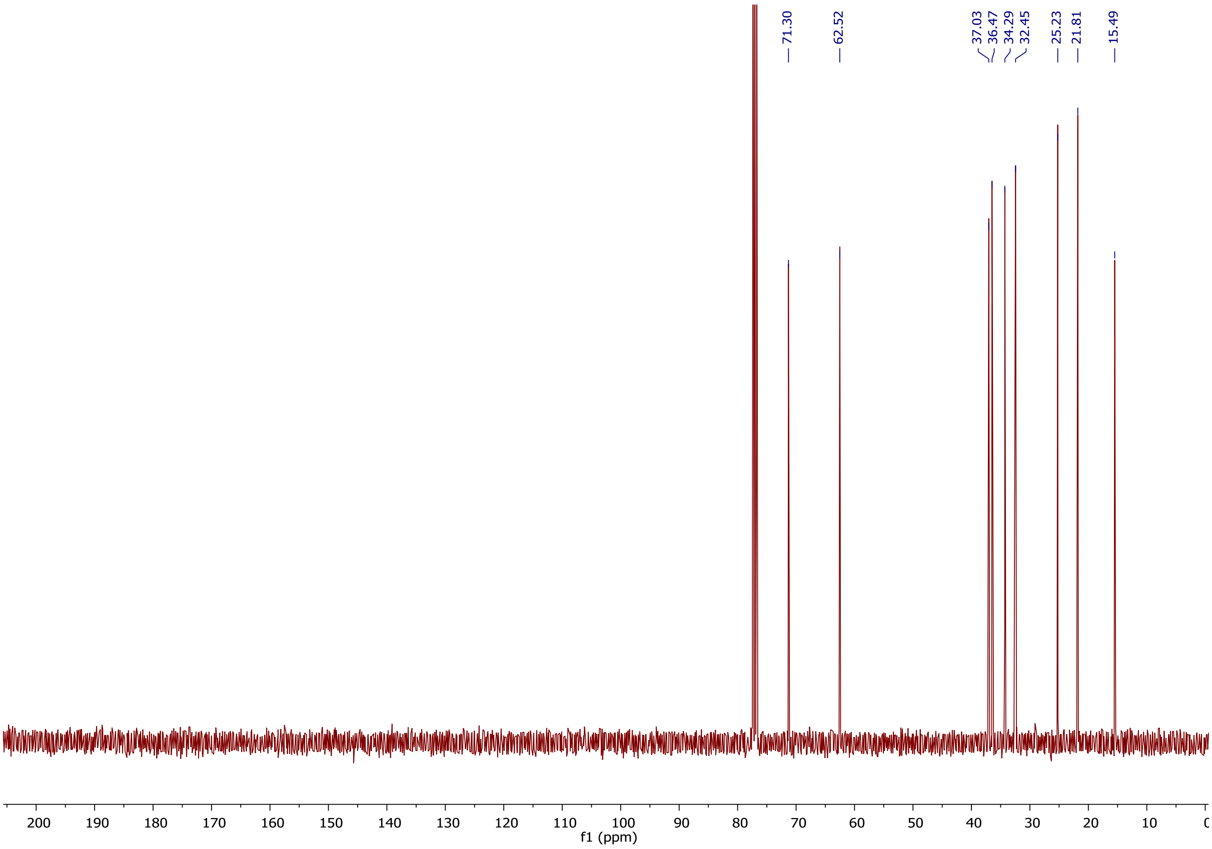
71.30
62.52
37.03
36.47
34.29
32.45
25.23
21.81
15.49
200
190
180
170
160
150
140
130
120
110
100
90
80
70
60
50
40
30
20
10
f1 (ppm)

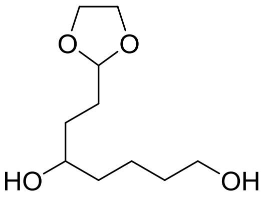
O
O
HO
OH

2l

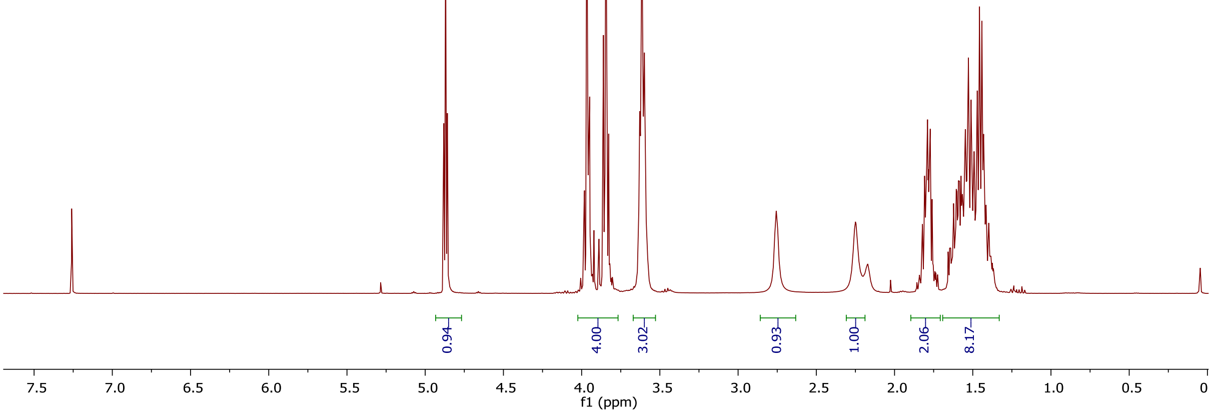
0.94
4.00
3.02
0.93
1.00
2.06
8.17
7.5
7.0
6.5
6.0
5.5
5.0
4.5
4.0
3.5
3.0
2.5
2.0
1.5
1.0
0.5
0
f1 (ppm)

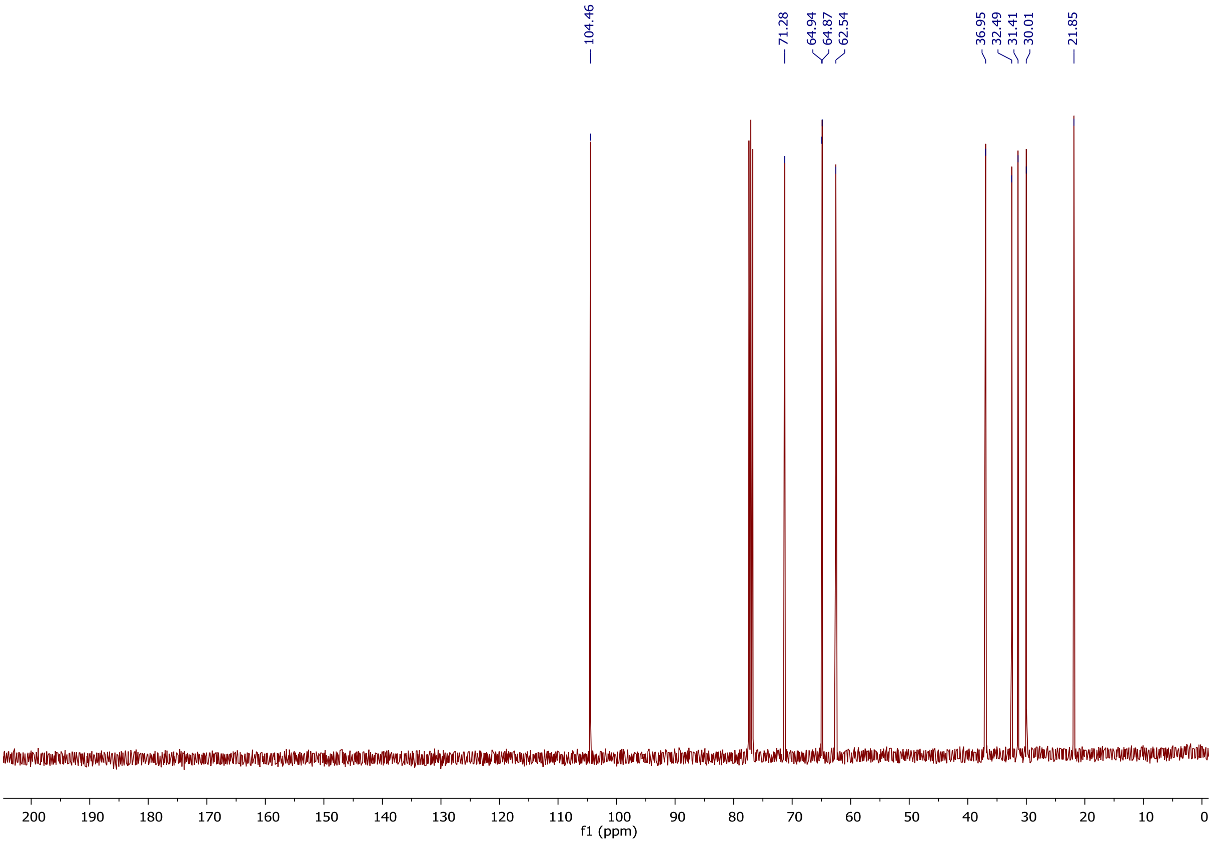
104.46
71.28
64.94
64.87
62.54
36.95
32.49
31.41
30.01
21.85
200
190
180
170
160
150
140
130
120
110
100
90
80
70
60
50
40
30
20
10
0
f1 (ppm)

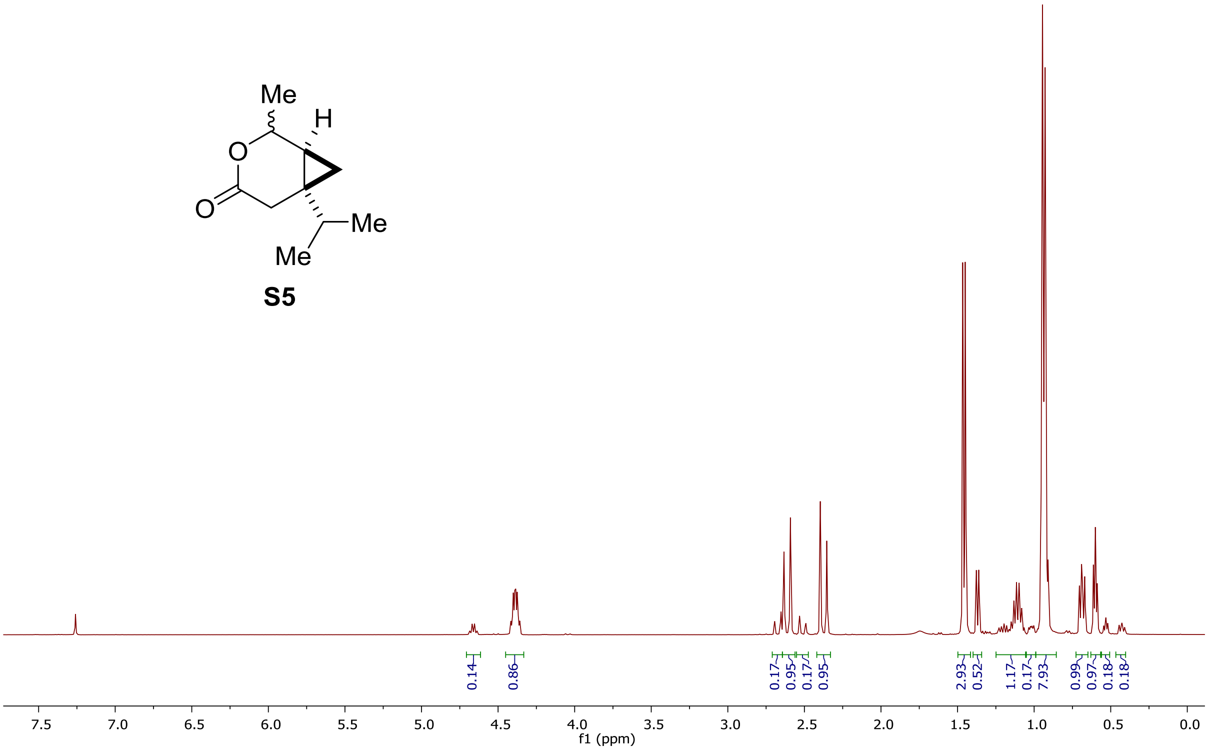
Me
H
O
O
Me
Me
0.14
0.86
0.17
0.95
0.17
0.95
2.93
0.52
1.17
0.17
7.93
0.99
0.97
0.18
0.18
7.5 7.0 6.5 6.0 5.5 5.0 4.5 4.0 3.5 3.0 2.5 2.0 1.5 1.0 0.5 0.0
f1 (ppm)

S5

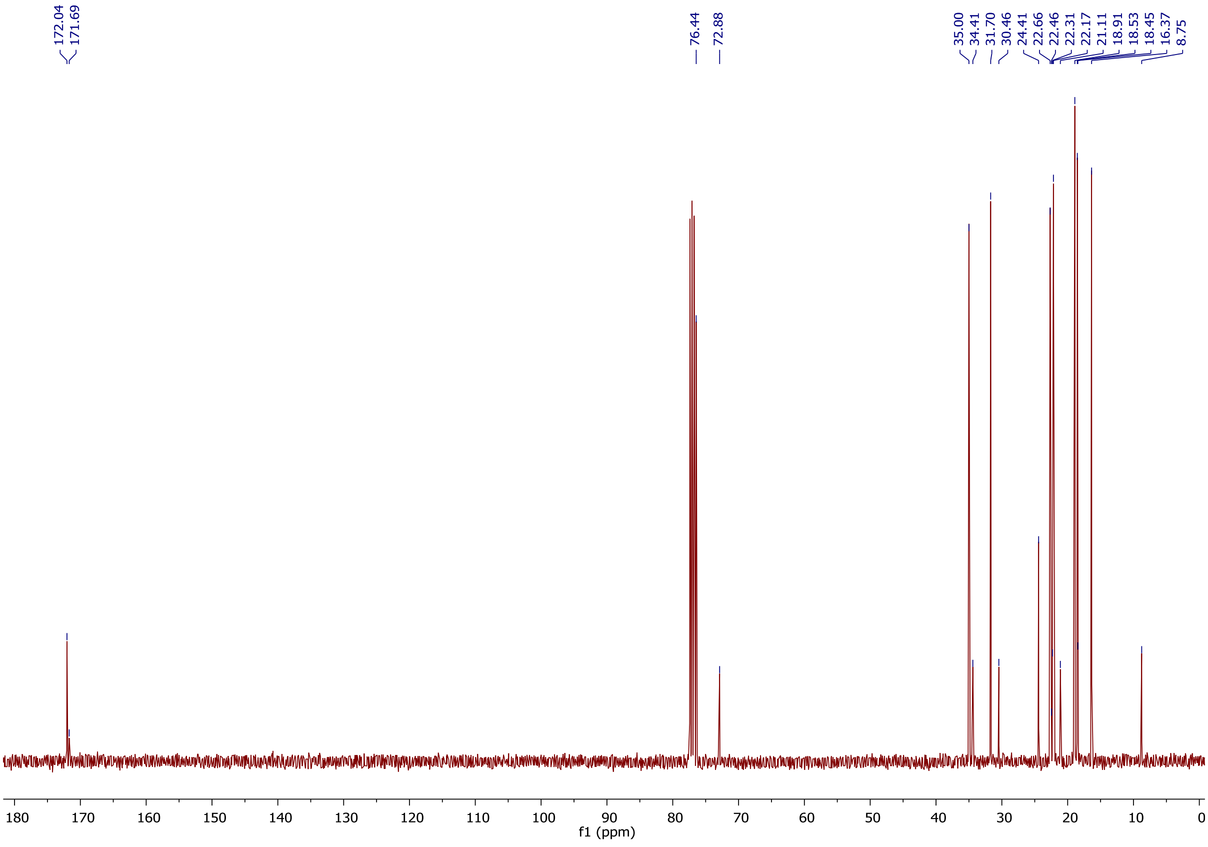
172.04
171.69
76.44
72.88
35.00
34.41
31.70
30.46
24.41
22.66
22.46
22.31
22.17
21.11
18.91
18.53
18.45
16.37
8.75
180 170 160 150 140 130 120 110 100 90 80 70 60 50 40 30 20 10 0
f1 (ppm)

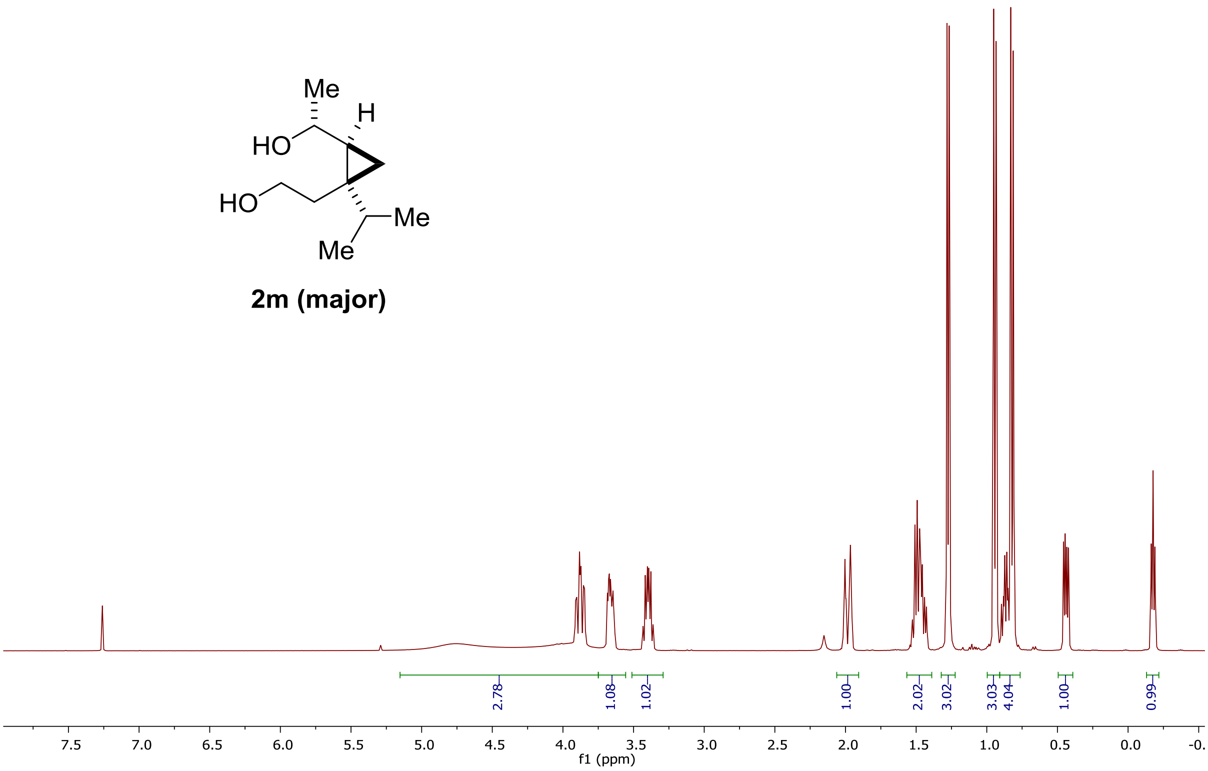
Me
H
HO
HO
Me
Me
2m (major)
2.78
1.08
1.02
1.00
2.02
3.02
3.03
4.04
1.00
0.99
7.5 7.0 6.5 6.0 5.5 5.0 4.5 4.0 3.5 3.0 2.5 2.0 1.5 1.0 0.5 0.0 -0.
f1 (ppm)

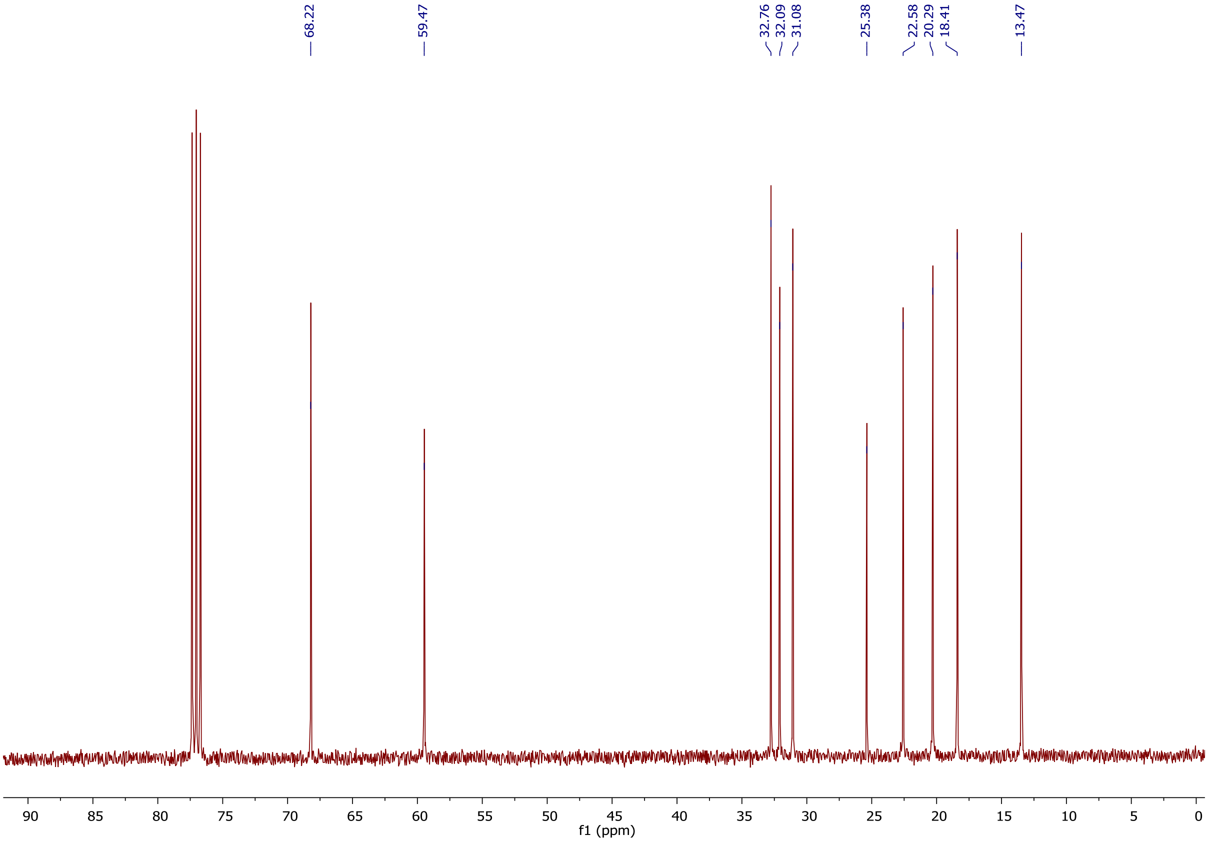
68.22
59.47
32.76
32.09
31.08
25.38
22.58
20.29
18.41
13.47
90 85 80 75 70 65 60 55 50 45 40 35 30 25 20 15 10 5 0
f1 (ppm)

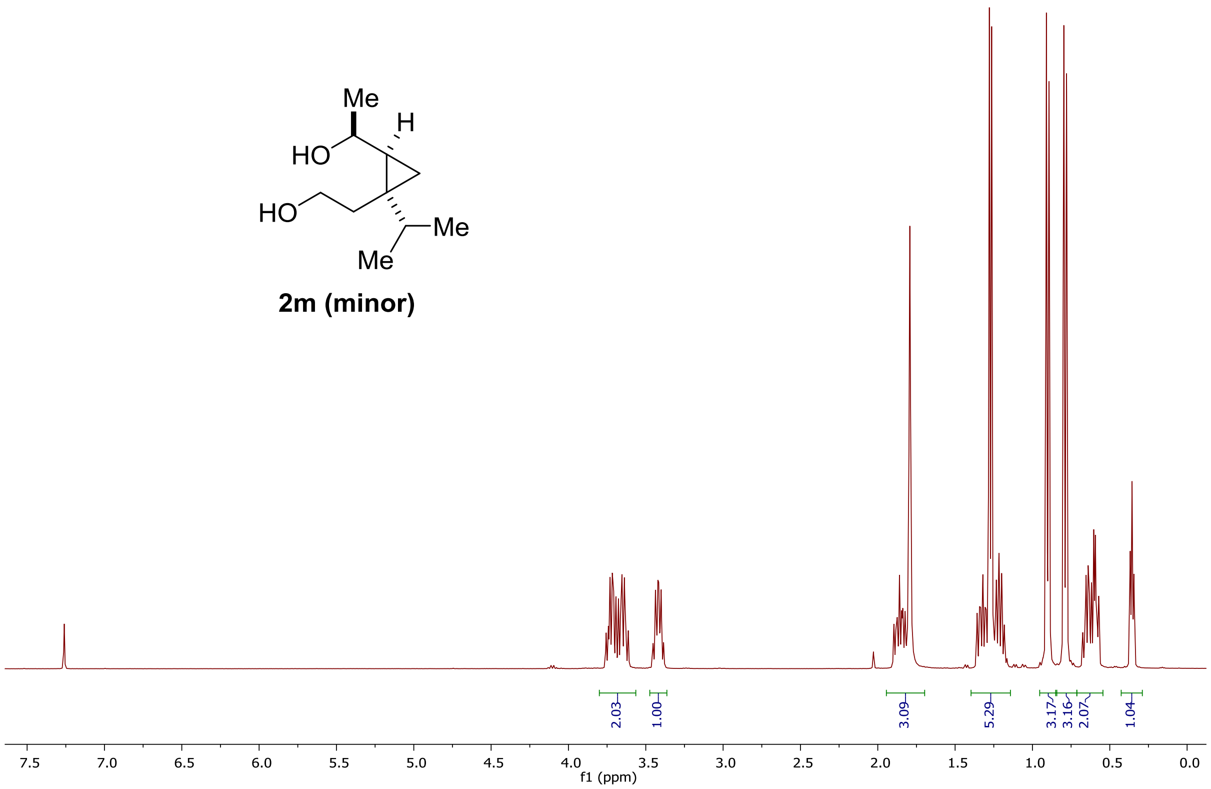
Me
H
HO
HO
Me
Me
2m (minor)
2.03
1.00
3.09
5.29
3.17
3.16
2.07
1.04
7.5 7.0 6.5 6.0 5.5 5.0 4.5 4.0 3.5 3.0 2.5 2.0 1.5 1.0 0.5 0.0
f1 (ppm)

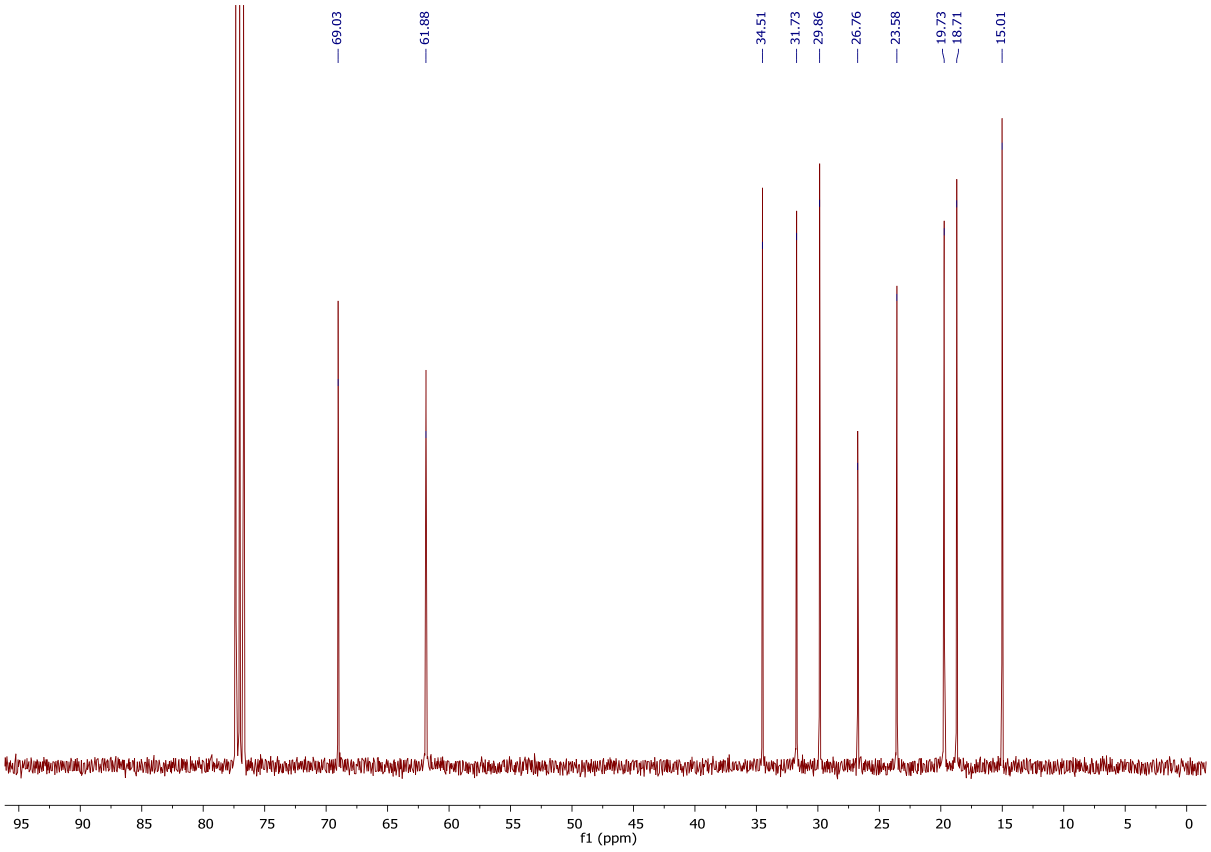
69.03
61.88
34.51
31.73
29.86
26.76
23.58
19.73
18.71
15.01
95 90 85 80 75 70 65 60 55 50 45 40 35 30 25 20 15 10 5 0
f1 (ppm)

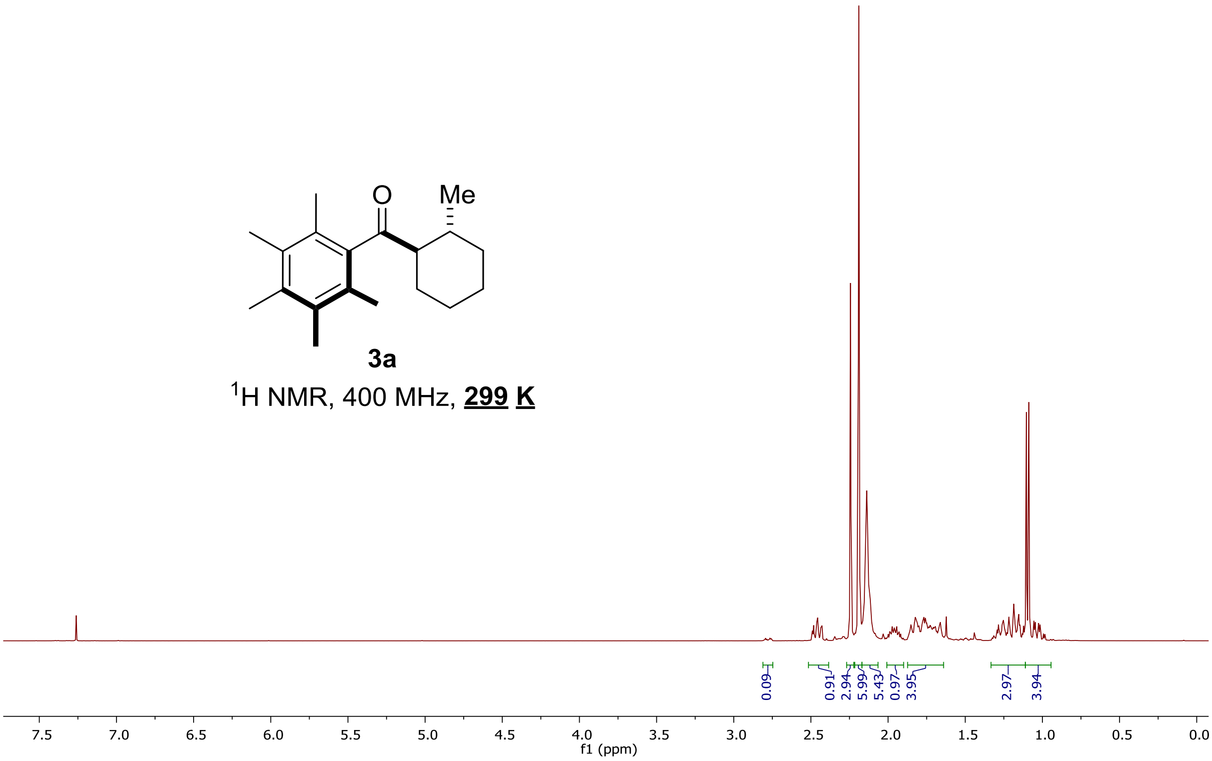
O
Me
3a
1H NMR, 400 MHz, 299 K
0.09
0.91
2.94
5.99
5.43
0.97
3.95
2.97
3.94
7.5 7.0 6.5 6.0 5.5 5.0 4.5 4.0 3.5 3.0 2.5 2.0 1.5 1.0 0.5 0.0
f1 (ppm)

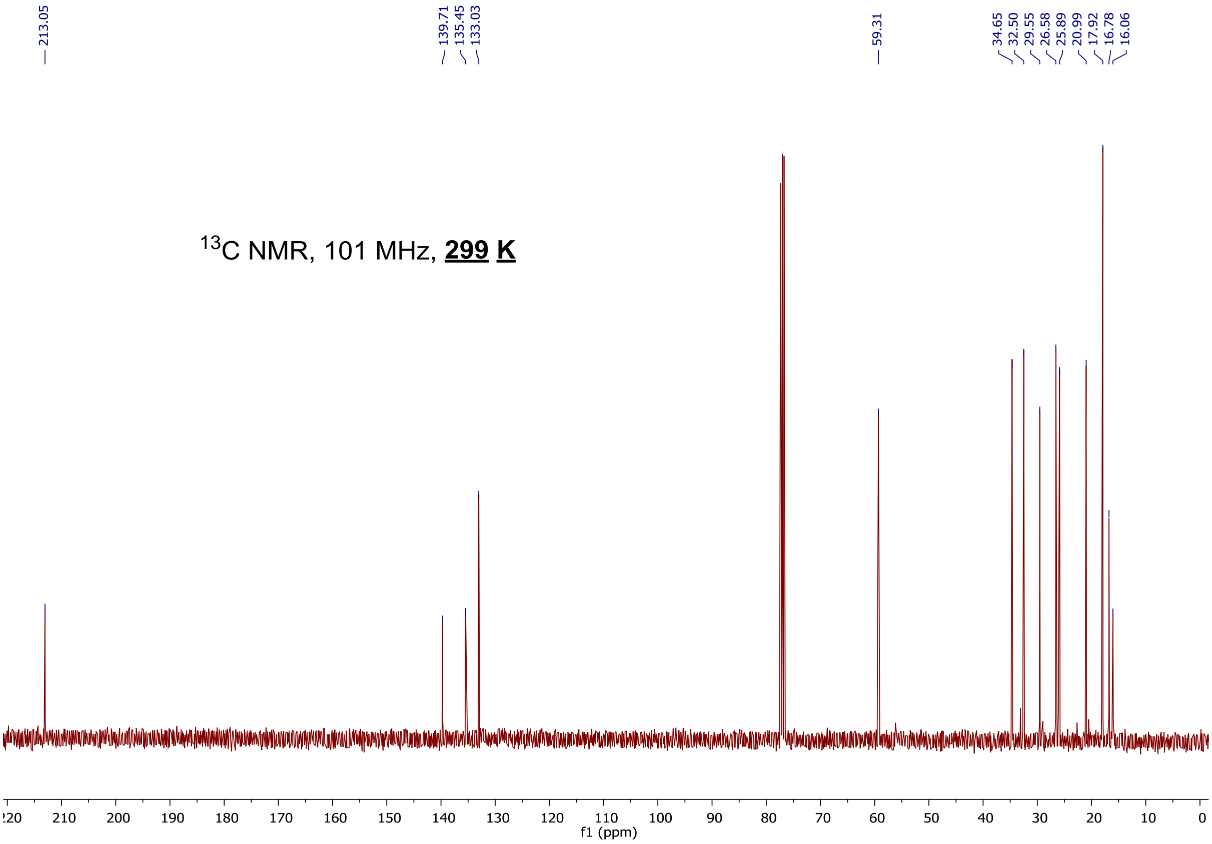
213.05
139.71
135.45
133.03
59.31
34.65
32.50
29.55
26.58
25.89
20.99
17.92
16.78
16.06
13C NMR, 101 MHz, 299 K
220 210 200 190 180 170 160 150 140 130 120 110 100 90 80 70 60 50 40 30 20 10 0
f1 (ppm)

3a

^{1}H NMR, 500 MHz, **318 K**

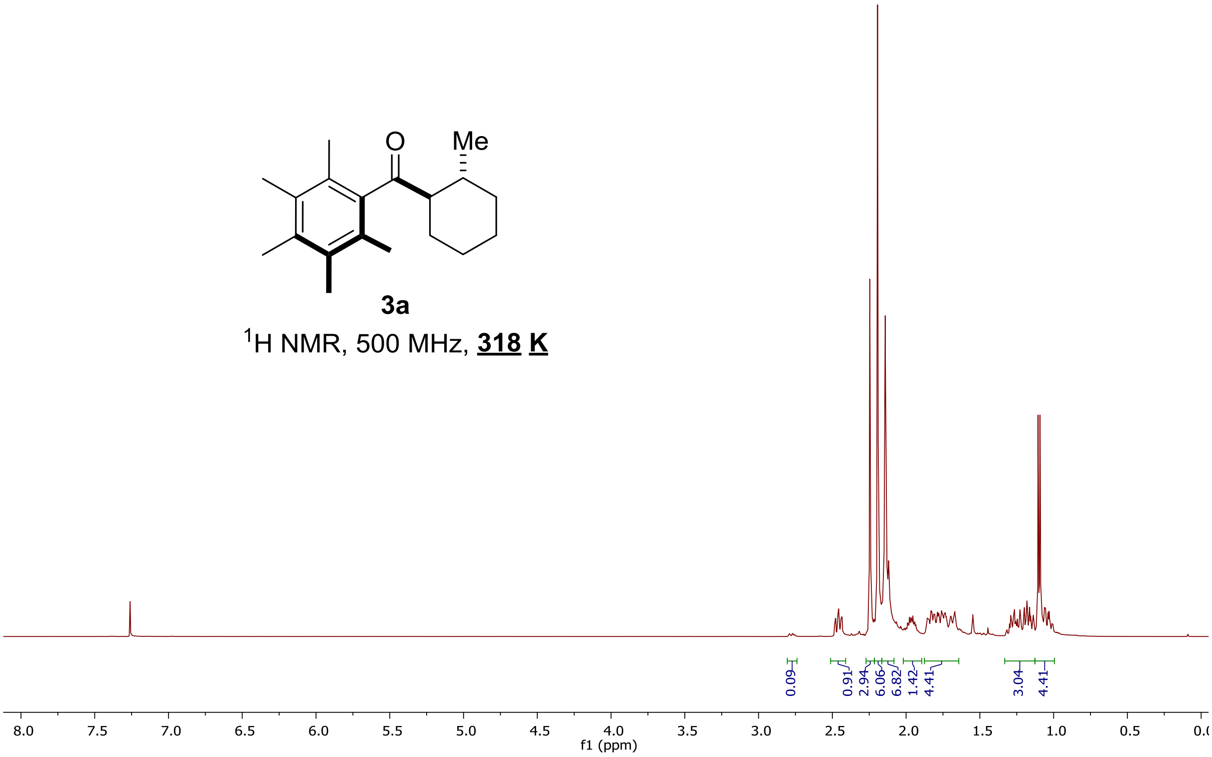

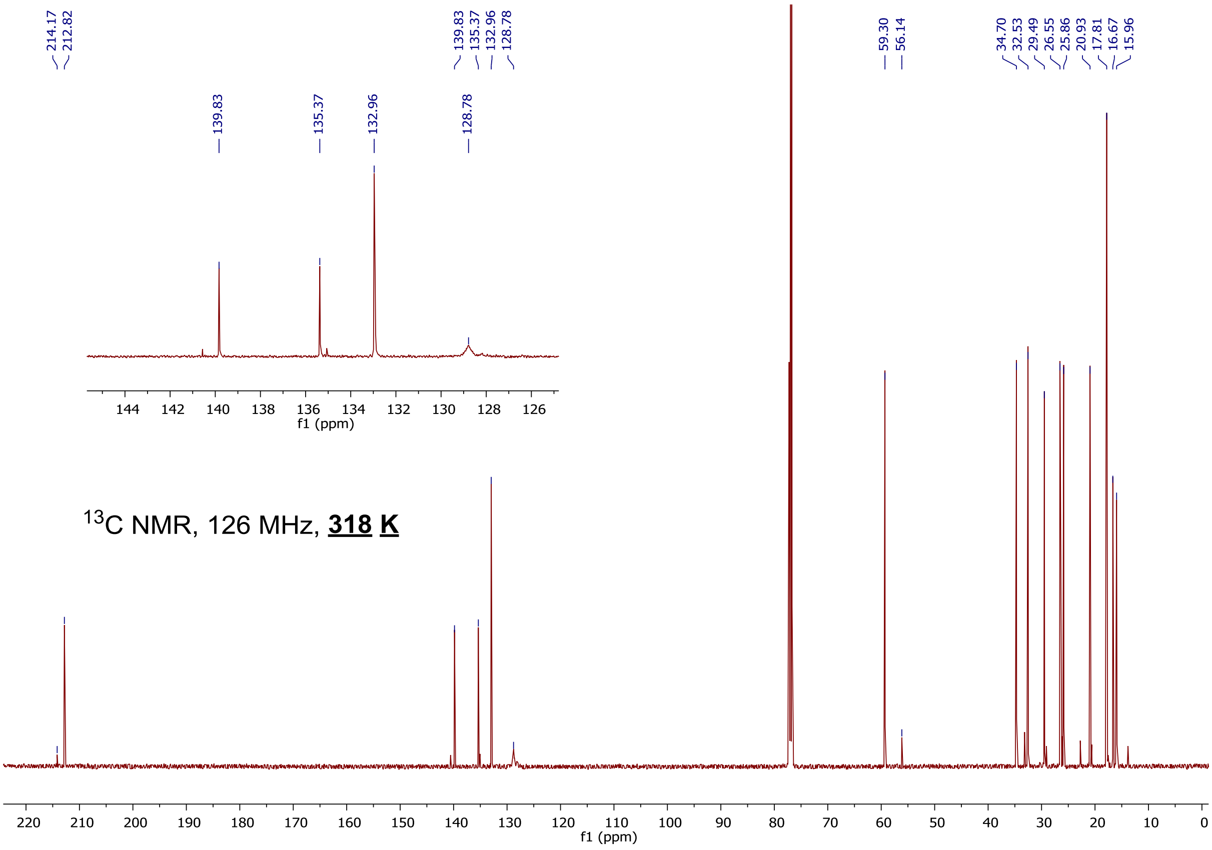

^{13}C NMR, 126 MHz, **318 K**

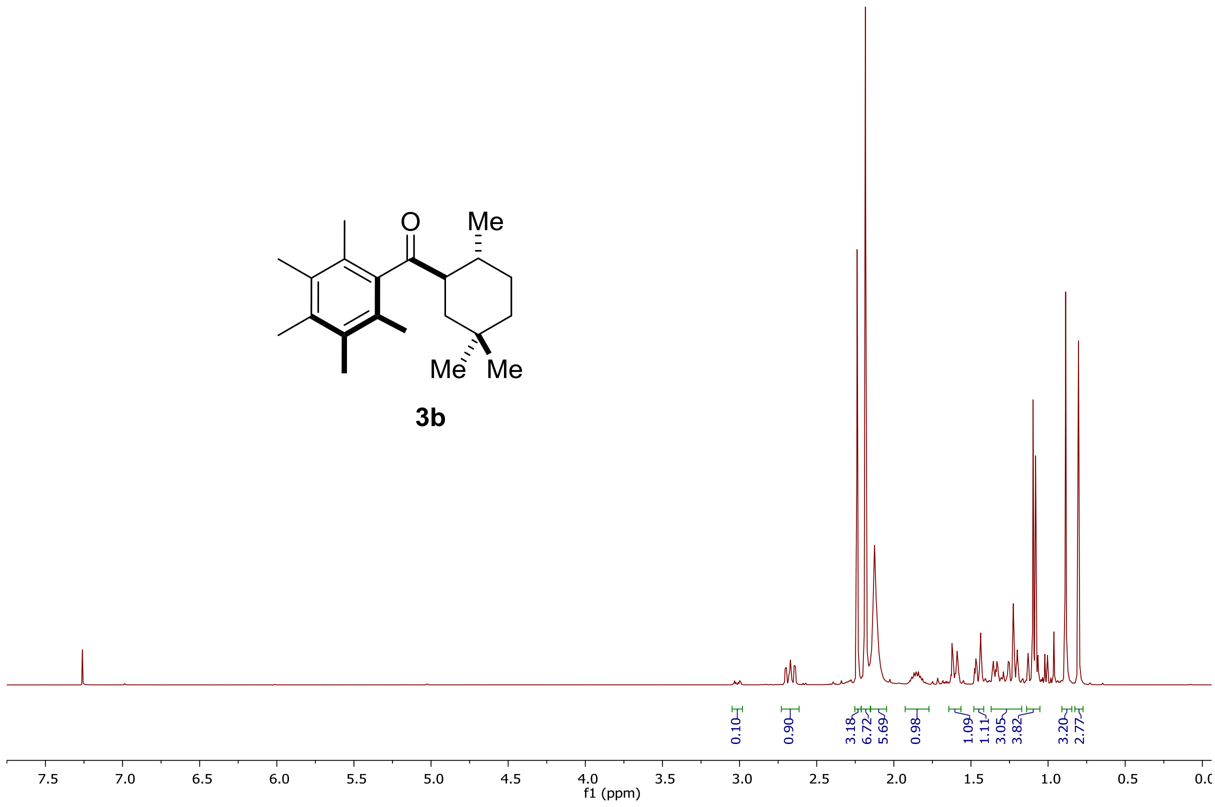
O
Me
Me
Me
3b
0.10
0.90
3.18
6.72
5.69
0.98
1.09
1.11
3.05
3.82
3.20
2.77
7.5 7.0 6.5 6.0 5.5 5.0 4.5 4.0 3.5 3.0 2.5 2.0 1.5 1.0 0.5 0.0
f1 (ppm)

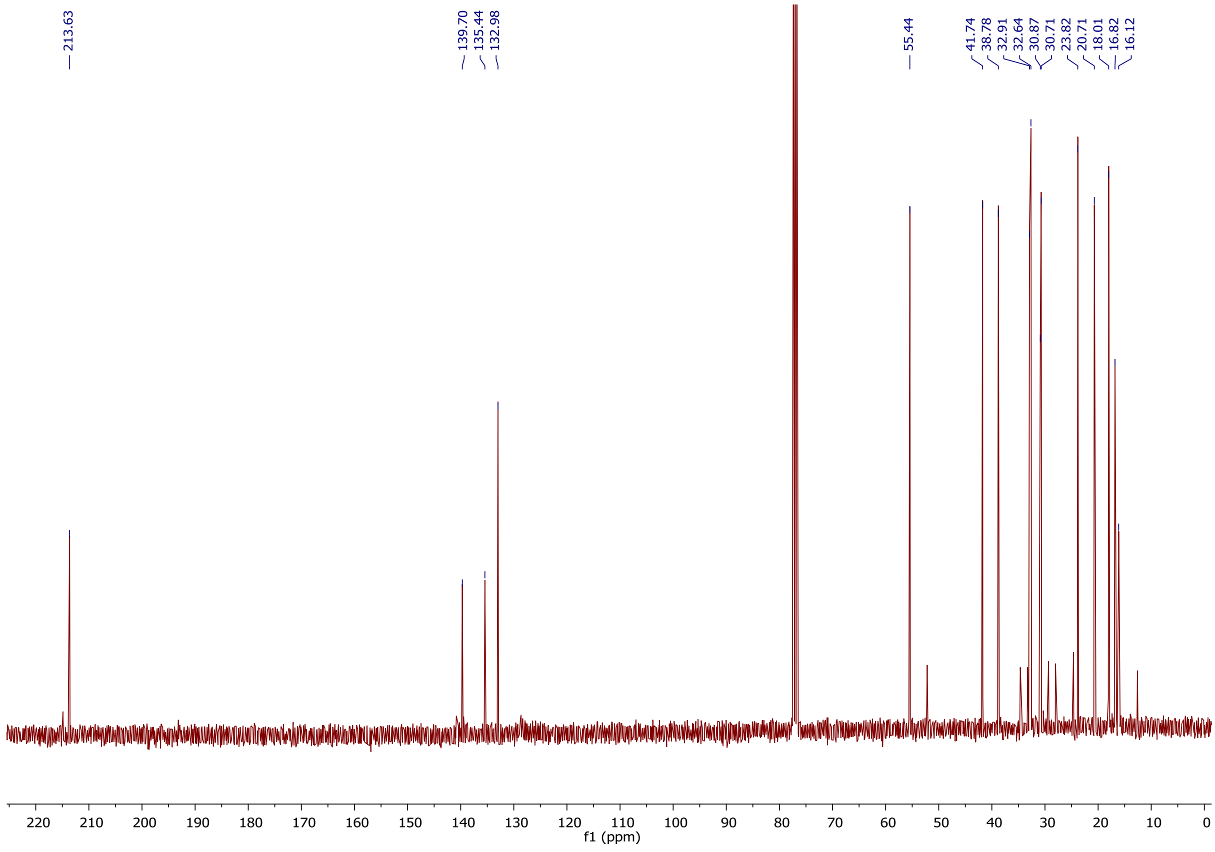
213.63
139.70
135.44
132.98
55.44
41.74
38.78
32.91
32.64
30.87
30.71
23.82
20.71
18.01
16.82
16.12
220 210 200 190 180 170 160 150 140 130 120 110 100 90 80 70 60 50 40 30 20 10 0
f1 (ppm)

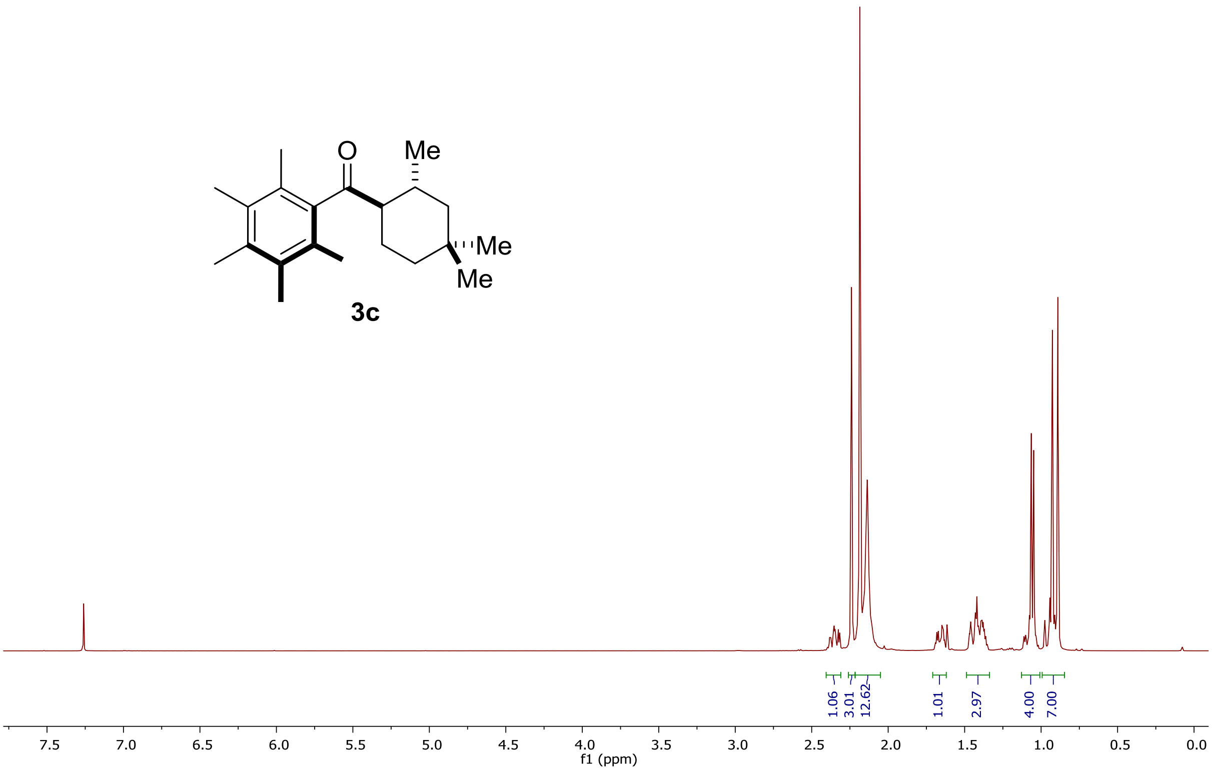
O
Me
Me
Me
1.06
3.01
12.62
1.01
2.97
4.00
7.00
7.5 7.0 6.5 6.0 5.5 5.0 4.5 4.0 3.5 3.0 2.5 2.0 1.5 1.0 0.5 0.0
f1 (ppm)

3c

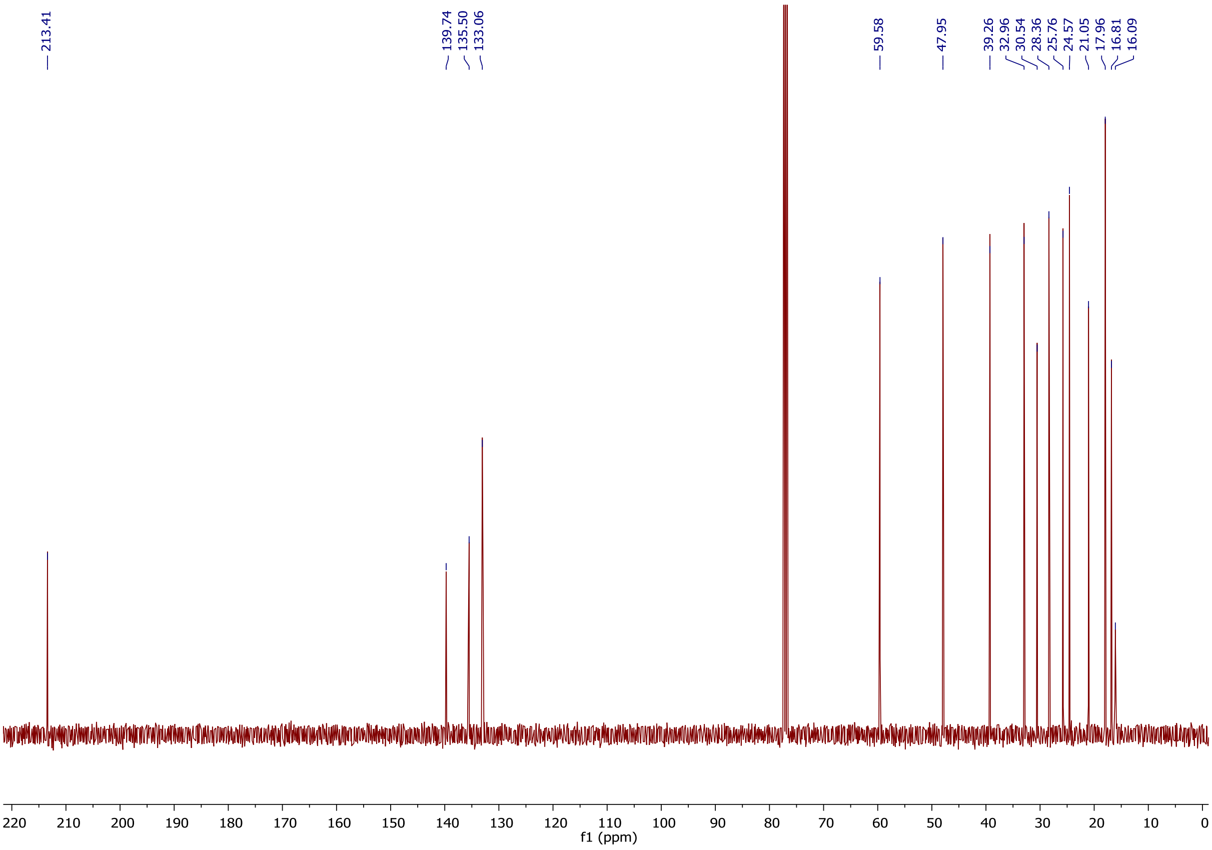
213.41
139.74
135.50
133.06
59.58
47.95
39.26
32.96
30.54
28.36
25.76
24.57
21.05
17.96
16.81
16.09
220 210 200 190 180 170 160 150 140 130 120 110 100 90 80 70 60 50 40 30 20 10 0
f1 (ppm)

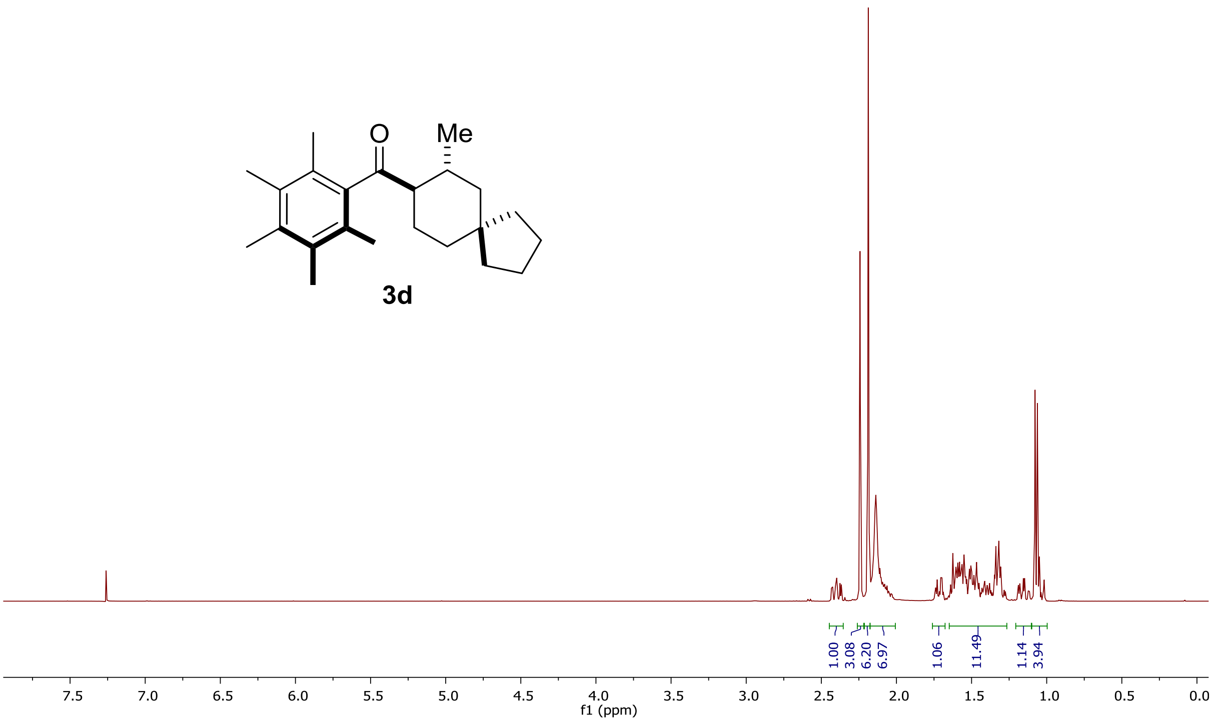
O
Me
3d
1.00
3.08
6.20
6.97
1.06
11.49
1.14
3.94
7.5 7.0 6.5 6.0 5.5 5.0 4.5 4.0 3.5 3.0 2.5 2.0 1.5 1.0 0.5 0.0
f1 (ppm)

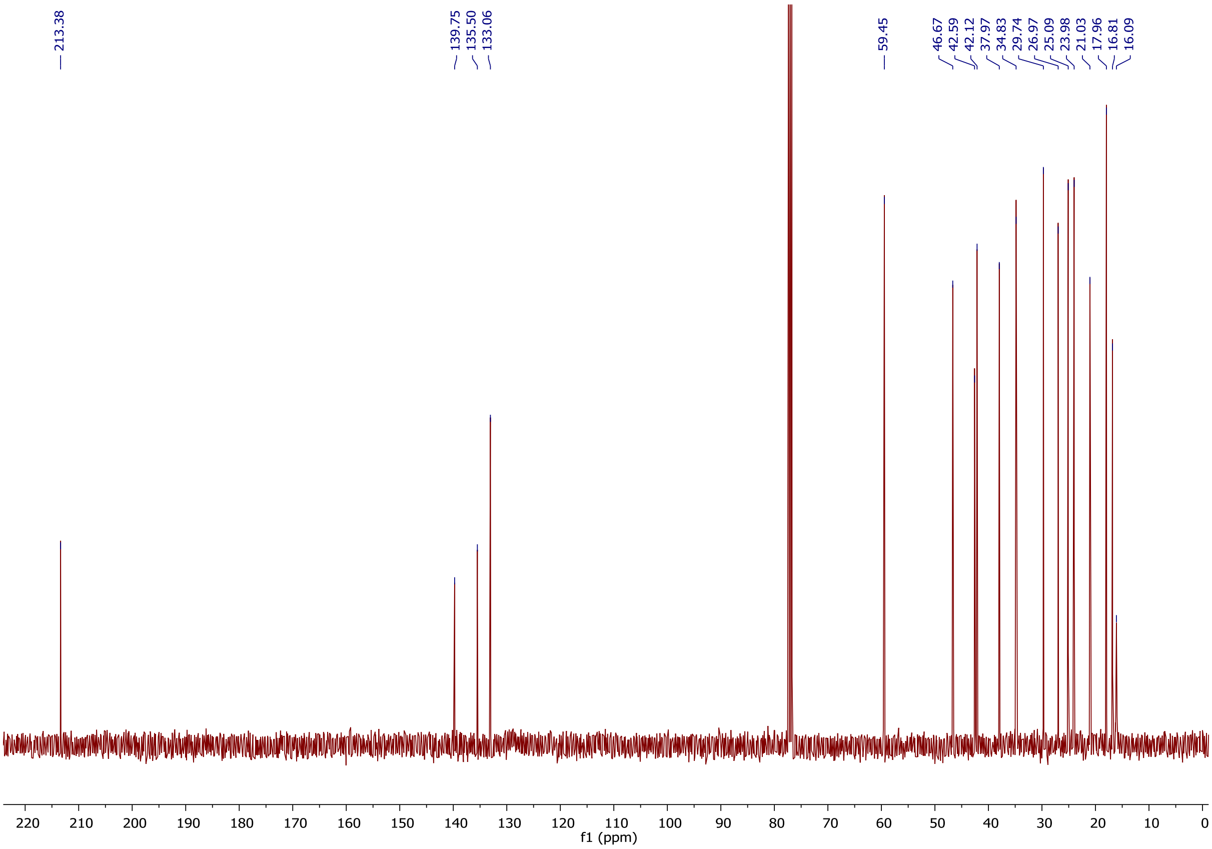
213.38
139.75
135.50
133.06
59.45
46.67
42.59
42.12
37.97
34.83
29.74
26.97
25.09
23.98
21.03
17.96
16.81
16.09
220 210 200 190 180 170 160 150 140 130 120 110 100 90 80 70 60 50 40 30 20 10 0
f1 (ppm)

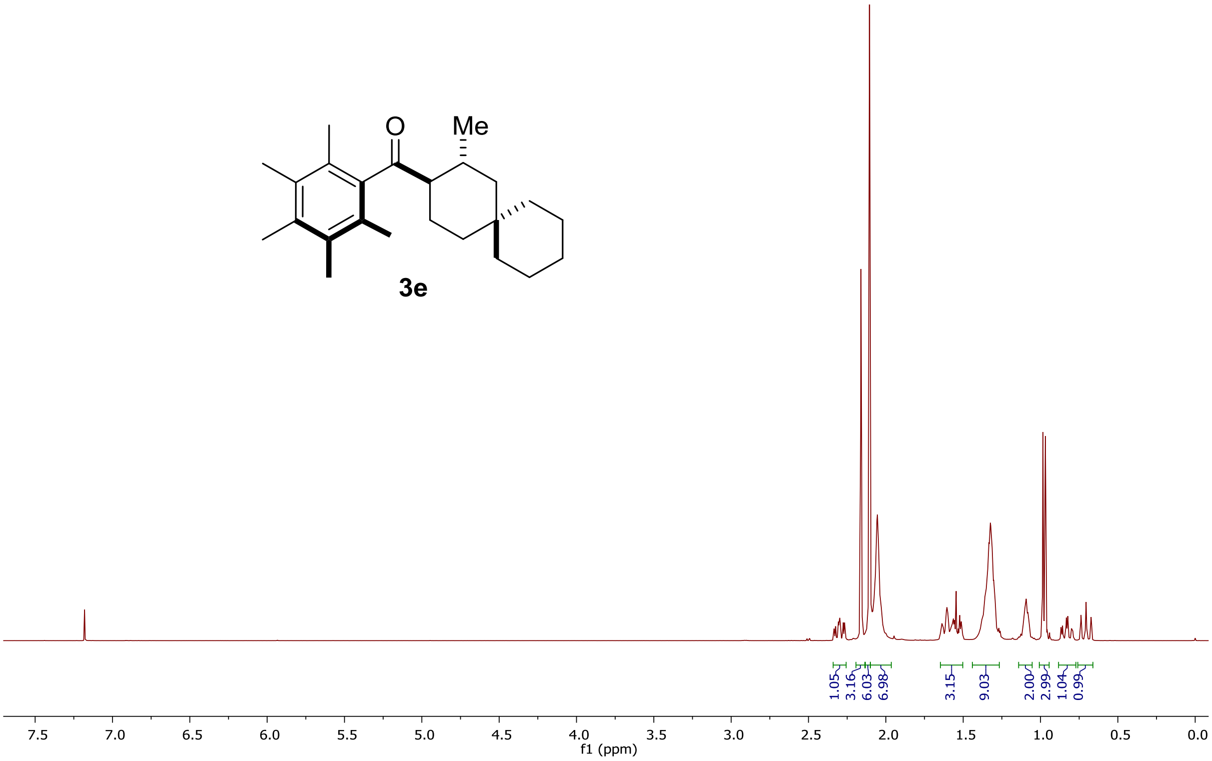
O
Me
3e
1.05
3.16
6.03
6.98
3.15
9.03
2.00
2.99
1.04
0.99
7.5 7.0 6.5 6.0 5.5 5.0 4.5 4.0 3.5 3.0 2.5 2.0 1.5 1.0 0.5 0.0
f1 (ppm)

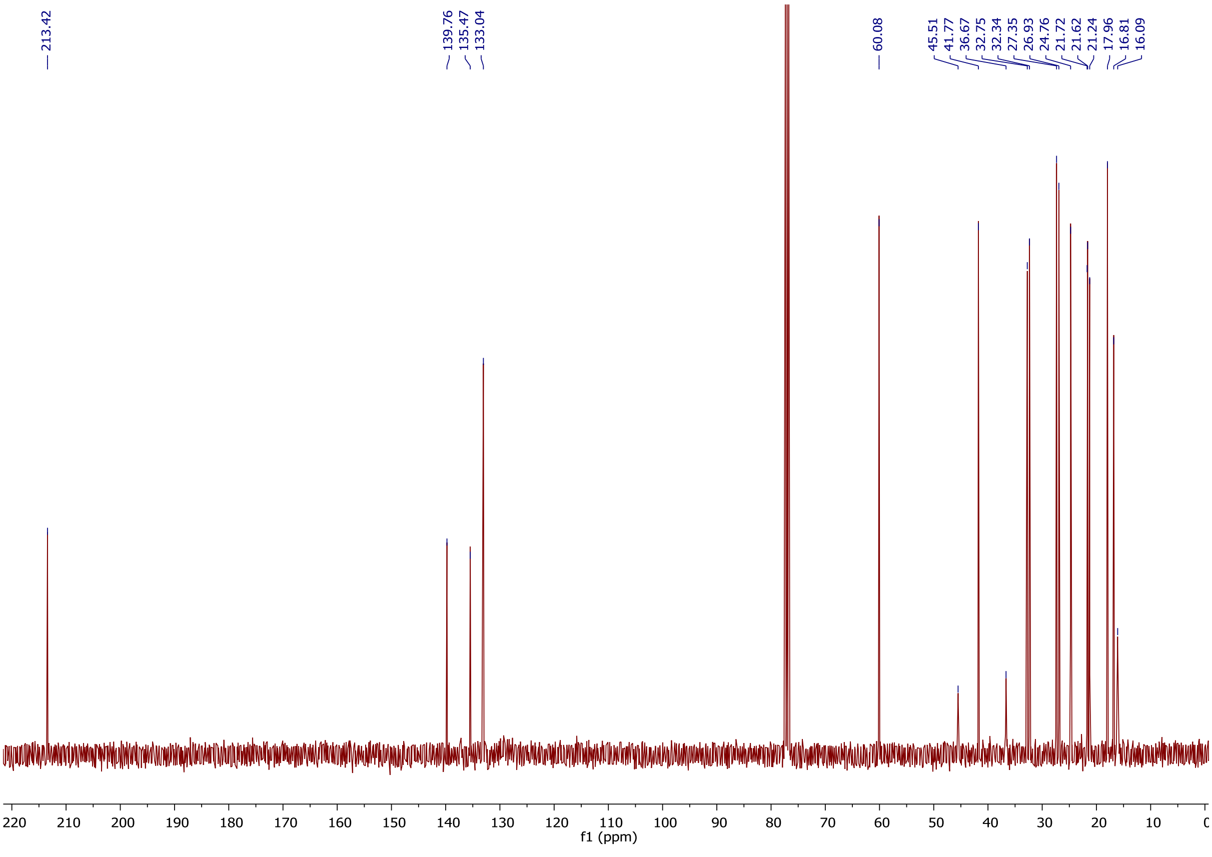
213.42
139.76
135.47
133.04
60.08
45.51
41.77
36.67
32.75
32.34
27.35
26.93
24.76
21.72
21.62
21.24
17.96
16.81
16.09
220 210 200 190 180 170 160 150 140 130 120 110 100 90 80 70 60 50 40 30 20 10
f1 (ppm)

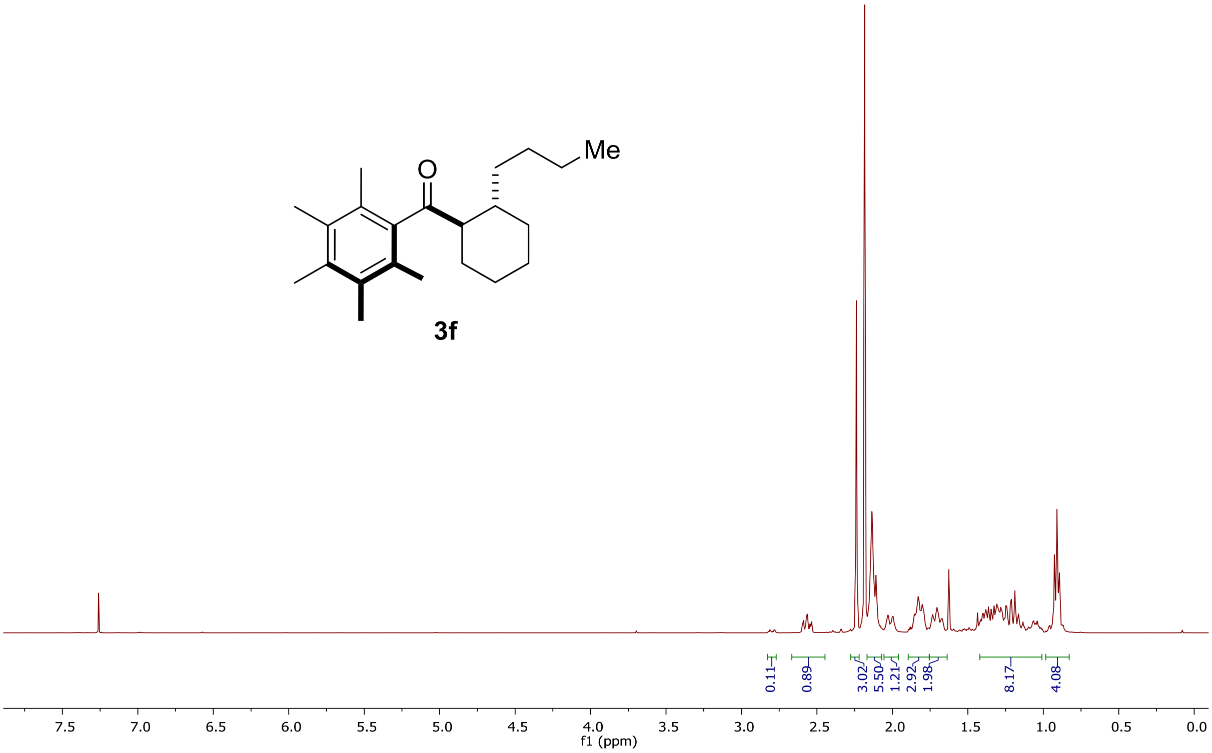

O
Me
3f
0.11
0.89
3.02
5.50
1.21
2.92
1.98
8.17
4.08
7.5 7.0 6.5 6.0 5.5 5.0 4.5 4.0 3.5 3.0 2.5 2.0 1.5 1.0 0.5 0.0
f1 (ppm)

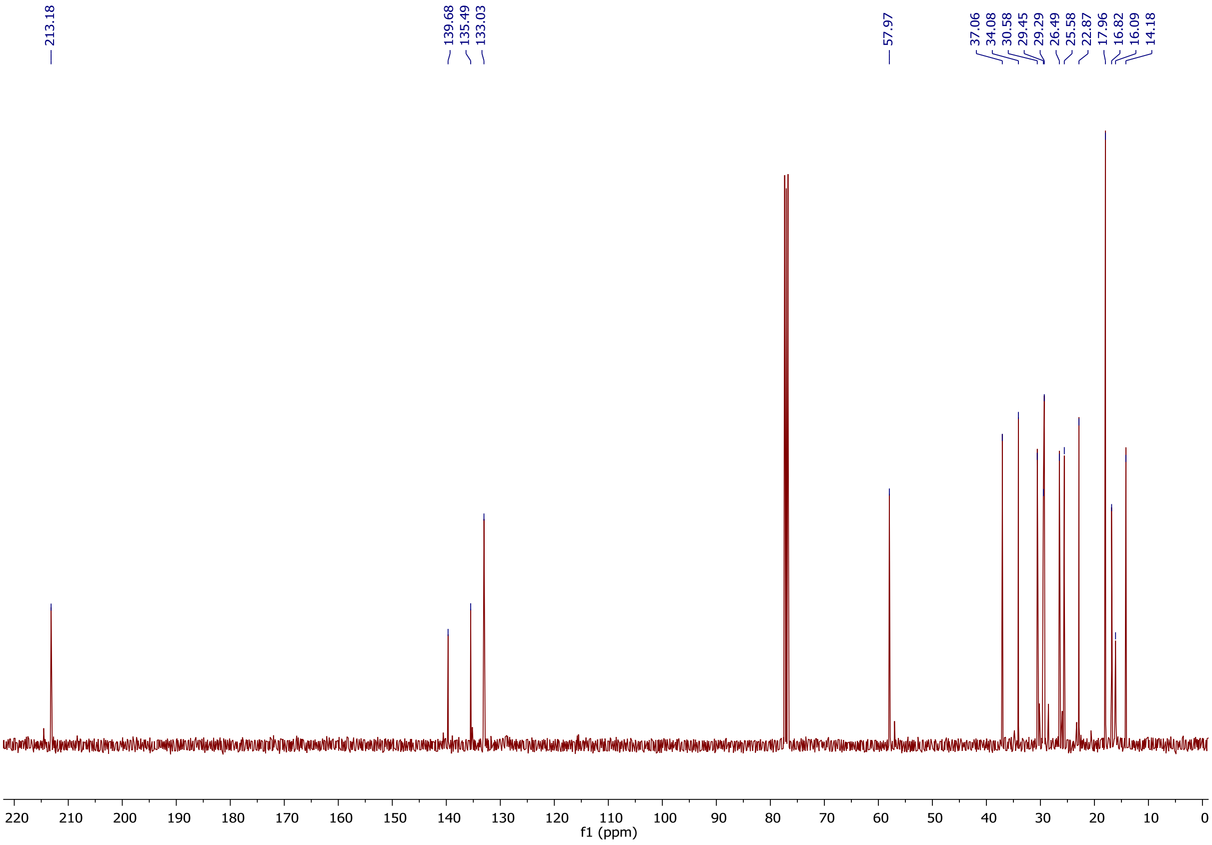

213.18
139.68
135.49
133.03
57.97
37.06
34.08
30.58
29.45
29.29
26.49
25.58
22.87
17.96
16.82
16.09
14.18
220 210 200 190 180 170 160 150 140 130 120 110 100 90 80 70 60 50 40 30 20 10 0
f1 (ppm)

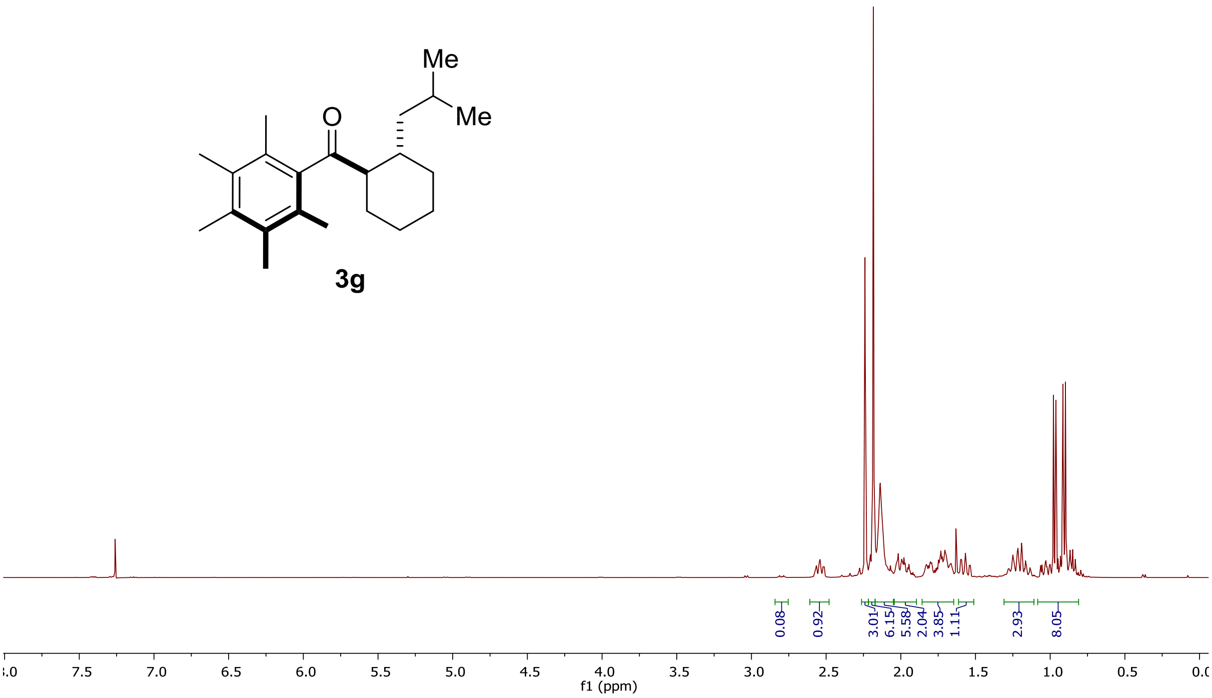

Me
Me
O
3g
0.08
0.92
3.01
6.15
5.58
2.04
3.85
1.11
2.93
8.05
f1 (ppm)

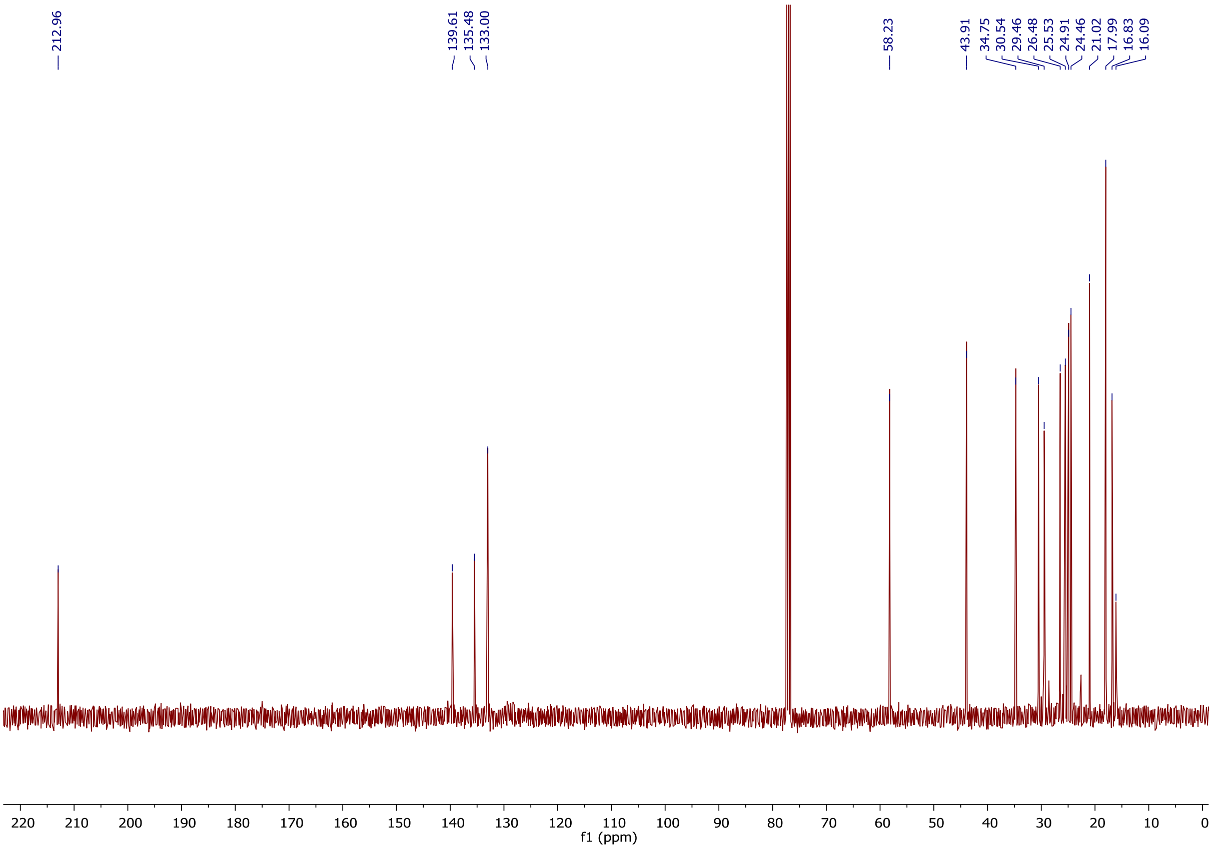

212.96
139.61
135.48
133.00
58.23
43.91
34.75
30.54
29.46
26.48
25.53
24.91
24.46
21.02
17.99
16.83
16.09
f1 (ppm)

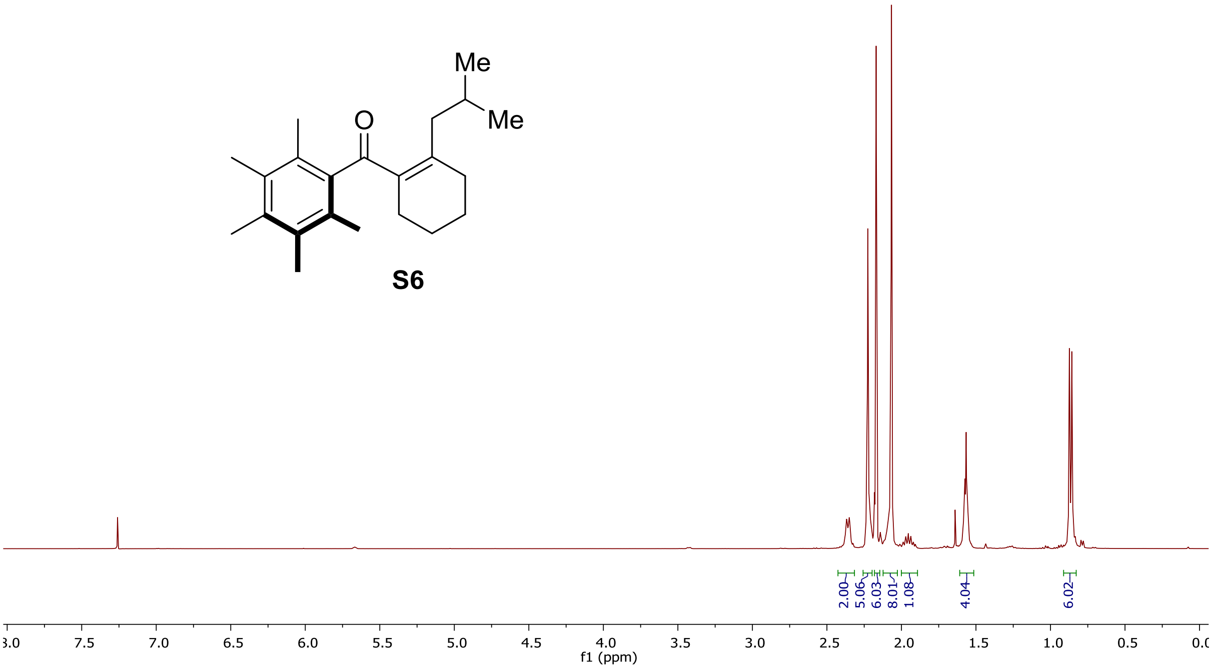

Me
O
Me
S6
2.00
5.06
6.03
8.01
1.08
4.04
6.02
f1 (ppm)

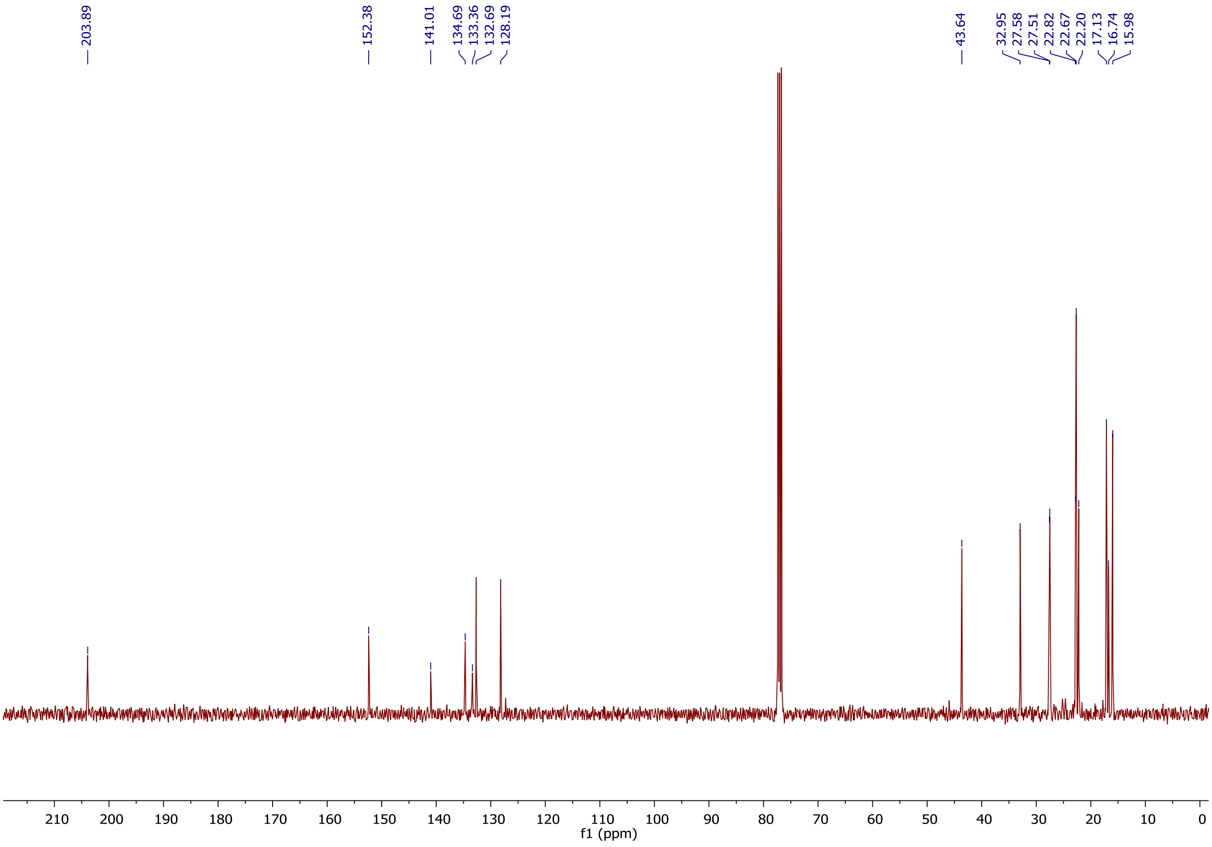

203.89
152.38
141.01
134.69
133.36
132.69
128.19
43.64
32.95
27.58
27.51
22.82
22.67
22.20
17.13
16.74
15.98
f1 (ppm)

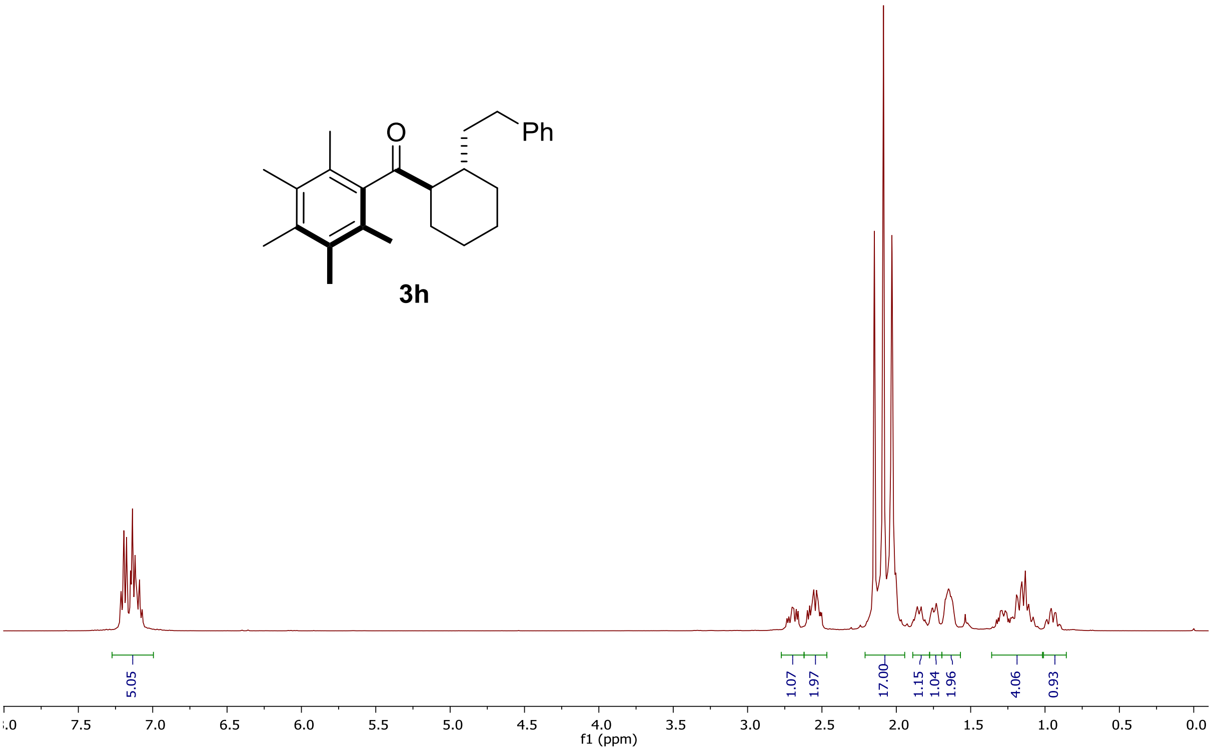
O
Ph
3h
5.05
1.07
1.97
17.00
1.15
1.04
1.96
4.06
0.93
f1 (ppm)

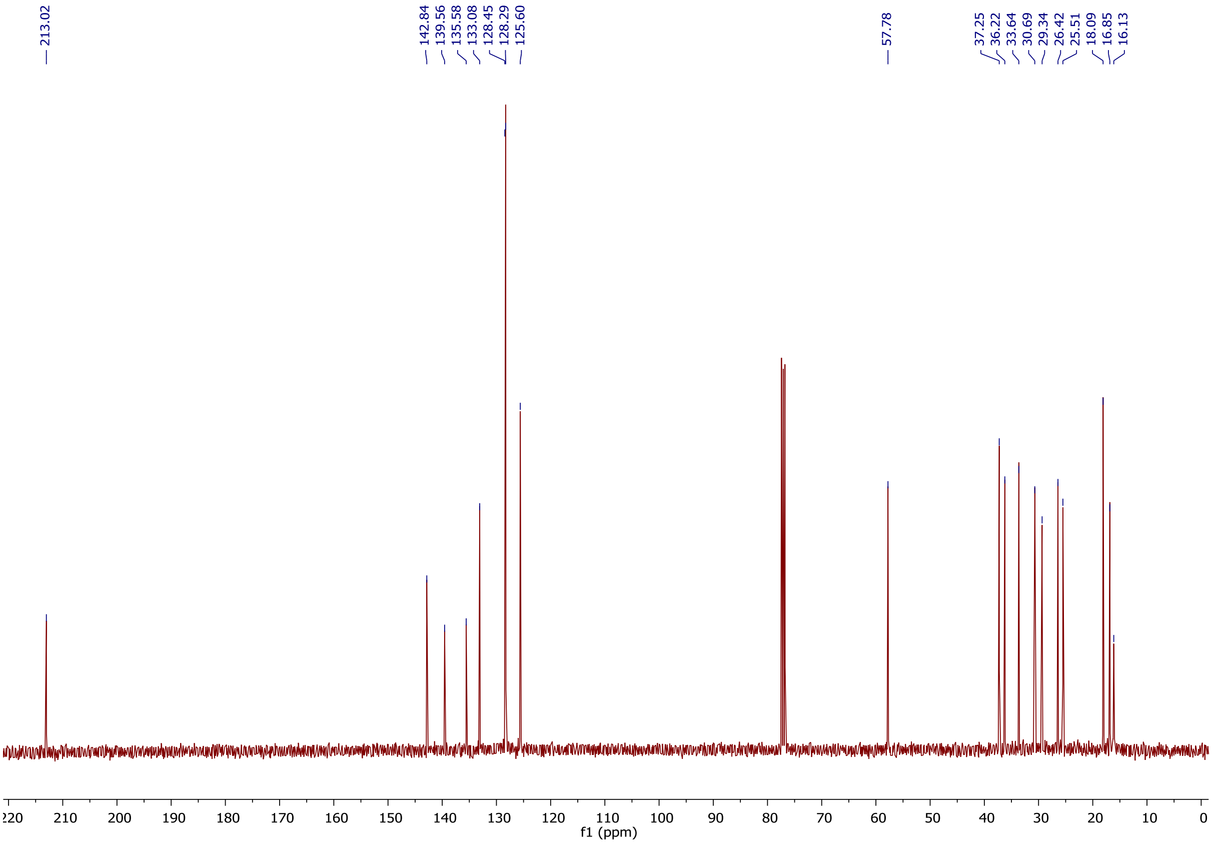
213.02
142.84
139.56
135.58
133.08
128.45
128.29
125.60
57.78
37.25
36.22
33.64
30.69
29.34
26.42
25.51
18.09
16.85
16.13
f1 (ppm)

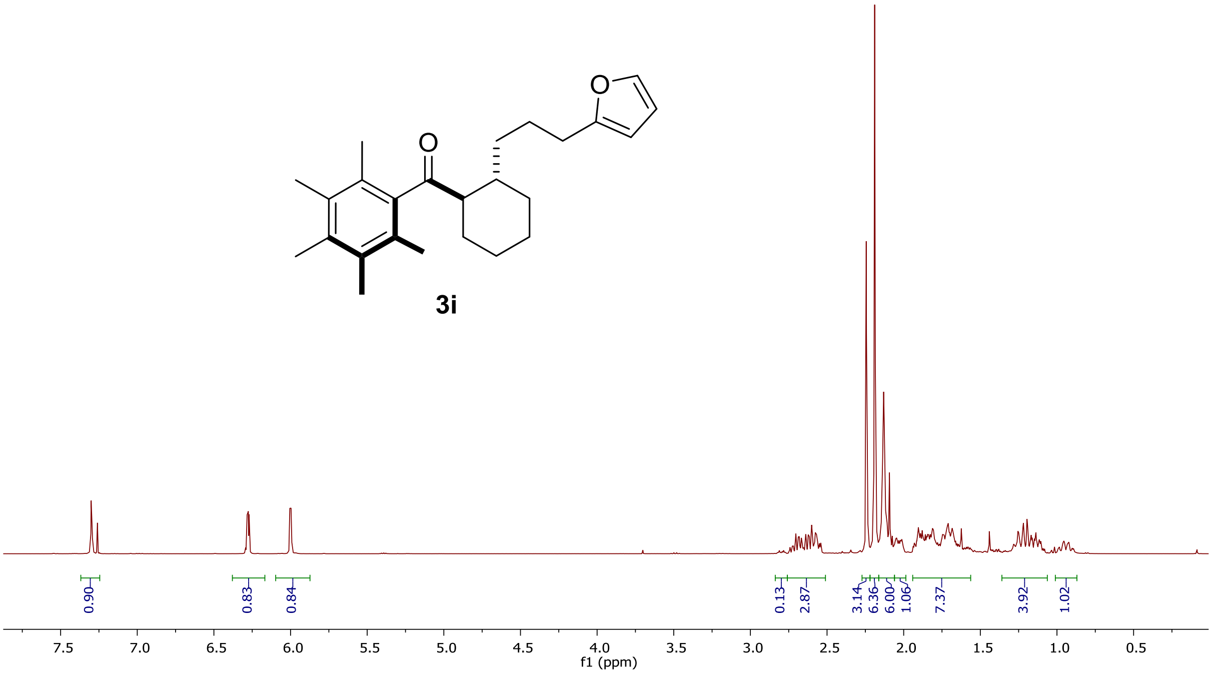
O
O
3i
0.90
0.83
0.84
0.13
2.87
3.14
6.36
6.00
1.06
7.37
3.92
1.02
7.5 7.0 6.5 6.0 5.5 5.0 4.5 4.0 3.5 3.0 2.5 2.0 1.5 1.0 0.5
f1 (ppm)

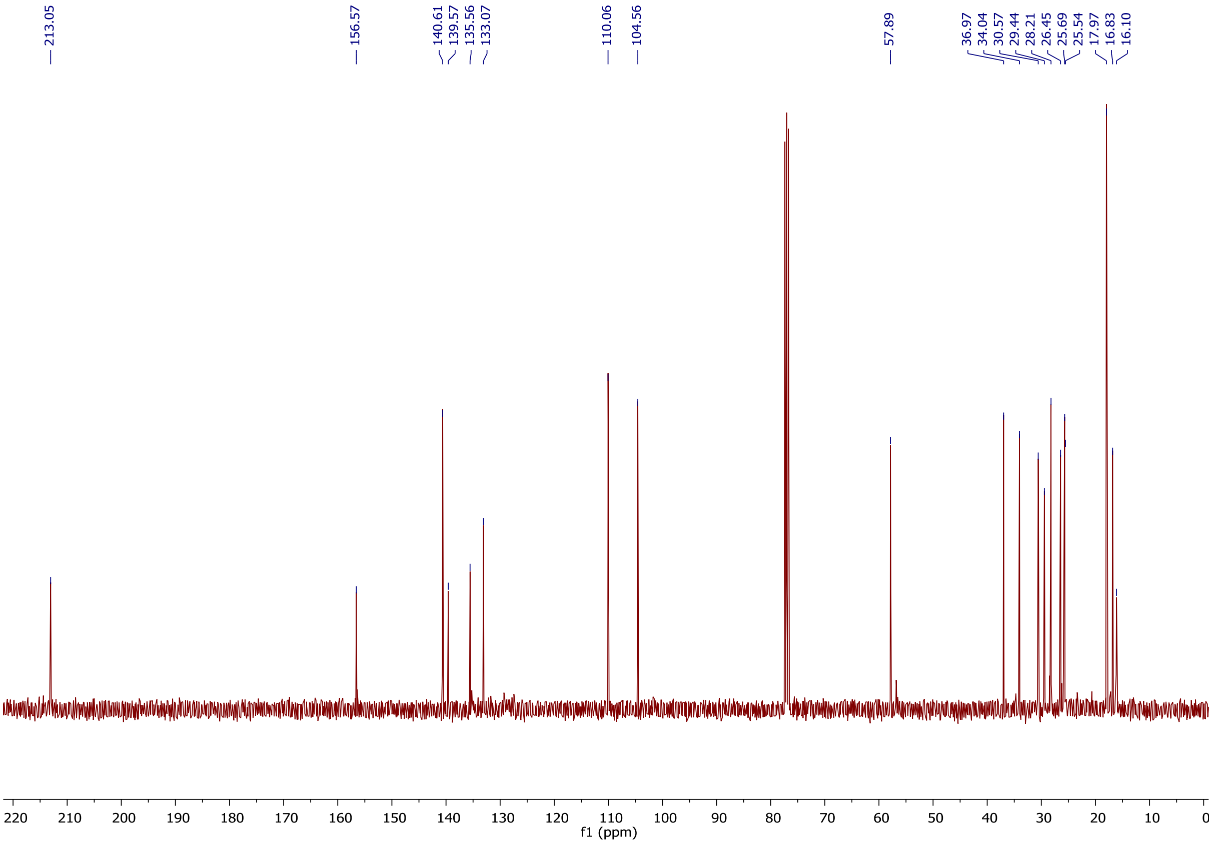
213.05
156.57
140.61
139.57
135.56
133.07
110.06
104.56
57.89
36.97
34.04
30.57
29.44
28.21
26.45
25.69
25.54
17.97
16.83
16.10
220 210 200 190 180 170 160 150 140 130 120 110 100 90 80 70 60 50 40 30 20 10 0
f1 (ppm)

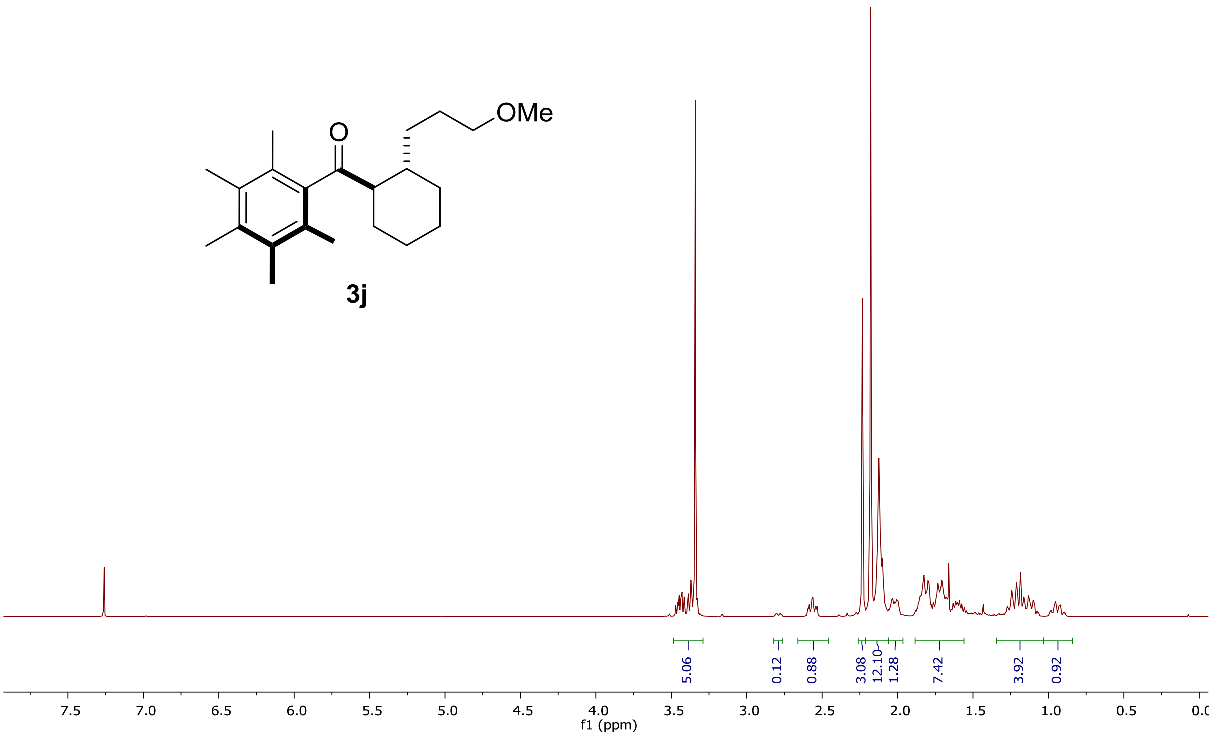
O
OMe
3j
5.06
0.12
0.88
3.08
12.10
1.28
7.42
3.92
0.92
7.5
7.0
6.5
6.0
5.5
5.0
4.5
4.0
3.5
3.0
2.5
2.0
1.5
1.0
0.5
f1 (ppm)

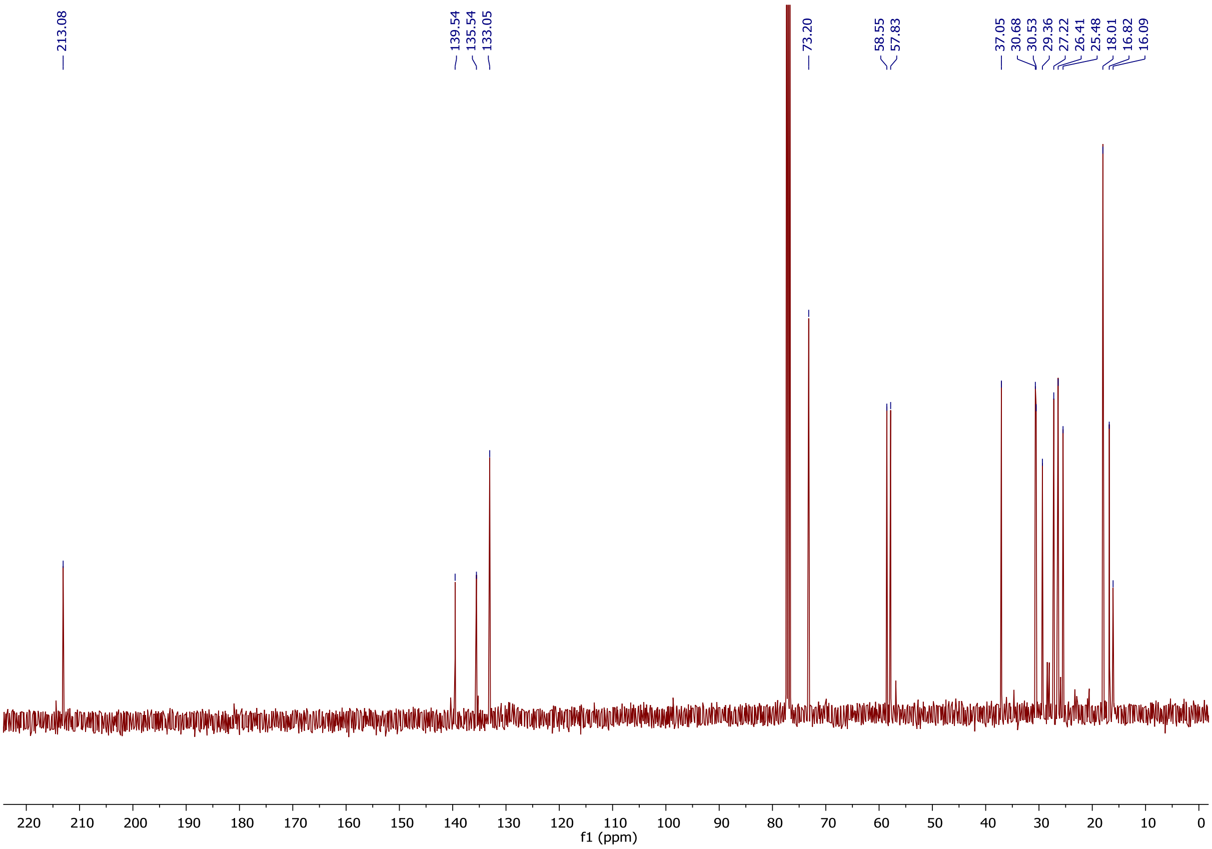
213.08
139.54
135.54
133.05
73.20
58.55
57.83
37.05
30.68
30.53
29.36
27.22
26.41
25.48
18.01
16.82
16.09
220
210
200
190
180
170
160
150
140
130
120
110
100
90
80
70
60
50
40
30
20
10
0
f1 (ppm)

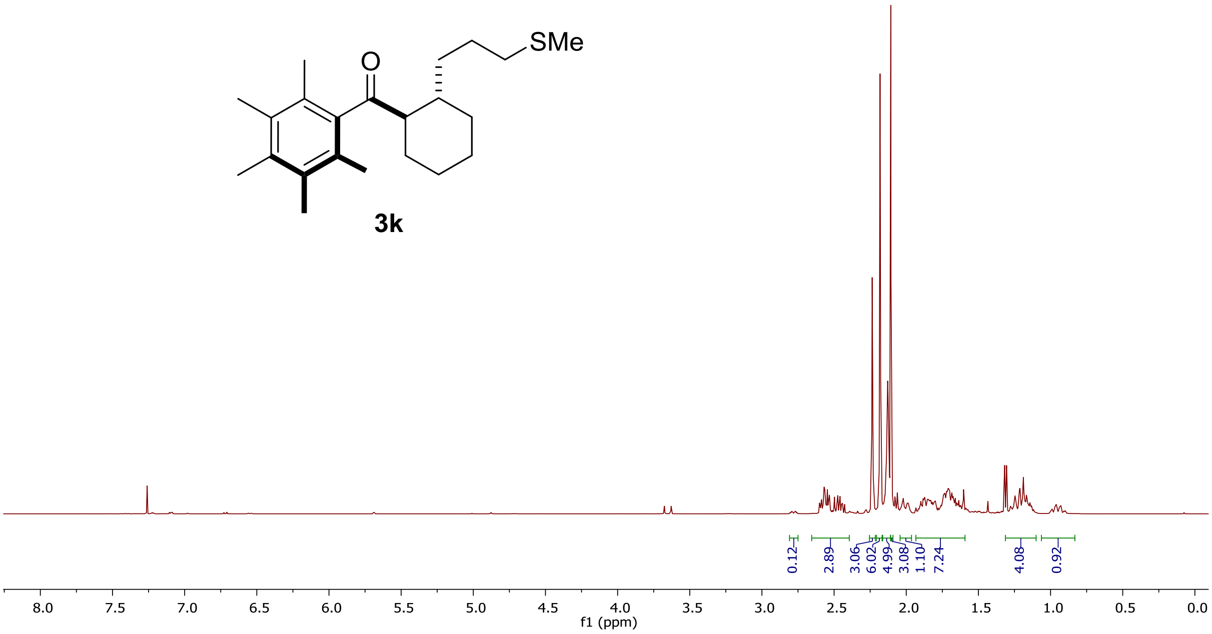

O
SMe
3k
0.12
2.89
3.06
6.02
4.99
3.08
1.10
7.24
4.08
0.92
f1 (ppm)

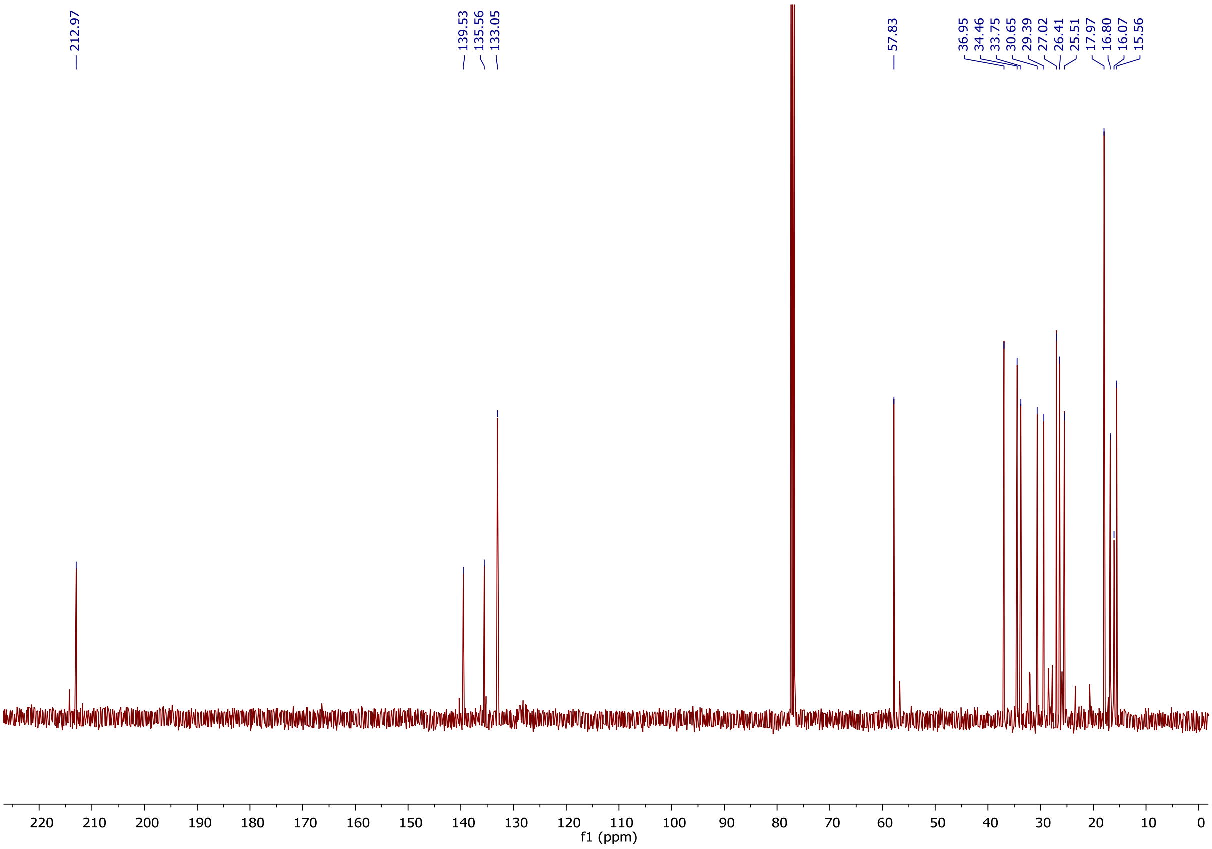

212.97
139.53
135.56
133.05
57.83
36.95
34.46
33.75
30.65
29.39
27.02
26.41
25.51
17.97
16.80
16.07
15.56
f1 (ppm)

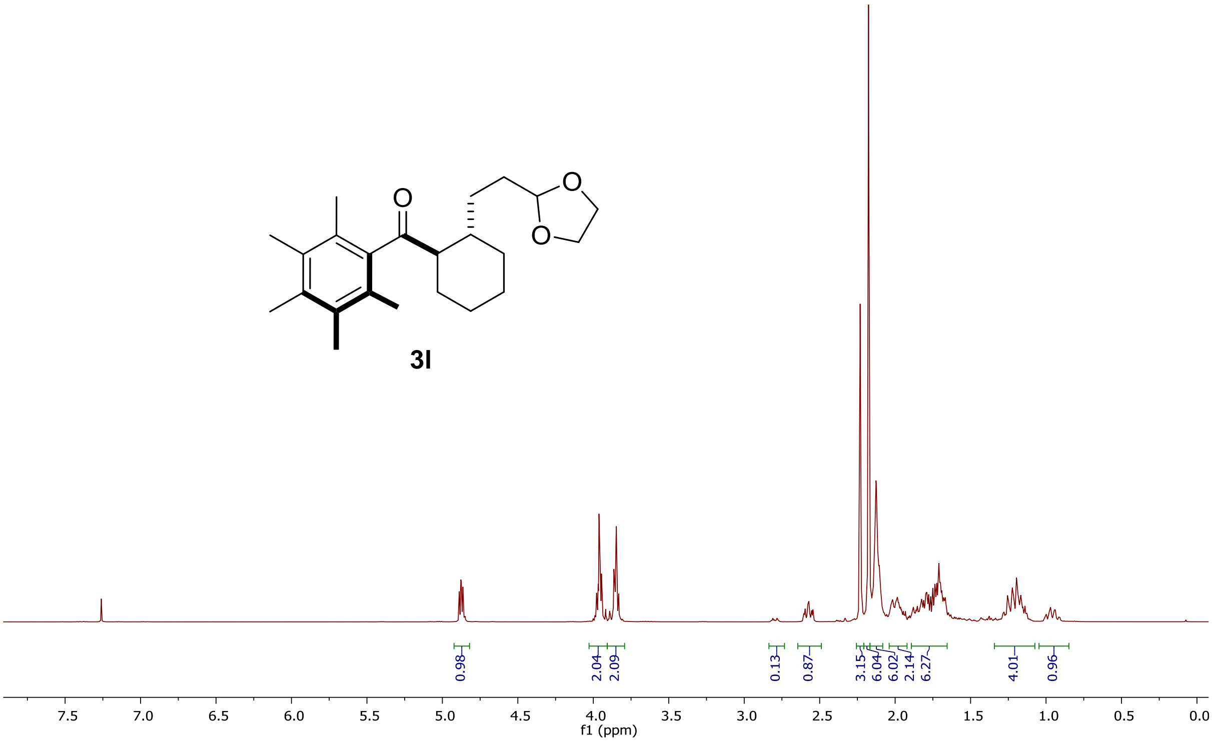
O
O
O
3l
0.98
2.04
2.09
0.13
0.87
3.15
6.04
6.02
2.14
6.27
4.01
0.96
7.5 7.0 6.5 6.0 5.5 5.0 4.5 4.0 3.5 3.0 2.5 2.0 1.5 1.0 0.5 0.0
f1 (ppm)

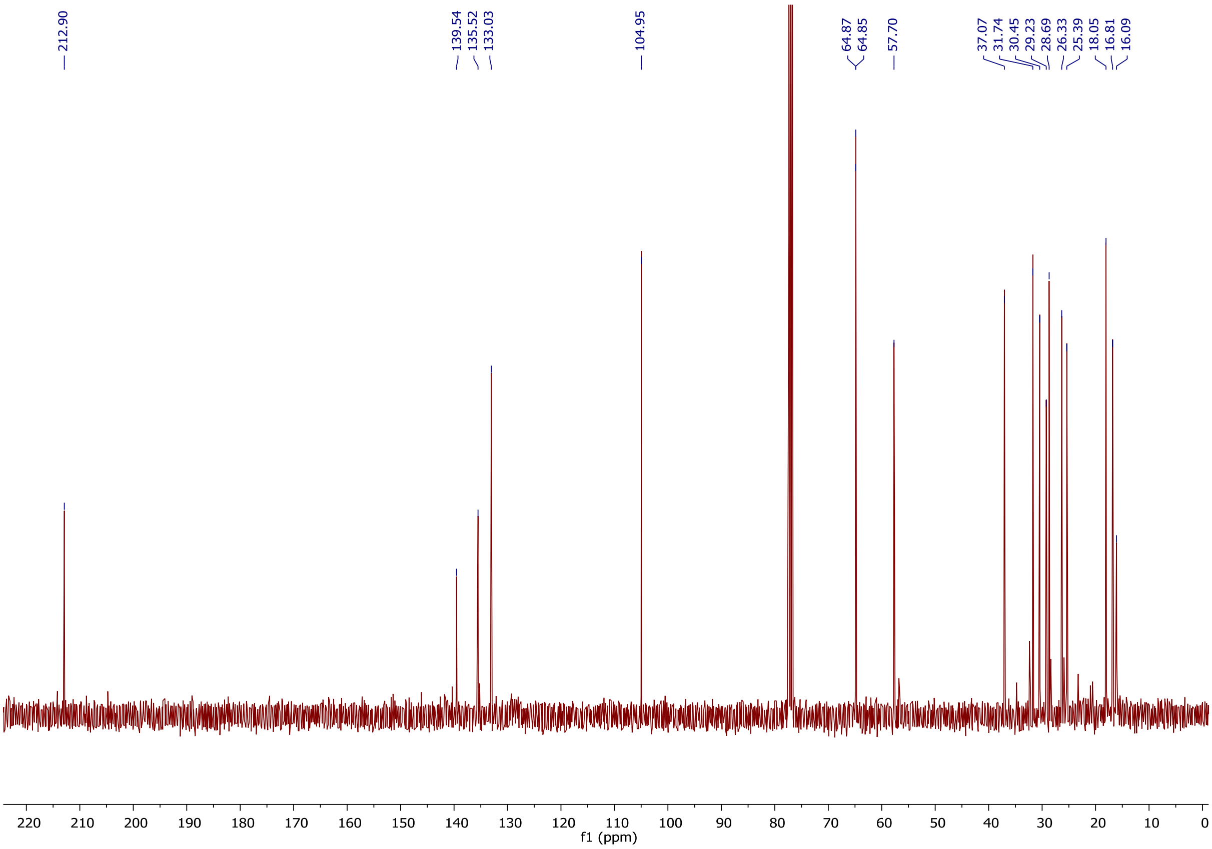
212.90
139.54
135.52
133.03
104.95
64.87
64.85
57.70
37.07
31.74
30.45
29.23
28.69
26.33
25.39
18.05
16.81
16.09
220 210 200 190 180 170 160 150 140 130 120 110 100 90 80 70 60 50 40 30 20 10 0
f1 (ppm)

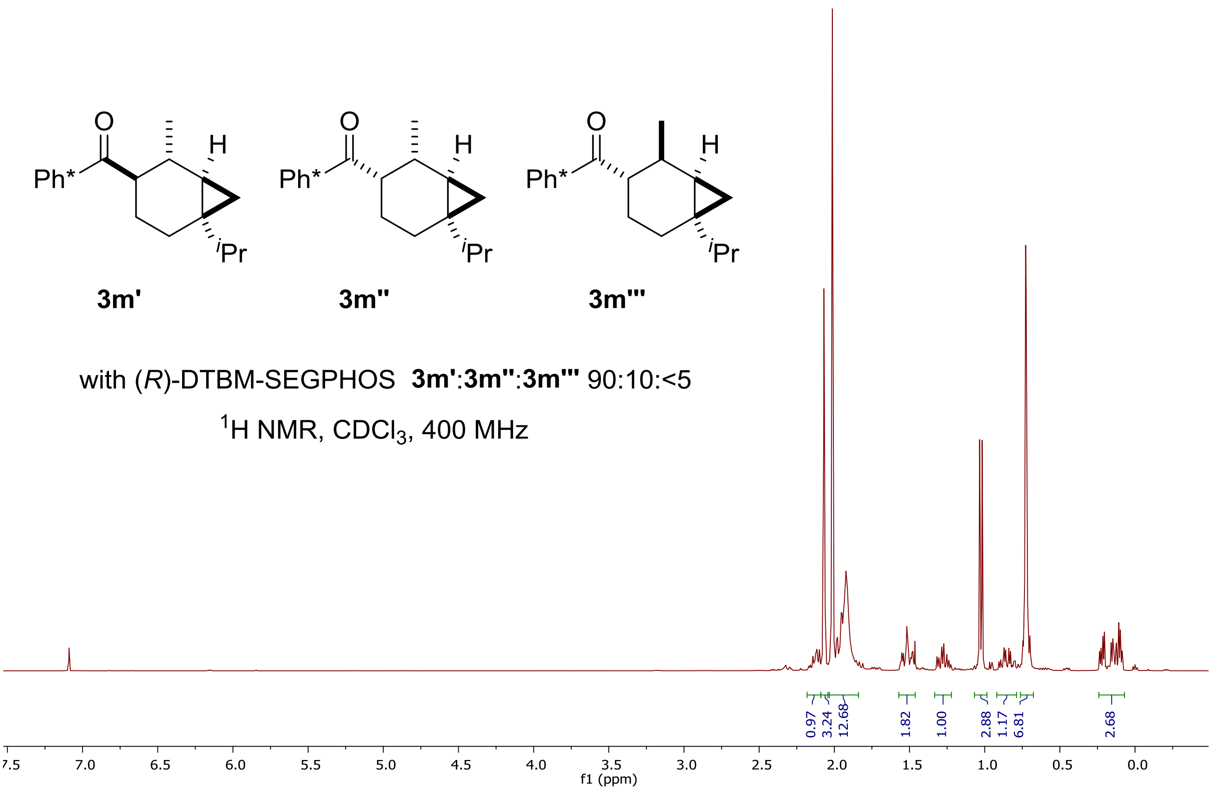
3m'
3m''
3m'''
with (R)-DTBM-SEGPHOS 3m':3m'':3m''' 90:10:<5
1H NMR, CDCl3, 400 MHz
0.97
3.24
12.68
1.82
1.00
2.88
1.17
6.81
2.68
f1 (ppm)

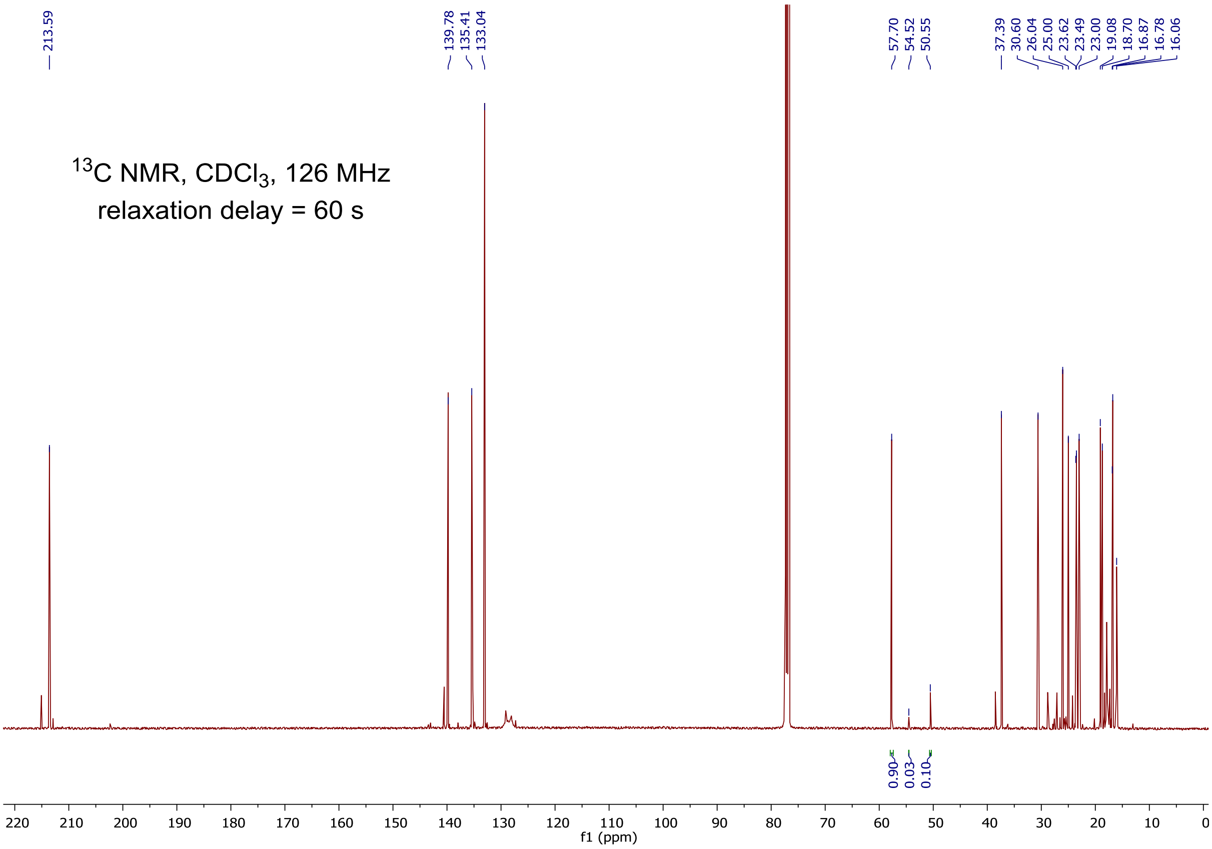
213.59
139.78
135.41
133.04
57.70
54.52
50.55
37.39
30.60
26.04
25.00
23.62
23.49
23.00
19.08
18.70
16.87
16.78
16.06
13C NMR, CDCl3, 126 MHz
relaxation delay = 60 s
0.90
0.03
0.10
f1 (ppm)

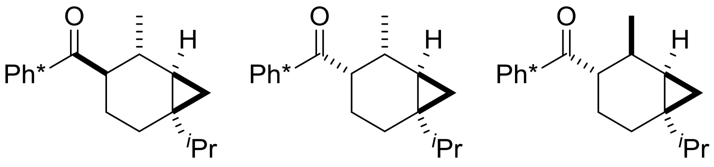

3m' **3m''** **3m'''**

with (*S*)-DTBM-SEGPHOS **3m':3m'':3m'''** 58:5:37

^{1}H NMR, $CDCl_3$, 400 MHz

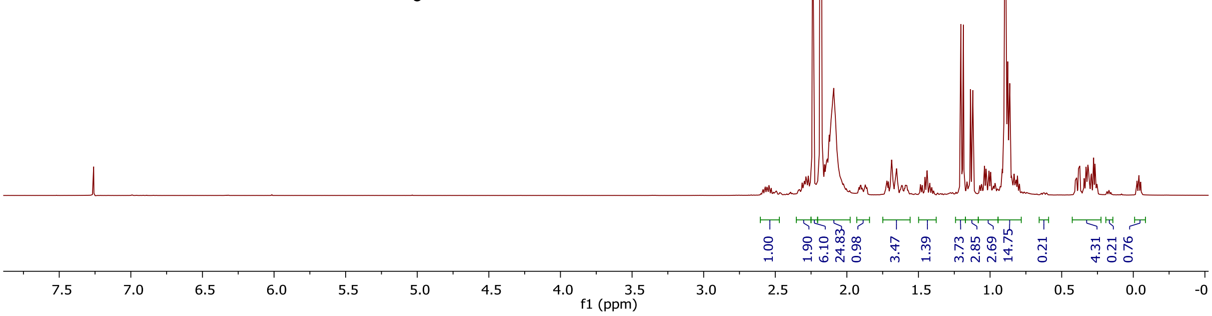

^{13}C NMR, $CDCl_3$, 126 MHz

relaxation delay = 60 s

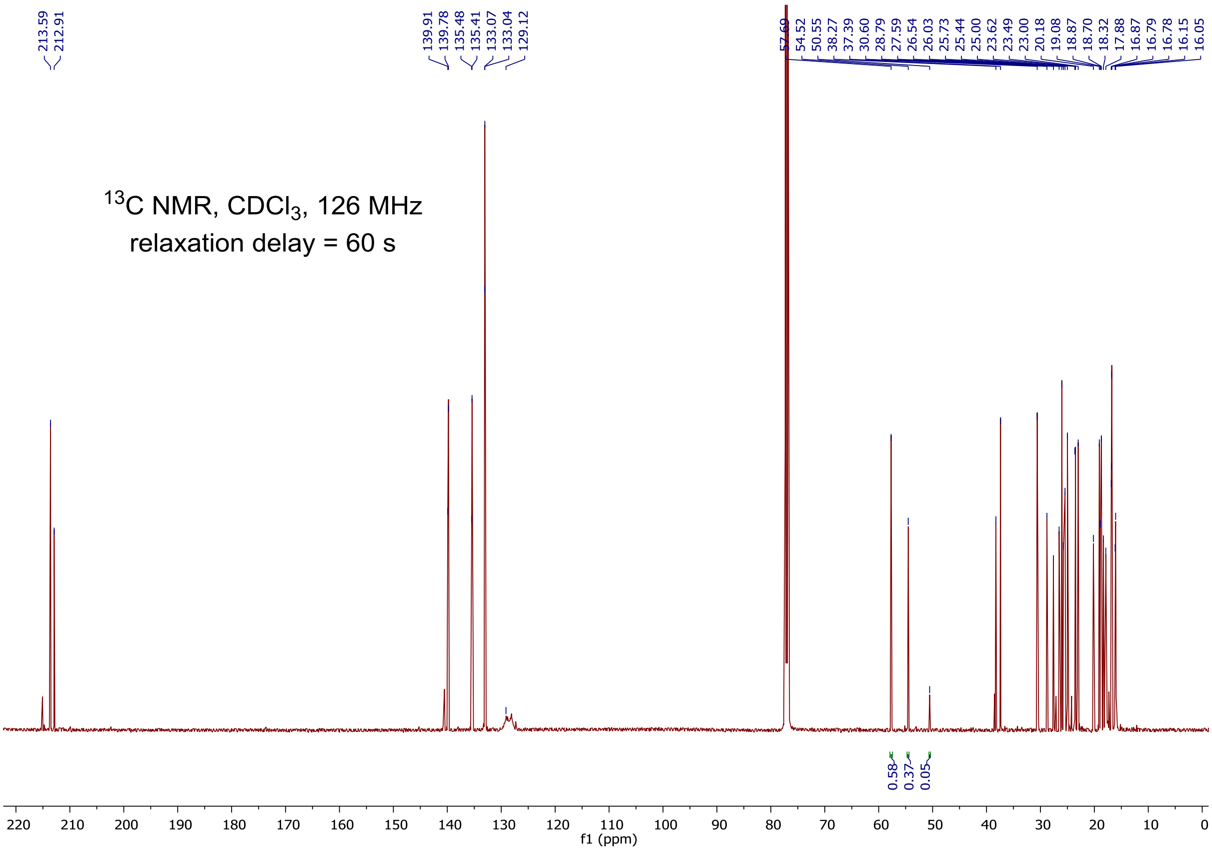

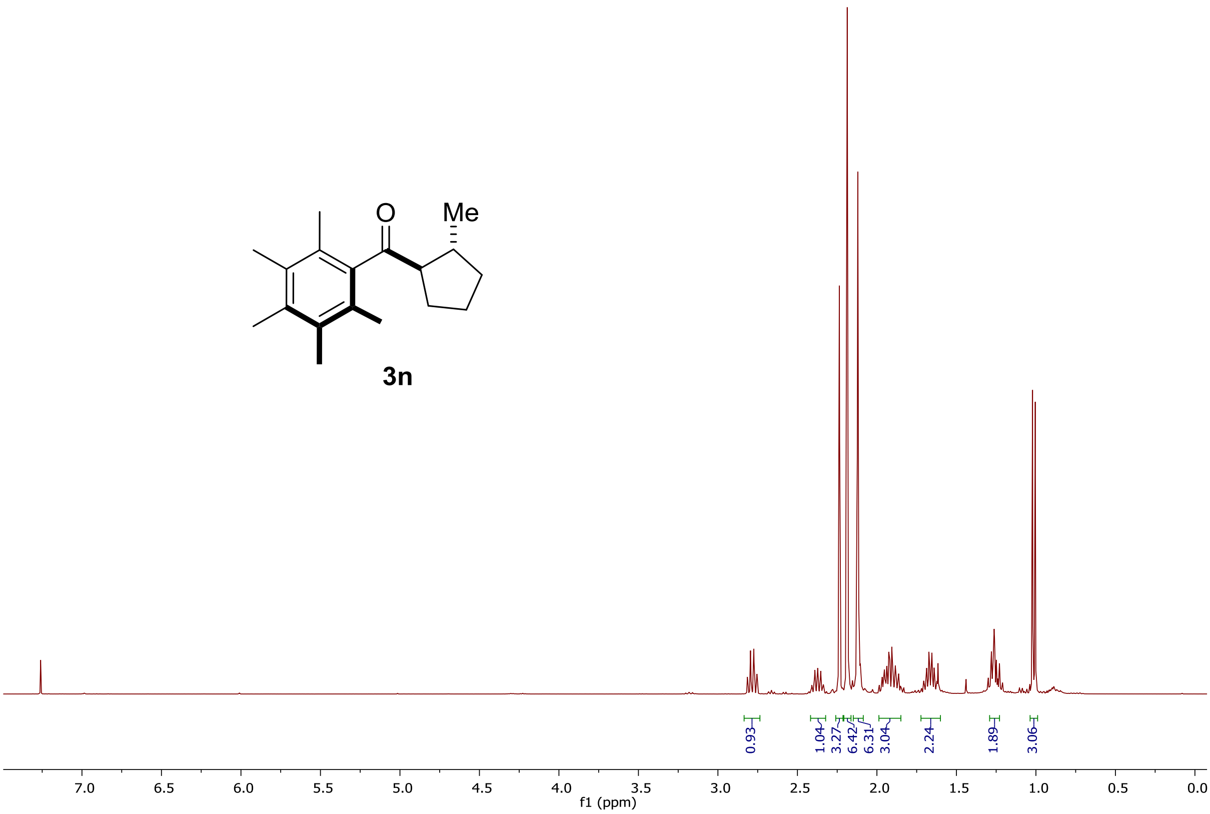
O
Me
0.93
1.04
3.27
6.42
6.31
3.04
2.24
1.89
3.06
7.0 6.5 6.0 5.5 5.0 4.5 4.0 3.5 3.0 2.5 2.0 1.5 1.0 0.5 0.0
f1 (ppm)

3n

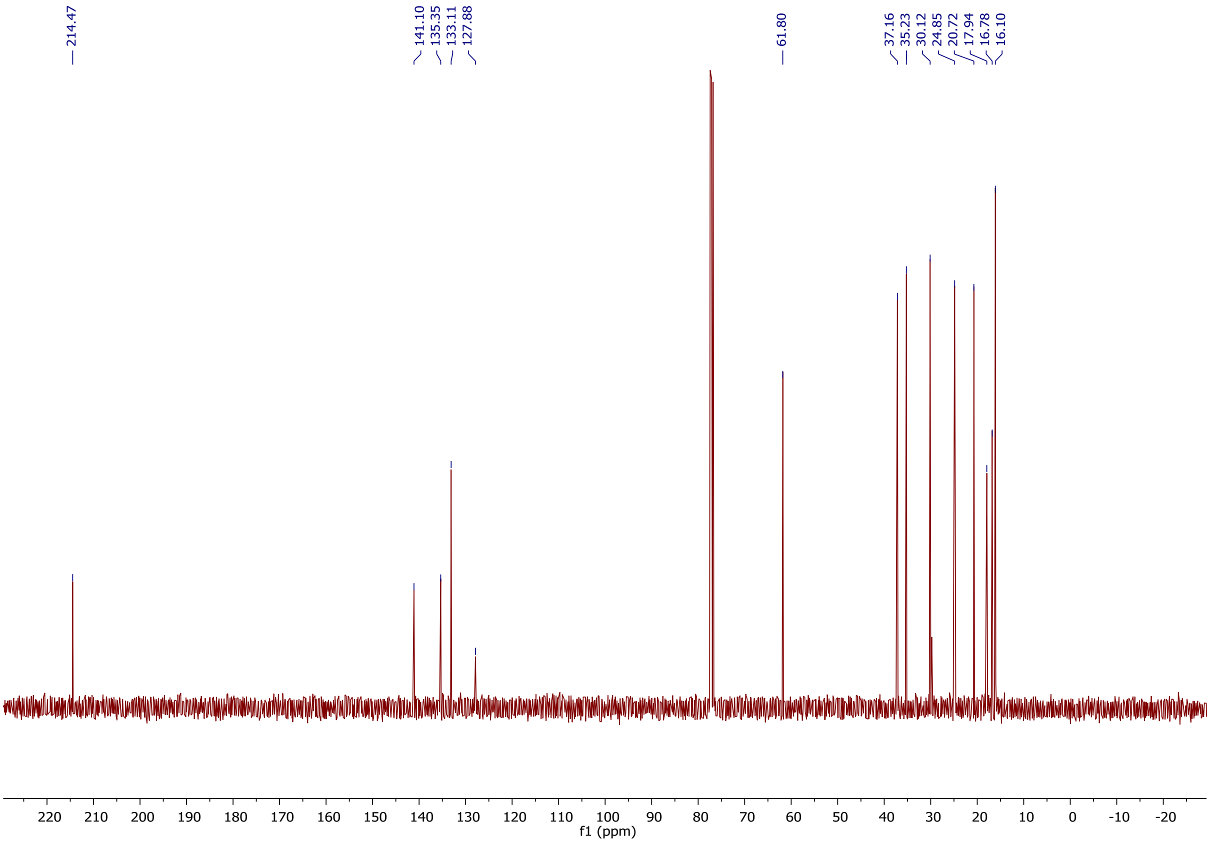
214.47
141.10
135.35
133.11
127.88
61.80
37.16
35.23
30.12
24.85
20.72
17.94
16.78
16.10
220 210 200 190 180 170 160 150 140 130 120 110 100 90 80 70 60 50 40 30 20 10 0 -10 -20
f1 (ppm)

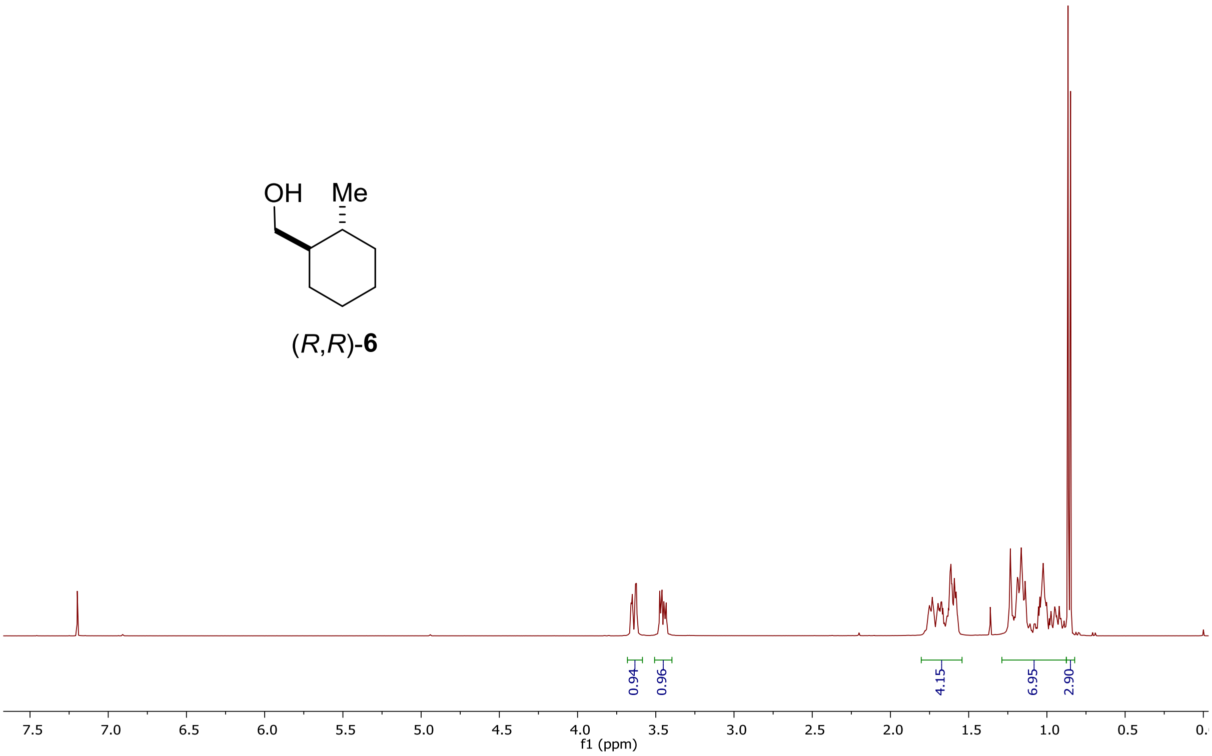
OH
Me
0.94
0.96
4.15
6.95
2.90
7.5
7.0
6.5
6.0
5.5
5.0
4.5
4.0
3.5
3.0
2.5
2.0
1.5
1.0
0.5
f1 (ppm)

(*R*,*R*)-**6**

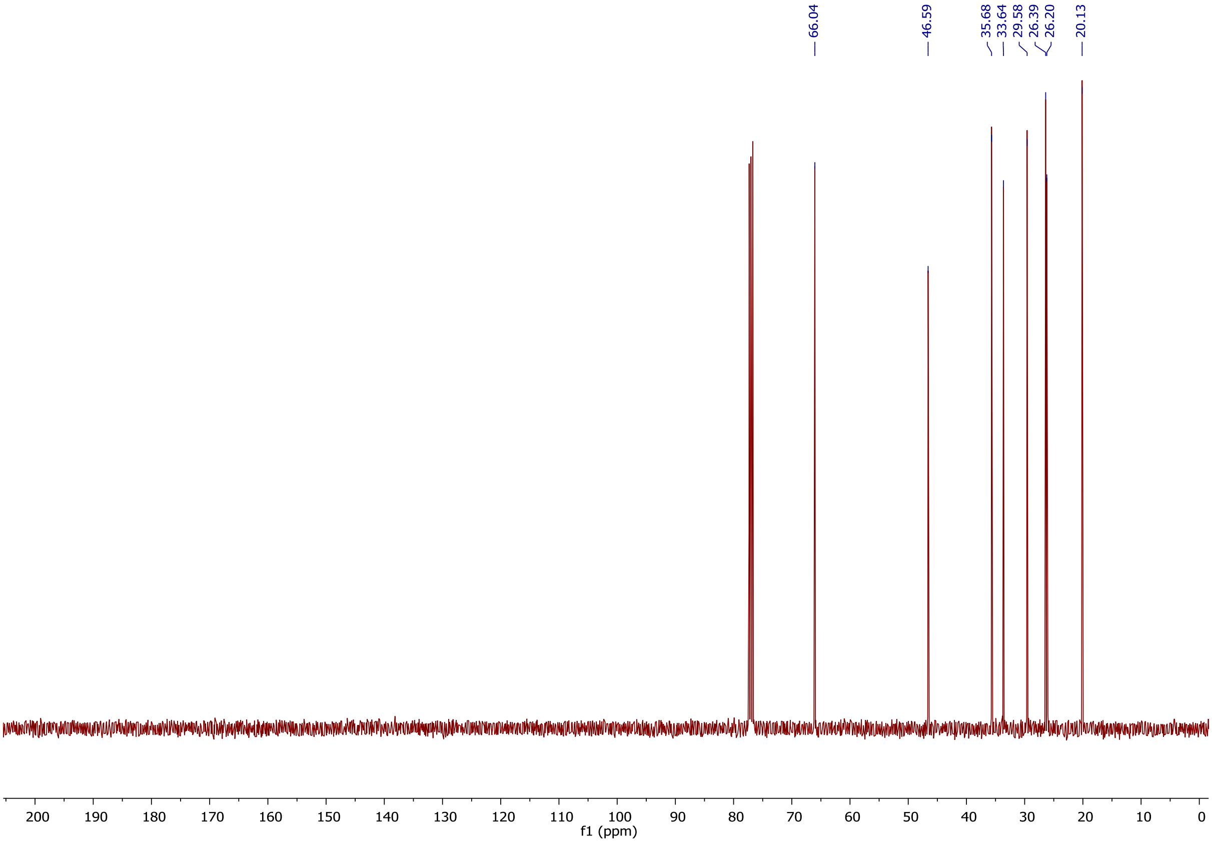
66.04
46.59
35.68
33.64
29.58
26.39
26.20
20.13
200
190
180
170
160
150
140
130
120
110
100
90
80
70
60
50
40
30
20
10
0
f1 (ppm)
